# Supplementary material for: Proteomic and metagenomic insights into prehistoric Spanish Levantine Rock Art
Source: Sci Rep. 2018 Jul 3;8:10011. doi: 10.1038/s41598-018-28121-6 (PMC6030215; doi:10.1038/s41598-018-28121-6)
Supplement: Supplementary file 1 — Supplementary Information [file 41598_2018_28121_MOESM1_ESM.pdf]

Proteomic and metagenomic insights into prehistoric Spanish Levantine Rock Art

Clodoaldo Roldán[1], Sonia Murcia-Mascarós[1*], Esther López-Montalvo[2], Cristina Vilanova[3] and Manuel Porcar[3,4]

1 Materials Science Institute of the University of Valencia (ICMUV), Catedrático José Beltrán 2, 46980 Paterna, Valencia, Spain

2 UMR 5608 TRACES, French National Center for the Scientific Research (CNRS) - University of Toulouse 2-Jean Jaurès. 5, Allée Antonio Machado, 31058 Toulouse, France.

3 Darwin Bioprospecting Excellence, SL., Parc Cientific Universitat de València, 46980 Paterna, Valencia, Spain

4 Institute for Integrative Systems Biology (I2SysBio, Universitat de València-CSIC). Parc Cientific Universitat de València, 46980 Paterna, Valencia, Spain.

*Correspondence to sonia.mascaros@uv.es.

Supplementary tables and figures

Supplementary Table S1 | Summary of sequencing statistics.

Sample	Raw reads				Trimmed reads			
	Num. Seq	Av. Length	Total Mb	Av. Qual	Num. Seq	Av. Length	Total Mb	Av. Qual
**CSI-01**	51266	457,33	23,45	36,92	50728	433,67	22	37,11
**CSI-04**	62185	452,38	28,13	35,6	61517	429,1	26,4	37,22
**CSVI-01**	103839	456,08	47,36	37,02	102928	433,27	44,6	37,22
**CRU**	75932	442,62	33,61	36,98	75250	419,75	31,59	37,24
**COL**	75151	446,41	33,55	36,81	74332	423,27	31,46	37,04

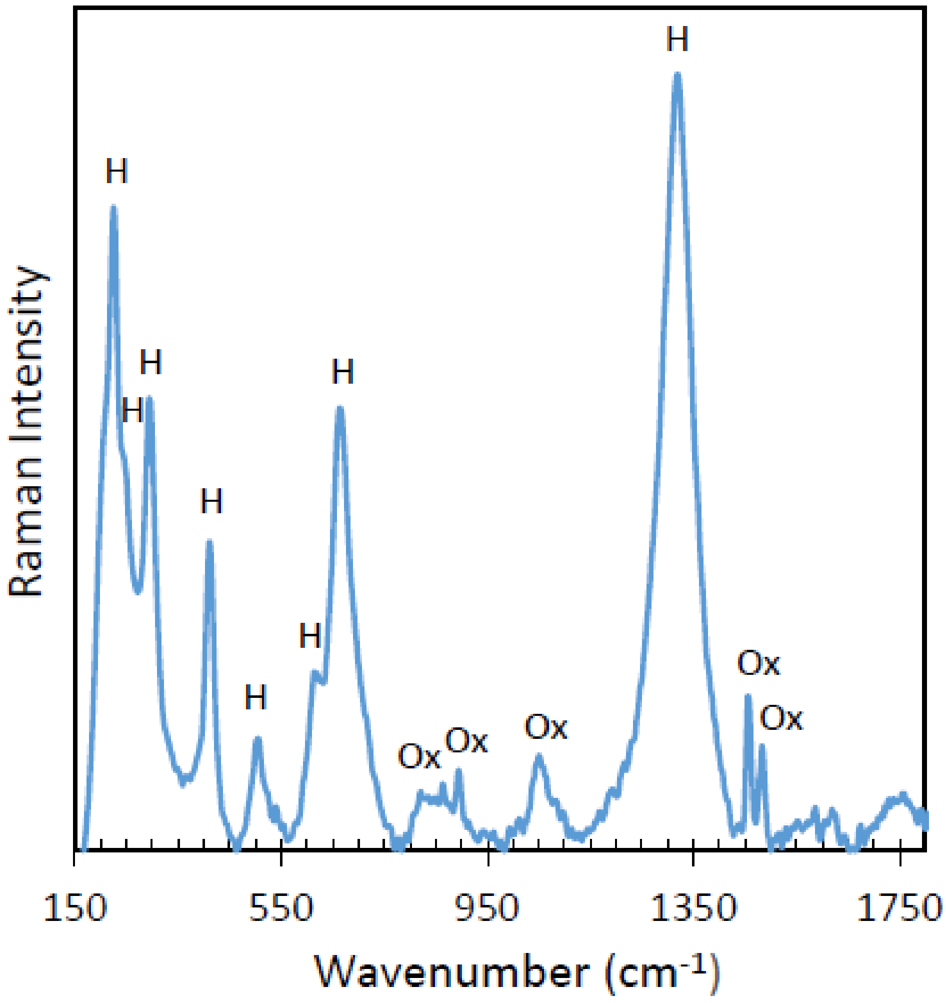

Supplementary Figure S1 | Raman spectrum obtained from sample CSV-02. The bands indicated by H, belong to Hematite iron oxide phase (Fe2O3) and the bands indicated by Ox, correspond to the whewellite phase (calcium oxalate monohydrate, CaC2O4 • H2O).

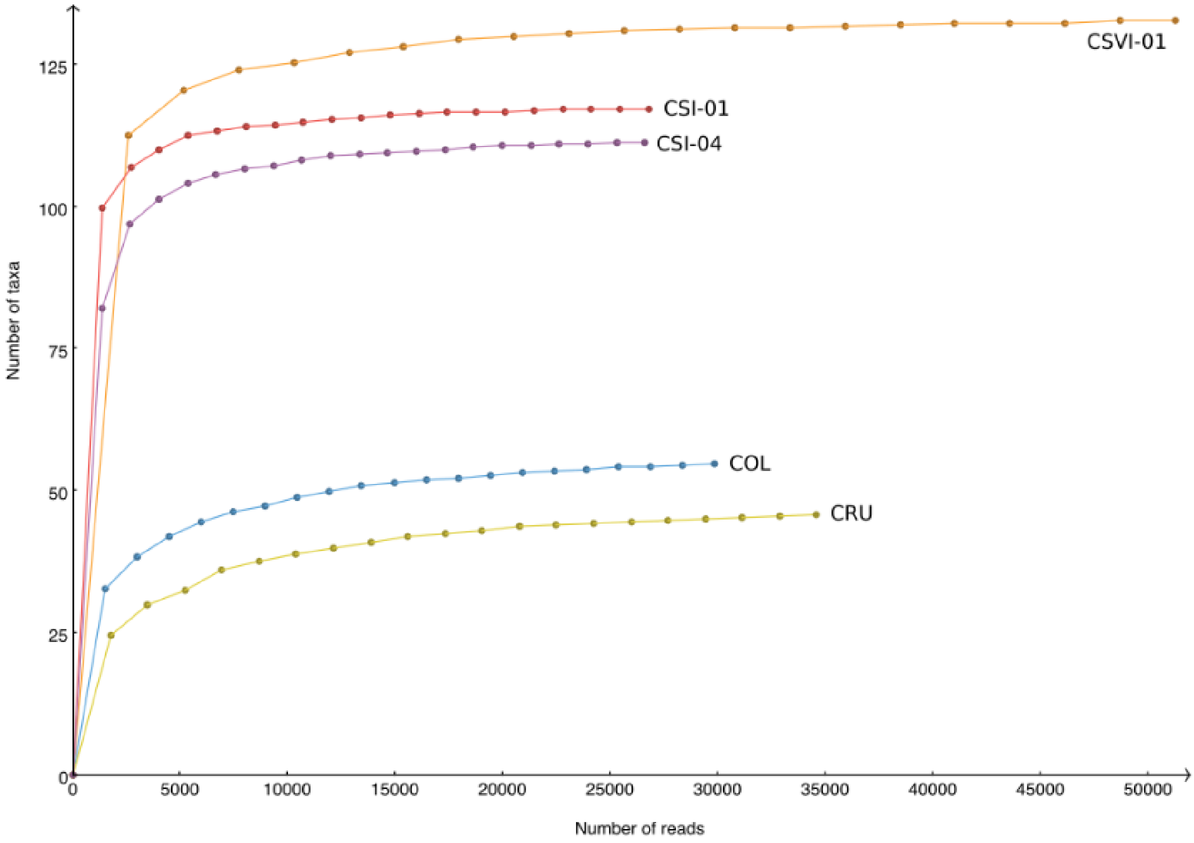

Supplementary Figure S2 | Rarefraction analysis of 16S rRNA data.

Supplementary Data S1 | Protein identification in individual searches against SWISSPROT database.

N	CSI_2	Total	%Cov	%Cov(50)	%Cov(95)	Accession	Name	Species	Peptides(95%)
1	80,71	80,71	70,81000209	68,48000288	60,55999994	sp|P04264|K2C1_HUMAN	Keratin, type II cytoskeletal 1 OS=Homo sapiens GN=KRT1 PE=1 SV	HUMAN	78
2	73,99	73,99	63,35999966	60,44999957	58,89999866	sp|P13645|K1C10_HUMAN	Keratin, type I cytoskeletal 10 OS=Homo sapiens GN=KRT10 PE=1 S	HUMAN	67
3	61,29	70,74	74,18000102	67,91999936	58,06000233	sp|P35908|K22E_HUMAN	Keratin, type II cytoskeletal 2 epidermal OS=Homo sapiens GN=KR	HUMAN	51
4	41,82	42,51	58,1099987	52,81000137	50,72000027	sp|P35527|K1C9_HUMAN	Keratin, type I cytoskeletal 9 OS=Homo sapiens GN=KRT9 PE=1 SV=	HUMAN	29
5	17,59	17,59	64,93999958	48,05000126	48,05000126	sp|P00761|TRYP_PIG	Trypsin OS=Sus scrofa PE=1 SV=1	PIG	20
9	10,67	26,62	31,56000078	26,76999867	22,86999971	sp|P48668|K2C6C_HUMAN	Keratin, type II cytoskeletal 6C OS=Homo sapiens GN=KRT6C PE=1	HUMAN	18
9	0	26,62	31,56000078	26,76999867	22,86999971	sp|P02538|K2C6A_HUMAN	Keratin, type II cytoskeletal 6A OS=Homo sapiens GN=KRT6A PE=1	HUMAN	18
16	6,11	22,99	37,63000071	26,44000053	21,69000059	sp|P13647|K2C5_HUMAN	Keratin, type II cytoskeletal 5 OS=Homo sapiens GN=KRT5 PE=1 SV	HUMAN	17
16	0	22,06	34,97000039	23,8199994	19,08999979	sp|A5A6M8|K2C5_PANTR	Keratin, type II cytoskeletal 5 OS=Pan troglodytes GN=KRT5 PE=2 S	PANTR	16
6	16,07	22,38	39,10999894	28,7499994	23,68000001	sp|P08779|K1C16_HUMAN	Keratin, type I cytoskeletal 16 OS=Homo sapiens GN=KRT16 PE=1 S	HUMAN	14
17	6,02	22,22	45,12999952	33,68999958	27,12000012	sp|P02533|K1C14_HUMAN	Keratin, type I cytoskeletal 14 OS=Homo sapiens GN=KRT14 PE=1 S	HUMAN	13
13	8,01	15,16	22,14999944	15,21999985	13,49000037	sp|Q7Z794|K2C1B_HUMAN	Keratin, type II cytoskeletal 1b OS=Homo sapiens GN=KRT77 PE=2	HUMAN	12
28	2,01	15,6	21,40000015	16,38000011	14,40999955	sp|P13646|K1C13_HUMAN	Keratin, type I cytoskeletal 13 OS=Homo sapiens GN=KRT13 PE=1 S	HUMAN	12
7	12,01	16,96	22,10000008	17,5999999	17,5999999	sp|P19013|K2C4_HUMAN	Keratin, type II cytoskeletal 4 OS=Homo sapiens GN=KRT4 PE=1 SV	HUMAN	11
8	11,33	11,33	37,72000074	37,72000074	37,72000074	sp|P06702|S10A9_HUMAN	Protein S100-A9 OS=Homo sapiens GN=S100A9 PE=1 SV=1	HUMAN	8
11	9,01	9,01	21,51000053	10,99999994	10,99999994	sp|P02768|ALBU_HUMAN	Serum albumin OS=Homo sapiens GN=ALB PE=1 SV=2	HUMAN	6
12	8,66	8,66	100	61,29000187	49,4599998	sp|P05109|S10A8_HUMAN	Protein S100-A8 OS=Homo sapiens GN=S100A8 PE=1 SV=1	HUMAN	6
29	2,01	4,61	15,37999958	5,000000075	5,000000075	sp|Q8N1N4|K2C78_HUMAN	Keratin, type II cytoskeletal 78 OS=Homo sapiens GN=KRT78 PE=2	HUMAN	6
54	0,24	10,2	25,45999885	12,72999942	8,332999796	sp|Q04695|K1C17_HUMAN	Keratin, type I cytoskeletal 17 OS=Homo sapiens GN=KRT17 PE=1 S	HUMAN	6
54	0	10,2	23,61000031	12,72999942	8,332999796	sp|A5A6M0|K1C17_PANTR	Keratin, type I cytoskeletal 17 OS=Pan troglodytes GN=KRT17 PE=2	PANTR	6
10	9,87	9,87	69,56999898	62,31999993	55,80000281	sp|P15252|REF_HEVBR	Rubber elongation factor protein OS=Hevea brasiliensis PE=1 SV=2	HEVBR	5
14	6,57	6,57	10,10000035	8,293999732	6,196000054	sp|Q02413|DSG1_HUMAN	Desmoglein-1 OS=Homo sapiens GN=DSG1 PE=1 SV=2	HUMAN	4
15	6,32	6,32	56,36000037	56,36000037	56,36000037	sp|P81605|DCD_HUMAN	Dermcidin OS=Homo sapiens GN=DCD PE=1 SV=2	HUMAN	4
18	5,62	5,62	22,82000035	17,44000018	11,54000014	sp|P29508|SPB3_HUMAN	Serpin B3 OS=Homo sapiens GN=SERPINB3 PE=1 SV=2	HUMAN	3
19	5,54	5,54	23,19999933	23,19999933	15,19999951	sp|Q91ZK5|ACTB_SIGHI	Actin, cytoplasmic 1 OS=Sigmodon hispidus GN=ACTB PE=2 SV=1	SIGHI	3
19	0	5,54	23,19999933	23,19999933	15,19999951	sp|Q8JJB8|ACTG_TRISC	Actin, cytoplasmic 2 OS=Triakis scyllium GN=actg1 PE=2 SV=1	TRISC	3
19	0	5,54	23,19999933	23,19999933	15,19999951	sp|Q76N69|ACTB_CHLAE	Actin, cytoplasmic 1 OS=Chlorocebus aethiops GN=ACTB PE=2 SV=	CHLAE	3
19	0	5,54	23,19999933	23,19999933	15,19999951	sp|Q71FK5|ACTB_CAVPO	Actin, cytoplasmic 1 OS=Cavia porcellus GN=ACTB PE=2 SV=1	CAVPO	3
19	0	5,54	23,19999933	23,19999933	15,19999951	sp|Q711N9|ACTB_MESAU	Actin, cytoplasmic 1 OS=Mesocricetus auratus GN=ACTB PE=1 SV=	MESAU	3
19	0	5,54	23,19999933	23,19999933	15,19999951	sp|Q6QAQ1|ACTB_PIG	Actin, cytoplasmic 1 OS=Sus scrofa GN=ACTB PE=1 SV=2	PIG	3
19	0	5,54	23,19999933	23,19999933	15,19999951	sp|Q6P378|ACTG_XENTR	Actin, cytoplasmic 2 OS=Xenopus tropicalis GN=actg1 PE=2 SV=1	XENTR	3
19	0	5,54	23,19999933	23,19999933	15,19999951	sp|Q5ZMQ2|ACTG_CHICK	Actin, cytoplasmic 2 OS=Gallus gallus GN=ACTG1 PE=1 SV=1	CHICK	3
19	0	5,54	23,19999933	23,19999933	15,19999951	sp|Q5R6G0|ACTB_PONAB	Actin, cytoplasmic 1 OS=Pongo abelii GN=ACTB PE=2 SV=1	PONAB	3
19	0	5,54	23,19999933	23,19999933	15,19999951	sp|Q5R1X3|ACTB_PANTR	Actin, cytoplasmic 1 OS=Pan troglodytes GN=ACTB PE=2 SV=1	PANTR	3
19	0	5,54	23,19999933	23,19999933	15,19999951	sp|Q5JAK2|ACTG_PELLE	Actin, cytoplasmic 2 OS=Pelophylax lessonae GN=actg1 PE=2 SV=1	PELLE	3
19	0	5,54	23,19999933	23,19999933	15,19999951	sp|Q4R561|ACTB_MACFA	Actin, cytoplasmic 1 OS=Macaca fascicularis GN=ACTB PE=2 SV=1	MACFA	3
19	0	5,54	23,19999933	23,19999933	15,19999951	sp|Q4L0Y2|ACTB_SPECI	Actin, cytoplasmic 1 OS=Spermophilus citellus GN=ACTB PE=2 SV=	SPECI	3
19	0	5,54	24,09999967	24,09999967	15,79000056	sp|P84856|ACTB_CHLPG	Actin, cytoplasmic 1 OS=Chlorocebus pygerythrus GN=ACTB PE=1 S	CHLPG	3
19	0	5,54	23,19999933	23,19999933	15,19999951	sp|P84336|ACTB_CAMDR	Actin, cytoplasmic 1 OS=Camelus dromedarius GN=ACTB PE=1 SV=	CAMDR	3
19	0	5,54	23,19999933	23,19999933	15,19999951	sp|P63261|ACTG_HUMAN	Actin, cytoplasmic 2 OS=Homo sapiens GN=ACTG1 PE=1 SV=1	HUMAN	3
19	0	5,54	23,19999933	23,19999933	15,19999951	sp|P63260|ACTG_MOUSE	Actin, cytoplasmic 2 OS=Mus musculus GN=Actg1 PE=1 SV=1	MOUSE	3
19	0	5,54	23,19999933	23,19999933	15,19999951	sp|P63259|ACTG_RAT	Actin, cytoplasmic 2 OS=Rattus norvegicus GN=Actg1 PE=1 SV=1	RAT	3
19	0	5,54	23,19999933	23,19999933	15,19999951	sp|P63258|ACTG_BOVIN	Actin, cytoplasmic 2 OS=Bos taurus GN=ACTG1 PE=1 SV=1	BOVIN	3
19	0	5,54	23,19999933	23,19999933	15,19999951	sp|P63257|ACTG_TRIVU	Actin, cytoplasmic 2 OS=Trichosurus vulpecula GN=ACTG1 PE=2 SV	TRIVU	3
19	0	5,54	23,19999933	23,19999933	15,19999951	sp|P63256|ACTG_ANSAN	Actin, cytoplasmic 2 OS=Anser anser anser GN=ACTG1 PE=2 SV=1	ANSAN	3
19	0	5,54	23,19999933	23,19999933	15,19999951	sp|P60713|ACTB_SHEEP	Actin, cytoplasmic 1 OS=Ovis aries GN=ACTB PE=2 SV=1	SHEEP	3
19	0	5,54	23,19999933	23,19999933	15,19999951	sp|P60712|ACTB_BOVIN	Actin, cytoplasmic 1 OS=Bos taurus GN=ACTB PE=1 SV=1	BOVIN	3
19	0	5,54	23,19999933	23,19999933	15,19999951	sp|P60711|ACTB_RAT	Actin, cytoplasmic 1 OS=Rattus norvegicus GN=Actb PE=1 SV=1	RAT	3
19	0	5,54	23,19999933	23,19999933	15,19999951	sp|P60710|ACTB_MOUSE	Actin, cytoplasmic 1 OS=Mus musculus GN=Actb PE=1 SV=1	MOUSE	3
19	0	5,54	23,19999933	23,19999933	15,19999951	sp|P60709|ACTB_HUMAN	Actin, cytoplasmic 1 OS=Homo sapiens GN=ACTB PE=1 SV=1	HUMAN	3
19	0	5,54	23,19999933	23,19999933	15,19999951	sp|P60708|ACTB_HORSE	Actin, cytoplasmic 1 OS=Equus caballus GN=ACTB PE=2 SV=1	HORSE	3
19	0	5,54	23,19999933	23,19999933	15,19999951	sp|P60707|ACTB_TRIVU	Actin, cytoplasmic 1 OS=Trichosurus vulpecula GN=ACTB PE=2 SV=	TRIVU	3
19	0	5,54	23,19999933	23,19999933	15,19999951	sp|P60706|ACTB_CHICK	Actin, cytoplasmic 1 OS=Gallus gallus GN=ACTB PE=1 SV=1	CHICK	3
19	0	5,54	23,13999981	23,13999981	15,16000032	sp|P53505|ACT5_XENLA	Actin, cytoplasmic type 5 OS=Xenopus laevis PE=3 SV=1	XENLA	3
19	0	5,54	23,13999981	23,13999981	15,16000032	sp|P53478|ACT5_CHICK	Actin, cytoplasmic type 5 OS=Gallus gallus PE=3 SV=1	CHICK	3
19	0	5,54	23,19999933	23,19999933	15,19999951	sp|P48975|ACTB_CRIGR	Actin, cytoplasmic 1 OS=Cricetulus griseus GN=ACTB PE=3 SV=1	CRIGR	3
19	0	5,54	23,13999981	23,13999981	15,16000032	sp|P15475|ACTB_XENBO	Actin, cytoplasmic 1 OS=Xenopus borealis GN=actb PE=3 SV=1	XENBO	3
19	0	5,54	23,19999933	23,19999933	15,19999951	sp|O18840|ACTB_CANLF	Actin, cytoplasmic 1 OS=Canis lupus familiaris GN=ACTB PE=2 SV=3	CANLF	3
19	0	5,54	23,19999933	23,19999933	15,19999951	sp|A2BDB0|ACTG_XENLA	Actin, cytoplasmic 2 OS=Xenopus laevis GN=actg1 PE=2 SV=1	XENLA	3
19	0	5,15	23,13999981	15,16000032	15,16000032	sp|P84185|ACT5C_ANOGA	Actin-5C OS=Anopheles gambiae GN=Act5C PE=2 SV=1	ANOGA	3
19	0	5,15	23,13999981	15,16000032	15,16000032	sp|P84184|ACT3B_HELAM	Actin-A3b, cytoplasmic OS=Helicoverpa armigera GN=actA3b PE=2	HELAM	3
19	0	5,15	23,13999981	15,16000032	15,16000032	sp|P84183|ACT4_BOMMO	Actin, cytoplasmic A4 OS=Bombyx mori GN=A4 PE=2 SV=1	BOMMO	3
19	0	5,15	23,13999981	15,16000032	15,16000032	sp|P10987|ACT1_DROME	Actin-5C OS=Drosophila melanogaster GN=Act5C PE=1 SV=4	DROME	3
19	0	5,15	15,19999951	15,19999951	15,19999951	sp|Q93129|ACTC_BRABE	Actin, cytoplasmic OS=Branchiostoma belcheri PE=2 SV=1	BRABE	3
19	0	5,15	21,43000066	21,43000066	21,43000066	sp|Q92193|ACT_CRAVI	Actin (Fragment) OS=Crassostrea virginica PE=2 SV=1	CRAVI	3
19	0	5,15	15,19999951	15,19999951	15,19999951	sp|Q6NVA9|ACTB_XENTR	Actin, cytoplasmic 1 OS=Xenopus tropicalis GN=actb PE=2 SV=1	XENTR	3
19	0	5,15	15,08000046	15,08000046	15,08000046	sp|Q25472|ACT2_MOLOC	Actin, muscle-type OS=Molgula oculata PE=3 SV=1	MOLOC	3
19	0	5,15	15,16000032	15,16000032	15,16000032	sp|Q25010|ACT3A_HELAM	Actin, cytoplasmic A3a OS=Helicoverpa armigera GN=actA3a PE=2	HELAM	3
19	0	5,15	18,44999939	18,44999939	18,44999939	sp|Q03342|ACT3_ECHGR	Actin-3 (Fragment) OS=Echinococcus granulosus GN=ACTIII PE=2 S	ECHGR	3
19	0	5,15	15,03999978	15,03999978	15,03999978	sp|Q00214|ACTM_STYPL	Actin, muscle OS=Styela plicata PE=3 SV=1	STYPL	3
19	0	5,15	15,16000032	15,16000032	15,16000032	sp|P90689|ACT_BRUMA	Actin OS=Brugia malayi PE=1 SV=1	BRUMA	3
19	0	5,15	15,16000032	15,16000032	15,16000032	sp|P68556|ACT1_DIPDE	Actin-1/4 OS=Diphyllobothrium dendriticum GN=ACT1 PE=2 SV=1	DIPDE	3
19	0	5,15	15,16000032	15,16000032	15,16000032	sp|P68555|ACT_TAESO	Actin OS=Taenia solium GN=ACT1 PE=3 SV=1	TAESO	3
19	0	5,15	15,16000032	15,16000032	15,16000032	sp|P53471|ACT2_SCHMA	Actin-2 OS=Schistosoma mansoni PE=2 SV=1	SCHMA	3
19	0	5,15	15,16000032	15,16000032	15,16000032	sp|P53456|ACT2_DIPDE	Actin-2 OS=Diphyllobothrium dendriticum GN=ACT2 PE=2 SV=1	DIPDE	3
19	0	5,15	15,16000032	15,16000032	15,16000032	sp|P04829|ACT3_BOMMO	Actin, cytoplasmic A3 OS=Bombyx mori PE=3 SV=3	BOMMO	3
19	0	5,15	15,16000032	15,16000032	15,16000032	sp|P02572|ACT2_DROME	Actin-42A OS=Drosophila melanogaster GN=Act42A PE=1 SV=3	DROME	3
19	0	5,15	15,19999951	15,19999951	15,19999951	sp|O93400|ACTB_XENLA	Actin, cytoplasmic 1 OS=Xenopus laevis GN=actb PE=2 SV=1	XENLA	3
19	0	5,15	15,16000032	15,16000032	15,16000032	sp|O18500|ACT2_SACKO	Actin-2 OS=Saccoglossus kowalevskii PE=2 SV=1	SACKO	3
19	0	5,15	15,16000032	15,16000032	15,16000032	sp|O18499|ACT1_SACKO	Actin-1 OS=Saccoglossus kowalevskii PE=2 SV=1	SACKO	3
20	4,9	4,9	14,03000057	14,03000057	12,83999979	sp|Q5RAB4|G3P_PONAB	Glyceraldehyde-3-phosphate dehydrogenase OS=Pongo abelii GN=	PONAB	3
20	0	4,9	14,03000057	14,03000057	12,83999979	sp|P04406|G3P_HUMAN	Glyceraldehyde-3-phosphate dehydrogenase OS=Homo sapiens GI	HUMAN	3
24	3,25	3,25	3,596000001	3,596000001	3,596000001	sp|Q8VNN2|BGAL_ECOLX	Beta-galactosidase OS=Escherichia coli GN=lacZ PE=3 SV=1	ECOLX	3
24	0	3,25	3,612999991	3,612999991	3,612999991	sp|Q3Z583|BGAL_SHISS	Beta-galactosidase OS=Shigella sonnei (strain Ss046) GN=lacZ PE=3	SHISS	3
24	0	3,25	3,612999991	3,612999991	3,612999991	sp|P00722|BGAL_ECOLI	Beta-galactosidase OS=Escherichia coli (strain K12) GN=lacZ PE=1 S	ECOLI	3
24	0	3,25	3,612999991	3,612999991	3,612999991	sp|B1J0T5|BGAL_ECOLC	Beta-galactosidase OS=Escherichia coli (strain ATCC 8739 / DSM 15	ECOLC	3
24	0	3,25	3,612999991	3,612999991	3,612999991	sp|A7ZWZ1|BGAL_ECOHS	Beta-galactosidase OS=Escherichia coli O9:H4 (strain HS) GN=lacZ I	ECOHS	3
24	0	3,25	3,612999991	3,612999991	3,612999991	sp|A7ZI91|BGAL_ECO24	Beta-galactosidase OS=Escherichia coli O139:H28 (strain E24377A ,	ECO24	3
32	2	3,04	4,887999967	4,887999967	4,887999967	sp|Q6IFX1|K1C24_RAT	Keratin, type I cytoskeletal 24 OS=Rattus norvegicus GN=Krt24 PE=	RAT	3
32	0	3,04	4,687999934	4,687999934	4,687999934	sp|A1L317|K1C24_MOUSE	Keratin, type I cytoskeletal 24 OS=Mus musculus GN=Krt24 PE=2 S	MOUSE	3
19	0	4,35	18,40000021	18,40000021	10,40000021	sp|P29751|ACTB_RABIT	Actin, cytoplasmic 1 OS=Oryctolagus cuniculus GN=ACTB PE=2 SV=	RABIT	2
19	0	4	3,627999872	3,627999872	3,627999872	sp|Q6S8J3|POTEE_HUMAN	POTE ankyrin domain family member E OS=Homo sapiens GN=POT	HUMAN	2
19	0	4	10,36999971	10,36999971	10,36999971	sp|P17304|ACTM_APLCA	Actin, muscle OS=Aplysia californica PE=2 SV=1	APLCA	2
19	0	3,4	17,06999987	17,06999987	9,066999704	sp|Q7ZVI7|ACTB1_DANRE	Actin, cytoplasmic 1 OS=Danio rerio GN=actba PE=2 SV=2	DANRE	2

19	0	3,4	17,06999987	17,06999987	9,066999704	sp|Q7ZVF9|ACTB2_DANRE	Actin, cytoplasmic 2 OS=Danio rerio GN=actbb PE=2 SV=2	DANRE	2
19	0	3,4	17,06999987	17,06999987	9,066999704	sp|P83751|ACTB_CTEID	Actin, cytoplasmic 1 OS=Ctenopharyngodon idella GN=actb PE=3 S\	CTEID	2
19	0	3,4	17,06999987	17,06999987	9,066999704	sp|P83750|ACTB_CYPCA	Actin, cytoplasmic 1 OS=Cyprinus carpio GN=actb PE=3 SV=1	CYPCA	2
19	0	3,4	17,02000052	17,02000052	9,042999893	sp|P53506|ACT8_XENLA	Actin, cytoplasmic type 8 OS=Xenopus laevis PE=3 SV=1	XENLA	2
19	0	3,4	17,06999987	17,06999987	9,066999704	sp|P53485|ACTB2_TAKRU	Actin, cytoplasmic 2 OS=Takifugu rubripes GN=actbb PE=3 SV=1	TAKRU	2
21	4,2	4,2	23,82999957	16,43999964	11,06999964	sp|P25311|ZA2G_HUMAN	Zinc-alpha-2-glycoprotein OS=Homo sapiens GN=AZGP1 PE=1 SV=2	HUMAN	2
22	3,61	3,61	13,51000071	8,23699981	5,601000041	sp|P02769|ALBU_BOVIN	Serum albumin OS=Bos taurus GN=ALB PE=1 SV=4	BOVIN	2
23	3,56	3,56	51,09000206	51,09000206	21,73999995	sp|P80511|S10AC_HUMAN	Protein S100-A12 OS=Homo sapiens GN=S100A12 PE=1 SV=2	HUMAN	2
24	0	2	2,147999965	2,147999965	2,147999965	sp|Q8X685|BGAL_ECO57	Beta-galactosidase OS=Escherichia coli O157:H7 GN=lacZ PE=3 SV=	ECO57	2
24	0	2	2,147999965	2,147999965	2,147999965	sp|Q8FKG6|BGAL_ECOL6	Beta-galactosidase OS=Escherichia coli O6:H1 (strain CFT073 / ATC	ECOL6	2
24	0	2	2,147999965	2,147999965	2,147999965	sp|Q32JB6|BGAL_SHIDS	Beta-galactosidase OS=Shigella dysenteriae serotype 1 (strain Sd1!	SHIDS	2
24	0	2	2,147999965	2,147999965	2,147999965	sp|Q1RFJ2|BGAL_ECOUT	Beta-galactosidase OS=Escherichia coli (strain UTI89 / UPEC) GN=la	ECOUT	2
24	0	2	2,147999965	2,147999965	2,147999965	sp|Q0TKT1|BGAL_ECOL5	Beta-galactosidase OS=Escherichia coli O6:K15:H31 (strain 536 / UI	ECOL5	2
24	0	2	2,147999965	2,147999965	2,147999965	sp|B7UJI9|BGAL_ECO27	Beta-galactosidase OS=Escherichia coli O127:H6 (strain E2348/69 /	ECO27	2
24	0	2	2,147999965	2,147999965	2,147999965	sp|B7N8Q1|BGAL_ECOLU	Beta-galactosidase OS=Escherichia coli O17:K52:H18 (strain UMN0	ECOLU	2
24	0	2	2,147999965	2,147999965	2,147999965	sp|B5Z2P7|BGAL_ECO5E	Beta-galactosidase OS=Escherichia coli O157:H7 (strain EC4115 / E	ECO5E	2
24	0	2	2,147999965	2,147999965	2,147999965	sp|B1LIM9|BGAL_ECOSM	Beta-galactosidase OS=Escherichia coli (strain SMS-3-5 / SECEC) GI	ECOSM	2
24	0	2	2,147999965	2,147999965	2,147999965	sp|A7KGA5|BGAL2_KLEPN	Beta-galactosidase OS=Klebsiella pneumoniae GN=lacZ PE=3 SV=1	KLEPN	2
24	0	2	2,147999965	2,147999965	2,147999965	sp|A6TI29|BGAL2_KLEP7	Beta-galactosidase 2 OS=Klebsiella pneumoniae subsp. pneumonia	KLEP7	2
24	0	2	2,147999965	2,147999965	2,147999965	sp|A1A831|BGAL_ECOK1	Beta-galactosidase OS=Escherichia coli O1:K1 / APEC GN=lacZ PE=3	ECOK1	2
25	2,86	2,86	65,82000256	65,82000256	65,82000256	sp|P02814|SMR3B_HUMAN	Submaxillary gland androgen-regulated protein 3B OS=Homo sapie	HUMAN	2
22	0	1,24	6,095999852	6,095999852	3,460000083	sp|P14639|ALBU_SHEEP	Serum albumin OS=Ovis aries GN=ALB PE=1 SV=1	SHEEP	1
26	2,11	2,11	3,892999887	3,892999887	2,415999956	sp|Q8WNW3|PLAK_PIG	Junction plakoglobin OS=Sus scrofa GN=Jup PE=2 SV=1	PIG	1
26	0	2,11	3,892999887	3,892999887	2,415999956	sp|Q8SPJ1|PLAK_BOVIN	Junction plakoglobin OS=Bos taurus GN=JUP PE=2 SV=1	BOVIN	1
26	0	2,11	3,892999887	3,892999887	2,415999956	sp|Q6P0K8|PLAK_RAT	Junction plakoglobin OS=Rattus norvegicus GN=Jup PE=1 SV=1	RAT	1
26	0	2,11	3,892999887	3,892999887	2,415999956	sp|Q02257|PLAK_MOUSE	Junction plakoglobin OS=Mus musculus GN=Jup PE=1 SV=3	MOUSE	1
26	0	2,11	3,892999887	3,892999887	2,415999956	sp|P14923|PLAK_HUMAN	Junction plakoglobin OS=Homo sapiens GN=JUP PE=1 SV=3	HUMAN	1
27	2,08	2,08	34,90999937	34,90999937	26,4200002	sp|Q5R9M3|THIO_PONAB	Thioredoxin OS=Pongo abelii GN=TXN PE=3 SV=3	PONAB	1
27	0	2,08	35,24000049	35,24000049	26,66999996	sp|P10599|THIO_HUMAN	Thioredoxin OS=Homo sapiens GN=TXN PE=1 SV=3	HUMAN	1
30	2,01	2,01	24,75000024	10,89000031	10,89000031	sp|P31151|S10A7_HUMAN	Protein S100-A7 OS=Homo sapiens GN=S100A7 PE=1 SV=4	HUMAN	1
31	2,01	2,01	27,54999995	11,21999994	11,21999994	sp|Q6B345|S10AB_RAT	Protein S100-A11 OS=Rattus norvegicus GN=S100a11 PE=3 SV=1	RAT	1
31	0	2,01	27,54999995	11,21999994	11,21999994	sp|P50543|S10AB_MOUSE	Protein S100-A11 OS=Mus musculus GN=S100a11 PE=1 SV=1	MOUSE	1
31	0	2,01	25,70999861	10,48000008	10,48000008	sp|P31949|S10AB_HUMAN	Protein S100-A11 OS=Homo sapiens GN=S100A11 PE=1 SV=2	HUMAN	1
31	0	2,01	26,46999955	10,77999994	10,77999994	sp|P24480|S10AB_RABIT	Protein S100-A11 OS=Oryctolagus cuniculus GN=S100A11 PE=1 SV	RABIT	1
33	2	2	18,3799997	10,58000028	10,58000028	sp|P12763|FETUA_BOVIN	Alpha-2-HS-glycoprotein OS=Bos taurus GN=AHSG PE=1 SV=2	BOVIN	1
34	2	2	11,80000007	4,129999876	4,129999876	sp|Q6TEQ7|ANXA2_CANLF	Annexin A2 OS=Canis lupus familiaris GN=ANXA2 PE=1 SV=1	CANLF	1
34	0	2	11,80000007	4,129999876	4,129999876	sp|Q5R5A0|ANXA2_PONAB	Annexin A2 OS=Pongo abelii GN=ANXA2 PE=2 SV=1	PONAB	1
34	0	2	11,80000007	4,129999876	4,129999876	sp|Q2Q1M6|ANXA2_CEREL	Annexin A2 OS=Cervus elaphus GN=ANXA2 PE=2 SV=1	CEREL	1
34	0	2	11,80000007	4,129999876	4,129999876	sp|Q07936|ANXA2_RAT	Annexin A2 OS=Rattus norvegicus GN=Anxa2 PE=1 SV=2	RAT	1
34	0	2	11,80000007	4,129999876	4,129999876	sp|P19620|ANXA2_PIG	Annexin A2 OS=Sus scrofa GN=ANXA2 PE=1 SV=4	PIG	1
34	0	2	11,80000007	4,129999876	4,129999876	sp|P07355|ANXA2_HUMAN	Annexin A2 OS=Homo sapiens GN=ANXA2 PE=1 SV=2	HUMAN	1
34	0	2	11,80000007	4,129999876	4,129999876	sp|P04272|ANXA2_BOVIN	Annexin A2 OS=Bos taurus GN=ANXA2 PE=1 SV=2	BOVIN	1
34	0	2	11,80000007	4,129999876	4,129999876	sp|A2SW69|ANXA2_SHEEP	Annexin A2 OS=Ovis aries GN=ANXA2 PE=1 SV=1	SHEEP	1
34	0	2	8,850000054	4,129999876	4,129999876	sp|P07356|ANXA2_MOUSE	Annexin A2 OS=Mus musculus GN=Anxa2 PE=1 SV=2	MOUSE	1
34	0	2	15,08000046	7,034999877	7,034999877	sp|C0HJG9|ANXA2_MESAU	Annexin A2 (Fragments) OS=Mesocricetus auratus PE=1 SV=1	MESAU	1
35	2	2	15,13999999	4,086999968	4,086999968	sp|P34955|A1AT_BOVIN	Alpha-1-antiproteinase OS=Bos taurus GN=SERPINA1 PE=1 SV=1	BOVIN	1
36	2	2	8,677999675	3,719000146	3,719000146	sp|P31944|CASPE_HUMAN	Caspase-14 OS=Homo sapiens GN=CASP14 PE=1 SV=2	HUMAN	1
37	2	2	0,961900037	0,460099988	0,460099988	sp|Q5D862|FILA2_HUMAN	Filaggrin-2 OS=Homo sapiens GN=FLG2 PE=1 SV=1	HUMAN	1
38	2	2	6,831999868	3,415999934	3,415999934	sp|P05089|ARGI1_HUMAN	Arginase-1 OS=Homo sapiens GN=ARG1 PE=1 SV=2	HUMAN	1
39	2	2	1,345000044	1,345000044	1,345000044	sp|Q9Z1P2|ACTN1_RAT	Alpha-actinin-1 OS=Rattus norvegicus GN=Actn1 PE=1 SV=1	RAT	1
39	0	2	1,317000017	1,317000017	1,317000017	sp|Q9QXQ0|ACTN4_RAT	Alpha-actinin-4 OS=Rattus norvegicus GN=Actn4 PE=1 SV=2	RAT	1
39	0	2	1,341999974	1,341999974	1,341999974	sp|Q9JI91|ACTN2_MOUSE	Alpha-actinin-2 OS=Mus musculus GN=Actn2 PE=1 SV=2	MOUSE	1
39	0	2	1,327	1,327	1,327	sp|Q90734|ACTN4_CHICK	Alpha-actinin-4 OS=Gallus gallus GN=ACTN4 PE=1 SV=1	CHICK	1
39	0	2	1,345000044	1,345000044	1,345000044	sp|Q7TPR4|ACTN1_MOUSE	Alpha-actinin-1 OS=Mus musculus GN=Actn1 PE=1 SV=1	MOUSE	1
39	0	2	1,317000017	1,317000017	1,317000017	sp|Q5RCS6|ACTN4_PONAB	Alpha-actinin-4 OS=Pongo abelii GN=ACTN4 PE=2 SV=1	PONAB	1
39	0	2	1,341999974	1,341999974	1,341999974	sp|Q3ZC55|ACTN2_BOVIN	Alpha-actinin-2 OS=Bos taurus GN=ACTN2 PE=2 SV=1	BOVIN	1
39	0	2	1,345000044	1,345000044	1,345000044	sp|Q3B7N2|ACTN1_BOVIN	Alpha-actinin-1 OS=Bos taurus GN=ACTN1 PE=2 SV=1	BOVIN	1
39	0	2	1,345000044	1,345000044	1,345000044	sp|Q2PFV7|ACTN1_MACFA	Alpha-actinin-1 OS=Macaca fascicularis GN=ACTN1 PE=2 SV=1	MACFA	1
39	0	2	1,331999991	1,331999991	1,331999991	sp|Q0III9|ACTN3_BOVIN	Alpha-actinin-3 OS=Bos taurus GN=ACTN3 PE=2 SV=1	BOVIN	1
39	0	2	1,331999991	1,331999991	1,331999991	sp|Q08043|ACTN3_HUMAN	Alpha-actinin-3 OS=Homo sapiens GN=ACTN3 PE=1 SV=2	HUMAN	1
39	0	2	1,315999962	1,315999962	1,315999962	sp|P57780|ACTN4_MOUSE	Alpha-actinin-4 OS=Mus musculus GN=Actn4 PE=1 SV=1	MOUSE	1
39	0	2	1,341999974	1,341999974	1,341999974	sp|P35609|ACTN2_HUMAN	Alpha-actinin-2 OS=Homo sapiens GN=ACTN2 PE=1 SV=1	HUMAN	1
39	0	2	1,338000037	1,338000037	1,338000037	sp|P20111|ACTN2_CHICK	Alpha-actinin-2 OS=Gallus gallus GN=ACTN2 PE=2 SV=1	CHICK	1
39	0	2	1,345000044	1,345000044	1,345000044	sp|P12814|ACTN1_HUMAN	Alpha-actinin-1 OS=Homo sapiens GN=ACTN1 PE=1 SV=2	HUMAN	1
39	0	2	1,343999989	1,343999989	1,343999989	sp|P05094|ACTN1_CHICK	Alpha-actinin-1 OS=Gallus gallus GN=ACTN1 PE=1 SV=3	CHICK	1
39	0	2	1,333000045	1,333000045	1,333000045	sp|O88990|ACTN3_MOUSE	Alpha-actinin-3 OS=Mus musculus GN=Actn3 PE=2 SV=1	MOUSE	1
39	0	2	1,317000017	1,317000017	1,317000017	sp|O43707|ACTN4_HUMAN	Alpha-actinin-4 OS=Homo sapiens GN=ACTN4 PE=1 SV=2	HUMAN	1
39	0	2	1,317000017	1,317000017	1,317000017	sp|A5D7D1|ACTN4_BOVIN	Alpha-actinin-4 OS=Bos taurus GN=ACTN4 PE=2 SV=1	BOVIN	1
40	2	2	1,456999965	1,456999965	1,456999965	sp|Q7SIH1|A2MG_BOVIN	Alpha-2-macroglobulin OS=Bos taurus GN=A2M PE=1 SV=2	BOVIN	1
41	2	2	2,051999979	2,051999979	2,051999979	sp|Q3SX14|GELS_BOVIN	Gelsolin OS=Bos taurus GN=GSN PE=2 SV=1	BOVIN	1
41	0	2	2,051999979	2,051999979	2,051999979	sp|Q28372|GELS_HORSE	Gelsolin OS=Equus caballus GN=GSN PE=1 SV=2	HORSE	1
41	0	2	1,943000033	1,943000033	1,943000033	sp|P20305|GELS_PIG	Gelsolin (Fragment) OS=Sus scrofa GN=GSN PE=1 SV=1	PIG	1
41	0	2	1,917999983	1,917999983	1,917999983	sp|P06396|GELS_HUMAN	Gelsolin OS=Homo sapiens GN=GSN PE=1 SV=1	HUMAN	1
42	2	2	1,913999952	1,913999952	1,913999952	sp|P22758|TGM1_RABIT	Protein-glutamine gamma-glutamyltransferase K OS=Oryctolagus c	RABIT	1
42	0	2	1,957999915	1,957999915	1,957999915	sp|P22735|TGM1_HUMAN	Protein-glutamine gamma-glutamyltransferase K OS=Homo sapien	HUMAN	1
43	2	2	9,544999897	9,544999897	9,544999897	sp|O75629|CREG1_HUMAN	Protein CREG1 OS=Homo sapiens GN=CREG1 PE=1 SV=1	HUMAN	1
44	1,68	1,68	32,78999925	20,9800005	20,9800005	sp|Q8MKD1|UBB_HORSE	Polyubiquitin-B OS=Equus caballus GN=UBB PE=2 SV=3	HORSE	1
44	0	1,68	32,89000094	21,05000019	21,05000019	sp|Q865C5|UBIQ_CAMDR	Ubiquitin OS=Camelus dromedarius PE=3 SV=2	CAMDR	1
44	0	1,68	30,86000085	19,75000054	19,75000054	sp|Q63429|UBC_RAT	Polyubiquitin-C OS=Rattus norvegicus GN=Ubc PE=1 SV=1	RAT	1
44	0	1,68	39,68000114	25,40000081	25,40000081	sp|P84589|UBIQ_LUMTE	Ubiquitin (Fragment) OS=Lumbricus terrestris PE=1 SV=2	LUMTE	1
44	0	1,68	16,03000015	10,26000008	10,26000008	sp|P79781|RS27A_CHICK	Ubiquitin-40S ribosomal protein S27a OS=Gallus gallus GN=RPS27A	CHICK	1
44	0	1,68	19,52999979	12,5	12,5	sp|P68205|RL40_OPHHA	Ubiquitin-60S ribosomal protein L40 OS=Ophiophagus hannah PE=	OPHHA	1
44	0	1,68	16,03000015	10,26000008	10,26000008	sp|P68203|RS27A_SPOFR	Ubiquitin-40S ribosomal protein S27a OS=Spodoptera frugiperda P	SPOFR	1
44	0	1,68	16,13000035	10,32000035	10,32000035	sp|P68202|RS27A_PLUXY	Ubiquitin-40S ribosomal protein S27a OS=Plutella xylostella PE=2 S	PLUXY	1
44	0	1,68	16,03000015	10,26000008	10,26000008	sp|P68200|RS27A_ICTPU	Ubiquitin-40S ribosomal protein S27a OS=Ictalurus punctatus GN=	ICTPU	1
44	0	1,68	32,89000094	21,05000019	21,05000019	sp|P68197|UBIQ_CERCA	Ubiquitin OS=Ceratitis capitata PE=1 SV=1	CERCA	1
44	0	1,68	19,52999979	12,5	12,5	sp|P63053|RL40_PIG	Ubiquitin-60S ribosomal protein L40 OS=Sus scrofa GN=UBA52 PE=	PIG	1
44	0	1,68	19,52999979	12,5	12,5	sp|P63052|RL40_FELCA	Ubiquitin-60S ribosomal protein L40 OS=Felis catus GN=UBA52 PE=	FELCA	1
44	0	1,68	19,52999979	12,5	12,5	sp|P63050|RL40_CANLF	Ubiquitin-60S ribosomal protein L40 OS=Canis lupus familiaris GN=	CANLF	1
44	0	1,68	19,52999979	12,5	12,5	sp|P63048|RL40_BOVIN	Ubiquitin-60S ribosomal protein L40 OS=Bos taurus GN=UBA52 PE=	BOVIN	1
44	0	1,68	16,03000015	10,26000008	10,26000008	sp|P62992|RS27A_BOVIN	Ubiquitin-40S ribosomal protein S27a OS=Bos taurus GN=RPS27A F	BOVIN	1
44	0	1,68	19,52999979	12,5	12,5	sp|P62987|RL40_HUMAN	Ubiquitin-60S ribosomal protein L40 OS=Homo sapiens GN=UBA52	HUMAN	1
44	0	1,68	19,52999979	12,5	12,5	sp|P62986|RL40_RAT	Ubiquitin-60S ribosomal protein L40 OS=Rattus norvegicus GN=Ub	RAT	1
44	0	1,68	19,52999979	12,5	12,5	sp|P62984|RL40_MOUSE	Ubiquitin-60S ribosomal protein L40 OS=Mus musculus GN=Uba52	MOUSE	1
44	0	1,68	16,03000015	10,26000008	10,26000008	sp|P62983|RS27A_MOUSE	Ubiquitin-40S ribosomal protein S27a OS=Mus musculus GN=Rps2	MOUSE	1

44	0	1,68	16,03000015	10,26000008	10,26000008	sp|P62982|RS27A_RAT	Ubiquitin-40S ribosomal protein S27a OS=Rattus norvegicus GN=R	RAT	1
44	0	1,68	16,03000015	10,26000008	10,26000008	sp|P62979|RS27A_HUMAN	Ubiquitin-40S ribosomal protein S27a OS=Homo sapiens GN=RPS2	HUMAN	1
44	0	1,68	16,03000015	10,26000008	10,26000008	sp|P62978|RS27A_CAVPO	Ubiquitin-40S ribosomal protein S27a OS=Cavia porcellus GN=RPS2	CAVPO	1
44	0	1,68	30,39999902	19,44999993	19,44999993	sp|P62976|UBIQP_CRIGR	Polyubiquitin OS=Cricetulus griseus PE=2 SV=2	CRIGR	1
44	0	1,68	32,89000094	21,05000019	21,05000019	sp|P62975|UBIQ_RABIT	Ubiquitin OS=Oryctolagus cuniculus PE=1 SV=1	RABIT	1
44	0	1,68	35,33000052	19,15999949	19,15999949	sp|P62972|UBIQP_XENLA	Polyubiquitin (Fragment) OS=Xenopus laevis PE=1 SV=2	XENLA	1
44	0	1,68	16,13000035	10,32000035	10,32000035	sp|P29504|RS27A_MANSE	Ubiquitin-40S ribosomal protein S27a OS=Manduca sexta PE=2 SV=	MANSE	1
44	0	1,68	19,52999979	12,5	12,5	sp|P18101|RL40_DROME	Ubiquitin-60S ribosomal protein L40 OS=Drosophila melanogaster	DROME	1
44	0	1,68	16,03000015	10,26000008	10,26000008	sp|P15357|RS27A_DROME	Ubiquitin-40S ribosomal protein S27a OS=Drosophila melanogaste	DROME	1
44	0	1,68	32,60999918	20,87000012	20,87000012	sp|P0CH28|UBC_BOVIN	Polyubiquitin-C OS=Bos taurus GN=UBC PE=1 SV=1	BOVIN	1
44	0	1,68	32,76999891	20,97000033	20,97000033	sp|P0CG69|UBIQP_DROME	Polyubiquitin OS=Drosophila melanogaster GN=Ubi-p63E PE=3 SV=	DROME	1
44	0	1,68	32,82999992	21,00999951	21,00999951	sp|P0CG68|UBC_PIG	Polyubiquitin-C OS=Sus scrofa GN=UBC PE=2 SV=1	PIG	1
44	0	1,68	32,74999857	20,96000016	20,96000016	sp|P0CG67|UBB_GORGO	Polyubiquitin-B OS=Gorilla gorilla gorilla GN=UBB PE=3 SV=1	GORGO	1
44	0	1,68	32,8399986	21,01999968	21,01999968	sp|P0CG66|UBC_GORGO	Polyubiquitin-C OS=Gorilla gorilla gorilla GN=UBC PE=3 SV=1	GORGO	1
44	0	1,68	32,74999857	20,96000016	20,96000016	sp|P0CG65|UBB_PANTR	Polyubiquitin-B OS=Pan troglodytes GN=UBB PE=3 SV=1	PANTR	1
44	0	1,68	32,85000026	21,01999968	21,01999968	sp|P0CG64|UBC_PANTR	Polyubiquitin-C OS=Pan troglodytes GN=UBC PE=3 SV=1	PANTR	1
44	0	1,68	32,78999925	20,9800005	20,9800005	sp|P0CG62|UBB_CHICK	Polyubiquitin-B OS=Gallus gallus GN=UBB PE=2 SV=1	CHICK	1
44	0	1,68	32,85000026	21,01999968	21,01999968	sp|P0CG61|UBC_PONPY	Polyubiquitin-C OS=Pongo pygmaeus GN=UBC PE=3 SV=1	PONPY	1
44	0	1,68	32,74999857	20,96000016	20,96000016	sp|P0CG60|UBB_PONPY	Polyubiquitin-B OS=Pongo pygmaeus GN=UBB PE=3 SV=1	PONPY	1
44	0	1,68	32,78999925	20,9800005	20,9800005	sp|P0CG55|UBB_SHEEP	Polyubiquitin-B OS=Ovis aries GN=UBB PE=2 SV=1	SHEEP	1
44	0	1,68	32,15000033	20,57999969	20,57999969	sp|P0CG54|UBB_CAVPO	Polyubiquitin-B OS=Cavia porcellus GN=UBB PE=2 SV=1	CAVPO	1
44	0	1,68	32,78999925	20,9800005	20,9800005	sp|P0CG53|UBB_BOVIN	Polyubiquitin-B OS=Bos taurus GN=UBB PE=1 SV=1	BOVIN	1
44	0	1,68	32,78999925	20,9800005	20,9800005	sp|P0CG51|UBB_RAT	Polyubiquitin-B OS=Rattus norvegicus GN=Ubb PE=1 SV=1	RAT	1
44	0	1,68	30,64999878	19,61999983	19,61999983	sp|P0CG50|UBC_MOUSE	Polyubiquitin-C OS=Mus musculus GN=Ubc PE=1 SV=2	MOUSE	1
44	0	1,68	32,78999925	20,9800005	20,9800005	sp|P0CG49|UBB_MOUSE	Polyubiquitin-B OS=Mus musculus GN=Ubb PE=2 SV=1	MOUSE	1
44	0	1,68	32,85000026	21,01999968	21,01999968	sp|P0CG48|UBC_HUMAN	Polyubiquitin-C OS=Homo sapiens GN=UBC PE=1 SV=3	HUMAN	1
44	0	1,68	32,74999857	20,96000016	20,96000016	sp|P0CG47|UBB_HUMAN	Polyubiquitin-B OS=Homo sapiens GN=UBB PE=1 SV=1	HUMAN	1
44	0	1,68	19,52999979	12,5	12,5	sp|P0C276|RL40_SHEEP	Ubiquitin-60S ribosomal protein L40 OS=Ovis aries GN=UBA52 PE=	SHEEP	1
44	0	1,68	19,52999979	12,5	12,5	sp|P0C275|RL40_PONPY	Ubiquitin-60S ribosomal protein L40 OS=Pongo pygmaeus GN=UB	PONPY	1
44	0	1,68	19,52999979	12,5	12,5	sp|P0C273|RL40_MACFA	Ubiquitin-60S ribosomal protein L40 OS=Macaca fascicularis GN=U	MACFA	1
45	1,5	1,5	7,767000049	3,155000135	3,155000135	sp|P07339|CATD_HUMAN	Cathepsin D OS=Homo sapiens GN=CTSD PE=1 SV=1	HUMAN	1
46	1,04	1,04	3,403000161	3,403000161	3,403000161	sp|Q7YR44|CDSN_PANTR	Corneodesmosin OS=Pan troglodytes GN=CDSN PE=2 SV=1	PANTR	1
46	0	1,04	3,370999917	3,370999917	3,370999917	sp|Q5TM45|CDSN_MACMU	Corneodesmosin OS=Macaca mulatta GN=CDSN PE=3 SV=1	MACMU	1
46	0	1,04	3,403000161	3,403000161	3,403000161	sp|Q15517|CDSN_HUMAN	Corneodesmosin OS=Homo sapiens GN=CDSN PE=1 SV=3	HUMAN	1
47	0,85	0,85	4,02700007	2,907999977	1,678000018	sp|Q08554|DSC1_HUMAN	Desmocollin-1 OS=Homo sapiens GN=DSC1 PE=1 SV=2	HUMAN	1
48	0,72	0,72	3,895999864	2,597000077	2,597000077	sp|Q08188|TGM3_HUMAN	Protein-glutamine gamma-glutamyltransferase E OS=Homo sapien	HUMAN	1
49	0,65	0,65	22,95999974	22,22000062	6,667000055	sp|Q01469|FABP5_HUMAN	Fatty acid-binding protein, epidermal OS=Homo sapiens GN=FABP	HUMAN	1
50	0,58	0,58	24,66000021	8,218999952	8,218999952	sp|P12273|PIP_HUMAN	Prolactin-inducible protein OS=Homo sapiens GN=PIP PE=1 SV=1	HUMAN	1
50	0	0,55	8,218999952	8,218999952	8,218999952	sp|A0A890|PIP_HYLAG	Prolactin-inducible protein homolog OS=Hylobates agilis GN=PIP P	HYLAG	1
50	0	0,55	8,218999952	8,218999952	8,218999952	sp|A0A888|PIP_PONPY	Prolactin-inducible protein homolog OS=Pongo pygmaeus GN=PIP	PONPY	1
51	0,41	0,41	1,950999908	0,626999978	0,626999978	sp|P15924|DESP_HUMAN	Desmoplakin OS=Homo sapiens GN=DSP PE=1 SV=3	HUMAN	1
51	0	0,41	1,248999964	0,624299981	0,624299981	sp|E9Q557|DESP_MOUSE	Desmoplakin OS=Mus musculus GN=Dsp PE=1 SV=1	MOUSE	1
52	0,36	0,36	7,512000203	7,512000203	7,512000203	RRRRRsp|P13673|SKGR_XENLA	REVERSED Skin granule protein OS=Xenopus laevis GN=sgp PE=2 S	XENLA	1
53	0,3	0,3	7,534000278	7,534000278	7,534000278	sp|Q9NZT1|CALL5_HUMAN	Calmodulin-like protein 5 OS=Homo sapiens GN=CALML5 PE=1 SV=	HUMAN	1
55	0,21	0,21	1,917999983	1,917999983	0	RRRRRsp|Q9SFC7|FB135_ARATH	REVERSED F-box protein At3g07870 OS=Arabidopsis thaliana GN=A	ARATH	0
56	0,18	0,18	1,28899999	1,28899999	0	sp|Q92E69|SECA2_LISIN	Protein translocase subunit SecA 2 OS=Listeria innocua serovar 6a	LISIN	0
56	0	0,18	1,28899999	1,28899999	0	sp|Q722W7|SECA2_LISMF	Protein translocase subunit SecA 2 OS=Listeria monocytogenes ser	LISMF	0
56	0	0,18	1,28899999	1,28899999	0	sp|P0DJP3|SECA2_LISMO	Protein translocase subunit SecA 2 OS=Listeria monocytogenes ser	LISMO	0
56	0	0,18	1,28899999	1,28899999	0	sp|G2K3V6|SECA2_LISM4	Protein translocase subunit SecA 2 OS=Listeria monocytogenes ser	LISM4	0
57	0,15	0,15	2,228000015	2,228000015	0	sp|Q5NXZ4|GLGC_AROAE	Glucose-1-phosphate adenylyltransferase OS=Aromatoleum aroma	AROAE	0
58	0,09	0,09	2,762999944	1,727000065	0	sp|Q5T749|KPRP_HUMAN	Keratinocyte proline-rich protein OS=Homo sapiens GN=KPRP PE=	HUMAN	0
58	0	0,09	1,431000046	1,431000046	0	sp|Q7TQM5|KPRP_RAT	Keratinocyte proline-rich protein OS=Rattus norvegicus GN=Kprp P	RAT	0
59	0,06	0,06	3,406000137	3,406000137	0	sp|Q6LY02|MCH_METMP	Methenyltetrahydromethanopterin cyclohydrolase OS=Methanoco	METMP	0
59	0	0,06	3,406000137	3,406000137	0	sp|A6VGD1|MCH_METM7	Methenyltetrahydromethanopterin cyclohydrolase OS=Methanoco	METM7	0
59	0	0,06	3,406000137	3,406000137	0	sp|A6UPJ2|MCH_METVS	Methenyltetrahydromethanopterin cyclohydrolase OS=Methanoco	METVS	0
59	0	0,06	3,406000137	3,406000137	0	sp|A4FWY5|MCH_METM5	Methenyltetrahydromethanopterin cyclohydrolase OS=Methanoco	METM5	0

N	CSI_3	Total	%Cov	%Cov(50)	%Cov(95)	Accession	Name	Species	Peptides(95%)
1	115,6	115,6	69,0999985	66,6100025	65,990001	sp|P04264|K2C1_HUMAN	Keratin, type II cytoskeletal 1 OS=Homo sapier	HUMAN	113
2	88,01	88,01	60,2699995	58,5600019	57,0200026	sp|P13645|K1C10_HUMAN	Keratin, type I cytoskeletal 10 OS=Homo sapie	HUMAN	89
3	74,33	74,37	77,6899993	77,6899993	77,6899993	sp|P35527|K1C9_HUMAN	Keratin, type I cytoskeletal 9 OS=Homo sapien	HUMAN	66
4	67,97	80,24	76,9999981	71,9900012	68,8600004	sp|P35908|K22E_HUMAN	Keratin, type II cytoskeletal 2 epidermal OS=H	HUMAN	51
5	38,34	38,34	65,7999992	59,740001	59,740001	sp|P00761|TRYP_PIG	Trypsin OS=Sus scrofa PE=1 SV=1	PIG	36
7	24,32	45,67	43,7299997	35,4200006	31,8599999	sp|P13647|K2C5_HUMAN	Keratin, type II cytoskeletal 5 OS=Homo sapier	HUMAN	27
7	0	43,67	41,049999	32,7699989	29,2199999	sp|A5A6M8|K2C5_PANTR	Keratin, type II cytoskeletal 5 OS=Pan troglody	PANTR	26
12	11,56	42	44,8599994	32,4499995	30,3200006	sp|P48668|K2C6C_HUMAN	Keratin, type II cytoskeletal 6C OS=Homo sapi	HUMAN	26
12	0,02	40,11	44,1500008	30,6699991	28,549999	sp|P02538|K2C6A_HUMAN	Keratin, type II cytoskeletal 6A OS=Homo sapi	HUMAN	25
6	34,05	42,02	67,1599984	50,849998	46,1899996	sp|P02533|K1C14_HUMAN	Keratin, type I cytoskeletal 14 OS=Homo sapie	HUMAN	24
9	14,55	38,98	63,6399984	51,8000007	44,8199987	sp|P08779|K1C16_HUMAN	Keratin, type I cytoskeletal 16 OS=Homo sapie	HUMAN	20
16	8	21,47	39,8099989	28,0099988	25	sp|Q04695|K1C17_HUMAN	Keratin, type I cytoskeletal 17 OS=Homo sapie	HUMAN	11
16	0	21,46	36,8099988	28,0099988	25	sp|A5A6M0|K1C17_PANTR	Keratin, type I cytoskeletal 17 OS=Pan troglody	PANTR	11
16	0	19,04	31,1800003	21,9400004	20,5500007	sp|Q9QWL7|K1C17_MOUSE	Keratin, type I cytoskeletal 17 OS=Mus muscul	MOUSE	10
16	0	19,04	31,1800003	21,9400004	20,5500007	sp|Q6IFU8|K1C17_RAT	Keratin, type I cytoskeletal 17 OS=Rattus norve	RAT	10
8	16,27	16,27	10,52	4,80699986	3,62199992	sp|P15924|DESP_HUMAN	Desmoplakin OS=Homo sapiens GN=DSP PE=1	HUMAN	8
10	14	14	37,7200007	37,7200007	37,7200007	sp|P06702|S10A9_HUMAN	Protein S100-A9 OS=Homo sapiens GN=S100A	HUMAN	8
13	9,14	9,14	59,1400027	59,1400027	59,1400027	sp|P05109|S10A8_HUMAN	Protein S100-A8 OS=Homo sapiens GN=S100A	HUMAN	8
29	2	11,52	8,80099982	8,80099982	8,80099982	sp|P19013|K2C4_HUMAN	Keratin, type II cytoskeletal 4 OS=Homo sapier	HUMAN	7
11	12,11	12,11	18,1199998	15,5699998	13,29	sp|Q8WNW3|PLAK_PIG	Junction plakoglobin OS=Sus scrofa GN=Jup PE	PIG	6
11	0	12,11	18,1199998	15,5699998	13,29	sp|Q8SPJ1|PLAK_BOVIN	Junction plakoglobin OS=Bos taurus GN=JUP P	BOVIN	6
11	0	12,11	18,1199998	15,5699998	13,29	sp|Q02257|PLAK_MOUSE	Junction plakoglobin OS=Mus musculus GN=Ju	MOUSE	6
11	0	12,11	18,1199998	15,5699998	13,29	sp|P14923|PLAK_HUMAN	Junction plakoglobin OS=Homo sapiens GN=JU	HUMAN	6
11	0	11,81	15,8399999	13,29	13,29	sp|Q6P0K8|PLAK_RAT	Junction plakoglobin OS=Rattus norvegicus GN	RAT	6
22	4,03	11,6	15,0000006	6,92299977	6,92299977	sp|Q8N1N4|K2C78_HUMAN	Keratin, type II cytoskeletal 78 OS=Homo sapie	HUMAN	6
15	8,06	8,06	60,2800012	46,0999995	46,0999995	sp|P01037|CYTN_HUMAN	Cystatin-SN OS=Homo sapiens GN=CST1 PE=1	HUMAN	5
17	8	8	11,3300003	8,86700004	8,86700004	sp|P02768|ALBU_HUMAN	Serum albumin OS=Homo sapiens GN=ALB PE=	HUMAN	5
14	8,14	8,14	71,0099995	44,9299991	38,409999	sp|P15252|REF_HEVBR	Rubber elongation factor protein OS=Hevea br	HEVBR	4
17	0	6	10,5099998	6,56799972	6,56799972	sp|Q5NVH5|ALBU_PONAB	Serum albumin OS=Pongo abelii GN=ALB PE=2	PONAB	4
18	7,64	7,64	23,1399998	13,82	13,82	sp|Q5T749|KPRP_HUMAN	Keratinocyte proline-rich protein OS=Homo sa	HUMAN	4
19	6,33	6,33	55,4799974	35,620001	23,2899994	sp|P12273|PIP_HUMAN	Prolactin-inducible protein OS=Homo sapiens	HUMAN	3
20	6,02	6,02	56,3600004	54,549998	54,549998	sp|P81605|DCD_HUMAN	Dermcidin OS=Homo sapiens GN=DCD PE=1 S	HUMAN	3
21	6	6	9,24699977	5,1479999	5,1479999	sp|Q02413|DSG1_HUMAN	Desmoglein-1 OS=Homo sapiens GN=DSG1 PE	HUMAN	3
30	2	4,55	6,72099963	4,88799997	4,88799997	sp|Q6IFX1|K1C24_RAT	Keratin, type I cytoskeletal 24 OS=Rattus norve	RAT	3
30	0	4,55	6,44500032	4,68799993	4,68799993	sp|A1L317|K1C24_MOUSE	Keratin, type I cytoskeletal 24 OS=Mus muscul	MOUSE	3
23	4,02	4,02	65,8200026	63,29	63,29	sp|P02814|SMR3B_HUMAN	Submaxillary gland androgen-regulated protei	HUMAN	2
31	2	4,01	34,7499996	21,2799996	21,2799996	sp|P09228|CYTT_HUMAN	Cystatin-SA OS=Homo sapiens GN=CST2 PE=1	HUMAN	2
39	1,3	1,3	32,7899992	32,7899992	32,7899992	sp|Q8MKD1|UBB_HORSE	Polyubiquitin-B OS=Equus caballus GN=UBB PE	HORSE	2
39	0	1,3	32,8900009	32,8900009	32,8900009	sp|Q865C5|UBIQ_CAMDR	Ubiquitin OS=Camelus dromedarius PE=3 SV=2	CAMDR	2
39	0	1,3	30,8600008	30,8600008	30,8600008	sp|Q63429|UBC_RAT	Polyubiquitin-C OS=Rattus norvegicus GN=Ubc	RAT	2
39	0	1,3	39,6800011	39,6800011	39,6800011	sp|P84589|UBIQ_LUMTE	Ubiquitin (Fragment) OS=Lumbricus terrestris	LUMTE	2
39	0	1,3	16,0300002	16,0300002	16,0300002	sp|P79781|RS27A_CHICK	Ubiquitin-40S ribosomal protein S27a OS=Gall	CHICK	2
39	0	1,3	19,5299998	19,5299998	19,5299998	sp|P68205|RL40_OPHHA	Ubiquitin-60S ribosomal protein L40 OS=Ophic	OPHHA	2
39	0	1,3	16,0300002	16,0300002	16,0300002	sp|P68203|RS27A_SPOFR	Ubiquitin-40S ribosomal protein S27a OS=Spo	SPOFR	2
39	0	1,3	16,1300004	16,1300004	16,1300004	sp|P68202|RS27A_PLUXY	Ubiquitin-40S ribosomal protein S27a OS=Plut	PLUXY	2
39	0	1,3	16,0300002	16,0300002	16,0300002	sp|P68200|RS27A_ICTPU	Ubiquitin-40S ribosomal protein S27a OS=Ictal	ICTPU	2
39	0	1,3	32,8900009	32,8900009	32,8900009	sp|P68197|UBIQ_CERCA	Ubiquitin OS=Ceratitis capitata PE=1 SV=1	CERCA	2
39	0	1,3	19,5299998	19,5299998	19,5299998	sp|P63053|RL40_PIG	Ubiquitin-60S ribosomal protein L40 OS=Sus sc	PIG	2
39	0	1,3	19,5299998	19,5299998	19,5299998	sp|P63052|RL40_FELCA	Ubiquitin-60S ribosomal protein L40 OS=Felis c	FELCA	2
39	0	1,3	19,5299998	19,5299998	19,5299998	sp|P63050|RL40_CANLF	Ubiquitin-60S ribosomal protein L40 OS=Canis	CANLF	2
39	0	1,3	19,5299998	19,5299998	19,5299998	sp|P63048|RL40_BOVIN	Ubiquitin-60S ribosomal protein L40 OS=Bos ta	BOVIN	2
39	0	1,3	16,0300002	16,0300002	16,0300002	sp|P62992|RS27A_BOVIN	Ubiquitin-40S ribosomal protein S27a OS=Bos	BOVIN	2
39	0	1,3	19,5299998	19,5299998	19,5299998	sp|P62987|RL40_HUMAN	Ubiquitin-60S ribosomal protein L40 OS=Homo	HUMAN	2
39	0	1,3	19,5299998	19,5299998	19,5299998	sp|P62986|RL40_RAT	Ubiquitin-60S ribosomal protein L40 OS=Rattu	RAT	2
39	0	1,3	19,5299998	19,5299998	19,5299998	sp|P62984|RL40_MOUSE	Ubiquitin-60S ribosomal protein L40 OS=Mus r	MOUSE	2
39	0	1,3	16,0300002	16,0300002	16,0300002	sp|P62983|RS27A_MOUSE	Ubiquitin-40S ribosomal protein S27a OS=Mus	MOUSE	2
39	0	1,3	16,0300002	16,0300002	16,0300002	sp|P62982|RS27A_RAT	Ubiquitin-40S ribosomal protein S27a OS=Ratt	RAT	2
39	0	1,3	16,0300002	16,0300002	16,0300002	sp|P62979|RS27A_HUMAN	Ubiquitin-40S ribosomal protein S27a OS=Hom	HUMAN	2
39	0	1,3	16,0300002	16,0300002	16,0300002	sp|P62978|RS27A_CAVPO	Ubiquitin-40S ribosomal protein S27a OS=Cavi	CAVPO	2
39	0	1,3	30,399999	30,399999	30,399999	sp|P62976|UBIQP_CRIGR	Polyubiquitin OS=Cricetulus griseus PE=2 SV=2	CRIGR	2
39	0	1,3	32,8900009	32,8900009	32,8900009	sp|P62975|UBIQ_RABIT	Ubiquitin OS=Oryctolagus cuniculus PE=1 SV=1	RABIT	2
39	0	1,3	35,3300005	35,3300005	35,3300005	sp|P62972|UBIQP_XENLA	Polyubiquitin (Fragment) OS=Xenopus laevis P	XENLA	2
39	0	1,3	16,1300004	16,1300004	16,1300004	sp|P29504|RS27A_MANSE	Ubiquitin-40S ribosomal protein S27a OS=Mar	MANSE	2
39	0	1,3	19,5299998	19,5299998	19,5299998	sp|P18101|RL40_DROME	Ubiquitin-60S ribosomal protein L40 OS=Drosc	DROME	2
39	0	1,3	16,0300002	16,0300002	16,0300002	sp|P15357|RS27A_DROME	Ubiquitin-40S ribosomal protein S27a OS=Dros	DROME	2
39	0	1,3	32,6099992	32,6099992	32,6099992	sp|P0CH28|UBC_BOVIN	Polyubiquitin-C OS=Bos taurus GN=UBC PE=1 S	BOVIN	2
39	0	1,3	32,7699989	32,7699989	32,7699989	sp|P0CG69|UBIQP_DROME	Polyubiquitin OS=Drosophila melanogaster GN	DROME	2
39	0	1,3	32,8299999	32,8299999	32,8299999	sp|P0CG68|UBC_PIG	Polyubiquitin-C OS=Sus scrofa GN=UBC PE=2 S	PIG	2
39	0	1,3	32,7499986	32,7499986	32,7499986	sp|P0CG67|UBB_GORGO	Polyubiquitin-B OS=Gorilla gorilla gorilla GN=U	GORGO	2
39	0	1,3	32,8399986	32,8399986	32,8399986	sp|P0CG66|UBC_GORGO	Polyubiquitin-C OS=Gorilla gorilla gorilla GN=U	GORGO	2
39	0	1,3	32,7499986	32,7499986	32,7499986	sp|P0CG65|UBB_PANTR	Polyubiquitin-B OS=Pan troglodytes GN=UBB P	PANTR	2
39	0	1,3	32,8500003	32,8500003	32,8500003	sp|P0CG64|UBC_PANTR	Polyubiquitin-C OS=Pan troglodytes GN=UBC P	PANTR	2
39	0	1,3	32,7899992	32,7899992	32,7899992	sp|P0CG62|UBB_CHICK	Polyubiquitin-B OS=Gallus gallus GN=UBB PE=1	CHICK	2
39	0	1,3	32,8500003	32,8500003	32,8500003	sp|P0CG61|UBC_PONPY	Polyubiquitin-C OS=Pongo pygmaeus GN=UBC	PONPY	2
39	0	1,3	32,7499986	32,7499986	32,7499986	sp|P0CG60|UBB_PONPY	Polyubiquitin-B OS=Pongo pygmaeus GN=UBB	PONPY	2
39	0	1,3	32,7899992	32,7899992	32,7899992	sp|P0CG55|UBB_SHEEP	Polyubiquitin-B OS=Ovis aries GN=UBB PE=2 S	SHEEP	2
39	0	1,3	32,1500003	32,1500003	32,1500003	sp|P0CG54|UBB_CAVPO	Polyubiquitin-B OS=Cavia porcellus GN=UBB P	CAVPO	2
39	0	1,3	32,7899992	32,7899992	32,7899992	sp|P0CG53|UBB_BOVIN	Polyubiquitin-B OS=Bos taurus GN=UBB PE=1 S	BOVIN	2
39	0	1,3	32,7899992	32,7899992	32,7899992	sp|P0CG51|UBB_RAT	Polyubiquitin-B OS=Rattus norvegicus GN=Ubb	RAT	2
39	0	1,3	30,6499988	30,6499988	30,6499988	sp|P0CG50|UBC_MOUSE	Polyubiquitin-C OS=Mus musculus GN=Ubc PE	MOUSE	2
39	0	1,3	32,7899992	32,7899992	32,7899992	sp|P0CG49|UBB_MOUSE	Polyubiquitin-B OS=Mus musculus GN=Ubb PE	MOUSE	2
39	0	1,3	32,8500003	32,8500003	32,8500003	sp|P0CG48|UBC_HUMAN	Polyubiquitin-C OS=Homo sapiens GN=UBC PE	HUMAN	2
39	0	1,3	32,7499986	32,7499986	32,7499986	sp|P0CG47|UBB_HUMAN	Polyubiquitin-B OS=Homo sapiens GN=UBB PE	HUMAN	2
39	0	1,3	19,5299998	19,5299998	19,5299998	sp|P0C276|RL40_SHEEP	Ubiquitin-60S ribosomal protein L40 OS=Ovis a	SHEEP	2
39	0	1,3	19,5299998	19,5299998	19,5299998	sp|P0C275|RL40_PONPY	Ubiquitin-60S ribosomal protein L40 OS=Pongo	PONPY	2
39	0	1,3	19,5299998	19,5299998	19,5299998	sp|P0C273|RL40_MACFA	Ubiquitin-60S ribosomal protein L40 OS=Maca	MACFA	2
24	2,08	2,08	4,14999984	4,14999984	2,14200001	sp|Q13835|PKP1_HUMAN	Plakophilin-1 OS=Homo sapiens GN=PKP1 PE=	HUMAN	1
24	0	2,08	4,25800011	4,25800011	2,19800007	sp|P97350|PKP1_MOUSE	Plakophilin-1 OS=Mus musculus GN=Pkp1 PE=	MOUSE	1

25	2,05	2,05	9,06499997	4,81600016	4,81600016	sp|P20758|IGHA1_GORGO	Ig alpha-1 chain C region OS=Gorilla gorilla gor	GORGO	1
25	0	2,05	9,06499997	4,81600016	4,81600016	sp|P01876|IGHA1_HUMAN	Ig alpha-1 chain C region OS=Homo sapiens GI	HUMAN	1
25	0	2	5,00000007	5,00000007	5,00000007	sp|P01877|IGHA2_HUMAN	Ig alpha-2 chain C region OS=Homo sapiens GI	HUMAN	1
26	2,01	2,01	12,7499998	4,02700007	4,02700007	sp|P25311|ZA2G_HUMAN	Zinc-alpha-2-glycoprotein OS=Homo sapiens G	HUMAN	1
27	2,01	2,01	26,4200002	26,4200002	26,4200002	sp|Q5R9M3|THIO_PONAB	Thioredoxin OS=Pongo abelii GN=TXN PE=3 SV	PONAB	1
27	0	2,01	26,67	26,67	26,67	sp|P10599|THIO_HUMAN	Thioredoxin OS=Homo sapiens GN=TXN PE=1 S	HUMAN	1
28	2,01	2,01	5,52899987	4,08699997	4,08699997	sp|P34955|A1AT_BOVIN	Alpha-1-antiproteinase OS=Bos taurus GN=SEI	BOVIN	1
32	2	2	8,072	2,14200001	2,14200001	sp|P02769|ALBU_BOVIN	Serum albumin OS=Bos taurus GN=ALB PE=1 S	BOVIN	1
33	2	2	2,44999994	1,45699997	1,45699997	sp|Q7SIH1|A2MG_BOVIN	Alpha-2-macroglobulin OS=Bos taurus GN=A2I	BOVIN	1
34	2	2	3,16799991	1,18800001	1,18800001	sp|P33610|PRI2_MOUSE	DNA primase large subunit OS=Mus musculus	MOUSE	1
34	0	2	3,156	1,18300002	1,18300002	sp|O89044|PRI2_RAT	DNA primase large subunit OS=Rattus norvegi	RAT	1
34	0	2	0,79889996	0,79889996	0,79889996	sp|Q9R6X3|PHYB_NOSS1	Cyanobacterial phytochrome B OS=Nostoc sp.	NOSS1	1
34	0	2	2,66699996	2,66699996	2,66699996	sp|Q5WGU8|KCY_BACSK	Cytidylate kinase OS=Bacillus clausii (strain KSI	BACSK	1
34	0	2	1,28499996	1,28499996	1,28499996	sp|Q5UNS8|YR665_MIMIV	Uncharacterized protein R665 OS=Acanthamo	MIMIV	1
34	0	2	0,6904	0,6904	0,6904	sp|Q03279|PO21_BRACO	Retrovirus-related Pol polyprotein from type-1	BRACO	1
34	0	2	0,48500001	0,48500001	0,48500001	sp|P53254|UTP22_YEAST	U3 small nucleolar RNA-associated protein 22	YEAST	1
34	0	2	1,17899999	1,17899999	1,17899999	sp|P49643|PRI2_HUMAN	DNA primase large subunit OS=Homo sapiens	HUMAN	1
34	0	2	0,63029998	0,63029998	0,63029998	sp|P0CN43|EIF3A_CRYNB	Eukaryotic translation initiation factor 3 subun	CRYNB	1
34	0	2	0,63029998	0,63029998	0,63029998	sp|P0CN42|EIF3A_CRYNJ	Eukaryotic translation initiation factor 3 subun	CRYNJ	1
34	0	2	2,24699993	2,24699993	2,24699993	sp|B3QPB1|TRPA_CHLP8	Tryptophan synthase alpha chain OS=Chloroba	CHLP8	1
34	0	2	1,91099998	1,91099998	1,91099998	sp|A4IHY0|OXND1_XENTR	Oxidoreductase NAD-binding domain-containi	XENTR	1
34	0	2	2,128	2,128	2,128	RRRRRsp|Q9TLX3|LPXC_CYACA	REVERSED UDP-3-O-[3-hydroxymyristoyl] N-ac	CYACA	1
34	0	2	0,96930005	0,96930005	0,96930005	RRRRRsp|Q9LTX2|TIR1L_ARATH	REVERSED Transport inhibitor response 1-like	ARATH	1
34	0	2	1,143	1,143	1,143	RRRRRsp|Q9ESB3|HRG_MOUSE	REVERSED Histidine-rich glycoprotein OS=Mus	MOUSE	1
34	0	2	1,143	1,143	1,143	RRRRRsp|Q99PS8|HRG_RAT	REVERSED Histidine-rich glycoprotein OS=Ratt	RAT	1
34	0	2	1,47799999	1,47799999	1,47799999	RRRRRsp|Q8FGZ9|ASTC_ECOL6	REVERSED Succinylornithine transaminase OS=	ECOL6	1
34	0	2	1,47799999	1,47799999	1,47799999	RRRRRsp|Q1RB45|ASTC_ECOUT	REVERSED Succinylornithine transaminase OS=	ECOUT	1
34	0	2	1,47799999	1,47799999	1,47799999	RRRRRsp|Q0TH82|ASTC_ECOL5	REVERSED Succinylornithine transaminase OS=	ECOL5	1
34	0	2	1,04	1,04	1,04	RRRRRsp|Q0I3B0|SYR_HAES1	REVERSED Arginine--tRNA ligase OS=Haemoph	HAES1	1
34	0	2	0,36859999	0,36859999	0,36859999	RRRRRsp|Q09779|THO2_SCHPO	REVERSED THO complex subunit 2 OS=Schizos	SCHPO	1
34	0	2	1,81300007	1,81300007	1,81300007	RRRRRsp|Q01558|G3PC_LEIME	REVERSED Glyceraldehyde-3-phosphate dehyc	LEIME	1
34	0	2	0,84149996	0,84149996	0,84149996	RRRRRsp|P53276|UTP8_YEAST	REVERSED U3 small nucleolar RNA-associated	YEAST	1
34	0	2	0,48349998	0,48349998	0,48349998	RRRRRsp|P23634|AT2B4_HUMAN	REVERSED Plasma membrane calcium-transpo	HUMAN	1
34	0	2	1,81300007	1,81300007	1,81300007	RRRRRsp|P0A9B5|G3P_SHIFL	REVERSED Glyceraldehyde-3-phosphate dehyc	SHIFL	1
34	0	2	1,81300007	1,81300007	1,81300007	RRRRRsp|P0A9B4|G3P1_ECO57	REVERSED Glyceraldehyde-3-phosphate dehyc	ECO57	1
34	0	2	1,81300007	1,81300007	1,81300007	RRRRRsp|P0A9B3|G3P_ECOL6	REVERSED Glyceraldehyde-3-phosphate dehyc	ECOL6	1
34	0	2	1,81300007	1,81300007	1,81300007	RRRRRsp|P0A9B2|G3P1_ECOLI	REVERSED Glyceraldehyde-3-phosphate dehyc	ECOLI	1
34	0	2	1,81300007	1,81300007	1,81300007	RRRRRsp|P0A1P1|G3P_SALTI	REVERSED Glyceraldehyde-3-phosphate dehyc	SALTI	1
34	0	2	1,81300007	1,81300007	1,81300007	RRRRRsp|P0A1P0|G3P_SALTY	REVERSED Glyceraldehyde-3-phosphate dehyc	SALTY	1
34	0	2	1,81300007	1,81300007	1,81300007	RRRRRsp|I2BA89|G3P_SHIBC	REVERSED Glyceraldehyde-3-phosphate dehyc	SHIBC	1
34	0	2	0,49709999	0,49709999	0,49709999	RRRRRsp|D3K0R6|AT2B4_BOVIN	REVERSED Plasma membrane calcium-transpo	BOVIN	1
34	0	2	1,47799999	1,47799999	1,47799999	RRRRRsp|B7USC9|ASTC_ECO27	REVERSED Succinylornithine transaminase OS=	ECO27	1
34	0	2	1,47799999	1,47799999	1,47799999	RRRRRsp|B7NT29|ASTC_ECO7I	REVERSED Succinylornithine transaminase OS=	ECO7I	1
34	0	2	1,47799999	1,47799999	1,47799999	RRRRRsp|B7N584|ASTC_ECOLU	REVERSED Succinylornithine transaminase OS=	ECOLU	1
34	0	2	1,47799999	1,47799999	1,47799999	RRRRRsp|B7MVM7|ASTC_ECO81	REVERSED Succinylornithine transaminase OS=	ECO81	1
34	0	2	1,47799999	1,47799999	1,47799999	RRRRRsp|B7MAV9|ASTC_ECO45	REVERSED Succinylornithine transaminase OS=	ECO45	1
34	0	2	1,47799999	1,47799999	1,47799999	RRRRRsp|B7LQ44|ASTC_ESCF3	REVERSED Succinylornithine transaminase OS=	ESCF3	1
34	0	2	1,81300007	1,81300007	1,81300007	RRRRRsp|B7LQ20|G3P_ESCF3	REVERSED Glyceraldehyde-3-phosphate dehyc	ESCF3	1
34	0	2	1,47799999	1,47799999	1,47799999	RRRRRsp|B1LDY3|ASTC_ECOSM	REVERSED Succinylornithine transaminase OS=	ECOSM	1
34	0	2	1,04	1,04	1,04	RRRRRsp|B0UUF1|SYR_HISS2	REVERSED Arginine--tRNA ligase OS=Histophil	HISS2	1
34	0	2	1,47799999	1,47799999	1,47799999	RRRRRsp|A1ABS9|ASTC_ECOK1	REVERSED Succinylornithine transaminase OS=	ECOK1	1
35	2	2	5,9390001	1,76599994	1,76599994	sp|P21910|LAML2_XENLA	Lamin-L(II) OS=Xenopus laevis PE=2 SV=1	XENLA	1
36	2	2	26,3500005	8,10799971	8,10799971	sp|P17897|LYZ1_MOUSE	Lysozyme C-1 OS=Mus musculus GN=Lyz1 PE=	MOUSE	1
36	0	2	8,3920002	8,3920002	8,3920002	sp|Q9PU28|LYSC_SCOMX	Lysozyme C OS=Scophthalmus maximus GN=ly	SCOMX	1
36	0	2	8,3920002	8,3920002	8,3920002	sp|Q9DD65|LYSC_PAROL	Lysozyme C OS=Paralichthys olivaceus PE=2 S\	PAROL	1
36	0	2	8,10799971	8,10799971	8,10799971	sp|Q6B411|LYSM_BOVIN	Lysozyme C, milk isozyme OS=Bos taurus PE=2	BOVIN	1
36	0	2	8,10799971	8,10799971	8,10799971	sp|Q659U5|LYSC_HALGR	Lysozyme C OS=Halichoerus grypus GN=LYZ PE	HALGR	1
36	0	2	8,10799971	8,10799971	8,10799971	sp|Q659U1|LYSC_PHOVI	Lysozyme C OS=Phoca vitulina GN=LYZ PE=2 S\	PHOVI	1
36	0	2	8,10799971	8,10799971	8,10799971	sp|Q659U0|LYSC_LEPWE	Lysozyme C OS=Leptonychotes weddellii GN=L	LEPWE	1
36	0	2	8,10799971	8,10799971	8,10799971	sp|Q05820|LYSC2_RAT	Putative lysozyme C-2 OS=Rattus norvegicus G	RAT	1
36	0	2	9,23099965	9,23099965	9,23099965	sp|P85345|LYSC_AMYCA	Lysozyme C OS=Amyda cartilaginea GN=LYZ PE	AMYCA	1
36	0	2	8,21899995	8,21899995	8,21899995	sp|P85045|LYS_BUFGA	Lysozyme C (Fragment) OS=Bufo gargarizans a	BUFGA	1
36	0	2	9,23099965	9,23099965	9,23099965	sp|P81709|LYSC2_CANLF	Lysozyme C, spleen isozyme OS=Canis lupus fa	CANLF	1
36	0	2	8,10799971	8,10799971	8,10799971	sp|P79847|LYSC_PYGNE	Lysozyme C OS=Pygathrix nemaeus GN=LYZ PE	PYGNE	1
36	0	2	8,10799971	8,10799971	8,10799971	sp|P79811|LYSC_NASLA	Lysozyme C OS=Nasalis larvatus GN=LYZ PE=3	NASLA	1
36	0	2	8,10799971	8,10799971	8,10799971	sp|P79239|LYSC_PONPY	Lysozyme C OS=Pongo pygmaeus GN=LYZ PE=:	PONPY	1
36	0	2	8,10799971	8,10799971	8,10799971	sp|P79180|LYSC_HYLLA	Lysozyme C OS=Hylobates lar GN=LYZ PE=2 SV	HYLLA	1
36	0	2	8,10799971	8,10799971	8,10799971	sp|P79179|LYSC_GORGO	Lysozyme C OS=Gorilla gorilla gorilla GN=LYZ P	GORGO	1
36	0	2	8,10799971	8,10799971	8,10799971	sp|P67980|LYSC_TRAFR	Lysozyme C OS=Trachypithecus francoisi GN=L	TRAFR	1
36	0	2	8,10799971	8,10799971	8,10799971	sp|P67979|LYSC_TRAOB	Lysozyme C OS=Trachypithecus obscurus GN=l	TRAOB	1
36	0	2	8,10799971	8,10799971	8,10799971	sp|P67978|LYSC_TRAVT	Lysozyme C OS=Trachypithecus vetulus GN=LY	TRAVT	1
36	0	2	8,10799971	8,10799971	8,10799971	sp|P67977|LYSC_SEMEN	Lysozyme C OS=Semnopithecus entellus GN=L	SEMEN	1
36	0	2	8,3920002	8,3920002	8,3920002	sp|P61944|LYSC_TAKRU	Lysozyme C OS=Takifugu rubripes PE=2 SV=1	TAKRU	1
36	0	2	8,10799971	8,10799971	8,10799971	sp|P61632|LYSC_COLGU	Lysozyme C OS=Colobus guereza GN=LYZ PE=2	COLGU	1
36	0	2	8,10799971	8,10799971	8,10799971	sp|P61631|LYSC_COLAN	Lysozyme C OS=Colobus angolensis GN=LYZ PE	COLAN	1
36	0	2	8,10799971	8,10799971	8,10799971	sp|P61628|LYSC_PANTR	Lysozyme C OS=Pan troglodytes GN=LYZ PE=2	PANTR	1
36	0	2	8,10799971	8,10799971	8,10799971	sp|P61627|LYSC_PANPA	Lysozyme C OS=Pan paniscus GN=LYZ PE=3 SV	PANPA	1
36	0	2	8,10799971	8,10799971	8,10799971	sp|P61626|LYSC_HUMAN	Lysozyme C OS=Homo sapiens GN=LYZ PE=1 S'	HUMAN	1
36	0	2	9,23099965	9,23099965	9,23099965	sp|P37712|LYSC_CAMDR	Lysozyme C OS=Camelus dromedarius GN=LYZ	CAMDR	1
36	0	2	9,23099965	9,23099965	9,23099965	sp|P16973|LYSC_RABIT	Lysozyme C OS=Oryctolagus cuniculus GN=LYZ	RABIT	1
36	0	2	8,10799971	8,10799971	8,10799971	sp|P12069|LYSC3_PIG	Lysozyme C-3 OS=Sus scrofa PE=1 SV=2	PIG	1
36	0	2	8,21899995	8,21899995	8,21899995	sp|P12068|LYSC2_PIG	Lysozyme C-2 OS=Sus scrofa PE=1 SV=2	PIG	1
36	0	2	9,375	9,375	9,375	sp|P12067|LYSC1_PIG	Lysozyme C-1 OS=Sus scrofa PE=1 SV=1	PIG	1
36	0	2	8,3329998	8,3329998	8,3329998	sp|P11941|LYSC2_ONCMY	Lysozyme C II OS=Oncorhynchus mykiss PE=1 S	ONCMY	1
36	0	2	8,10799971	8,10799971	8,10799971	sp|P08905|LYZ2_MOUSE	Lysozyme C-2 OS=Mus musculus GN=Lyz2 PE=	MOUSE	1
36	0	2	8,10799971	8,10799971	8,10799971	sp|P00697|LYSC1_RAT	Lysozyme C-1 OS=Rattus norvegicus GN=Lyz1 I	RAT	1
37	2	2	6,68499991	6,68499991	6,68499991	sp|P12763|FETUA_BOVIN	Alpha-2-HS-glycoprotein OS=Bos taurus GN=A	BOVIN	1
38	1,58	1,58	7,82999992	3,46800014	1,67800002	sp|Q08554|DSC1_HUMAN	Desmocollin-1 OS=Homo sapiens GN=DSC1 PE	HUMAN	1
40	0,77	0,77	13,73	5,07499985	5,07499985	sp|P04406|G3P_HUMAN	Glyceraldehyde-3-phosphate dehydrogenase (	HUMAN	1

40	0	0,75	5,09000011	5,09000011	5,09000011	sp|Q9UVC0|G3P_WICCF	Glyceraldehyde-3-phosphate dehydrogenase (	WICCF	1
40	0	0,75	5,07499985	5,07499985	5,07499985	sp|Q9PJN6|G3P_CHLMU	Glyceraldehyde-3-phosphate dehydrogenase (	CHLMU	1
40	0	0,75	5,07499985	5,07499985	5,07499985	sp|Q92211|G3P_CANAW	Glyceraldehyde-3-phosphate dehydrogenase (	CANAW	1
40	0	0,75	5,13599999	5,13599999	5,13599999	sp|Q01558|G3PC_LEIME	Glyceraldehyde-3-phosphate dehydrogenase,	LEIME	1
40	0	0,75	5,01499996	5,01499996	5,01499996	sp|P9WN83|G3P_MYCTU	Glyceraldehyde-3-phosphate dehydrogenase (	MYCTU	1
40	0	0,75	5,01499996	5,01499996	5,01499996	sp|P9WN82|G3P_MYCTO	Glyceraldehyde-3-phosphate dehydrogenase (	MYCTO	1
40	0	0,75	5,01499996	5,01499996	5,01499996	sp|P64179|G3P_MYCBO	Glyceraldehyde-3-phosphate dehydrogenase (	MYCBO	1
40	0	0,75	4,98499982	4,98499982	4,98499982	sp|P49644|G3PC_CHLRE	Glyceraldehyde-3-phosphate dehydrogenase,	CHLRE	1
40	0	0,75	5,01499996	5,01499996	5,01499996	sp|P48812|G3P_BRUMA	Glyceraldehyde-3-phosphate dehydrogenase (	BRUMA	1
40	0	0,75	5,01499996	5,01499996	5,01499996	sp|P46713|G3P_MYCLE	Glyceraldehyde-3-phosphate dehydrogenase (	MYCLE	1
40	0	0,75	5,02999984	5,02999984	5,02999984	sp|P34922|G3PC_PEA	Glyceraldehyde-3-phosphate dehydrogenase,	PEA	1
40	0	0,75	4,98499982	4,98499982	4,98499982	sp|P32810|G3P3_CAEBR	Glyceraldehyde-3-phosphate dehydrogenase 3	CAEBR	1
40	0	0,75	4,98499982	4,98499982	4,98499982	sp|P32809|G3P2_CAEBR	Glyceraldehyde-3-phosphate dehydrogenase 2	CAEBR	1
40	0	0,75	4,98499982	4,98499982	4,98499982	sp|P17330|G3P3_CAEEL	Glyceraldehyde-3-phosphate dehydrogenase 3	CAEEL	1
40	0	0,75	4,98499982	4,98499982	4,98499982	sp|P17329|G3P2_CAEEL	Glyceraldehyde-3-phosphate dehydrogenase 2	CAEEL	1
40	0	0,75	5,09000011	5,09000011	5,09000011	sp|P0CE13|G3P_CHLTR	Glyceraldehyde-3-phosphate dehydrogenase (	CHLTR	1
40	0	0,75	5,07499985	5,07499985	5,07499985	sp|O59841|G3P_OGAPD	Glyceraldehyde-3-phosphate dehydrogenase (	OGAPD	1
40	0	0,75	5,01499996	5,01499996	5,01499996	sp|O01360|G3P_ONCVO	Glyceraldehyde-3-phosphate dehydrogenase (	ONCVO	1
40	0	0,75	5,09000011	5,09000011	5,09000011	sp|B0B879|G3P_CHLT2	Glyceraldehyde-3-phosphate dehydrogenase (	CHLT2	1
41	0,63	0,63	14,1499996	14,1499996	14,1499996	sp|P0CG06|LAC3_HUMAN	Ig lambda-3 chain C regions OS=Homo sapiens	HUMAN	1
41	0	0,63	14,1499996	14,1499996	14,1499996	sp|P0CG05|LAC2_HUMAN	Ig lambda-2 chain C regions OS=Homo sapiens	HUMAN	1
41	0	0,63	14,1499996	14,1499996	14,1499996	sp|P0CG04|LAC1_HUMAN	Ig lambda-1 chain C regions OS=Homo sapiens	HUMAN	1
41	0	0,63	14,1499996	14,1499996	14,1499996	sp|P0CF74|LAC6_HUMAN	Ig lambda-6 chain C region OS=Homo sapiens (	HUMAN	1
41	0	0,63	7,00900033	7,00900033	7,00900033	sp|B9A064|IGLL5_HUMAN	Immunoglobulin lambda-like polypeptide 5 OS	HUMAN	1
41	0	0,63	14,1499996	14,1499996	14,1499996	sp|A0M8Q6|LAC7_HUMAN	Ig lambda-7 chain C region OS=Homo sapiens (	HUMAN	1
42	0,56	0,56	3,53399999	1,43999998	1,43999998	sp|P01833|PIGR_HUMAN	Polymeric immunoglobulin receptor OS=Homo	HUMAN	1
43	0,3	0,3	6,83199987	3,41599993	0	sp|P05089|ARGI1_HUMAN	Arginase-1 OS=Homo sapiens GN=ARG1 PE=1	HUMAN	0
44	0,28	0,28	1,89399999	1,89399999	0	RRRRRsp|P94282|SYFA_BORBU	REVERSED Phenylalanine--tRNA ligase alpha su	BORBU	0
45	0,19	0,19	2,76800003	2,76800003	0	RRRRRsp|P97281|SGCD_MESAU	REVERSED Delta-sarcoglycan OS=Mesocricetu	MESAU	0
45	0	0,19	2,76800003	2,76800003	0	RRRRRsp|P82347|SGCD_MOUSE	REVERSED Delta-sarcoglycan OS=Mus musculu	MOUSE	0
46	0,12	0,12	1,88299995	1,88299995	0	sp|Q50339|Y588_MYCPN	Uncharacterized lipoprotein MPN_588 OS=My	MYCPN	0

N	Ctrldudoso_CSI_4	Total	%Cov	%Cov(50)	%Cov(95)	Accession	Name	Species	Peptides(95%)
1	109,68	109,68	70,8100021	70,8100021	67,6999986	sp|P04264|K2C1_HUMAN	Keratin, type II cytoskeletal 1 OS=Homo sapiens GN=KRT1 PE=	HUMAN	87
2	91,02	91,02	61,8200004	61,8200004	56,3399971	sp|P13645|K1C10_HUMAN	Keratin, type I cytoskeletal 10 OS=Homo sapiens GN=KRT10 P	HUMAN	80
3	57,75	67,12	68,8600004	60,2500021	58,8400006	sp|P35908|K22E_HUMAN	Keratin, type II cytoskeletal 2 epidermal OS=Homo sapiens GN	HUMAN	47
4	53,23	54,34	64,3700004	62,2799993	59,0699971	sp|P35527|K1C9_HUMAN	Keratin, type I cytoskeletal 9 OS=Homo sapiens GN=KRT9 PE=	HUMAN	40
5	45,59	45,59	65,7999992	65,7999992	59,740001	sp|P00761|TRYP_PIG	Trypsin OS=Sus scrofa PE=1 SV=1	PIG	39
8	20,1	32,82	34,7499996	26,7800003	26,7800003	sp|P13647|K2C5_HUMAN	Keratin, type II cytoskeletal 5 OS=Homo sapiens GN=KRT5 PE=	HUMAN	21
26	0	25,24	29,2600006	23,3999997	19,4999993	sp|P04259|K2C6B_HUMAN	Keratin, type II cytoskeletal 6B OS=Homo sapiens GN=KRT6B F	HUMAN	21
8	0	30,82	32,0899993	24,1600007	24,1600007	sp|A5A6M8|K2C5_PANTR	Keratin, type II cytoskeletal 5 OS=Pan troglodytes GN=KRT5 PE	PANTR	20
26	4,52	25,25	32,980001	25,3500015	19,3299994	sp|P48668|K2C6C_HUMAN	Keratin, type II cytoskeletal 6C OS=Homo sapiens GN=KRT6C F	HUMAN	19
26	0	25,24	30,8499992	23,2299998	21,0999995	sp|P02538|K2C6A_HUMAN	Keratin, type II cytoskeletal 6A OS=Homo sapiens GN=KRT6A I	HUMAN	19
7	25,71	31,8	50,4199982	45,3399986	37,9200011	sp|P02533|K1C14_HUMAN	Keratin, type I cytoskeletal 14 OS=Homo sapiens GN=KRT14 P	HUMAN	16
23	5,9	16,21	20,07	16,6099995	15,05	sp|Q7Z794|K2C1B_HUMAN	Keratin, type II cytoskeletal 1b OS=Homo sapiens GN=KRT77 F	HUMAN	15
6	26,33	26,33	44,0800011	29,58	20,2800006	sp|P02788|TRFL_HUMAN	Lactotransferrin OS=Homo sapiens GN=LTF PE=1 SV=6	HUMAN	12
9	19,85	19,85	29,0600002	23,5599995	21,9899997	sp|P01833|PIGR_HUMAN	Polymeric immunoglobulin receptor OS=Homo sapiens GN=PI	HUMAN	11
25	4,92	18,53	26,2199998	25,5800009	20,51	sp|P08779|K1C16_HUMAN	Keratin, type I cytoskeletal 16 OS=Homo sapiens GN=KRT16 P	HUMAN	10
37	2,11	14,59	13,6500001	7,88500011	5,38499989	sp|Q8N1N4|K2C78_HUMAN	Keratin, type II cytoskeletal 78 OS=Homo sapiens GN=KRT78 F	HUMAN	10
12	15,02	15,02	47,8799999	40,7900006	40,7900006	sp|P01876|IGHA1_HUMAN	Ig alpha-1 chain C region OS=Homo sapiens GN=IGHA1 PE=1 S	HUMAN	9
10	15,55	15,55	55,4799974	55,4799974	55,4799974	sp|P12273|PIP_HUMAN	Prolactin-inducible protein OS=Homo sapiens GN=PIP PE=1 SV	HUMAN	8
11	15,19	15,19	83,3299994	71,0099995	71,0099995	sp|P15252|REF_HEVBR	Rubber elongation factor protein OS=Hevea brasiliensis PE=1	HEVBR	8
39	2,01	14,28	21,9899997	18,9799994	16,9	sp|Q04695|K1C17_HUMAN	Keratin, type I cytoskeletal 17 OS=Homo sapiens GN=KRT17 P	HUMAN	8
39	0	14,28	21,9899997	18,9799994	16,9	sp|A5A6M0|K1C17_PANTR	Keratin, type I cytoskeletal 17 OS=Pan troglodytes GN=KRT17	PANTR	8
13	13,81	13,81	43,2900012	31,8800002	28,52	sp|P25311|ZA2G_HUMAN	Zinc-alpha-2-glycoprotein OS=Homo sapiens GN=AZGP1 PE=1	HUMAN	7
14	13,05	13,05	15,4400006	10,87	10,87	sp|Q02413|DSG1_HUMAN	Desmoglein-1 OS=Homo sapiens GN=DSG1 PE=1 SV=2	HUMAN	7
40	2,01	11,03	49,7099996	30,0000012	30,0000012	sp|P01877|IGHA2_HUMAN	Ig alpha-2 chain C region OS=Homo sapiens GN=IGHA2 PE=1 S	HUMAN	7
16	9,85	9,85	17,08	14,4500002	11,9900003	sp|Q5NVH5|ALBU_PONAB	Serum albumin OS=Pongo abelii GN=ALB PE=2 SV=2	PONAB	6
16	0	9,85	17,08	14,4500002	11,9900003	sp|P02768|ALBU_HUMAN	Serum albumin OS=Homo sapiens GN=ALB PE=1 SV=2	HUMAN	6
17	8,81	8,81	44,1199988	35,2899998	35,2899998	sp|O82803|SRPP_HEVBR	Small rubber particle protein OS=Hevea brasiliensis GN=SRPP	HEVBR	6
15	10	10	70,9699988	70,9699988	70,9699988	sp|P05109|S10A8_HUMAN	Protein S100-A8 OS=Homo sapiens GN=S100A8 PE=1 SV=1	HUMAN	5
19	8,01	8,01	64,1499996	64,1499996	64,1499996	sp|P01834|IGKC_HUMAN	Ig kappa chain C region OS=Homo sapiens GN=IGKC PE=1 SV=1	HUMAN	5
21	7,6	7,6	49,12	37,7200007	37,7200007	sp|P06702|S10A9_HUMAN	Protein S100-A9 OS=Homo sapiens GN=S100A9 PE=1 SV=1	HUMAN	5
18	8,58	8,58	31,9499999	15,1999995	13,6399999	sp|Q5T749|KPRP_HUMAN	Keratinocyte proline-rich protein OS=Homo sapiens GN=KPRP	HUMAN	4
20	8	8	56,3600004	56,3600004	56,3600004	sp|P81605|DCD_HUMAN	Dermcidin OS=Homo sapiens GN=DCD PE=1 SV=2	HUMAN	4
22	6,07	6,07	22,0899999	13,73	13,73	sp|P04406|G3P_HUMAN	Glyceraldehyde-3-phosphate dehydrogenase OS=Homo sapie	HUMAN	3
24	5,35	5,35	39,8600012	39,8600012	31,7600012	sp|P79239|LYSC_PONPY	Lysozyme C OS=Pongo pygmaeus GN=LYZ PE=2 SV=1	PONPY	3
24	0	5,35	39,8600012	39,8600012	31,7600012	sp|P79179|LYSC_GORGO	Lysozyme C OS=Gorilla gorilla gorilla GN=LYZ PE=2 SV=1	GORGO	3
24	0	5,35	39,8600012	39,8600012	31,7600012	sp|P61628|LYSC_PANTR	Lysozyme C OS=Pan troglodytes GN=LYZ PE=2 SV=1	PANTR	3
24	0	5,35	39,8600012	39,8600012	31,7600012	sp|P61627|LYSC_PANPA	Lysozyme C OS=Pan paniscus GN=LYZ PE=3 SV=1	PANPA	3
24	0	5,35	39,8600012	39,8600012	31,7600012	sp|P61626|LYSC_HUMAN	Lysozyme C OS=Homo sapiens GN=LYZ PE=1 SV=1	HUMAN	3
27	0	4,35	22,9300007	17,0699999	17,0699999	sp|Q91ZK5|ACTB_SIGHI	Actin, cytoplasmic 1 OS=Sigmodon hispidus GN=ACTB PE=2 SV	SIGHI	3
27	0	4,35	22,9300007	17,0699999	17,0699999	sp|Q8JJB8|ACTG_TRISC	Actin, cytoplasmic 2 OS=Triakis scyllium GN=actg1 PE=2 SV=1	TRISC	3
27	0	4,35	22,9300007	17,0699999	17,0699999	sp|Q7ZVI7|ACTB1_DANRE	Actin, cytoplasmic 1 OS=Danio rerio GN=actba PE=2 SV=2	DANRE	3
27	0	4,35	22,9300007	17,0699999	17,0699999	sp|Q7ZVF9|ACTB2_DANRE	Actin, cytoplasmic 2 OS=Danio rerio GN=actbb PE=2 SV=2	DANRE	3
27	0	4,35	22,9300007	17,0699999	17,0699999	sp|Q76N69|ACTB_CHLAE	Actin, cytoplasmic 1 OS=Chlorocebus aethiops GN=ACTB PE=2	CHLAE	3
27	0	4,35	22,9300007	17,0699999	17,0699999	sp|Q71FK5|ACTB_CAVPO	Actin, cytoplasmic 1 OS=Cavia porcellus GN=ACTB PE=2 SV=1	CAVPO	3
27	0	4,35	22,9300007	17,0699999	17,0699999	sp|Q711N9|ACTB_MESAU	Actin, cytoplasmic 1 OS=Mesocricetus auratus GN=ACTB PE=1	MESAU	3
27	0	4,35	22,9300007	17,0699999	17,0699999	sp|Q6QAQ1|ACTB_PIG	Actin, cytoplasmic 1 OS=Sus scrofa GN=ACTB PE=1 SV=2	PIG	3
27	0	4,35	22,9300007	17,0699999	17,0699999	sp|Q6P378|ACTG_XENTR	Actin, cytoplasmic 2 OS=Xenopus tropicalis GN=actg1 PE=2 SV	XENTR	3
27	0	4,35	22,9300007	17,0699999	17,0699999	sp|Q6NVA9|ACTB_XENTR	Actin, cytoplasmic 1 OS=Xenopus tropicalis GN=actb PE=2 SV=	XENTR	3
27	0	4,35	22,9300007	17,0699999	17,0699999	sp|Q5ZMQ2|ACTG_CHICK	Actin, cytoplasmic 2 OS=Gallus gallus GN=ACTG1 PE=1 SV=1	CHICK	3
27	0	4,35	22,9300007	17,0699999	17,0699999	sp|Q5R6G0|ACTB_PONAB	Actin, cytoplasmic 1 OS=Pongo abelii GN=ACTB PE=2 SV=1	PONAB	3
27	0	4,35	22,9300007	17,0699999	17,0699999	sp|Q5R1X3|ACTB_PANTR	Actin, cytoplasmic 1 OS=Pan troglodytes GN=ACTB PE=2 SV=1	PANTR	3
27	0	4,35	22,9300007	17,0699999	17,0699999	sp|Q5JAK2|ACTG_PELLE	Actin, cytoplasmic 2 OS=Pelophylax lessonae GN=actg1 PE=2 S	PELLE	3
27	0	4,35	22,9300007	17,0699999	17,0699999	sp|Q4R561|ACTB_MACFA	Actin, cytoplasmic 1 OS=Macaca fascicularis GN=ACTB PE=2 SV	MACFA	3
27	0	4,35	22,9300007	17,0699999	17,0699999	sp|Q4L0Y2|ACTB_SPECI	Actin, cytoplasmic 1 OS=Spermophilus citellus GN=ACTB PE=2	SPECI	3
27	0	4,35	23,8199994	17,7300006	17,7300006	sp|P84856|ACTB_CHLPG	Actin, cytoplasmic 1 OS=Chlorocebus pygerythrus GN=ACTB P	CHLPG	3
27	0	4,35	22,9300007	17,0699999	17,0699999	sp|P84336|ACTB_CAMDR	Actin, cytoplasmic 1 OS=Camelus dromedarius GN=ACTB PE=1	CAMDR	3
27	0	4,35	22,9300007	17,0699999	17,0699999	sp|P83751|ACTB_CTEID	Actin, cytoplasmic 1 OS=Ctenopharyngodon idella GN=actb PE	CTEID	3
27	0	4,35	22,9300007	17,0699999	17,0699999	sp|P83750|ACTB_CYPCA	Actin, cytoplasmic 1 OS=Cyprinus carpio GN=actb PE=3 SV=1	CYPCA	3
27	0	4,35	22,9300007	17,0699999	17,0699999	sp|P68143|ACTB_OREMO	Actin, cytoplasmic 1 OS=Oreochromis mossambicus GN=actb I	OREMO	3
27	0	4,35	22,9300007	17,0699999	17,0699999	sp|P68142|ACTB1_TAKRU	Actin, cytoplasmic 1 OS=Takifugu rubripes GN=actba PE=2 SV=	TAKRU	3
27	0	4,35	22,9300007	17,0699999	17,0699999	sp|P63261|ACTG_HUMAN	Actin, cytoplasmic 2 OS=Homo sapiens GN=ACTG1 PE=1 SV=1	HUMAN	3
27	0	4,35	22,9300007	17,0699999	17,0699999	sp|P63260|ACTG_MOUSE	Actin, cytoplasmic 2 OS=Mus musculus GN=Actg1 PE=1 SV=1	MOUSE	3
27	0	4,35	22,9300007	17,0699999	17,0699999	sp|P63259|ACTG_RAT	Actin, cytoplasmic 2 OS=Rattus norvegicus GN=Actg1 PE=1 SV	RAT	3
27	0	4,35	22,9300007	17,0699999	17,0699999	sp|P63258|ACTG_BOVIN	Actin, cytoplasmic 2 OS=Bos taurus GN=ACTG1 PE=1 SV=1	BOVIN	3
27	0	4,35	22,9300007	17,0699999	17,0699999	sp|P63257|ACTG_TRIVU	Actin, cytoplasmic 2 OS=Trichosurus vulpecula GN=ACTG1 PE=	TRIVU	3
27	0	4,35	22,9300007	17,0699999	17,0699999	sp|P63256|ACTG_ANSAN	Actin, cytoplasmic 2 OS=Anser anser anser GN=ACTG1 PE=2 SV	ANSAN	3
27	0	4,35	22,9300007	17,0699999	17,0699999	sp|P60713|ACTB_SHEEP	Actin, cytoplasmic 1 OS=Ovis aries GN=ACTB PE=2 SV=1	SHEEP	3
27	0	4,35	22,9300007	17,0699999	17,0699999	sp|P60712|ACTB_BOVIN	Actin, cytoplasmic 1 OS=Bos taurus GN=ACTB PE=1 SV=1	BOVIN	3
27	0	4,35	22,9300007	17,0699999	17,0699999	sp|P60711|ACTB_RAT	Actin, cytoplasmic 1 OS=Rattus norvegicus GN=Actb PE=1 SV=	RAT	3
27	0	4,35	22,9300007	17,0699999	17,0699999	sp|P60710|ACTB_MOUSE	Actin, cytoplasmic 1 OS=Mus musculus GN=Actb PE=1 SV=1	MOUSE	3
27	0	4,35	22,9300007	17,0699999	17,0699999	sp|P60709|ACTB_HUMAN	Actin, cytoplasmic 1 OS=Homo sapiens GN=ACTB PE=1 SV=1	HUMAN	3
27	0	4,35	22,9300007	17,0699999	17,0699999	sp|P60708|ACTB_HORSE	Actin, cytoplasmic 1 OS=Equus caballus GN=ACTB PE=2 SV=1	HORSE	3
27	0	4,35	22,9300007	17,0699999	17,0699999	sp|P60707|ACTB_TRIVU	Actin, cytoplasmic 1 OS=Trichosurus vulpecula GN=ACTB PE=2	TRIVU	3
27	0	4,35	22,9300007	17,0699999	17,0699999	sp|P60706|ACTB_CHICK	Actin, cytoplasmic 1 OS=Gallus gallus GN=ACTB PE=1 SV=1	CHICK	3
27	0	4,35	22,8699997	17,0200005	17,0200005	sp|P53506|ACT8_XENLA	Actin, cytoplasmic type 8 OS=Xenopus laevis PE=3 SV=1	XENLA	3
27	0	4,35	22,8699997	17,0200005	17,0200005	sp|P53505|ACT5_XENLA	Actin, cytoplasmic type 5 OS=Xenopus laevis PE=3 SV=1	XENLA	3
27	0	4,35	22,9300007	17,0699999	17,0699999	sp|P53486|ACTB3_TAKRU	Actin, cytoplasmic 3 OS=Takifugu rubripes GN=actbc PE=2 SV=	TAKRU	3
27	0	4,35	22,9300007	17,0699999	17,0699999	sp|P53485|ACTB2_TAKRU	Actin, cytoplasmic 2 OS=Takifugu rubripes GN=actbb PE=3 SV=	TAKRU	3
27	0	4,35	22,8699997	17,0200005	17,0200005	sp|P53478|ACT5_CHICK	Actin, cytoplasmic type 5 OS=Gallus gallus PE=3 SV=1	CHICK	3
27	0	4,35	22,9300007	17,0699999	17,0699999	sp|P48975|ACTB_CRIGR	Actin, cytoplasmic 1 OS=Cricetulus griseus GN=ACTB PE=3 SV=	CRIGR	3
27	0	4,35	22,8699997	17,0200005	17,0200005	sp|P15475|ACTB_XENBO	Actin, cytoplasmic 1 OS=Xenopus borealis GN=actb PE=3 SV=1	XENBO	3
27	0	4,35	22,9300007	17,0699999	17,0699999	sp|O93400|ACTB_XENLA	Actin, cytoplasmic 1 OS=Xenopus laevis GN=actb PE=2 SV=1	XENLA	3
27	0	4,35	22,9300007	17,0699999	17,0699999	sp|O42161|ACTB_SALSA	Actin, cytoplasmic 1 OS=Salmo salar GN=actb PE=2 SV=1	SALSA	3
27	0	4,35	22,9300007	17,0699999	17,0699999	sp|O18840|ACTB_CANLF	Actin, cytoplasmic 1 OS=Canis lupus familiaris GN=ACTB PE=2	CANLF	3
27	0	4,35	22,9300007	17,0699999	17,0699999	sp|A2BDB0|ACTG_XENLA	Actin, cytoplasmic 2 OS=Xenopus laevis GN=actg1 PE=2 SV=1	XENLA	3
22	0	4,06	17,0100003	8,65700021	8,65700021	sp|Q5RAB4|G3P_PONAB	Glyceraldehyde-3-phosphate dehydrogenase OS=Pongo abelii	PONAB	2
27	4,45	4,45	22,8699997	17,0200005	9,04299989	sp|Q26065|ACT_PLAMG	Actin, adductor muscle OS=Placopecten magellanicus PE=2 SV	PLAMG	2
27	0	4,45	22,8699997	17,0200005	9,04299989	sp|P92182|ACT1_LUMTE	Actin-1 OS=Lumbricus terrestris GN=ACT1 PE=2 SV=1	LUMTE	2
27	0	4,45	22,8699997	17,0200005	9,04299989	sp|P92176|ACT2_LUMTE	Actin-2 OS=Lumbricus terrestris GN=ACT2 PE=2 SV=1	LUMTE	2
27	0	4,45	23,1199995	17,2000006	9,13999975	sp|P91754|ACT_LUMRU	Actin (Fragment) OS=Lumbricus rubellus PE=2 SV=1	LUMRU	2
27	0	4,45	22,8699997	17,0200005	9,04299989	sp|P90689|ACT_BRUMA	Actin OS=Brugia malayi PE=1 SV=1	BRUMA	2
27	0	4,45	22,8699997	17,0200005	9,04299989	sp|P53471|ACT2_SCHMA	Actin-2 OS=Schistosoma mansoni PE=2 SV=1	SCHMA	2
27	0	4,45	22,8699997	17,0200005	9,04299989	sp|P53470|ACT1_SCHMA	Actin-1 OS=Schistosoma mansoni PE=2 SV=1	SCHMA	2
27	0	4,45	22,8699997	17,0200005	9,04299989	sp|P53464|ACTM_HELTB	Actin, cytoskeletal OS=Heliocidaris tuberculata PE=3 SV=1	HELTB	2
27	0	4,45	22,8699997	17,0200005	9,04299989	sp|P53463|ACTM_HELER	Actin, cytoskeletal OS=Heliocidaris erythrogramma PE=3 SV=1	HELER	2
27	0	4,45	23,1800005	17,2499999	9,1640003	sp|P53458|ACT5_DIPDE	Actin-5 (Fragment) OS=Diphyllobothrium dendriticum GN=AC	DIPDE	2

27	0	4,45	22,9300007	17,0699999	9,0669997	sp|P02578|ACT1_ACACA	Actin-1 OS=Acanthamoeba castellanii PE=1 SV=1	ACACA	2
27	0	4,45	22,8100002	16,9799998	9,01900008	sp|O65316|ACT_MESVI	Actin OS=Mesostigma viride PE=3 SV=1	MESVI	2
27	0	4,45	22,8699997	17,0200005	9,04299989	sp|O16808|ACT_MAYDE	Actin OS=Mayetiola destructor PE=2 SV=1	MAYDE	2
27	0	4,45	24,2699996	20,7100004	10,9999999	sp|Q03342|ACT3_ECHGR	Actin-3 (Fragment) OS=Echinococcus granulosus GN=ACTIII PE	ECHGR	2
27	0	4,45	20,0000003	17,0699999	9,0669997	sp|P50138|ACT_PUCGR	Actin OS=Puccinia graminis PE=3 SV=1	PUCGR	2
27	0	4,01	22,9300007	9,0669997	9,0669997	sp|P48465|ACT_CRYNH	Actin OS=Cryptococcus neoformans var. grubii serotype A (str	CRYNH	2
27	0	4	14,9299994	9,0669997	9,0669997	sp|Q9Y707|ACT2_SUIBO	Actin-2 OS=Suillus bovinus GN=ACT2 PE=2 SV=1	SUIBO	2
27	0	4	14,9299994	9,0669997	9,0669997	sp|Q9Y702|ACT1_SCHCO	Actin-1 OS=Schizophyllum commune GN=ACT1 PE=2 SV=1	SCHCO	2
27	0	4	14,9299994	9,0669997	9,0669997	sp|Q9UVX4|ACT_COPC7	Actin OS=Coprinopsis cinerea (strain Okayama-7 / 130 / ATCC	COPC7	2
27	0	4	14,9299994	9,0669997	9,0669997	sp|Q9UVW9|ACTG_ACRCH	Actin, gamma OS=Acremonium chrysogenum GN=ACT PE=3 S\	ACRCH	2
27	0	4	14,8900002	9,04299989	9,04299989	sp|Q964E3|ACTC_BIOAL	Actin, cytoplasmic OS=Biomphalaria alexandrina PE=3 SV=1	BIOAL	2
27	0	4	14,8900002	9,04299989	9,04299989	sp|Q964E2|ACTC_BIOPF	Actin, cytoplasmic OS=Biomphalaria pfeifferi PE=3 SV=1	BIOPF	2
27	0	4	14,8900002	9,04299989	9,04299989	sp|Q964E1|ACTC_BIOOB	Actin, cytoplasmic OS=Biomphalaria obstructa PE=3 SV=1	BIOOB	2
27	0	4	14,8900002	9,04299989	9,04299989	sp|Q964E0|ACTC_BIOTE	Actin, cytoplasmic OS=Biomphalaria tenagophila PE=3 SV=1	BIOTE	2
27	0	4	14,8900002	9,04299989	9,04299989	sp|Q964D9|ACTC_PLATR	Actin, cytoplasmic OS=Planorbella trivolvis PE=3 SV=1	PLATR	2
27	0	4	14,9299994	9,0669997	9,0669997	sp|Q93131|ACTC_BRAFL	Actin, cytoplasmic OS=Branchiostoma floridae PE=2 SV=1	BRAFL	2
27	0	4	14,9299994	9,0669997	9,0669997	sp|Q93129|ACTC_BRABE	Actin, cytoplasmic OS=Branchiostoma belcheri PE=2 SV=1	BRABE	2
27	0	4	14,9299994	9,0669997	9,0669997	sp|Q8X119|ACT_EXODE	Actin OS=Exophiala dermatitidis PE=3 SV=1	EXODE	2
27	0	4	14,9299994	9,0669997	9,0669997	sp|Q6TCF2|ACT_GAEGA	Actin OS=Gaeumannomyces graminis var. avenae GN=ACT PE:	GAEGA	2
27	0	4	14,8900002	9,04299989	9,04299989	sp|Q553U6|ACT22_DICDI	Putative actin-22 OS=Dictyostelium discoideum GN=act22 PE=	DICDI	2
27	0	4	14,8900002	9,04299989	9,04299989	sp|Q54GX7|ACT10_DICDI	Actin-10 OS=Dictyostelium discoideum GN=act10 PE=1 SV=1	DICDI	2
27	0	4	14,8100004	8,99500027	8,99500027	sp|Q25472|ACT2_MOLOC	Actin, muscle-type OS=Molgula oculata PE=3 SV=1	MOLOC	2
27	0	4	14,8900002	9,04299989	9,04299989	sp|Q25010|ACT3A_HELAM	Actin, cytoplasmic A3a OS=Helicoverpa armigera GN=actA3a F	HELAM	2
27	0	4	14,8900002	9,04299989	9,04299989	sp|Q07903|ACTC_STRPU	Actin, cytoskeletal 2A OS=Strongylocentrotus purpuratus GN=	STRPU	2
27	0	4	14,9299994	9,0669997	9,0669997	sp|Q00215|ACTC_STYPL	Actin, cytoplasmic OS=Styela plicata PE=3 SV=1	STYPL	2
27	0	4	14,8900002	9,04299989	9,04299989	sp|P92179|ACTC_BIOGL	Actin, cytoplasmic OS=Biomphalaria glabrata PE=2 SV=2	BIOGL	2
27	0	4	14,8900002	9,04299989	9,04299989	sp|P84185|ACT5C_ANOGA	Actin-5C OS=Anopheles gambiae GN=Act5C PE=2 SV=1	ANOGA	2
27	0	4	14,8900002	9,04299989	9,04299989	sp|P84184|ACT3B_HELAM	Actin-A3b, cytoplasmic OS=Helicoverpa armigera GN=actA3b I	HELAM	2
27	0	4	14,8900002	9,04299989	9,04299989	sp|P84183|ACT4_BOMMO	Actin, cytoplasmic A4 OS=Bombyx mori GN=A4 PE=2 SV=1	BOMMO	2
27	0	4	14,8900002	9,04299989	9,04299989	sp|P83969|ACT1_BACDO	Actin, indirect flight muscle OS=Bactrocera dorsalis PE=3 SV=1	BACDO	2
27	0	4	14,8900002	9,04299989	9,04299989	sp|P83968|ACT6_DROSI	Actin, indirect flight muscle OS=Drosophila simulans GN=Act8	DROSI	2
27	0	4	14,8900002	9,04299989	9,04299989	sp|P83967|ACT6_DROME	Actin, indirect flight muscle OS=Drosophila melanogaster GN=	DROME	2
27	0	4	14,9299994	9,0669997	9,0669997	sp|P78711|ACT_NEUCR	Actin OS=Neurospora crassa (strain ATCC 24698 / 74-OR23-1A	NEUCR	2
27	0	4	14,8900002	9,04299989	9,04299989	sp|P69005|ACTD_STRPU	Actin, cytoskeletal 2B OS=Strongylocentrotus purpuratus GN=	STRPU	2
27	0	4	14,8900002	9,04299989	9,04299989	sp|P69004|ACT2_STRFN	Actin-15B OS=Strongylocentrotus franciscanus PE=2 SV=1	STRFN	2
27	0	4	14,8900002	9,04299989	9,04299989	sp|P69003|ACT1_HELTB	Actin CyI, cytoplasmic OS=Heliocidaris tuberculata PE=3 SV=1	HELTB	2
27	0	4	14,8900002	9,04299989	9,04299989	sp|P69002|ACT1_HELER	Actin CyI, cytoplasmic OS=Heliocidaris erythrogramma PE=3 S\	HELER	2
27	0	4	14,8900002	9,04299989	9,04299989	sp|P68556|ACT1_DIPDE	Actin-1/4 OS=Diphyllobothrium dendriticum GN=ACT1 PE=2 S	DIPDE	2
27	0	4	14,8900002	9,04299989	9,04299989	sp|P68555|ACT_TAESO	Actin OS=Taenia solium GN=ACT1 PE=3 SV=1	TAESO	2
27	0	4	14,9299994	9,0669997	9,0669997	sp|P53689|ACT_PHARH	Actin OS=Phaffia rhodozyma PE=3 SV=1	PHARH	2
27	0	4	14,8900002	9,04299989	9,04299989	sp|P53501|ACT3_DROME	Actin-57B OS=Drosophila melanogaster GN=Act57B PE=1 SV=1	DROME	2
27	0	4	14,8900002	9,04299989	9,04299989	sp|P53474|ACTE_STRPU	Actin, cytoskeletal 3A OS=Strongylocentrotus purpuratus GN=	STRPU	2
27	0	4	14,8900002	9,04299989	9,04299989	sp|P53473|ACTB_STRPU	Actin, cytoskeletal 1B OS=Strongylocentrotus purpuratus GN=	STRPU	2
27	0	4	14,8900002	9,04299989	9,04299989	sp|P53472|ACTA_STRPU	Actin, cytoskeletal 1A OS=Strongylocentrotus purpuratus GN=	STRPU	2
27	0	4	14,8900002	9,04299989	9,04299989	sp|P53466|ACT2_LYTPI	Actin, cytoskeletal 2 OS=Lytechinus pictus PE=2 SV=1	LYTPI	2
27	0	4	14,8900002	9,04299989	9,04299989	sp|P53465|ACT1_LYTPI	Actin, cytoskeletal 1 OS=Lytechinus pictus PE=2 SV=1	LYTPI	2
27	0	4	14,8900002	9,04299989	9,04299989	sp|P53456|ACT2_DIPDE	Actin-2 OS=Diphyllobothrium dendriticum GN=ACT2 PE=2 SV=	DIPDE	2
27	0	4	14,9299994	9,0669997	9,0669997	sp|P53455|ACT_AJECG	Actin OS=Ajellomyces capsulatus (strain G186AR / H82 / ATCC	AJECG	2
27	0	4	14,8900002	9,04299989	9,04299989	sp|P49871|ACT_MANSE	Actin, muscle OS=Manduca sexta PE=2 SV=1	MANSE	2
27	0	4	14,8900002	9,04299989	9,04299989	sp|P49128|ACT1_AEDAE	Actin-1 OS=Aedes aegypti GN=ACT-1 PE=2 SV=2	AEDAE	2
27	0	4	14,8900002	9,04299989	9,04299989	sp|P45886|ACT3_BACDO	Actin-3, muscle-specific OS=Bactrocera dorsalis PE=2 SV=1	BACDO	2
27	0	4	14,8900002	9,04299989	9,04299989	sp|P45885|ACT2_BACDO	Actin-2, muscle-specific OS=Bactrocera dorsalis PE=2 SV=1	BACDO	2
27	0	4	14,8900002	9,04299989	9,04299989	sp|P41341|ACTY_LIMPO	Actin-11 OS=Limulus polyphemus PE=2 SV=1	LIMPO	2
27	0	4	14,8900002	9,04299989	9,04299989	sp|P41340|ACT3_LIMPO	Actin-3 OS=Limulus polyphemus PE=1 SV=1	LIMPO	2
27	0	4	14,8900002	9,04299989	9,04299989	sp|P41339|ACTA_LIMPO	Actin, acrosomal process isoform OS=Limulus polyphemus PE=	LIMPO	2
27	0	4	14,8900002	9,04299989	9,04299989	sp|P41113|ACT3_PODCA	Actin-3 OS=Podocoryna carnea GN=ACT3 PE=3 SV=1	PODCA	2
27	0	4	14,8900002	9,04299989	9,04299989	sp|P41112|ACT1_PODCA	Actin-1/2 OS=Podocoryna carnea GN=ACTIA PE=2 SV=1	PODCA	2
27	0	4	14,9299994	9,0669997	9,0669997	sp|P20359|ACTG_EMENI	Actin, gamma OS=Emericella nidulans (strain FGSC A4 / ATCC	EMENI	2
27	0	4	14,8900002	9,04299989	9,04299989	sp|P18603|ACT4_ARTSX	Actin, clone 403 OS=Artemia sp. PE=2 SV=1	ARTSX	2
27	0	4	14,8900002	9,04299989	9,04299989	sp|P18499|ACTF_STRPU	Actin, cytoskeletal 3B OS=Strongylocentrotus purpuratus GN=	STRPU	2
27	0	4	14,8900002	9,04299989	9,04299989	sp|P17126|ACT_HYDVU	Actin, non-muscle 6.2 OS=Hydra vulgaris PE=3 SV=1	HYDVU	2
27	0	4	14,8900002	9,04299989	9,04299989	sp|P12717|ACTM_PISOC	Actin, muscle OS=Pisaster ochraceus PE=3 SV=1	PISOC	2
27	0	4	14,8900002	9,04299989	9,04299989	sp|P12716|ACTC_PISOC	Actin, cytoplasmic OS=Pisaster ochraceus PE=3 SV=1	PISOC	2
27	0	4	14,9700001	9,09100026	9,09100026	sp|P12431|ACTM_STRPU	Actin, muscle OS=Strongylocentrotus purpuratus PE=3 SV=1	STRPU	2
27	0	4	14,8900002	9,04299989	9,04299989	sp|P10990|ACT1_STRFN	Actin-15A OS=Strongylocentrotus franciscanus PE=3 SV=1	STRFN	2
27	0	4	14,8900002	9,04299989	9,04299989	sp|P10987|ACT1_DROME	Actin-5C OS=Drosophila melanogaster GN=Act5C PE=1 SV=4	DROME	2
27	0	4	14,8900002	9,04299989	9,04299989	sp|P10981|ACT5_DROME	Actin-87E OS=Drosophila melanogaster GN=Act87E PE=1 SV=1	DROME	2
27	0	4	14,9299994	9,0669997	9,0669997	sp|P10365|ACT_THELA	Actin OS=Thermomyces lanuginosus PE=3 SV=1	THELA	2
27	0	4	14,8900002	9,04299989	9,04299989	sp|P07836|ACT1_BOMMO	Actin, muscle-type A1 OS=Bombyx mori PE=3 SV=1	BOMMO	2
27	0	4	14,8900002	9,04299989	9,04299989	sp|P07830|ACT1_DICDI	Major actin OS=Dictyostelium discoideum GN=act1 PE=1 SV=2	DICDI	2
27	0	4	14,8900002	9,04299989	9,04299989	sp|P07829|ACT3_DICDI	Actin-3 OS=Dictyostelium discoideum GN=act3 PE=3 SV=3	DICDI	2
27	0	4	14,8900002	9,04299989	9,04299989	sp|P04829|ACT3_BOMMO	Actin, cytoplasmic A3 OS=Bombyx mori PE=3 SV=3	BOMMO	2
27	0	4	14,8900002	9,04299989	9,04299989	sp|P02576|ACTA_PHYPO	Actin, plasmodial isoform OS=Physarum polycephalum GN=AF	PHYPO	2
27	0	4	14,8900002	9,04299989	9,04299989	sp|P02572|ACT2_DROME	Actin-42A OS=Drosophila melanogaster GN=Act42A PE=1 SV=	DROME	2
27	0	4	14,8900002	9,04299989	9,04299989	sp|O18500|ACT2_SACKO	Actin-2 OS=Saccoglossus kowalevskii PE=2 SV=1	SACKO	2
27	0	4	14,8900002	9,04299989	9,04299989	sp|O17320|ACT_CRAGI	Actin OS=Crassostrea gigas PE=2 SV=1	CRAGI	2
27	0	4	14,9299994	9,0669997	9,0669997	sp|O13419|ACT_BOTFU	Actin OS=Botryotinia fuckeliana GN=actA PE=3 SV=1	BOTFU	2
27	0	4	16,9200003	12,7800003	12,7800003	sp|Q92193|ACT_CRAVI	Actin (Fragment) OS=Crassostrea virginica PE=2 SV=1	CRAVI	2
27	0	4	12,5699997	9,49700028	9,49700028	sp|Q55EU6|ACT23_DICDI	Putative actin-23 OS=Dictyostelium discoideum GN=act23 PE=	DICDI	2
27	0	4	11,8699998	8,97099972	8,97099972	sp|Q00214|ACTM_STYPL	Actin, muscle OS=Styela plicata PE=3 SV=1	STYPL	2
27	0	4	11,97	9,04299989	9,04299989	sp|P45887|ACT5_BACDO	Actin-5, muscle-specific OS=Bactrocera dorsalis PE=2 SV=1	BACDO	2
27	0	4	11,97	9,04299989	9,04299989	sp|P02574|ACT4_DROME	Actin, larval muscle OS=Drosophila melanogaster GN=Act79B	DROME	2
27	0	4	11,97	9,04299989	9,04299989	sp|O18499|ACT1_SACKO	Actin-1 OS=Saccoglossus kowalevskii PE=2 SV=1	SACKO	2
28	4,32	4,32	32,2299987	20,2500001	14,8800001	sp|P31944|CASPE_HUMAN	Caspase-14 OS=Homo sapiens GN=CASP14 PE=1 SV=2	HUMAN	2
29	4,18	4,18	30,250001	25,9299994	19,1400006	sp|Q5VSP4|LC1L1_HUMAN	Putative lipocalin 1-like protein 1 OS=Homo sapiens GN=LCN1	HUMAN	2
29	0	4,18	27,8400004	23,8600001	17,6100001	sp|P31025|LCN1_HUMAN	Lipocalin-1 OS=Homo sapiens GN=LCN1 PE=1 SV=1	HUMAN	2
30	4	4	14,4899994	14,4899994	14,4899994	sp|Q9GZZ8|LACRT_HUMAN	Extracellular glycoprotein lacritin OS=Homo sapiens GN=LACR	HUMAN	2
31	3,83	3,83	6,42699972	4,34800014	4,34800014	sp|Q7YR44|CDSN_PANTR	Corneodesmosin OS=Pan troglodytes GN=CDSN PE=2 SV=1	PANTR	2
31	0	3,83	6,42699972	4,34800014	4,34800014	sp|Q15517|CDSN_HUMAN	Corneodesmosin OS=Homo sapiens GN=CDSN PE=1 SV=3	HUMAN	2
32	3,82	4,2	9,22600031	8,072	5,60100004	sp|P02769|ALBU_BOVIN	Serum albumin OS=Bos taurus GN=ALB PE=1 SV=4	BOVIN	2
33	3,18	3,18	23,2700005	16,9799998	16,9799998	sp|P01591|IGJ_HUMAN	Immunoglobulin J chain OS=Homo sapiens GN=JCHAIN PE=1 S	HUMAN	2
34	2,67	2,67	44,5499986	32,6700002	32,6700002	sp|P31151|S10A7_HUMAN	Protein S100-A7 OS=Homo sapiens GN=S100A7 PE=1 SV=4	HUMAN	2
36	2,5	2,5	50,940001	33,0199987	33,0199987	sp|P0CG06|LAC3_HUMAN	Ig lambda-3 chain C regions OS=Homo sapiens GN=IGLC3 PE=1	HUMAN	2
36	0	2,5	50,940001	33,0199987	33,0199987	sp|P0CG05|LAC2_HUMAN	Ig lambda-2 chain C regions OS=Homo sapiens GN=IGLC2 PE=1	HUMAN	2
36	0	2,48	20,0900003	16,3599998	16,3599998	sp|B9A064|IGLL5_HUMAN	Immunoglobulin lambda-like polypeptide 5 OS=Homo sapiens	HUMAN	2
36	0	2,48	33,0199987	33,0199987	33,0199987	sp|P0CG04|LAC1_HUMAN	Ig lambda-1 chain C regions OS=Homo sapiens GN=IGLC1 PE=1	HUMAN	2
48	1,84	1,84	6,02600016	1,84599999	1,08000003	sp|P15924|DESP_HUMAN	Desmoplakin OS=Homo sapiens GN=DSP PE=1 SV=3	HUMAN	2

48	0	1,84	5,93100004	1,83799993	1,07500004	sp|E9Q557|DESP_MOUSE	Desmoplakin OS=Mus musculus GN=Dsp PE=1 SV=1	MOUSE	2
27	0	2,41	18,5699999	12,7299994	4,77499999	sp|P53457|ACT3_DIPDE	Actin-3 OS=Diphyllobothrium dendriticum GN=ACT3 PE=2 SV=	DIPDE	1
27	0	2,41	18,1299999	12,2699998	4,26700003	sp|P43239|ACT1_PNECA	Actin-1 OS=Pneumocystis carinii PE=2 SV=1	PNECA	1
27	0	2,41	18,6199993	12,7700001	4,7869999	sp|P30163|ACT2_ONCVO	Actin-2 OS=Onchocerca volvulus GN=act-2b PE=3 SV=1	ONCVO	1
27	0	2,41	18,6199993	12,7700001	4,7869999	sp|P30162|ACT1_ONCVO	Actin-1 OS=Onchocerca volvulus GN=act-1a PE=3 SV=1	ONCVO	1
27	0	2,41	18,0899993	12,2299999	4,25500013	sp|P17304|ACTM_APLCA	Actin, muscle OS=Aplysia californica PE=2 SV=1	APLCA	1
27	0	2,41	18,6199993	12,7700001	4,7869999	sp|P10984|ACT2_CAEEL	Actin-2 OS=Caenorhabditis elegans GN=act-2 PE=3 SV=3	CAEEL	1
27	0	2,41	18,5699999	12,7299994	4,77499999	sp|O65315|ACT_COLSC	Actin OS=Coleochaete scutata PE=2 SV=1	COLSC	1
27	0	2,41	15,6499997	12,7299994	4,77499999	sp|P53498|ACT_CHLRE	Actin OS=Chlamydomonas reinhardtii PE=2 SV=1	CHLRE	1
27	0	2,41	15,6499997	12,7299994	4,77499999	sp|P20904|ACT_VOLCA	Actin OS=Volvox carteri PE=3 SV=1	VOLCA	1
31	0	2,01	5,43100014	3,37099992	3,37099992	sp|Q5TM45|CDSN_MACMU	Corneodesmosin OS=Macaca mulatta GN=CDSN PE=3 SV=1	MACMU	1
32	0	1,7	3,46000008	3,46000008	3,46000008	sp|P14639|ALBU_SHEEP	Serum albumin OS=Ovis aries GN=ALB PE=1 SV=1	SHEEP	1
34	0	1,54	12,8700003	12,8700003	12,8700003	sp|Q86SG5|S1A7A_HUMAN	Protein S100-A7A OS=Homo sapiens GN=S100A7A PE=1 SV=3	HUMAN	1
35	2,58	2,58	30,6100011	30,6100011	18,3699995	sp|P01040|CYTA_HUMAN	Cystatin-A OS=Homo sapiens GN=CSTA PE=1 SV=1	HUMAN	1
36	0	1,75	32,0800006	14,1499996	14,1499996	sp|P0CF74|LAC6_HUMAN	Ig lambda-6 chain C region OS=Homo sapiens GN=IGLC6 PE=4	HUMAN	1
36	0	1,75	32,0800006	14,1499996	14,1499996	sp|A0M8Q6|LAC7_HUMAN	Ig lambda-7 chain C region OS=Homo sapiens GN=IGLC7 PE=4	HUMAN	1
38	2,04	2,04	12,0499998	12,0499998	2,56399997	sp|P29508|SPB3_HUMAN	Serpin B3 OS=Homo sapiens GN=SERPINB3 PE=1 SV=2	HUMAN	1
41	2,01	2,01	8,14799964	3,45700011	3,45700011	sp|Q96P63|SPB12_HUMAN	Serpin B12 OS=Homo sapiens GN=SERPINB12 PE=1 SV=1	HUMAN	1
42	2	2,47	6,51699975	4,68400009	3,25900018	sp|Q6IFX1|K1C24_RAT	Keratin, type I cytoskeletal 24 OS=Rattus norvegicus GN=Krt24	RAT	1
42	0	2,47	6,25	4,49200012	3,125	sp|A1L317|K1C24_MOUSE	Keratin, type I cytoskeletal 24 OS=Mus musculus GN=Krt24 PE	MOUSE	1
43	2	2	37,2799993	19,7500005	19,7500005	sp|Q63429|UBC_RAT	Polyubiquitin-C OS=Rattus norvegicus GN=Ubc PE=1 SV=1	RAT	1
43	0	2	32,7899992	20,9800005	20,9800005	sp|Q8MKD1|UBB_HORSE	Polyubiquitin-B OS=Equus caballus GN=UBB PE=2 SV=3	HORSE	1
43	0	2	32,8900009	21,0500002	21,0500002	sp|Q865C5|UBIQ_CAMDR	Ubiquitin OS=Camelus dromedarius PE=3 SV=2	CAMDR	1
43	0	2	39,6800011	25,4000008	25,4000008	sp|P84589|UBIQ_LUMTE	Ubiquitin (Fragment) OS=Lumbricus terrestris PE=1 SV=2	LUMTE	1
43	0	2	16,0300002	10,2600001	10,2600001	sp|P79781|RS27A_CHICK	Ubiquitin-40S ribosomal protein S27a OS=Gallus gallus GN=RF	CHICK	1
43	0	2	19,5299998	12,5	12,5	sp|P68205|RL40_OPHHA	Ubiquitin-60S ribosomal protein L40 OS=Ophiophagus hannah	OPHHA	1
43	0	2	16,0300002	10,2600001	10,2600001	sp|P68203|RS27A_SPOFR	Ubiquitin-40S ribosomal protein S27a OS=Spodoptera frugipe	SPOFR	1
43	0	2	16,1300004	10,3200004	10,3200004	sp|P68202|RS27A_PLUXY	Ubiquitin-40S ribosomal protein S27a OS=Plutella xylostella Pl	PLUXY	1
43	0	2	16,0300002	10,2600001	10,2600001	sp|P68200|RS27A_ICTPU	Ubiquitin-40S ribosomal protein S27a OS=Ictalurus punctatus	ICTPU	1
43	0	2	32,8900009	21,0500002	21,0500002	sp|P68197|UBIQ_CERCA	Ubiquitin OS=Ceratitis capitata PE=1 SV=1	CERCA	1
43	0	2	19,5299998	12,5	12,5	sp|P63053|RL40_PIG	Ubiquitin-60S ribosomal protein L40 OS=Sus scrofa GN=UBA5	PIG	1
43	0	2	19,5299998	12,5	12,5	sp|P63052|RL40_FELCA	Ubiquitin-60S ribosomal protein L40 OS=Felis catus GN=UBA5	FELCA	1
43	0	2	19,5299998	12,5	12,5	sp|P63050|RL40_CANLF	Ubiquitin-60S ribosomal protein L40 OS=Canis lupus familiaris	CANLF	1
43	0	2	19,5299998	12,5	12,5	sp|P63048|RL40_BOVIN	Ubiquitin-60S ribosomal protein L40 OS=Bos taurus GN=UBA5	BOVIN	1
43	0	2	16,0300002	10,2600001	10,2600001	sp|P62992|RS27A_BOVIN	Ubiquitin-40S ribosomal protein S27a OS=Bos taurus GN=RPS	BOVIN	1
43	0	2	19,5299998	12,5	12,5	sp|P62987|RL40_HUMAN	Ubiquitin-60S ribosomal protein L40 OS=Homo sapiens GN=U	HUMAN	1
43	0	2	19,5299998	12,5	12,5	sp|P62986|RL40_RAT	Ubiquitin-60S ribosomal protein L40 OS=Rattus norvegicus GN	RAT	1
43	0	2	19,5299998	12,5	12,5	sp|P62984|RL40_MOUSE	Ubiquitin-60S ribosomal protein L40 OS=Mus musculus GN=U	MOUSE	1
43	0	2	16,0300002	10,2600001	10,2600001	sp|P62983|RS27A_MOUSE	Ubiquitin-40S ribosomal protein S27a OS=Mus musculus GN=I	MOUSE	1
43	0	2	16,0300002	10,2600001	10,2600001	sp|P62982|RS27A_RAT	Ubiquitin-40S ribosomal protein S27a OS=Rattus norvegicus G	RAT	1
43	0	2	16,0300002	10,2600001	10,2600001	sp|P62979|RS27A_HUMAN	Ubiquitin-40S ribosomal protein S27a OS=Homo sapiens GN=I	HUMAN	1
43	0	2	16,0300002	10,2600001	10,2600001	sp|P62978|RS27A_CAVPO	Ubiquitin-40S ribosomal protein S27a OS=Cavia porcellus GN=	CAVPO	1
43	0	2	30,399999	19,4499999	19,4499999	sp|P62976|UBIQP_CRIGR	Polyubiquitin OS=Cricetulus griseus PE=2 SV=2	CRIGR	1
43	0	2	32,8900009	21,0500002	21,0500002	sp|P62975|UBIQ_RABIT	Ubiquitin OS=Oryctolagus cuniculus PE=1 SV=1	RABIT	1
43	0	2	35,3300005	19,1599995	19,1599995	sp|P62972|UBIQP_XENLA	Polyubiquitin (Fragment) OS=Xenopus laevis PE=1 SV=2	XENLA	1
43	0	2	16,1300004	10,3200004	10,3200004	sp|P29504|RS27A_MANSE	Ubiquitin-40S ribosomal protein S27a OS=Manduca sexta PE=	MANSE	1
43	0	2	19,5299998	12,5	12,5	sp|P18101|RL40_DROME	Ubiquitin-60S ribosomal protein L40 OS=Drosophila melanoga	DROME	1
43	0	2	16,0300002	10,2600001	10,2600001	sp|P15357|RS27A_DROME	Ubiquitin-40S ribosomal protein S27a OS=Drosophila melanog	DROME	1
43	0	2	32,6099992	20,8700001	20,8700001	sp|P0CH28|UBC_BOVIN	Polyubiquitin-C OS=Bos taurus GN=UBC PE=1 SV=1	BOVIN	1
43	0	2	32,7699989	20,9700003	20,9700003	sp|P0CG69|UBIQP_DROME	Polyubiquitin OS=Drosophila melanogaster GN=Ubi-p63E PE=	DROME	1
43	0	2	32,8299999	21,0099995	21,0099995	sp|P0CG68|UBC_PIG	Polyubiquitin-C OS=Sus scrofa GN=UBC PE=2 SV=1	PIG	1
43	0	2	32,7499986	20,9600002	20,9600002	sp|P0CG67|UBB_GORGO	Polyubiquitin-B OS=Gorilla gorilla gorilla GN=UBB PE=3 SV=1	GORGO	1
43	0	2	32,8399986	21,0199997	21,0199997	sp|P0CG66|UBC_GORGO	Polyubiquitin-C OS=Gorilla gorilla gorilla GN=UBC PE=3 SV=1	GORGO	1
43	0	2	32,7499986	20,9600002	20,9600002	sp|P0CG65|UBB_PANTR	Polyubiquitin-B OS=Pan troglodytes GN=UBB PE=3 SV=1	PANTR	1
43	0	2	32,8500003	21,0199997	21,0199997	sp|P0CG64|UBC_PANTR	Polyubiquitin-C OS=Pan troglodytes GN=UBC PE=3 SV=1	PANTR	1
43	0	2	32,7899992	20,9800005	20,9800005	sp|P0CG62|UBB_CHICK	Polyubiquitin-B OS=Gallus gallus GN=UBB PE=2 SV=1	CHICK	1
43	0	2	32,8500003	21,0199997	21,0199997	sp|P0CG61|UBC_PONPY	Polyubiquitin-C OS=Pongo pygmaeus GN=UBC PE=3 SV=1	PONPY	1
43	0	2	32,7499986	20,9600002	20,9600002	sp|P0CG60|UBB_PONPY	Polyubiquitin-B OS=Pongo pygmaeus GN=UBB PE=3 SV=1	PONPY	1
43	0	2	32,7899992	20,9800005	20,9800005	sp|P0CG55|UBB_SHEEP	Polyubiquitin-B OS=Ovis aries GN=UBB PE=2 SV=1	SHEEP	1
43	0	2	32,1500003	20,5799997	20,5799997	sp|P0CG54|UBB_CAVPO	Polyubiquitin-B OS=Cavia porcellus GN=UBB PE=2 SV=1	CAVPO	1
43	0	2	32,7899992	20,9800005	20,9800005	sp|P0CG53|UBB_BOVIN	Polyubiquitin-B OS=Bos taurus GN=UBB PE=1 SV=1	BOVIN	1
43	0	2	32,7899992	20,9800005	20,9800005	sp|P0CG51|UBB_RAT	Polyubiquitin-B OS=Rattus norvegicus GN=Ubb PE=1 SV=1	RAT	1
43	0	2	30,6499988	19,6199998	19,6199998	sp|P0CG50|UBC_MOUSE	Polyubiquitin-C OS=Mus musculus GN=Ubc PE=1 SV=2	MOUSE	1
43	0	2	32,7899992	20,9800005	20,9800005	sp|P0CG49|UBB_MOUSE	Polyubiquitin-B OS=Mus musculus GN=Ubb PE=2 SV=1	MOUSE	1
43	0	2	32,8500003	21,0199997	21,0199997	sp|P0CG48|UBC_HUMAN	Polyubiquitin-C OS=Homo sapiens GN=UBC PE=1 SV=3	HUMAN	1
43	0	2	32,7499986	20,9600002	20,9600002	sp|P0CG47|UBB_HUMAN	Polyubiquitin-B OS=Homo sapiens GN=UBB PE=1 SV=1	HUMAN	1
43	0	2	19,5299998	12,5	12,5	sp|P0C276|RL40_SHEEP	Ubiquitin-60S ribosomal protein L40 OS=Ovis aries GN=UBA52	SHEEP	1
43	0	2	19,5299998	12,5	12,5	sp|P0C275|RL40_PONPY	Ubiquitin-60S ribosomal protein L40 OS=Pongo pygmaeus GN	PONPY	1
43	0	2	19,5299998	12,5	12,5	sp|P0C273|RL40_MACFA	Ubiquitin-60S ribosomal protein L40 OS=Macaca fascicularis G	MACFA	1
44	2	2	1,798	0,46009999	0,46009999	sp|Q5D862|FILA2_HUMAN	Filaggrin-2 OS=Homo sapiens GN=FLG2 PE=1 SV=1	HUMAN	1
45	2	2	6,20499998	2,59700008	2,59700008	sp|Q08188|TGM3_HUMAN	Protein-glutamine gamma-glutamyltransferase E OS=Homo sa	HUMAN	1
46	2	2	15,9700006	15,9700006	15,9700006	sp|P01777|HV316_HUMAN	Ig heavy chain V-III region TEI OS=Homo sapiens PE=1 SV=1	HUMAN	1
46	0	2	15,8299997	15,8299997	15,8299997	sp|P01766|HV305_HUMAN	Ig heavy chain V-III region BRO OS=Homo sapiens PE=1 SV=1	HUMAN	1
46	0	2	16,2400007	16,2400007	16,2400007	sp|P01764|HV303_HUMAN	Ig heavy chain V-III region 23 OS=Homo sapiens GN=IGHV3-23	HUMAN	1
47	2	2	7,80100003	7,80100003	7,80100003	sp|P01037|CYTN_HUMAN	Cystatin-SN OS=Homo sapiens GN=CST1 PE=1 SV=3	HUMAN	1
47	0	2	7,80100003	7,80100003	7,80100003	sp|P01036|CYTS_HUMAN	Cystatin-S OS=Homo sapiens GN=CST4 PE=1 SV=3	HUMAN	1
49	1,65	1,65	11,3600001	3,78599986	3,78599986	sp|P10909|CLUS_HUMAN	Clusterin OS=Homo sapiens GN=CLU PE=1 SV=1	HUMAN	1
50	1,51	1,51	31,7499995	12,7000004	6,87799975	sp|P05090|APOD_HUMAN	Apolipoprotein D OS=Homo sapiens GN=APOD PE=1 SV=1	HUMAN	1
51	1,42	1,42	4,36200015	1,67800002	1,67800002	sp|Q08554|DSC1_HUMAN	Desmocollin-1 OS=Homo sapiens GN=DSC1 PE=1 SV=2	HUMAN	1
52	1,26	1,26	4,29500006	1,47700002	1,47700002	sp|Q8WNW3|PLAK_PIG	Junction plakoglobin OS=Sus scrofa GN=Jup PE=2 SV=1	PIG	1
52	0	1,26	4,29500006	1,47700002	1,47700002	sp|Q8SPJ1|PLAK_BOVIN	Junction plakoglobin OS=Bos taurus GN=JUP PE=2 SV=1	BOVIN	1
52	0	1,26	4,29500006	1,47700002	1,47700002	sp|Q6P0K8|PLAK_RAT	Junction plakoglobin OS=Rattus norvegicus GN=Jup PE=1 SV=	RAT	1
52	0	1,26	4,29500006	1,47700002	1,47700002	sp|Q02257|PLAK_MOUSE	Junction plakoglobin OS=Mus musculus GN=Jup PE=1 SV=3	MOUSE	1
52	0	1,26	4,29500006	1,47700002	1,47700002	sp|P14923|PLAK_HUMAN	Junction plakoglobin OS=Homo sapiens GN=JUP PE=1 SV=3	HUMAN	1
53	1,25	1,26	24,2699996	24,2699996	24,2699996	sp|P55000|SLUR1_HUMAN	Secreted Ly-6/uPAR-related protein 1 OS=Homo sapiens GN=S	HUMAN	1
54	0,98	0,98	11,8000001	2,95000002	2,95000002	sp|Q6TEQ7|ANXA2_CANLF	Annexin A2 OS=Canis lupus familiaris GN=ANXA2 PE=1 SV=1	CANLF	1
54	0	0,98	11,8000001	2,95000002	2,95000002	sp|Q5R5A0|ANXA2_PONAB	Annexin A2 OS=Pongo abelii GN=ANXA2 PE=2 SV=1	PONAB	1
54	0	0,98	11,8000001	2,95000002	2,95000002	sp|Q2Q1M6|ANXA2_CEREL	Annexin A2 OS=Cervus elaphus GN=ANXA2 PE=2 SV=1	CEREL	1
54	0	0,98	11,8000001	2,95000002	2,95000002	sp|Q07936|ANXA2_RAT	Annexin A2 OS=Rattus norvegicus GN=Anxa2 PE=1 SV=2	RAT	1
54	0	0,98	11,8000001	2,95000002	2,95000002	sp|P19620|ANXA2_PIG	Annexin A2 OS=Sus scrofa GN=ANXA2 PE=1 SV=4	PIG	1
54	0	0,98	11,8000001	2,95000002	2,95000002	sp|P07355|ANXA2_HUMAN	Annexin A2 OS=Homo sapiens GN=ANXA2 PE=1 SV=2	HUMAN	1
54	0	0,98	11,8000001	2,95000002	2,95000002	sp|P04272|ANXA2_BOVIN	Annexin A2 OS=Bos taurus GN=ANXA2 PE=1 SV=2	BOVIN	1
54	0	0,98	11,8000001	2,95000002	2,95000002	sp|A2SW69|ANXA2_SHEEP	Annexin A2 OS=Ovis aries GN=ANXA2 PE=1 SV=1	SHEEP	1
54	0	0,93	7,6700002	2,95000002	2,95000002	sp|A6NMY6|AXA2L_HUMAN	Putative annexin A2-like protein OS=Homo sapiens GN=ANXA	HUMAN	1
55	0,92	0,92	3,48399989	3,48399989	3,48399989	sp|A6VL73|Y342_ACTSZ	UPF0761 membrane protein Asuc_0342 OS=Actinobacillus suc	ACTSZ	1
56	0,86	0,86	100	100	100	sp|P84735|PS19_PINST	Putative LRR disease resistance protein/transmembrane recep	PINST	1
56	0	0,86	100	100	100	sp|P84732|PS16_PINST	Putative LRR disease resistance protein/transmembrane recep	PINST	1

<table>
<tr><td>56</td><td>0</td><td>0,86</td><td>100</td><td>100</td><td>100</td><td>sp|P84720|PS4_PINST</td><td>Putative LRR disease resistance protein/transmembrane recep</td><td>PINST</td><td>1</td></tr>
<tr><td>57</td><td>0,78</td><td>0,78</td><td>2,42599994</td><td>2,42599994</td><td>2,42599994</td><td>sp|Q0CQH1|NOP58_ASPTN</td><td>Nucleolar protein 58 OS=Aspergillus terreus (strain NIH 2624 /</td><td>ASPTN</td><td>1</td></tr>
<tr><td>58</td><td>0,76</td><td>0,76</td><td>0,87050004</td><td>0,87050004</td><td>0,87050004</td><td>sp|Q05884|AMY_STRLI</td><td>Alpha-amylase OS=Streptomyces lividans GN=amy PE=1 SV=1</td><td>STRLI</td><td>1</td></tr>
<tr><td>59</td><td>0,67</td><td>0,67</td><td>3,63599993</td><td>3,63599993</td><td>3,63599993</td><td>sp|P01857|IGHG1_HUMAN</td><td>Ig gamma-1 chain C region OS=Homo sapiens GN=IGHG1 PE=1</td><td>HUMAN</td><td>1</td></tr>
<tr><td>60</td><td>0,65</td><td>0,65</td><td>7,76700005</td><td>3,15500014</td><td>3,15500014</td><td>sp|P07339|CATD_HUMAN</td><td>Cathepsin D OS=Homo sapiens GN=CTSD PE=1 SV=1</td><td>HUMAN</td><td>1</td></tr>
<tr><td>61</td><td>0,58</td><td>0,58</td><td>20,2099994</td><td>5,85100017</td><td>5,85100017</td><td>sp|O75223|GGCT_HUMAN</td><td>Gamma-glutamylcyclotransferase OS=Homo sapiens GN=GGC</td><td>HUMAN</td><td>1</td></tr>
<tr><td>62</td><td>0,5</td><td>0,5</td><td>0,96420003</td><td>0,96420003</td><td>0,96420003</td><td>RRRRRsp|Q8Z303|KATG_SALTI</td><td>REVERSED Catalase-peroxidase OS=Salmonella typhi GN=katG</td><td>SALTI</td><td>1</td></tr>
<tr><td>62</td><td>0</td><td>0,5</td><td>0,96420003</td><td>0,96420003</td><td>0,96420003</td><td>RRRRRsp|Q5PK60|KATG_SALPA</td><td>REVERSED Catalase-peroxidase OS=Salmonella paratyphi A (st</td><td>SALPA</td><td>1</td></tr>
<tr><td>62</td><td>0</td><td>0,5</td><td>0,96420003</td><td>0,96420003</td><td>0,96420003</td><td>RRRRRsp|Q57HA8|KATG_SALCH</td><td>REVERSED Catalase-peroxidase OS=Salmonella choleraesuis (s</td><td>SALCH</td><td>1</td></tr>
<tr><td>62</td><td>0</td><td>0,5</td><td>1,97199993</td><td>1,97199993</td><td>1,97199993</td><td>RRRRRsp|P59487|AROB_BUCBP</td><td>REVERSED 3-dehydroquinate synthase OS=Buchnera aphidico</td><td>BUCBP</td><td>1</td></tr>
<tr><td>62</td><td>0</td><td>0,5</td><td>0,96420003</td><td>0,96420003</td><td>0,96420003</td><td>RRRRRsp|P17750|KATG_SALTY</td><td>REVERSED Catalase-peroxidase OS=Salmonella typhimurium (</td><td>SALTY</td><td>1</td></tr>
<tr><td>62</td><td>0</td><td>0,5</td><td>0,96420003</td><td>0,96420003</td><td>0,96420003</td><td>RRRRRsp|B5BJM5|KATG_SALPK</td><td>REVERSED Catalase-peroxidase OS=Salmonella paratyphi A (st</td><td>SALPK</td><td>1</td></tr>
<tr><td>62</td><td>0</td><td>0,5</td><td>0,96420003</td><td>0,96420003</td><td>0,96420003</td><td>RRRRRsp|B4TQG7|KATG_SALSV</td><td>REVERSED Catalase-peroxidase OS=Salmonella schwarzengrur</td><td>SALSV</td><td>1</td></tr>
<tr><td>62</td><td>0</td><td>0,5</td><td>0,96420003</td><td>0,96420003</td><td>0,96420003</td><td>RRRRRsp|B4TCP6|KATG_SALHS</td><td>REVERSED Catalase-peroxidase OS=Salmonella heidelberg (str</td><td>SALHS</td><td>1</td></tr>
<tr><td>62</td><td>0</td><td>0,5</td><td>0,96420003</td><td>0,96420003</td><td>0,96420003</td><td>RRRRRsp|B4T0V5|KATG_SALNS</td><td>REVERSED Catalase-peroxidase OS=Salmonella newport (strai</td><td>SALNS</td><td>1</td></tr>
<tr><td>62</td><td>0</td><td>0,5</td><td>0,96420003</td><td>0,96420003</td><td>0,96420003</td><td>RRRRRsp|A9N0F0|KATG_SALPB</td><td>REVERSED Catalase-peroxidase OS=Salmonella paratyphi B (st</td><td>SALPB</td><td>1</td></tr>
<tr><td>62</td><td>0</td><td>0,5</td><td>0,96420003</td><td>0,96420003</td><td>0,96420003</td><td>RRRRRsp|A9MI19|KATG_SALAR</td><td>REVERSED Catalase-peroxidase OS=Salmonella arizonae (strai</td><td>SALAR</td><td>1</td></tr>
<tr><td>62</td><td>0</td><td>0,5</td><td>0,96420003</td><td>0,96420003</td><td>0,96420003</td><td>RRRRRsp|A8AKX8|KATG_CITK8</td><td>REVERSED Catalase-peroxidase OS=Citrobacter koseri (strain A</td><td>CITK8</td><td>1</td></tr>
<tr><td>62</td><td>0</td><td>0,5</td><td>0,96549997</td><td>0,96549997</td><td>0,96549997</td><td>RRRRRsp|A6T9H9|KATG_KLEP7</td><td>REVERSED Catalase-peroxidase OS=Klebsiella pneumoniae sul</td><td>KLEP7</td><td>1</td></tr>
<tr><td>62</td><td>0</td><td>0,5</td><td>0,96420003</td><td>0,96420003</td><td>0,96420003</td><td>RRRRRsp|A4WG57|KATG_ENT38</td><td>REVERSED Catalase-peroxidase OS=Enterobacter sp. (strain 63</td><td>ENT38</td><td>1</td></tr>
<tr><td>66</td><td>0,22</td><td>0,22</td><td>1,76599994</td><td>1,76599994</td><td>1,76599994</td><td>sp|P21910|LAML2_XENLA</td><td>Lamin-L(II) OS=Xenopus laevis PE=2 SV=1</td><td>XENLA</td><td>1</td></tr>
<tr><td>63</td><td>0,27</td><td>0,27</td><td>6,96199983</td><td>6,96199983</td><td>0</td><td>sp|Q9UGM3|DMBT1_HUMAN</td><td>Deleted in malignant brain tumors 1 protein OS=Homo sapien</td><td>HUMAN</td><td>0</td></tr>
<tr><td>64</td><td>0,25</td><td>0,25</td><td>1,04900002</td><td>1,04900002</td><td>0</td><td>RRRRRsp|P48479|NIM1_NEUCR</td><td>REVERSED G2-specific protein kinase nim-1 OS=Neurospora cr</td><td>NEUCR</td><td>0</td></tr>
<tr><td>65</td><td>0,24</td><td>0,24</td><td>4,80000004</td><td>4,80000004</td><td>0</td><td>sp|Q5T750|XP32_HUMAN</td><td>Skin-specific protein 32 OS=Homo sapiens GN=XP32 PE=1 SV=</td><td>HUMAN</td><td>0</td></tr>
<tr><td>67</td><td>0,21</td><td>0,21</td><td>0,93649998</td><td>0,93649998</td><td>0</td><td>sp|Q9LJX4|PUM5_ARATH</td><td>Pumilio homolog 5 OS=Arabidopsis thaliana GN=APUM5 PE=1</td><td>ARATH</td><td>0</td></tr>
<tr><td>68</td><td>0,17</td><td>0,17</td><td>1,17300004</td><td>1,17300004</td><td>0</td><td>sp|Q2KWY3|LEPA_BORA1</td><td>Elongation factor 4 OS=Bordetella avium (strain 197N) GN=lep</td><td>BORA1</td><td>0</td></tr>
<tr><td>68</td><td>0</td><td>0,17</td><td>1,04</td><td>1,04</td><td>0</td><td>RRRRRsp|Q1LKN9|UVRC_CUPMC</td><td>REVERSED UvrABC system protein C OS=Cupriavidus metallidu</td><td>CUPMC</td><td>0</td></tr>
<tr><td>69</td><td>0,16</td><td>0,16</td><td>1,91799998</td><td>1,91799998</td><td>0</td><td>RRRRRsp|Q9SFC7|FB135_ARATH</td><td>REVERSED F-box protein At3g07870 OS=Arabidopsis thaliana</td><td>ARATH</td><td>0</td></tr>
<tr><td>70</td><td>0,15</td><td>0,15</td><td>6,68499991</td><td>6,68499991</td><td>0</td><td>sp|P12763|FETUA_BOVIN</td><td>Alpha-2-HS-glycoprotein OS=Bos taurus GN=AHSG PE=1 SV=2</td><td>BOVIN</td><td>0</td></tr>
<tr><td>71</td><td>0,13</td><td>0,13</td><td>100</td><td>100</td><td>0</td><td>sp|P85958|UP02_PSEMZ</td><td>Unknown protein 2 (Fragment) OS=Pseudotsuga menziesii PE</td><td>PSEMZ</td><td>0</td></tr>
<tr><td>72</td><td>0,12</td><td>0,12</td><td>1,53999999</td><td>1,53999999</td><td>0</td><td>RRRRRsp|Q8IX18|DHX40_HUMA</td><td>REVERSED Probable ATP-dependent RNA helicase DHX40 OS=</td><td>HUMAN</td><td>0</td></tr>
<tr><td>72</td><td>0</td><td>0,12</td><td>1,53999999</td><td>1,53999999</td><td>0</td><td>RRRRRsp|Q6PE54|DHX40_MOUS</td><td>REVERSED Probable ATP-dependent RNA helicase DHX40 OS=</td><td>MOUSE</td><td>0</td></tr>
<tr><td>72</td><td>0</td><td>0,12</td><td>1,53999999</td><td>1,53999999</td><td>0</td><td>RRRRRsp|Q5XI69|DHX40_RAT</td><td>REVERSED Probable ATP-dependent RNA helicase DHX40 OS=</td><td>RAT</td><td>0</td></tr>
<tr><td>72</td><td>0</td><td>0,12</td><td>1,53999999</td><td>1,53999999</td><td>0</td><td>RRRRRsp|Q5R864|DHX40_PONA</td><td>REVERSED Probable ATP-dependent RNA helicase DHX40 OS=</td><td>PONAB</td><td>0</td></tr>
<tr><td>73</td><td>0,11</td><td>0,11</td><td>0,76819998</td><td>0,76819998</td><td>0</td><td>sp|Q8T9W4|ABCB3_DICDI</td><td>ABC transporter B family member 3 OS=Dictyostelium discoid</td><td>DICDI</td><td>0</td></tr>
<tr><td>74</td><td>0,11</td><td>0,11</td><td>12,0899998</td><td>12,0899998</td><td>0</td><td>sp|A6LPJ0|SP5G_CLOB8</td><td>Putative septation protein SpoVG OS=Clostridium beijerinckii</td><td>CLOB8</td><td>0</td></tr>
<tr><td>75</td><td>0,11</td><td>0,11</td><td>10,2799997</td><td>10,2799997</td><td>0</td><td>RRRRRsp|B9K6M6|Y433_THENN</td><td>REVERSED UPF0102 protein CTN_0433 OS=Thermotoga neapo</td><td>THENN</td><td>0</td></tr>
<tr><td>76</td><td>0,1</td><td>0,1</td><td>2,38099992</td><td>2,38099992</td><td>0</td><td>sp|Q7V121|LEU1_PROMP</td><td>2-isopropylmalate synthase OS=Prochlorococcus marinus sub</td><td>PROMP</td><td>0</td></tr>
<tr><td>76</td><td>0</td><td>0,1</td><td>2,38099992</td><td>2,38099992</td><td>0</td><td>sp|A2BX52|LEU1_PROM5</td><td>2-isopropylmalate synthase OS=Prochlorococcus marinus (stra</td><td>PROM5</td><td>0</td></tr>
<tr><td>77</td><td>0,08</td><td>0,08</td><td>7,01799989</td><td>7,01799989</td><td>0</td><td>sp|Q03QY4|ATPF_LACBA</td><td>ATP synthase subunit b OS=Lactobacillus brevis (strain ATCC 3</td><td>LACBA</td><td>0</td></tr>
<tr><td>78</td><td>0,08</td><td>0,08</td><td>0,50090002</td><td>0,50090002</td><td>0</td><td>sp|O77384|LRR4_PLAF7</td><td>Protein PFC0760c OS=Plasmodium falciparum (isolate 3D7) GI</td><td>PLAF7</td><td>0</td></tr>
<tr><td>79</td><td>0,07</td><td>0,07</td><td>1,77500006</td><td>1,33100003</td><td>0</td><td>sp|Q6C186|CLF1_YARLI</td><td>Pre-mRNA-splicing factor CLF1 OS=Yarrowia lipolytica (strain C</td><td>YARLI</td><td>0</td></tr>
<tr><td>80</td><td>0,05</td><td>0,05</td><td>0,5988</td><td>0,5988</td><td>0</td><td>sp|Q9LZW9|RTOR2_ARATH</td><td>Regulatory-associated protein of TOR 2 OS=Arabidopsis thalia</td><td>ARATH</td><td>0</td></tr>
<tr><td>81</td><td>0,05</td><td>0,05</td><td>0,24550001</td><td>0,24550001</td><td>0</td><td>sp|Q756G2|TOM1_ASHGO</td><td>Probable E3 ubiquitin-protein ligase TOM1 OS=Ashbya gossyp</td><td>ASHGO</td><td>0</td></tr>
<tr><td>82</td><td>0,05</td><td>0,05</td><td>0,60840002</td><td>0,60840002</td><td>0</td><td>RRRRRsp|Q9NRP7|STK36_HUMA</td><td>REVERSED Serine/threonine-protein kinase 36 OS=Homo sapi</td><td>HUMAN</td><td>0</td></tr>
<tr><td>82</td><td>0</td><td>0,05</td><td>0,6079</td><td>0,6079</td><td>0</td><td>RRRRRsp|Q69ZM6|STK36_MOUS</td><td>REVERSED Serine/threonine-protein kinase 36 OS=Mus muscu</td><td>MOUSE</td><td>0</td></tr>
<tr><td>83</td><td>0,05</td><td>0,05</td><td>2,00500004</td><td>2,00500004</td><td>0</td><td>RRRRRsp|Q21DC2|RLMN_RHOPB</td><td>REVERSED Dual-specificity RNA methyltransferase RlmN OS=R</td><td>RHOPB</td><td>0</td></tr>
<tr><td>84</td><td>0,05</td><td>0,05</td><td>3,66199985</td><td>3,66199985</td><td>0</td><td>RRRRRsp|Q04852|T2C1_CITFR</td><td>REVERSED Type-2 restriction enzyme CfrBI OS=Citrobacter fre</td><td>CITFR</td><td>0</td></tr>
</table>

N	CSV_1	Total	%Cov	%Cov(50)	%Cov(95)	Accession	Name	Species	Peptides(95%)
1	74,54	74,54	64,60000277	61,01999879	54,97000217	sp|P04264|K2C1_HUMAN	Keratin, type II cytoskeletal 1 OS=Homo sapiens GN=KRT1 PE=1 SV=	HUMAN	51
2	58,75	58,75	55,98999858	55,98999858	54,44999933	sp|P13645|K1C10_HUMAN	Keratin, type I cytoskeletal 10 OS=Homo sapiens GN=KRT10 PE=1 SV	HUMAN	37
3	48,86	56,87	62,27999926	56,33999705	53,99000049	sp|P35908|K22E_HUMAN	Keratin, type II cytoskeletal 2 epidermal OS=Homo sapiens GN=KRT.	HUMAN	32
4	47,53	47,53	65,96999764	64,37000036	55,22000194	sp|P35527|K1C9_HUMAN	Keratin, type I cytoskeletal 9 OS=Homo sapiens GN=KRT9 PE=1 SV=:	HUMAN	28
5	30,77	35,39	55,39000034	52,21999884	49,47000146	sp|P08779|K1C16_HUMAN	Keratin, type I cytoskeletal 16 OS=Homo sapiens GN=KRT16 PE=1 SV	HUMAN	19
6	17,15	34,87	37,05999851	32,44999945	30,66999912	sp|P02538|K2C6A_HUMAN	Keratin, type II cytoskeletal 6A OS=Homo sapiens GN=KRT6A PE=1 S	HUMAN	18
6	0	32,87	34,92999971	30,32000065	28,549999	sp|P48668|K2C6C_HUMAN	Keratin, type II cytoskeletal 6C OS=Homo sapiens GN=KRT6C PE=1 S	HUMAN	17
8	10,23	28,09	49,79000092	41,10000134	34,11000073	sp|P02533|K1C14_HUMAN	Keratin, type I cytoskeletal 14 OS=Homo sapiens GN=KRT14 PE=1 SV	HUMAN	16
10	9,79	25,1	32,37000108	22,54000008	20,50999999	sp|P13647|K2C5_HUMAN	Keratin, type II cytoskeletal 5 OS=Homo sapiens GN=KRT5 PE=1 SV=	HUMAN	13
20	1,66	21,47	19,3900004	17,87	17,67999977	sp|P06394|K1C10_BOVIN	Keratin, type I cytoskeletal 10 OS=Bos taurus GN=KRT10 PE=3 SV=1	BOVIN	13
7	14,1	14,1	43,29000115	38,10000122	38,10000122	sp|P00761|TRYP_PIG	Trypsin OS=Sus scrofa PE=1 SV=1	PIG	12
10	0	23,1	28,0400008	19,9300006	17,91000068	sp|A5A6M8|K2C5_PANTR	Keratin, type II cytoskeletal 5 OS=Pan troglodytes GN=KRT5 PE=2 SV	PANTR	12
19	1,8	11,4	13,28999996	11,36000007	11,36000007	sp|Q6IFZ6|K2C1B_MOUSE	Keratin, type II cytoskeletal 1b OS=Mus musculus GN=Krt77 PE=1 SV	MOUSE	10
19	0	9,63	16,9599995	12,52000034	12,52000034	sp|Q6IG01|K2C1B_RAT	Keratin, type II cytoskeletal 1b OS=Rattus norvegicus GN=Krt77 PE=:	RAT	10
12	3,59	11,57	28,70000005	17,82000065	17,58999974	sp|Q04695|K1C17_HUMAN	Keratin, type I cytoskeletal 17 OS=Homo sapiens GN=KRT17 PE=1 SV	HUMAN	7
12	0	11,57	28,70000005	17,82000065	17,58999974	sp|A5A6M0|K1C17_PANTR	Keratin, type I cytoskeletal 17 OS=Pan troglodytes GN=KRT17 PE=2 S	PANTR	7
19	0	8,19	12,98000067	7,43900016	7,43900016	sp|Q7Z794|K2C1B_HUMAN	Keratin, type II cytoskeletal 1b OS=Homo sapiens GN=KRT77 PE=2 S	HUMAN	7
12	0	9,18	22,63000011	15,94000012	13,16000074	sp|Q9QWL7|K1C17_MOUSE	Keratin, type I cytoskeletal 17 OS=Mus musculus GN=Krt17 PE=1 SV	MOUSE	6
12	0	9,18	22,63000011	15,94000012	13,16000074	sp|Q6IFU8|K1C17_RAT	Keratin, type I cytoskeletal 17 OS=Rattus norvegicus GN=Krt17 PE=1	RAT	6
12	0	9,18	19,49999928	13,15000057	12,92999983	sp|A1L595|K1C17_BOVIN	Keratin, type I cytoskeletal 17 OS=Bos taurus GN=KRT17 PE=2 SV=1	BOVIN	6
9	10	10	76,09000206	51,45000219	51,45000219	sp|P15252|REF_HEVBR	Rubber elongation factor protein OS=Hevea brasiliensis PE=1 SV=2	HEVBR	5
22	1,14	9,37	11,72999963	7,308000326	7,308000326	sp|Q8N1N4|K2C78_HUMAN	Keratin, type II cytoskeletal 78 OS=Homo sapiens GN=KRT78 PE=2 S	HUMAN	5
13	3,31	3,31	25,45000017	25,45000017	25,45000017	sp|P81605|DCD_HUMAN	Dermcidin OS=Homo sapiens GN=DCD PE=1 SV=2	HUMAN	3
14	3,24	3,24	25	17,64999926	17,64999926	sp|O82803|SRPP_HEVBR	Small rubber particle protein OS=Hevea brasiliensis GN=SRPP PE=1 !	HEVBR	3
11	4	4	35,47999859	23,65999967	23,65999967	sp|P05109|S10A8_HUMAN	Protein S100-A8 OS=Homo sapiens GN=S100A8 PE=1 SV=1	HUMAN	2
15	2	2,82	4,68400009	4,68400009	4,68400009	sp|Q6IFX1|K1C24_RAT	Keratin, type I cytoskeletal 24 OS=Rattus norvegicus GN=Krt24 PE=3	RAT	2
15	0	2,82	4,492000118	4,492000118	4,492000118	sp|A1L317|K1C24_MOUSE	Keratin, type I cytoskeletal 24 OS=Mus musculus GN=Krt24 PE=2 SV	MOUSE	2
16	2	2	24,56	11,40000001	11,40000001	sp|P06702|S10A9_HUMAN	Protein S100-A9 OS=Homo sapiens GN=S100A9 PE=1 SV=1	HUMAN	1
17	2	2	8,392000198	8,392000198	8,392000198	sp|Q9PU28|LYSC_SCOMX	Lysozyme C OS=Scophthalmus maximus GN=lys PE=2 SV=1	SCOMX	1
17	0	2	8,392000198	8,392000198	8,392000198	sp|Q9DD65|LYSC_PAROL	Lysozyme C OS=Paralichthys olivaceus PE=2 SV=1	PAROL	1
17	0	2	8,107999712	8,107999712	8,107999712	sp|Q6B411|LYSM_BOVIN	Lysozyme C, milk isozyme OS=Bos taurus PE=2 SV=1	BOVIN	1
17	0	2	8,107999712	8,107999712	8,107999712	sp|Q659U5|LYSC_HALGR	Lysozyme C OS=Halichoerus grypus GN=LYZ PE=2 SV=1	HALGR	1
17	0	2	8,107999712	8,107999712	8,107999712	sp|Q659U1|LYSC_PHOVI	Lysozyme C OS=Phoca vitulina GN=LYZ PE=2 SV=1	PHOVI	1
17	0	2	8,107999712	8,107999712	8,107999712	sp|Q659U0|LYSC_LEPWE	Lysozyme C OS=Leptonychotes weddellii GN=LYZ PE=2 SV=1	LEPWE	1
17	0	2	8,107999712	8,107999712	8,107999712	sp|Q05820|LYSC2_RAT	Putative lysozyme C-2 OS=Rattus norvegicus GN=Lyz2 PE=5 SV=1	RAT	1
17	0	2	9,230999649	9,230999649	9,230999649	sp|P85345|LYSC_AMYCA	Lysozyme C OS=Amyda cartilaginea GN=LYZ PE=1 SV=1	AMYCA	1
17	0	2	8,218999952	8,218999952	8,218999952	sp|P85045|LYS_BUFGA	Lysozyme C (Fragment) OS=Bufo gargarizans andrewsi PE=1 SV=1	BUFGA	1
17	0	2	9,230999649	9,230999649	9,230999649	sp|P81709|LYSC2_CANLF	Lysozyme C, spleen isozyme OS=Canis lupus familiaris PE=1 SV=1	CANLF	1
17	0	2	8,107999712	8,107999712	8,107999712	sp|P79847|LYSC_PYGNE	Lysozyme C OS=Pygathrix nemaeus GN=LYZ PE=3 SV=1	PYGNE	1
17	0	2	8,107999712	8,107999712	8,107999712	sp|P79811|LYSC_NASLA	Lysozyme C OS=Nasalis larvatus GN=LYZ PE=3 SV=1	NASLA	1
17	0	2	8,107999712	8,107999712	8,107999712	sp|P79239|LYSC_PONPY	Lysozyme C OS=Pongo pygmaeus GN=LYZ PE=2 SV=1	PONPY	1
17	0	2	8,107999712	8,107999712	8,107999712	sp|P79180|LYSC_HYLLA	Lysozyme C OS=Hylobates lar GN=LYZ PE=2 SV=1	HYLLA	1
17	0	2	8,107999712	8,107999712	8,107999712	sp|P79179|LYSC_GORGO	Lysozyme C OS=Gorilla gorilla gorilla GN=LYZ PE=2 SV=1	GORGO	1
17	0	2	8,107999712	8,107999712	8,107999712	sp|P67980|LYSC_TRAFR	Lysozyme C OS=Trachypithecus francoisi GN=LYZ PE=2 SV=1	TRAFR	1
17	0	2	8,107999712	8,107999712	8,107999712	sp|P67979|LYSC_TRAOB	Lysozyme C OS=Trachypithecus obscurus GN=LYZ PE=2 SV=1	TRAOB	1
17	0	2	8,107999712	8,107999712	8,107999712	sp|P67978|LYSC_TRAVT	Lysozyme C OS=Trachypithecus vetulus GN=LYZ PE=3 SV=1	TRAVT	1
17	0	2	8,107999712	8,107999712	8,107999712	sp|P67977|LYSC_SEMEN	Lysozyme C OS=Semnopithecus entellus GN=LYZ PE=1 SV=1	SEMEN	1
17	0	2	8,392000198	8,392000198	8,392000198	sp|P61944|LYSC_TAKRU	Lysozyme C OS=Takifugu rubripes PE=2 SV=1	TAKRU	1
17	0	2	8,107999712	8,107999712	8,107999712	sp|P61632|LYSC_COLGU	Lysozyme C OS=Colobus guereza GN=LYZ PE=2 SV=1	COLGU	1
17	0	2	8,107999712	8,107999712	8,107999712	sp|P61631|LYSC_COLAN	Lysozyme C OS=Colobus angolensis GN=LYZ PE=3 SV=1	COLAN	1
17	0	2	8,107999712	8,107999712	8,107999712	sp|P61628|LYSC_PANTR	Lysozyme C OS=Pan troglodytes GN=LYZ PE=2 SV=1	PANTR	1
17	0	2	8,107999712	8,107999712	8,107999712	sp|P61627|LYSC_PANPA	Lysozyme C OS=Pan paniscus GN=LYZ PE=3 SV=1	PANPA	1
17	0	2	8,107999712	8,107999712	8,107999712	sp|P61626|LYSC_HUMAN	Lysozyme C OS=Homo sapiens GN=LYZ PE=1 SV=1	HUMAN	1
17	0	2	9,230999649	9,230999649	9,230999649	sp|P37712|LYSC_CAMDR	Lysozyme C OS=Camelus dromedarius GN=LYZ PE=1 SV=1	CAMDR	1
17	0	2	8,107999712	8,107999712	8,107999712	sp|P17897|LYZ1_MOUSE	Lysozyme C-1 OS=Mus musculus GN=Lyz1 PE=1 SV=1	MOUSE	1
17	0	2	9,230999649	9,230999649	9,230999649	sp|P16973|LYSC_RABIT	Lysozyme C OS=Oryctolagus cuniculus GN=LYZ PE=1 SV=1	RABIT	1
17	0	2	8,107999712	8,107999712	8,107999712	sp|P12069|LYSC3_PIG	Lysozyme C-3 OS=Sus scrofa PE=1 SV=2	PIG	1
17	0	2	8,218999952	8,218999952	8,218999952	sp|P12068|LYSC2_PIG	Lysozyme C-2 OS=Sus scrofa PE=1 SV=2	PIG	1
17	0	2	9,375	9,375	9,375	sp|P12067|LYSC1_PIG	Lysozyme C-1 OS=Sus scrofa PE=1 SV=1	PIG	1
17	0	2	8,332999796	8,332999796	8,332999796	sp|P11941|LYSC2_ONCMY	Lysozyme C II OS=Oncorhynchus mykiss PE=1 SV=2	ONCMY	1
17	0	2	8,107999712	8,107999712	8,107999712	sp|P08905|LYZ2_MOUSE	Lysozyme C-2 OS=Mus musculus GN=Lyz2 PE=1 SV=2	MOUSE	1
17	0	2	8,107999712	8,107999712	8,107999712	sp|P00697|LYSC1_RAT	Lysozyme C-1 OS=Rattus norvegicus GN=Lyz1 PE=1 SV=2	RAT	1
18	1,89	1,89	8,696000278	3,415999934	3,415999934	sp|P05089|ARGI1_HUMAN	Arginase-1 OS=Homo sapiens GN=ARG1 PE=1 SV=2	HUMAN	1
21	1,36	1,36	8,218999952	8,218999952	8,218999952	sp|P12273|PIP_HUMAN	Prolactin-inducible protein OS=Homo sapiens GN=PIP PE=1 SV=1	HUMAN	1
21	0	1,36	8,218999952	8,218999952	8,218999952	sp|A0A890|PIP_HYLAG	Prolactin-inducible protein homolog OS=Hylobates agilis GN=PIP PE:	HYLAG	1
21	0	1,36	8,218999952	8,218999952	8,218999952	sp|A0A888|PIP_PONPY	Prolactin-inducible protein homolog OS=Pongo pygmaeus GN=PIP P	PONPY	1
23	0,9	0,9	2,135000005	2,135000005	2,135000005	sp|Q5NVH5|ALBU_PONAB	Serum albumin OS=Pongo abelii GN=ALB PE=2 SV=2	PONAB	1
23	0	0,9	2,167000063	2,167000063	2,167000063	sp|Q28522|ALBU_MACMU	Serum albumin (Fragment) OS=Macaca mulatta GN=ALB PE=2 SV=1	MACMU	1
23	0	0,9	2,135000005	2,135000005	2,135000005	sp|P02768|ALBU_HUMAN	Serum albumin OS=Homo sapiens GN=ALB PE=1 SV=2	HUMAN	1
23	0	0,9	2,137999982	2,137999982	2,137999982	sp|A2V9Z4|ALBU_MACFA	Serum albumin OS=Macaca fascicularis GN=ALB PE=2 SV=1	MACFA	1
24	0,55	0,55	5,601000041	2,142000012	2,142000012	sp|P02769|ALBU_BOVIN	Serum albumin OS=Bos taurus GN=ALB PE=1 SV=4	BOVIN	1
25	0,36	0,36	1,765999943	1,765999943	1,765999943	sp|P21910|LAML2_XENLA	Lamin-L(II) OS=Xenopus laevis PE=2 SV=1	XENLA	1
26	0,3	0,3	6,577000022	1,477000024	0	sp|Q8WNW3|PLAK_PIG	Junction plakoglobin OS=Sus scrofa GN=Jup PE=2 SV=1	PIG	0
26	0	0,3	6,577000022	1,477000024	0	sp|Q8SPJ1|PLAK_BOVIN	Junction plakoglobin OS=Bos taurus GN=JUP PE=2 SV=1	BOVIN	0
26	0	0,3	6,577000022	1,477000024	0	sp|Q6P0K8|PLAK_RAT	Junction plakoglobin OS=Rattus norvegicus GN=Jup PE=1 SV=1	RAT	0
26	0	0,3	6,577000022	1,477000024	0	sp|Q02257|PLAK_MOUSE	Junction plakoglobin OS=Mus musculus GN=Jup PE=1 SV=3	MOUSE	0
26	0	0,3	6,577000022	1,477000024	0	sp|P14923|PLAK_HUMAN	Junction plakoglobin OS=Homo sapiens GN=JUP PE=1 SV=3	HUMAN	0
27	0,15	0,15	0,359000009	0,359000009	0	RRRRRsp|Q19317|NBEA_CAEEI	REVERSED Putative neurobeachin homolog OS=Caenorhabditis eleg	CAEEL	0
27	0	0,15	0,355600007	0,355600007	0	RRRRRsp|A8XSV3|NBEA_CAEBI	REVERSED Putative neurobeachin homolog OS=Caenorhabditis brigg	CAEBR	0
28	0,12	0,12	3,908000141	1,81099996	0	sp|Q02413|DSG1_HUMAN	Desmoglein-1 OS=Homo sapiens GN=DSG1 PE=1 SV=2	HUMAN	0
28	0	0,12	1,817999966	1,817999966	0	sp|Q3BDI7|DSG1_PIG	Desmoglein-1 OS=Sus scrofa GN=DSG1 PE=2 SV=1	PIG	0
28	0	0,12	1,821999997	1,821999997	0	sp|Q03763|DSG1_BOVIN	Desmoglein-1 OS=Bos taurus GN=DSG1 PE=1 SV=1	BOVIN	0
29	0,1	0,1	2,425999939	2,425999939	0	sp|Q0CQH1|NOP58_ASPTN	Nucleolar protein 58 OS=Aspergillus terreus (strain NIH 2624 / FGSC	ASPTN	0
30	0,09	0,09	1,631999947	1,631999947	0	sp|Q42191|OXA1_ARATH	Mitochondrial inner membrane protein OXA1 OS=Arabidopsis thalia	ARATH	0
31	0,08	0,08	1,917999983	1,917999983	0	RRRRRsp|Q9SFC7|FB135_ARAT	REVERSED F-box protein At3g07870 OS=Arabidopsis thaliana GN=At	ARATH	0
32	0,05	0,05	0,245500007	0,245500007	0	sp|Q756G2|TOM1_ASHGO	Probable E3 ubiquitin-protein ligase TOM1 OS=Ashbya gossypii (stra	ASHGO	0
33	0,05	0,05	4,241999984	4,241999984	0	sp|Q5I0K7|ALG13_RAT	UDP-N-acetylglucosamine transferase subunit ALG13 homolog OS=F	RAT	0
33	0	0,05	3,333000094	3,333000094	0	sp|B1XPZ8|HIS1_SYNP2	ATP phosphoribosyltransferase OS=Synechococcus sp. (strain ATCC	SYNP2	0
34	0,05	0,05	0,608400023	0,608400023	0	RRRRRsp|Q9NRP7|STK36_HUM	REVERSED Serine/threonine-protein kinase 36 OS=Homo sapiens GI	HUMAN	0
34	0	0,05	0,607899996	0,607899996	0	RRRRRsp|Q69ZM6|STK36_MOI	REVERSED Serine/threonine-protein kinase 36 OS=Mus musculus GI	MOUSE	0
35	0,05	0,05	2,00500004	2,00500004	0	RRRRRsp|Q21DC2|RLMN_RHO	REVERSED Dual-specificity RNA methyltransferase RlmN OS=Rhodop	RHOPB	0

N	CSV_2	Total	%Cov	%Cov(50)	%Cov(95)	Accession	Name	Species	Peptides(95%)
3	43,27	43,27	58,4399998	58,4399998	58,4399998	sp|P00761|TRYP_PIG	Trypsin OS=Sus scrofa PE=1 SV=1	PIG	34
1	54,17	54,17	57,2799981	54,7699988	52,8999984	sp|P35908|K22E_HUMAN	Keratin, type II cytoskeletal 2 epidermal OS=Homo sapi	HUMAN	28
4	40,02	48,04	46,9399989	43,7999994	32,9699993	sp|A5A6M6|K2C1_PANTR	Keratin, type II cytoskeletal 1 OS=Pan troglodytes GN=K	PANTR	28
4	0	48,02	42,6999986	39,5999998	32,6099992	sp|P04264|K2C1_HUMAN	Keratin, type II cytoskeletal 1 OS=Homo sapiens GN=KR	HUMAN	28
2	46,08	46,08	51,3700008	47,7699995	47,7699995	sp|P13645|K1C10_HUMAN	Keratin, type I cytoskeletal 10 OS=Homo sapiens GN=KI	HUMAN	27
5	26	26	41,5699989	32,100001	32,100001	sp|P35527|K1C9_HUMAN	Keratin, type I cytoskeletal 9 OS=Homo sapiens GN=KR	HUMAN	14
12	2	18	13,1200001	13,1200001	13,1200001	sp|P04259|K2C6B_HUMAN	Keratin, type II cytoskeletal 6B OS=Homo sapiens GN=K	HUMAN	9
6	16	16	71,0099995	71,0099995	71,0099995	sp|P15252|REF_HEVBR	Rubber elongation factor protein OS=Hevea brasiliensis	HEVBR	8
12	0	16	11,3499999	11,3499999	11,3499999	sp|P48668|K2C6C_HUMAN	Keratin, type II cytoskeletal 6C OS=Homo sapiens GN=K	HUMAN	8
12	0	16	11,3499999	11,3499999	11,3499999	sp|P02538|K2C6A_HUMAN	Keratin, type II cytoskeletal 6A OS=Homo sapiens GN=K	HUMAN	8
13	2	14	11,1900002	9,15300027	9,15300027	sp|P13647|K2C5_HUMAN	Keratin, type II cytoskeletal 5 OS=Homo sapiens GN=KR	HUMAN	7
13	0	14	11,1500002	9,12199989	9,12199989	sp|A5A6M8|K2C5_PANTR	Keratin, type II cytoskeletal 5 OS=Pan troglodytes GN=K	PANTR	7
7	4,01	8,01	16,6999996	9,93700027	9,93700027	sp|P08779|K1C16_HUMAN	Keratin, type I cytoskeletal 16 OS=Homo sapiens GN=KI	HUMAN	4
14	2	8,01	19,4900006	9,95799974	9,95799974	sp|P02533|K1C14_HUMAN	Keratin, type I cytoskeletal 14 OS=Homo sapiens GN=KI	HUMAN	4
8	4	4	25,4500002	23,6399993	23,6399993	sp|P81605|DCD_HUMAN	Dermcidin OS=Homo sapiens GN=DCD PE=1 SV=2	HUMAN	2
9	4	4	19,1200003	19,1200003	12,7499998	sp|O82803|SRPP_HEVBR	Small rubber particle protein OS=Hevea brasiliensis GN	HEVBR	2
10	4	4	5,58300018	5,58300018	5,58300018	sp|Q5NVH5|ALBU_PONAB	Serum albumin OS=Pongo abelii GN=ALB PE=2 SV=2	PONAB	2
10	0	4	5,58300018	5,58300018	5,58300018	sp|P02768|ALBU_HUMAN	Serum albumin OS=Homo sapiens GN=ALB PE=1 SV=2	HUMAN	2
11	4	4	24,56	24,56	24,56	sp|P06702|S10A9_HUMAN	Protein S100-A9 OS=Homo sapiens GN=S100A9 PE=1 S	HUMAN	2
10	0	2	2,16700006	2,16700006	2,16700006	sp|Q28522|ALBU_MACMU	Serum albumin (Fragment) OS=Macaca mulatta GN=AL	MACMU	1
10	0	2	2,13799998	2,13799998	2,13799998	sp|A2V9Z4|ALBU_MACFA	Serum albumin OS=Macaca fascicularis GN=ALB PE=2 S	MACFA	1
15	2	2	34,4099998	22,5799993	22,5799993	sp|P05109|S10A8_HUMAN	Protein S100-A8 OS=Homo sapiens GN=S100A8 PE=1 S	HUMAN	1
16	2	2	5,60100004	2,14200001	2,14200001	sp|P02769|ALBU_BOVIN	Serum albumin OS=Bos taurus GN=ALB PE=1 SV=4	BOVIN	1
17	2	2	4,44400012	4,44400012	4,44400012	sp|Q9A1W2|RL5_STRP1	50S ribosomal protein L5 OS=Streptococcus pyogenes s	STRP1	1
17	0	2	4,44400012	4,44400012	4,44400012	sp|Q97SV1|RL5_STRPN	50S ribosomal protein L5 OS=Streptococcus pneumonia	STRPN	1
17	0	2	4,44400012	4,44400012	4,44400012	sp|Q8E7S9|RL5_STRA3	50S ribosomal protein L5 OS=Streptococcus agalactiae	STRA3	1
17	0	2	4,44400012	4,44400012	4,44400012	sp|Q8E2C2|RL5_STRA5	50S ribosomal protein L5 OS=Streptococcus agalactiae	STRA5	1
17	0	2	4,44400012	4,44400012	4,44400012	sp|Q8CWV4|RL5_STRR6	50S ribosomal protein L5 OS=Streptococcus pneumonia	STRR6	1
17	0	2	4,46900018	4,46900018	4,46900018	sp|Q81VR8|RL5_BACAN	50S ribosomal protein L5 OS=Bacillus anthracis GN=rplE	BACAN	1
17	0	2	4,46900018	4,46900018	4,46900018	sp|Q81J30|RL5_BACCR	50S ribosomal protein L5 OS=Bacillus cereus (strain ATC	BACCR	1
17	0	2	4,44400012	4,44400012	4,44400012	sp|Q7CNP5|RL5_STRP8	50S ribosomal protein L5 OS=Streptococcus pyogenes s	STRP8	1
17	0	2	4,46900018	4,46900018	4,46900018	sp|Q73F84|RL5_BACC1	50S ribosomal protein L5 OS=Bacillus cereus (strain ATC	BACC1	1
17	0	2	4,46900018	4,46900018	4,46900018	sp|Q6HPP6|RL5_BACHK	50S ribosomal protein L5 OS=Bacillus thuringiensis subs	BACHK	1
17	0	2	4,46900018	4,46900018	4,46900018	sp|Q63H78|RL5_BACCZ	50S ribosomal protein L5 OS=Bacillus cereus (strain ZK /	BACCZ	1
17	0	2	4,44400012	4,44400012	4,44400012	sp|Q5XEC3|RL5_STRP6	50S ribosomal protein L5 OS=Streptococcus pyogenes s	STRP6	1
17	0	2	4,46900018	4,46900018	4,46900018	sp|Q5WLQ0|RL5_BACSK	50S ribosomal protein L5 OS=Bacillus clausii (strain KSM	BACSK	1
17	0	2	4,44400012	4,44400012	4,44400012	sp|Q5M2C5|RL5_STRT2	50S ribosomal protein L5 OS=Streptococcus thermophil	STRT2	1
17	0	2	4,44400012	4,44400012	4,44400012	sp|Q5LXS3|RL5_STRT1	50S ribosomal protein L5 OS=Streptococcus thermophil	STRT1	1
17	0	2	4,44400012	4,44400012	4,44400012	sp|Q48VT7|RL5_STRPM	50S ribosomal protein L5 OS=Streptococcus pyogenes s	STRPM	1
17	0	2	4,44400012	4,44400012	4,44400012	sp|Q3K3V7|RL5_STRA1	50S ribosomal protein L5 OS=Streptococcus agalactiae	STRA1	1
17	0	2	4,44400012	4,44400012	4,44400012	sp|Q1WSA2|RL5_LACS1	50S ribosomal protein L5 OS=Lactobacillus salivarius (st	LACS1	1
17	0	2	4,44400012	4,44400012	4,44400012	sp|Q1JP05|RL5_STRPC	50S ribosomal protein L5 OS=Streptococcus pyogenes s	STRPC	1
17	0	2	4,44400012	4,44400012	4,44400012	sp|Q1JJ50|RL5_STRPD	50S ribosomal protein L5 OS=Streptococcus pyogenes s	STRPD	1
17	0	2	4,44400012	4,44400012	4,44400012	sp|Q1JE46|RL5_STRPB	50S ribosomal protein L5 OS=Streptococcus pyogenes s	STRPB	1
17	0	2	4,44400012	4,44400012	4,44400012	sp|Q1J901|RL5_STRPF	50S ribosomal protein L5 OS=Streptococcus pyogenes s	STRPF	1
17	0	2	4,44400012	4,44400012	4,44400012	sp|Q04MM4|RL5_STRP2	50S ribosomal protein L5 OS=Streptococcus pneumonia	STRP2	1
17	0	2	4,44400012	4,44400012	4,44400012	sp|Q03IG3|RL5_STRTD	50S ribosomal protein L5 OS=Streptococcus thermophil	STRTD	1
17	0	2	4,44400012	4,44400012	4,44400012	sp|P0DE57|RL5_STRPQ	50S ribosomal protein L5 OS=Streptococcus pyogenes s	STRPQ	1
17	0	2	4,44400012	4,44400012	4,44400012	sp|P0DE56|RL5_STRP3	50S ribosomal protein L5 OS=Streptococcus pyogenes s	STRP3	1
17	0	2	4,46900018	4,46900018	4,46900018	sp|C6E4P5|RL5_GEOSM	50S ribosomal protein L5 OS=Geobacter sp. (strain M21	GEOSM	1
17	0	2	4,46900018	4,46900018	4,46900018	sp|C3P9R7|RL5_BACAA	50S ribosomal protein L5 OS=Bacillus anthracis (strain A	BACAA	1
17	0	2	4,46900018	4,46900018	4,46900018	sp|C3LJ94|RL5_BACAC	50S ribosomal protein L5 OS=Bacillus anthracis (strain C	BACAC	1
17	0	2	4,46900018	4,46900018	4,46900018	sp|C1ET51|RL5_BACC3	50S ribosomal protein L5 OS=Bacillus cereus (strain 03E	BACC3	1
17	0	2	4,44400012	4,44400012	4,44400012	sp|C1CPA0|RL5_STRZT	50S ribosomal protein L5 OS=Streptococcus pneumonia	STRZT	1
17	0	2	4,44400012	4,44400012	4,44400012	sp|C1CIA9|RL5_STRZP	50S ribosomal protein L5 OS=Streptococcus pneumonia	STRZP	1
17	0	2	4,44400012	4,44400012	4,44400012	sp|C1CC18|RL5_STRZJ	50S ribosomal protein L5 OS=Streptococcus pneumonia	STRZJ	1
17	0	2	4,44400012	4,44400012	4,44400012	sp|C1CAM4|RL5_STRP7	50S ribosomal protein L5 OS=Streptococcus pneumonia	STRP7	1
17	0	2	4,44400012	4,44400012	4,44400012	sp|C0MCC2|RL5_STRS7	50S ribosomal protein L5 OS=Streptococcus equi subsp	STRS7	1
17	0	2	4,44400012	4,44400012	4,44400012	sp|C0M9H3|RL5_STRE4	50S ribosomal protein L5 OS=Streptococcus equi subsp	STRE4	1
17	0	2	4,46900018	4,46900018	4,46900018	sp|B9IZK6|RL5_BACCQ	50S ribosomal protein L5 OS=Bacillus cereus (strain Q1)	BACCQ	1
17	0	2	4,44400012	4,44400012	4,44400012	sp|B8ZKG9|RL5_STRPJ	50S ribosomal protein L5 OS=Streptococcus pneumonia	STRPJ	1
17	0	2	4,46900018	4,46900018	4,46900018	sp|B7JKD1|RL5_BACC0	50S ribosomal protein L5 OS=Bacillus cereus (strain AH8	BACC0	1
17	0	2	4,46900018	4,46900018	4,46900018	sp|B7IT31|RL5_BACC2	50S ribosomal protein L5 OS=Bacillus cereus (strain G98	BACC2	1
17	0	2	4,46900018	4,46900018	4,46900018	sp|B7HQV6|RL5_BACC7	50S ribosomal protein L5 OS=Bacillus cereus (strain AH1	BACC7	1
17	0	2	4,46900018	4,46900018	4,46900018	sp|B7HJ60|RL5_BACC4	50S ribosomal protein L5 OS=Bacillus cereus (strain B42	BACC4	1
17	0	2	4,46900018	4,46900018	4,46900018	sp|B7GJ79|RL5_ANOFW	50S ribosomal protein L5 OS=Anoxybacillus flavithermu	ANOFW	1
17	0	2	4,44400012	4,44400012	4,44400012	sp|B5XJ48|RL5_STRPZ	50S ribosomal protein L5 OS=Streptococcus pyogenes s	STRPZ	1
17	0	2	4,46900018	4,46900018	4,46900018	sp|B5EFR2|RL5_GEOBB	50S ribosomal protein L5 OS=Geobacter bemidjiensis (s	GEOBB	1
17	0	2	4,44400012	4,44400012	4,44400012	sp|B5E6G7|RL5_STRP4	50S ribosomal protein L5 OS=Streptococcus pneumonia	STRP4	1
17	0	2	4,44400012	4,44400012	4,44400012	sp|B4U512|RL5_STREM	50S ribosomal protein L5 OS=Streptococcus equi subsp	STREM	1
17	0	2	4,44400012	4,44400012	4,44400012	sp|B2IS52|RL5_STRPS	50S ribosomal protein L5 OS=Streptococcus pneumonia	STRPS	1
17	0	2	4,44400012	4,44400012	4,44400012	sp|B1I8L0|RL5_STRPI	50S ribosomal protein L5 OS=Streptococcus pneumonia	STRPI	1
17	0	2	4,46900018	4,46900018	4,46900018	sp|A9VP89|RL5_BACWK	50S ribosomal protein L5 OS=Bacillus weihenstephanen	BACWK	1
17	0	2	4,44400012	4,44400012	4,44400012	sp|A8AZL3|RL5_STRGC	50S ribosomal protein L5 OS=Streptococcus gordonii (st	STRGC	1
17	0	2	4,44400012	4,44400012	4,44400012	sp|A3CK75|RL5_STRSV	50S ribosomal protein L5 OS=Streptococcus sanguinis (s	STRSV	1
17	0	2	4,44400012	4,44400012	4,44400012	sp|A2RC26|RL5_STRPG	50S ribosomal protein L5 OS=Streptococcus pyogenes s	STRPG	1
17	0	2	4,46900018	4,46900018	4,46900018	sp|A0R8J2|RL5_BACAH	50S ribosomal protein L5 OS=Bacillus thuringiensis (stra	BACAH	1
18	2	2	0,46009999	0,46009999	0,46009999	sp|Q5D862|FILA2_HUMAN	Filaggrin-2 OS=Homo sapiens GN=FLG2 PE=1 SV=1	HUMAN	1
19	2	2	1,63199995	1,63199995	1,63199995	sp|Q42191|OXA1_ARATH	Mitochondrial inner membrane protein OXA1 OS=Arabi	ARATH	1
20	2	2	1,89699996	1,89699996	1,89699996	sp|Q10CE4|GLO1_ORYSJ	Peroxisomal (S)-2-hydroxy-acid oxidase GLO1 OS=Oryza	ORYSJ	1
20	0	2	1,89699996	1,89699996	1,89699996	sp|B8AKX6|GLO1_ORYSI	Peroxisomal (S)-2-hydroxy-acid oxidase GLO1 OS=Oryza	ORYSI	1
21	2	2	0,87050004	0,87050004	0,87050004	sp|Q05884|AMY_STRLI	Alpha-amylase OS=Streptomyces lividans GN=amy PE=1	STRLI	1
22	2	2	3,78299989	3,78299989	3,78299989	sp|P49065|ALBU_RABIT	Serum albumin OS=Oryctolagus cuniculus GN=ALB PE=1	RABIT	1
23	0,33	0,33	1,91799998	1,91799998	1,91799998	RRRRRsp|Q9SFC7|FB135_ARATH	REVERSED F-box protein At3g07870 OS=Arabidopsis tha	ARATH	1
24	0,09	2	100	100	100	sp|P84735|PS19_PINST	Putative LRR disease resistance protein/transmembran	PINST	1
24	0	2	100	100	100	sp|P84732|PS16_PINST	Putative LRR disease resistance protein/transmembran	PINST	1
24	0	2	100	100	100	sp|P84720|PS4_PINST	Putative LRR disease resistance protein/transmembran	PINST	1

N	CSV_3	Total	%Cov	%Cov(50)	%Cov(95)	Accession	Name	Species	Peptides(95%)
1	21,82	21,82	44,9000001	22,29000032	20,56999952	sp|A5A6M6|K2C1_PANTR	Keratin, type II cytoskeletal 1 OS=Pan troglodytes	PANTR	11
1	0	19,65	42,39000082	19,41000074	17,70000011	sp|P04264|K2C1_HUMAN	Keratin, type II cytoskeletal 1 OS=Homo sapiens G	HUMAN	10
2	15,52	15,52	36,80000007	32,89999962	32,89999962	sp|P00761|TRYP_PIG	Trypsin OS=Sus scrofa PE=1 SV=1	PIG	18
3	4,29	4,29	35,04000008	7,041999698	4,930000007	sp|Q6EIZ0|K1C10_CANLF	Keratin, type I cytoskeletal 10 OS=Canis lupus fan	CANLF	2
3	0	4,29	32,53000081	6,848999858	4,794999957	sp|P13645|K1C10_HUMAN	Keratin, type I cytoskeletal 10 OS=Homo sapiens (	HUMAN	2
4	4,06	4,06	31,61999881	5,9390001	5,9390001	sp|P35527|K1C9_HUMAN	Keratin, type I cytoskeletal 9 OS=Homo sapiens G	HUMAN	2
5	3,1	3,1	11,85000017	5,491000041	5,491000041	sp|Q72KU3|PYRD_THET2	Dihydroorotate dehydrogenase (quinone) OS=The	THET2	2
6	0,05	0,05	1,844999939	0	0	RRRRRsp|Q7VN62|UBIB_HAEDU	REVERSED Probable protein kinase UbiB OS=Haer	HAEDU	0

N	Ctrldudoso_CSVI_1	Total	%Cov	%Cov(50)	%Cov(95)	Accession	Name	Species	Peptides(95%)
1	78,22	78,22	69,24999952	66,61000252	57,60999918	sp|P04264|K2C1_HUMAN	Keratin, type II cytoskeletal 1 OS=Homo sapiens GN=KRT1 I	HUMAN	61
2	62,37	62,37	62,5	52,2300005	52,05000043	sp|P13645|K1C10_HUMAN	Keratin, type I cytoskeletal 10 OS=Homo sapiens GN=KRT1(	HUMAN	56
3	49,5	58,27	74,01999831	61,6599977	50,55000186	sp|P35908|K22E_HUMAN	Keratin, type II cytoskeletal 2 epidermal OS=Homo sapiens	HUMAN	37
4	42,41	42,41	69,81999874	63,08000088	57,45999813	sp|P35527|K1C9_HUMAN	Keratin, type I cytoskeletal 9 OS=Homo sapiens GN=KRT9 P	HUMAN	33
18	2	27,56	32,74999857	30,98999858	29,39999998	sp|Q6EIZ0|K1C10_CANLF	Keratin, type I cytoskeletal 10 OS=Canis lupus familiaris GN	CANLF	25
7	13,59	26,47	36,61000133	27,9700011	24,92000014	sp|P13647|K2C5_HUMAN	Keratin, type II cytoskeletal 5 OS=Homo sapiens GN=KRT5 I	HUMAN	19
7	0	24,47	33,950001	25,33999979	22,30000049	sp|A5A6M8|K2C5_PANTR	Keratin, type II cytoskeletal 5 OS=Pan troglodytes GN=KRT5	PANTR	18
11	0	24,61	29,96000051	27,30000019	18,79000068	sp|P48668|K2C6C_HUMAN	Keratin, type II cytoskeletal 6C OS=Homo sapiens GN=KRT6	HUMAN	17
11	0	24,61	29,96000051	27,30000019	18,79000068	sp|P02538|K2C6A_HUMAN	Keratin, type II cytoskeletal 6A OS=Homo sapiens GN=KRT6	HUMAN	17
11	4	24,68	29,96000051	24,2899999	18,97000074	sp|P04259|K2C6B_HUMAN	Keratin, type II cytoskeletal 6B OS=Homo sapiens GN=KRT6	HUMAN	16
5	19,37	23,82	55,29999733	41,53000116	31,77999854	sp|P02533|K1C14_HUMAN	Keratin, type I cytoskeletal 14 OS=Homo sapiens GN=KRT14	HUMAN	14
8	8,82	8,82	41,99000001	38,96000087	32,89999962	sp|P00761|TRYP_PIG	Trypsin OS=Sus scrofa PE=1 SV=1	PIG	13
6	18,16	22,65	41,99999869	28,16999853	25,33000112	sp|Q9NSB2|KRT84_HUMAN	Keratin, type II cuticular Hb4 OS=Homo sapiens GN=KRT84	HUMAN	12
9	7,03	21,43	54,3299973	33,61999989	31,70999885	sp|P08779|K1C16_HUMAN	Keratin, type I cytoskeletal 16 OS=Homo sapiens GN=KRT1(	HUMAN	12
12	4	14,16	23,3099997	20,03999949	18,1099996	sp|Q6IG01|K2C1B_RAT	Keratin, type II cytoskeletal 1b OS=Rattus norvegicus GN=K	RAT	10
12	0	12,09	16,9599995	16,07999951	14,33999985	sp|Q6IFZ6|K2C1B_MOUSE	Keratin, type II cytoskeletal 1b OS=Mus musculus GN=Krt7	MOUSE	9
19	2	8,93	29,39999998	18,28999966	16,20000005	sp|Q04695|K1C17_HUMAN	Keratin, type I cytoskeletal 17 OS=Homo sapiens GN=KRT1	HUMAN	6
19	0	7,82	25	13,88999969	11,81000024	sp|A5A6M0|K1C17_PANTR	Keratin, type I cytoskeletal 17 OS=Pan troglodytes GN=KRT	PANTR	5
13	3,84	4,69	21,62999958	11,2999998	8,173000067	sp|Q15323|K1H1_HUMAN	Keratin, type I cuticular Ha1 OS=Homo sapiens GN=KRT31 I	HUMAN	3
13	0	4,69	21,62999958	11,2999998	8,173000067	sp|A5A6M5|K1H1_PANTR	Keratin, type I cuticular Ha1 OS=Pan troglodytes GN=KRT3	PANTR	3
13	0	4,07	8,252000064	8,252000064	8,252000064	sp|P02534|K1M1_SHEEP	Keratin, type I microfibrillar 48 kDa, component 8C-1 OS=C	SHEEP	3
14	3,72	4,92	9,670999646	9,670999646	9,670999646	sp|O43790|KRT86_HUMAN	Keratin, type II cuticular Hb6 OS=Homo sapiens GN=KRT86	HUMAN	3
37	1,72	4,55	14,84999955	7,920999825	7,920999825	sp|Q14525|KT33B_HUMAN	Keratin, type I cuticular Ha3-II OS=Homo sapiens GN=KRT3	HUMAN	3
10	4,45	4,45	53,61999869	40,58000147	18,11999977	sp|P15252|REF_HEVBR	Rubber elongation factor protein OS=Hevea brasiliensis PE	HEVBR	2
13	0	3,32	13,00999969	10,19999981	6,887999922	sp|Q9D646|KRT34_MOUSE	Keratin, type I cuticular Ha4 OS=Mus musculus GN=Krt34 F	MOUSE	2
13	0	2,84	7,142999768	7,142999768	4,241000116	sp|Q14532|K1H2_HUMAN	Keratin, type I cuticular Ha2 OS=Homo sapiens GN=KRT32 I	HUMAN	2
13	0	2,19	8,41299966	8,41299966	5,288000032	sp|Q61765|K1H1_MOUSE	Keratin, type I cuticular Ha1 OS=Mus musculus GN=Krt31 F	MOUSE	2
14	0	3,09	5,347000062	5,347000062	5,347000062	sp|Q14533|KRT81_HUMAN	Keratin, type II cuticular Hb1 OS=Homo sapiens GN=KRT81	HUMAN	2
14	0	3,09	5,477000028	5,477000028	5,477000028	sp|P78385|KRT83_HUMAN	Keratin, type II cuticular Hb3 OS=Homo sapiens GN=KRT83	HUMAN	2
15	3,02	3,02	3,657000139	1,183999982	0,801100023	sp|P15924|DESP_HUMAN	Desmoplakin OS=Homo sapiens GN=DSP PE=1 SV=3	HUMAN	2
16	2,93	2,93	22,73000032	12,72999942	12,72999942	sp|P81605|DCD_HUMAN	Dermcidin OS=Homo sapiens GN=DCD PE=1 SV=2	HUMAN	2
20	2	2,64	4,68400009	4,68400009	4,68400009	sp|Q6IFX1|K1C24_RAT	Keratin, type I cytoskeletal 24 OS=Rattus norvegicus GN=Kı	RAT	2
20	0	2,64	4,492000118	4,492000118	4,492000118	sp|A1L317|K1C24_MOUSE	Keratin, type I cytoskeletal 24 OS=Mus musculus GN=Krt24	MOUSE	2
39	1,57	2,44	7,495000213	4,283000156	4,283000156	sp|O76013|KRT36_HUMAN	Keratin, type I cuticular Ha6 OS=Homo sapiens GN=KRT36 I	HUMAN	2
39	0	2,44	4,22799997	4,22799997	4,22799997	sp|B1AQ75|KRT36_MOUSE	Keratin, type I cuticular Ha6 OS=Mus musculus GN=Krt36 F	MOUSE	2
13	0	2,2	10,14999971	6,187999994	2,969999984	sp|O76009|KT33A_HUMAN	Keratin, type I cuticular Ha3-I OS=Homo sapiens GN=KRT33	HUMAN	1
13	0	2,2	10,14999971	6,187999994	2,969999984	sp|A5A6P3|KT33A_PANTR	Keratin, type I cuticular Ha3-I OS=Pan troglodytes GN=KRT	PANTR	1
13	0	1,31	9,652999789	6,931000203	3,712999821	sp|Q8K0Y2|KT33A_MOUSE	Keratin, type I cuticular Ha3-I OS=Mus musculus GN=Krt33	MOUSE	1
15	0	2,23	1,665000059	0,832500029	0,450900011	sp|E9Q557|DESP_MOUSE	Desmoplakin OS=Mus musculus GN=Dsp PE=1 SV=1	MOUSE	1
17	2,03	2,03	46,23999894	22,57999927	22,57999927	sp|P05109|S10A8_HUMAN	Protein S100-A8 OS=Homo sapiens GN=S100A8 PE=1 SV=1	HUMAN	1
21	2	2	7,323999703	1,465000026	1,465000026	sp|A7ZWZ1|BGAL_ECOHS	Beta-galactosidase OS=Escherichia coli O9:H4 (strain HS) G	ECOHS	1
21	0	2	7,323999703	1,465000026	1,465000026	sp|A7ZI91|BGAL_ECO24	Beta-galactosidase OS=Escherichia coli O139:H28 (strain E2	ECO24	1
21	0	2	6,055000052	1,465000026	1,465000026	sp|Q3Z583|BGAL_SHISS	Beta-galactosidase OS=Shigella sonnei (strain Ss046) GN=la	SHISS	1
21	0	2	4,58999984	1,465000026	1,465000026	sp|P00722|BGAL_ECOLI	Beta-galactosidase OS=Escherichia coli (strain K12) GN=lac	ECOLI	1
21	0	2	4,58999984	1,465000026	1,465000026	sp|B1J0T5|BGAL_ECOLC	Beta-galactosidase OS=Escherichia coli (strain ATCC 8739 /	ECOLC	1
21	0	2	3,303999826	1,458000019	1,458000019	sp|Q8VNN2|BGAL_ECOLX	Beta-galactosidase OS=Escherichia coli GN=lacZ PE=3 SV=1	ECOLX	1
22	2	2	8,668000251	4,22799997	4,22799997	RRRRRsp|P08779|K1C16_HUMAN	REVERSED Keratin, type I cytoskeletal 16 OS=Homo sapiens	HUMAN	1
23	2	2	0,961900037	0,460099988	0,460099988	sp|Q5D862|FILA2_HUMAN	Filaggrin-2 OS=Homo sapiens GN=FLG2 PE=1 SV=1	HUMAN	1
24	2	2	28,13000083	19,37000006	19,37000006	sp|Q3LI77|KR134_HUMAN	Keratin-associated protein 13-4 OS=Homo sapiens GN=KRT	HUMAN	1
25	2	2	16,43999964	10,96000001	10,96000001	sp|P12273|PIP_HUMAN	Prolactin-inducible protein OS=Homo sapiens GN=PIP PE=1	HUMAN	1
25	0	2	10,96000001	10,96000001	10,96000001	sp|P60989|PIP_PANTR	Prolactin-inducible protein homolog OS=Pan troglodytes G	PANTR	1
26	2	2	24,56	11,40000001	11,40000001	sp|P06702|S10A9_HUMAN	Protein S100-A9 OS=Homo sapiens GN=S100A9 PE=1 SV=1	HUMAN	1
27	2	2	1,511000004	1,511000004	1,511000004	sp|Q9FMA3|PEX5_ARATH	Peroxisome biogenesis protein 5 OS=Arabidopsis thaliana (	ARATH	1
28	2	2	2,096999995	2,096999995	2,096999995	sp|Q2KZ15|DXS_BORA1	1-deoxy-D-xylulose-5-phosphate synthase OS=Bordetella a	BORA1	1
29	2	2	4,072000086	4,072000086	4,072000086	sp|P21883|ODP2_BACSU	Dihydrolipoyllysine-residue acetyltransferase component c	BACSU	1
30	2	2	5,606999993	5,606999993	5,606999993	sp|P02662|CASA1_BOVIN	Alpha-S1-casein OS=Bos taurus GN=CSN1S1 PE=1 SV=2	BOVIN	1
30	0	2	5,606999993	5,606999993	5,606999993	sp|O62823|CASA1_BUBBU	Alpha-S1-casein OS=Bubalus bubalis GN=CSN1S1 PE=2 SV=	BUBBU	1
31	2	2	7,353000343	7,353000343	7,353000343	sp|O82803|SRPP_HEVBR	Small rubber particle protein OS=Hevea brasiliensis GN=SR	HEVBR	1
32	2	2	2,199999988	2,199999988	2,199999988	sp|O64793|Y1675_ARATH	G-type lectin S-receptor-like serine/threonine-protein kina	ARATH	1
33	2	2	1,286000013	1,286000013	1,286000013	sp|F1MDL2|INTU_BOVIN	Protein inturned OS=Bos taurus GN=INTU PE=3 SV=2	BOVIN	1
34	2	2	1,28899999	1,28899999	1,28899999	sp|A6ZP10|SEY1_YEAS7	Protein SEY1 OS=Saccharomyces cerevisiae (strain YJM789	YEAS7	1
35	2	2	0,897500012	0,897500012	0,897500012	sp|A1A316|RPOC_BIFAA	DNA-directed RNA polymerase subunit beta' OS=Bifidobac	BIFAA	1
36	1,89	2	4,478000104	4,478000104	4,478000104	sp|Q5RAB4|G3P_PONAB	Glyceraldehyde-3-phosphate dehydrogenase OS=Pongo ab	PONAB	1
36	0	2	4,478000104	4,478000104	4,478000104	sp|P04406|G3P_HUMAN	Glyceraldehyde-3-phosphate dehydrogenase OS=Homo saj	HUMAN	1
38	1,64	1,64	13,30000013	4,14500013	4,14500013	sp|Q5T749|KPRP_HUMAN	Keratinocyte proline-rich protein OS=Homo sapiens GN=KF	HUMAN	1
40	1,15	1,15	10,86999997	3,415999934	3,415999934	sp|P05089|ARGI1_HUMAN	Arginase-1 OS=Homo sapiens GN=ARG1 PE=1 SV=2	HUMAN	1
41	0,99	0,99	1,030999981	1,030999981	1,030999981	sp|A4J927|SECA_DESRM	Protein translocase subunit SecA OS=Desulfotomaculum re	DESRM	1
42	0,95	0,95	1,683999971	1,683999971	1,683999971	sp|Q86YZ3|HORN_HUMAN	Hornerin OS=Homo sapiens GN=HRNR PE=1 SV=2	HUMAN	1
43	0,86	0,86	1,882999949	1,882999949	1,882999949	sp|Q50339|Y588_MYCPN	Uncharacterized lipoprotein MPN_588 OS=Mycoplasma pr	MYCPN	1
45	0,65	0,65	3,398999944	3,398999944	3,398999944	sp|A5EKG6|PLSX_BRASB	Phosphate acyltransferase OS=Bradyrhizobium sp. (strain E	BRASB	1
45	0	0,65	3,398999944	3,398999944	3,398999944	sp|A4YW85|PLSX_BRASO	Phosphate acyltransferase OS=Bradyrhizobium sp. (strain (	BRASO	1
46	0,38	0,38	6,25	6,25	6,25	RRRRRsp|Q8PM31|3HAO_XANAC	REVERSED 3-hydroxyanthranilate 3,4-dioxygenase OS=Xan	XANAC	1
52	0,11	0,11	1,765999943	1,765999943	1,765999943	sp|P21910|LAML2_XENLA	Lamin-L(II) OS=Xenopus laevis PE=2 SV=1	XENLA	1
44	0,77	0,77	14,22999948	4,966000095	0	sp|Q8WNW3|PLAK_PIG	Junction plakoglobin OS=Sus scrofa GN=Jup PE=2 SV=1	PIG	0
44	0	0,77	14,22999948	4,966000095	0	sp|Q8SPJ1|PLAK_BOVIN	Junction plakoglobin OS=Bos taurus GN=JUP PE=2 SV=1	BOVIN	0
44	0	0,77	14,22999948	4,966000095	0	sp|Q02257|PLAK_MOUSE	Junction plakoglobin OS=Mus musculus GN=Jup PE=1 SV=3	MOUSE	0
44	0	0,77	14,22999948	4,966000095	0	sp|P14923|PLAK_HUMAN	Junction plakoglobin OS=Homo sapiens GN=JUP PE=1 SV=3	HUMAN	0
47	0,36	0,36	2,563999966	2,563999966	0	sp|Q67N85|TDH_SYMTH	L-threonine 3-dehydrogenase OS=Symbiobacterium therm	SYMTH	0
48	0,2	0,2	1,522000041	1,522000041	0	sp|P42357|HUTH_HUMAN	Histidine ammonia-lyase OS=Homo sapiens GN=HAL PE=1 S	HUMAN	0
48	0	0,2	1,522000041	1,522000041	0	sp|P35492|HUTH_MOUSE	Histidine ammonia-lyase OS=Mus musculus GN=Hal PE=1 S	MOUSE	0
48	0	0,2	1,522000041	1,522000041	0	sp|P21213|HUTH_RAT	Histidine ammonia-lyase OS=Rattus norvegicus GN=Hal PE	RAT	0
49	0,18	0,18	1,049000025	1,049000025	0	RRRRRsp|P48479|NIM1_NEUCR	REVERSED G2-specific protein kinase nim-1 OS=Neurospor	NEUCR	0
50	0,17	0,17	30,61000109	12,24000007	0	sp|P01040|CYTA_HUMAN	Cystatin-A OS=Homo sapiens GN=CSTA PE=1 SV=1	HUMAN	0
51	0,13	0,13	1,532999985	1,532999985	0	sp|P13894|LT_BFPYV	Large T antigen OS=Budgerigar fledgling disease virus PE=3	BFPYV	0
53	0,06	0,06	10,19999981	1,716000028	0	sp|Q02413|DSG1_HUMAN	Desmoglein-1 OS=Homo sapiens GN=DSG1 PE=1 SV=2	HUMAN	0
54	0,06	0,06	3,599999845	3,599999845	0	sp|O22328|AGL8_SOLCO	Agamous-like MADS-box protein AGL8 homolog OS=Solanı	SOLCO	0
55	0,05	0,05	2,452000044	2,452000044	0	RRRRRsp|Q81M67|BUK_BACAN	REVERSED Probable butyrate kinase OS=Bacillus anthracis	BACAN	0
55	0	0,05	2,452000044	2,452000044	0	RRRRRsp|Q818T1|BUK_BACCR	REVERSED Probable butyrate kinase OS=Bacillus cereus (st	BACCR	0
55	0	0,05	2,452000044	2,452000044	0	RRRRRsp|Q731D0|BUK_BACC1	REVERSED Probable butyrate kinase OS=Bacillus cereus (st	BACC1	0
55	0	0,05	2,452000044	2,452000044	0	RRRRRsp|Q6HE00|BUK_BACHK	REVERSED Probable butyrate kinase OS=Bacillus thuringien	BACHK	0
55	0	0,05	2,452000044	2,452000044	0	RRRRRsp|Q635C1|BUK_BACCZ	REVERSED Probable butyrate kinase OS=Bacillus cereus (st	BACCZ	0
55	0	0,05	2,452000044	2,452000044	0	RRRRRsp|C3P7U3|BUK_BACAA	REVERSED Probable butyrate kinase OS=Bacillus anthracis	BACAA	0
55	0	0,05	2,452000044	2,452000044	0	RRRRRsp|C3LJT8|BUK_BACAC	REVERSED Probable butyrate kinase OS=Bacillus anthracis	BACAC	0
55	0	0,05	2,452000044	2,452000044	0	RRRRRsp|C1ERN7|BUK_BACC3	REVERSED Probable butyrate kinase OS=Bacillus cereus (st	BACC3	0
55	0	0,05	2,452000044	2,452000044	0	RRRRRsp|B9IXF8|BUK_BACCQ	REVERSED Probable butyrate kinase OS=Bacillus cereus (st	BACCQ	0
55	0	0,05	2,452000044	2,452000044	0	RRRRRsp|B7JM15|BUK_BACC0	REVERSED Probable butyrate kinase OS=Bacillus cereus (st	BACC0	0
55	0	0,05	2,452000044	2,452000044	0	RRRRRsp|B7IXF5|BUK_BACC2	REVERSED Probable butyrate kinase OS=Bacillus cereus (st	BACC2	0
55	0	0,05	2,452000044	2,452000044	0	RRRRRsp|B7HNS7|BUK_BACC7	REVERSED Probable butyrate kinase OS=Bacillus cereus (st	BACC7	0
55	0	0,05	2,452000044	2,452000044	0	RRRRRsp|B7HB35|BUK_BACC4	REVERSED Probable butyrate kinase OS=Bacillus cereus (st	BACC4	0
55	0	0,05	2,452000044	2,452000044	0	RRRRRsp|A7GSI3|BUK_BACCN	REVERSED Probable butyrate kinase OS=Bacillus cytotoxicu	BACCN	0

N	Ctrl_CSVII_1	Total	%Cov	%Cov(50)	%Cov(95)	Accession	Name	Species	Peptides(95%)
1	89,54	89,54	71,11999989	69,88000274	66,46000147	sp|P04264|K2C1_HUMAN	Keratin, type II cytoskeletal 1 OS=Homo sapiens GN=KRT1 PE=	HUMAN	67
2	71,35	71,35	58,73000026	57,19000101	54,44999933	sp|P13645|K1C10_HUMAN	Keratin, type I cytoskeletal 10 OS=Homo sapiens GN=KRT10 PI	HUMAN	58
3	55,57	62,35	80,27999997	63,38000298	56,33999705	sp|P35908|K22E_HUMAN	Keratin, type II cytoskeletal 2 epidermal OS=Homo sapiens GN	HUMAN	38
4	43,8	44,07	72,86999822	68,22000146	57,13999867	sp|P35527|K1C9_HUMAN	Keratin, type I cytoskeletal 9 OS=Homo sapiens GN=KRT9 PE=:	HUMAN	30
6	21,23	21,23	48,9199996	48,9199996	39,82999921	sp|P00761|TRYP_PIG	Trypsin OS=Sus scrofa PE=1 SV=1	PIG	21
7	17,48	28,77	49,11000133	40,95999897	30,84999919	sp|P02538|K2C6A_HUMAN	Keratin, type II cytoskeletal 6A OS=Homo sapiens GN=KRT6A F	HUMAN	16
5	24,87	27,33	56,56999946	43,22000146	36,23000085	sp|P02533|K1C14_HUMAN	Keratin, type I cytoskeletal 14 OS=Homo sapiens GN=KRT14 PI	HUMAN	15
7	0,01	26,8	46,81000113	41,31000042	29,08000052	sp|P48668|K2C6C_HUMAN	Keratin, type II cytoskeletal 6C OS=Homo sapiens GN=KRT6C P	HUMAN	15
12	4,52	21,77	46,09000087	34,67000127	25,15999973	sp|P08779|K1C16_HUMAN	Keratin, type I cytoskeletal 16 OS=Homo sapiens GN=KRT16 PI	HUMAN	11
13	4,18	19,79	39,6600008	25,42000115	16,26999974	sp|P13647|K2C5_HUMAN	Keratin, type II cytoskeletal 5 OS=Homo sapiens GN=KRT5 PE=	HUMAN	10
21	0	9,23	11,8900001	11,8900001	11,36000007	sp|Q6IFZ6|K2C1B_MOUSE	Keratin, type II cytoskeletal 1b OS=Mus musculus GN=Krt77 PI	MOUSE	10
21	0	7,97	15,41000009	13,09999973	12,52000034	sp|Q6IG01|K2C1B_RAT	Keratin, type II cytoskeletal 1b OS=Rattus norvegicus GN=Krt7	RAT	10
13	0	17,79	36,98999882	22,80000001	13,6800006	sp|A5A6M8|K2C5_PANTR	Keratin, type II cytoskeletal 5 OS=Pan troglodytes GN=KRT5 PE	PANTR	9
16	4	11,21	26,15999877	15,51000029	13,88999969	sp|Q04695|K1C17_HUMAN	Keratin, type I cytoskeletal 17 OS=Homo sapiens GN=KRT17 PI	HUMAN	6
16	0	11,21	24,31000024	15,51000029	13,88999969	sp|A5A6M0|K1C17_PANTR	Keratin, type I cytoskeletal 17 OS=Pan troglodytes GN=KRT17	PANTR	6
8	10,34	10,34	71,00999951	69,56999898	55,07000089	sp|P15252|REF_HEVBR	Rubber elongation factor protein OS=Hevea brasiliensis PE=1 !	HEVBR	5
9	10	10	70,96999884	70,96999884	70,96999884	sp|P05109|S10A8_HUMAN	Protein S100-A8 OS=Homo sapiens GN=S100A8 PE=1 SV=1	HUMAN	5
10	7,8	7,8	37,72000074	37,72000074	37,72000074	sp|P06702|S10A9_HUMAN	Protein S100-A9 OS=Homo sapiens GN=S100A9 PE=1 SV=1	HUMAN	5
16	0	9,19	20,09000033	13,62999976	9,46900025	sp|Q9QWL7|K1C17_MOUSE	Keratin, type I cytoskeletal 17 OS=Mus musculus GN=Krt17 PE	MOUSE	5
16	0	9,19	20,09000033	13,62999976	9,46900025	sp|Q6IFU8|K1C17_RAT	Keratin, type I cytoskeletal 17 OS=Rattus norvegicus GN=Krt17	RAT	5
16	0	9,04	25,61999857	14,66999948	10,94999984	sp|Q61781|K1C14_MOUSE	Keratin, type I cytoskeletal 14 OS=Mus musculus GN=Krt14 PE	MOUSE	5
21	2,17	6,69	16,77999943	11,06999964	7,43900016	sp|Q7Z794|K2C1B_HUMAN	Keratin, type II cytoskeletal 1b OS=Homo sapiens GN=KRT77 P	HUMAN	5
11	5,68	5,68	12,38999963	7,722000033	6,005999818	sp|Q02413|DSG1_HUMAN	Desmoglein-1 OS=Homo sapiens GN=DSG1 PE=1 SV=2	HUMAN	4
16	0	5,03	10,50999984	8,276999742	6,711000204	sp|Q6IFV3|K1C15_RAT	Keratin, type I cytoskeletal 15 OS=Rattus norvegicus GN=Krt15	RAT	3
17	3,65	3,65	56,36000037	56,36000037	56,36000037	sp|P81605|DCD_HUMAN	Dermcidin OS=Homo sapiens GN=DCD PE=1 SV=2	HUMAN	3
25	2,01	4,61	16,92000031	5,000000075	4,61499989	sp|Q8N1N4|K2C78_HUMAN	Keratin, type II cytoskeletal 78 OS=Homo sapiens GN=KRT78 P	HUMAN	3
14	4,17	4,18	17,44000018	6,735999882	5,699000135	sp|Q5T749|KPRP_HUMAN	Keratinocyte proline-rich protein OS=Homo sapiens GN=KPRP	HUMAN	2
15	4,05	4,05	9,030999988	3,119999915	3,119999915	sp|Q5NVH5|ALBU_PONAB	Serum albumin OS=Pongo abelii GN=ALB PE=2 SV=2	PONAB	2
15	0	4,05	9,030999988	3,119999915	3,119999915	sp|P02768|ALBU_HUMAN	Serum albumin OS=Homo sapiens GN=ALB PE=1 SV=2	HUMAN	2
15	0	4,02	7,666999847	3,16700004	3,16700004	sp|Q28522|ALBU_MACMU	Serum albumin (Fragment) OS=Macaca mulatta GN=ALB PE=2	MACMU	2
15	0	4,02	7,565999776	3,125	3,125	sp|A2V9Z4|ALBU_MACFA	Serum albumin OS=Macaca fascicularis GN=ALB PE=2 SV=1	MACFA	2
18	3,45	3,45	38,35999966	17,81000048	17,81000048	sp|P12273|PIP_HUMAN	Prolactin-inducible protein OS=Homo sapiens GN=PIP PE=1 SV	HUMAN	2
19	2,86	2,86	12,83999979	12,83999979	8,657000214	sp|Q5RAB4|G3P_PONAB	Glyceraldehyde-3-phosphate dehydrogenase OS=Pongo abelii	PONAB	2
19	0	2,86	12,83999979	12,83999979	8,657000214	sp|P04406|G3P_HUMAN	Glyceraldehyde-3-phosphate dehydrogenase OS=Homo sapier	HUMAN	2
31	1,83	1,86	8,071999997	5,601000041	5,601000041	sp|P02769|ALBU_BOVIN	Serum albumin OS=Bos taurus GN=ALB PE=1 SV=4	BOVIN	2
18	0	2	23,28999937	10,96000001	10,96000001	sp|P60989|PIP_PANTR	Prolactin-inducible protein homolog OS=Pan troglodytes GN=I	PANTR	1
20	2,6	2,6	19,05000061	19,05000061	10,11999995	sp|P17314|IAAC3_WHEAT	Alpha-amylase/trypsin inhibitor CM3 OS=Triticum aestivum PI	WHEAT	1
22	2,17	2,17	14,4600004	9,091000259	3,719000146	sp|P31944|CASPE_HUMAN	Caspase-14 OS=Homo sapiens GN=CASP14 PE=1 SV=2	HUMAN	1
23	2,05	2,05	32,78999925	20,9800005	20,9800005	sp|Q8MKD1|UBB_HORSE	Polyubiquitin-B OS=Equus caballus GN=UBB PE=2 SV=3	HORSE	1
23	0	2,05	32,89000094	21,05000019	21,05000019	sp|Q865C5|UBIQ_CAMDR	Ubiquitin OS=Camelus dromedarius PE=3 SV=2	CAMDR	1
23	0	2,05	30,86000085	19,75000054	19,75000054	sp|Q63429|UBC_RAT	Polyubiquitin-C OS=Rattus norvegicus GN=Ubc PE=1 SV=1	RAT	1
23	0	2,05	39,68000114	25,40000081	25,40000081	sp|P84589|UBIQ_LUMTE	Ubiquitin (Fragment) OS=Lumbricus terrestris PE=1 SV=2	LUMTE	1
23	0	2,05	16,03000015	10,26000008	10,26000008	sp|P79781|RS27A_CHICK	Ubiquitin-40S ribosomal protein S27a OS=Gallus gallus GN=RP	CHICK	1
23	0	2,05	19,52999979	12,5	12,5	sp|P68205|RL40_OPHHA	Ubiquitin-60S ribosomal protein L40 OS=Ophiophagus hannah	OPHHA	1
23	0	2,05	16,03000015	10,26000008	10,26000008	sp|P68203|RS27A_SPOFR	Ubiquitin-40S ribosomal protein S27a OS=Spodoptera frugiper	SPOFR	1
23	0	2,05	16,13000035	10,32000035	10,32000035	sp|P68202|RS27A_PLUXY	Ubiquitin-40S ribosomal protein S27a OS=Plutella xylostella PI	PLUXY	1
23	0	2,05	16,03000015	10,26000008	10,26000008	sp|P68200|RS27A_ICTPU	Ubiquitin-40S ribosomal protein S27a OS=Ictalurus punctatus	ICTPU	1
23	0	2,05	32,89000094	21,05000019	21,05000019	sp|P68197|UBIQ_CERCA	Ubiquitin OS=Ceratitis capitata PE=1 SV=1	CERCA	1
23	0	2,05	19,52999979	12,5	12,5	sp|P63053|RL40_PIG	Ubiquitin-60S ribosomal protein L40 OS=Sus scrofa GN=UBA52	PIG	1
23	0	2,05	19,52999979	12,5	12,5	sp|P63052|RL40_FELCA	Ubiquitin-60S ribosomal protein L40 OS=Felis catus GN=UBA5	FELCA	1
23	0	2,05	19,52999979	12,5	12,5	sp|P63050|RL40_CANLF	Ubiquitin-60S ribosomal protein L40 OS=Canis lupus familiaris	CANLF	1
23	0	2,05	19,52999979	12,5	12,5	sp|P63048|RL40_BOVIN	Ubiquitin-60S ribosomal protein L40 OS=Bos taurus GN=UBA5	BOVIN	1
23	0	2,05	16,03000015	10,26000008	10,26000008	sp|P62992|RS27A_BOVIN	Ubiquitin-40S ribosomal protein S27a OS=Bos taurus GN=RPS2	BOVIN	1
23	0	2,05	19,52999979	12,5	12,5	sp|P62987|RL40_HUMAN	Ubiquitin-60S ribosomal protein L40 OS=Homo sapiens GN=UI	HUMAN	1
23	0	2,05	19,52999979	12,5	12,5	sp|P62986|RL40_RAT	Ubiquitin-60S ribosomal protein L40 OS=Rattus norvegicus GN	RAT	1
23	0	2,05	19,52999979	12,5	12,5	sp|P62984|RL40_MOUSE	Ubiquitin-60S ribosomal protein L40 OS=Mus musculus GN=UI	MOUSE	1
23	0	2,05	16,03000015	10,26000008	10,26000008	sp|P62983|RS27A_MOUSE	Ubiquitin-40S ribosomal protein S27a OS=Mus musculus GN=I	MOUSE	1
23	0	2,05	16,03000015	10,26000008	10,26000008	sp|P62982|RS27A_RAT	Ubiquitin-40S ribosomal protein S27a OS=Rattus norvegicus G	RAT	1
23	0	2,05	16,03000015	10,26000008	10,26000008	sp|P62979|RS27A_HUMAN	Ubiquitin-40S ribosomal protein S27a OS=Homo sapiens GN=I	HUMAN	1
23	0	2,05	16,03000015	10,26000008	10,26000008	sp|P62978|RS27A_CAVPO	Ubiquitin-40S ribosomal protein S27a OS=Cavia porcellus GN=	CAVPO	1
23	0	2,05	30,39999902	19,44999993	19,44999993	sp|P62976|UBIQP_CRIGR	Polyubiquitin OS=Cricetulus griseus PE=2 SV=2	CRIGR	1
23	0	2,05	32,89000094	21,05000019	21,05000019	sp|P62975|UBIQ_RABIT	Ubiquitin OS=Oryctolagus cuniculus PE=1 SV=1	RABIT	1
23	0	2,05	35,33000052	19,15999949	19,15999949	sp|P62972|UBIQP_XENLA	Polyubiquitin (Fragment) OS=Xenopus laevis PE=1 SV=2	XENLA	1
23	0	2,05	16,13000035	10,32000035	10,32000035	sp|P29504|RS27A_MANSE	Ubiquitin-40S ribosomal protein S27a OS=Manduca sexta PE=:	MANSE	1
23	0	2,05	19,52999979	12,5	12,5	sp|P18101|RL40_DROME	Ubiquitin-60S ribosomal protein L40 OS=Drosophila melanoga	DROME	1
23	0	2,05	16,03000015	10,26000008	10,26000008	sp|P15357|RS27A_DROME	Ubiquitin-40S ribosomal protein S27a OS=Drosophila melanog	DROME	1
23	0	2,05	32,60999918	20,87000012	20,87000012	sp|P0CH28|UBC_BOVIN	Polyubiquitin-C OS=Bos taurus GN=UBC PE=1 SV=1	BOVIN	1
23	0	2,05	32,76999891	20,97000033	20,97000033	sp|P0CG69|UBIQP_DROME	Polyubiquitin OS=Drosophila melanogaster GN=Ubi-p63E PE=:	DROME	1
23	0	2,05	32,82999992	21,00999951	21,00999951	sp|P0CG68|UBC_PIG	Polyubiquitin-C OS=Sus scrofa GN=UBC PE=2 SV=1	PIG	1
23	0	2,05	32,74999857	20,96000016	20,96000016	sp|P0CG67|UBB_GORGO	Polyubiquitin-B OS=Gorilla gorilla gorilla GN=UBB PE=3 SV=1	GORGO	1
23	0	2,05	32,8399986	21,01999968	21,01999968	sp|P0CG66|UBC_GORGO	Polyubiquitin-C OS=Gorilla gorilla gorilla GN=UBC PE=3 SV=1	GORGO	1
23	0	2,05	32,74999857	20,96000016	20,96000016	sp|P0CG65|UBB_PANTR	Polyubiquitin-B OS=Pan troglodytes GN=UBB PE=3 SV=1	PANTR	1
23	0	2,05	32,85000026	21,01999968	21,01999968	sp|P0CG64|UBC_PANTR	Polyubiquitin-C OS=Pan troglodytes GN=UBC PE=3 SV=1	PANTR	1
23	0	2,05	32,78999925	20,9800005	20,9800005	sp|P0CG62|UBB_CHICK	Polyubiquitin-B OS=Gallus gallus GN=UBB PE=2 SV=1	CHICK	1
23	0	2,05	32,85000026	21,01999968	21,01999968	sp|P0CG61|UBC_PONPY	Polyubiquitin-C OS=Pongo pygmaeus GN=UBC PE=3 SV=1	PONPY	1
23	0	2,05	32,74999857	20,96000016	20,96000016	sp|P0CG60|UBB_PONPY	Polyubiquitin-B OS=Pongo pygmaeus GN=UBB PE=3 SV=1	PONPY	1
23	0	2,05	32,78999925	20,9800005	20,9800005	sp|P0CG55|UBB_SHEEP	Polyubiquitin-B OS=Ovis aries GN=UBB PE=2 SV=1	SHEEP	1
23	0	2,05	32,15000033	20,57999969	20,57999969	sp|P0CG54|UBB_CAVPO	Polyubiquitin-B OS=Cavia porcellus GN=UBB PE=2 SV=1	CAVPO	1
23	0	2,05	32,78999925	20,9800005	20,9800005	sp|P0CG53|UBB_BOVIN	Polyubiquitin-B OS=Bos taurus GN=UBB PE=1 SV=1	BOVIN	1
23	0	2,05	32,78999925	20,9800005	20,9800005	sp|P0CG51|UBB_RAT	Polyubiquitin-B OS=Rattus norvegicus GN=Ubb PE=1 SV=1	RAT	1
23	0	2,05	30,64999878	19,61999983	19,61999983	sp|P0CG50|UBC_MOUSE	Polyubiquitin-C OS=Mus musculus GN=Ubc PE=1 SV=2	MOUSE	1
23	0	2,05	32,78999925	20,9800005	20,9800005	sp|P0CG49|UBB_MOUSE	Polyubiquitin-B OS=Mus musculus GN=Ubb PE=2 SV=1	MOUSE	1
23	0	2,05	32,85000026	21,01999968	21,01999968	sp|P0CG48|UBC_HUMAN	Polyubiquitin-C OS=Homo sapiens GN=UBC PE=1 SV=3	HUMAN	1
23	0	2,05	32,74999857	20,96000016	20,96000016	sp|P0CG47|UBB_HUMAN	Polyubiquitin-B OS=Homo sapiens GN=UBB PE=1 SV=1	HUMAN	1
23	0	2,05	19,52999979	12,5	12,5	sp|P0C276|RL40_SHEEP	Ubiquitin-60S ribosomal protein L40 OS=Ovis aries GN=UBA52	SHEEP	1
23	0	2,05	19,52999979	12,5	12,5	sp|P0C275|RL40_PONPY	Ubiquitin-60S ribosomal protein L40 OS=Pongo pygmaeus GN=	PONPY	1
23	0	2,05	19,52999979	12,5	12,5	sp|P0C273|RL40_MACFA	Ubiquitin-60S ribosomal protein L40 OS=Macaca fascicularis G	MACFA	1
24	2,02	2,02	11,99999973	4,267000034	4,267000034	sp|Q9Y707|ACT2_SUIBO	Actin-2 OS=Suillus bovinus GN=ACT2 PE=2 SV=1	SUIBO	1
24	0	2,02	11,99999973	4,267000034	4,267000034	sp|Q9Y702|ACT1_SCHCO	Actin-1 OS=Schizophyllum commune GN=ACT1 PE=2 SV=1	SCHCO	1
24	0	2,02	11,99999973	4,267000034	4,267000034	sp|Q9UVX4|ACT_COPC7	Actin OS=Coprinopsis cinerea (strain Okayama-7 / 130 / ATCC	COPC7	1
24	0	2,02	11,96999997	4,255000129	4,255000129	sp|Q964E3|ACTC_BIOAL	Actin, cytoplasmic OS=Biomphalaria alexandrina PE=3 SV=1	BIOAL	1
24	0	2,02	11,96999997	4,255000129	4,255000129	sp|Q964E2|ACTC_BIOPF	Actin, cytoplasmic OS=Biomphalaria pfeifferi PE=3 SV=1	BIOPF	1
24	0	2,02	11,96999997	4,255000129	4,255000129	sp|Q964E1|ACTC_BIOOB	Actin, cytoplasmic OS=Biomphalaria obstructa PE=3 SV=1	BIOOB	1
24	0	2,02	11,96999997	4,255000129	4,255000129	sp|Q964E0|ACTC_BIOTE	Actin, cytoplasmic OS=Biomphalaria tenagophila PE=3 SV=1	BIOTE	1
24	0	2,02	11,96999997	4,255000129	4,255000129	sp|Q964D9|ACTC_PLATR	Actin, cytoplasmic OS=Planorbella trivolvis PE=3 SV=1	PLATR	1
24	0	2,02	11,99999973	4,267000034	4,267000034	sp|Q93131|ACTC_BRAFL	Actin, cytoplasmic OS=Branchiostoma floridae PE=2 SV=1	BRAFL	1
24	0	2,02	11,99999973	4,267000034	4,267000034	sp|Q93129|ACTC_BRABE	Actin, cytoplasmic OS=Branchiostoma belcheri PE=2 SV=1	BRABE	1
24	0	2,02	11,99999973	4,267000034	4,267000034	sp|Q91ZK5|ACTB_SIGHI	Actin, cytoplasmic 1 OS=Sigmodon hispidus GN=ACTB PE=2 SV	SIGHI	1
24	0	2,02	11,99999973	4,267000034	4,267000034	sp|Q8JJB8|ACTG_TRISC	Actin, cytoplasmic 2 OS=Triakis scyllium GN=actg1 PE=2 SV=1	TRISC	1
24	0	2,02	11,99999973	4,267000034	4,267000034	sp|Q7ZVI7|ACTB1_DANRE	Actin, cytoplasmic 1 OS=Danio rerio GN=actba PE=2 SV=2	DANRE	1

24	0	2,02	11,99999973	4,267000034	4,267000034	sp|Q7ZVF9|ACTB2_DANRE	Actin, cytoplasmic 2 OS=Danio rerio GN=actbb PE=2 SV=2	DANRE	1
24	0	2,02	11,99999973	4,267000034	4,267000034	sp|Q76N69|ACTB_CHLAE	Actin, cytoplasmic 1 OS=Chlorocebus aethiops GN=ACTB PE=2	CHLAE	1
24	0	2,02	11,99999973	4,267000034	4,267000034	sp|Q71FK5|ACTB_CAVPO	Actin, cytoplasmic 1 OS=Cavia porcellus GN=ACTB PE=2 SV=1	CAVPO	1
24	0	2,02	11,99999973	4,267000034	4,267000034	sp|Q711N9|ACTB_MESAU	Actin, cytoplasmic 1 OS=Mesocricetus auratus GN=ACTB PE=1	MESAU	1
24	0	2,02	11,99999973	4,267000034	4,267000034	sp|Q6QAQ1|ACTB_PIG	Actin, cytoplasmic 1 OS=Sus scrofa GN=ACTB PE=1 SV=2	PIG	1
24	0	2,02	11,99999973	4,267000034	4,267000034	sp|Q6P378|ACTG_XENTR	Actin, cytoplasmic 2 OS=Xenopus tropicalis GN=actg1 PE=2 SV	XENTR	1
24	0	2,02	11,99999973	4,267000034	4,267000034	sp|Q6NVA9|ACTB_XENTR	Actin, cytoplasmic 1 OS=Xenopus tropicalis GN=actb PE=2 SV=	XENTR	1
24	0	2,02	11,99999973	4,267000034	4,267000034	sp|Q5ZMQ2|ACTG_CHICK	Actin, cytoplasmic 2 OS=Gallus gallus GN=ACTG1 PE=1 SV=1	CHICK	1
24	0	2,02	11,99999973	4,267000034	4,267000034	sp|Q5R6G0|ACTB_PONAB	Actin, cytoplasmic 1 OS=Pongo abelii GN=ACTB PE=2 SV=1	PONAB	1
24	0	2,02	11,99999973	4,267000034	4,267000034	sp|Q5R1X3|ACTB_PANTR	Actin, cytoplasmic 1 OS=Pan troglodytes GN=ACTB PE=2 SV=1	PANTR	1
24	0	2,02	11,99999973	4,267000034	4,267000034	sp|Q5JAK2|ACTG_PELLE	Actin, cytoplasmic 2 OS=Pelophylax lessonae GN=actg1 PE=2 S	PELLE	1
24	0	2,02	11,96999997	4,255000129	4,255000129	sp|Q553U6|ACT22_DICDI	Putative actin-22 OS=Dictyostelium discoideum GN=act22 PE=	DICDI	1
24	0	2,02	11,96999997	4,255000129	4,255000129	sp|Q54GX7|ACT10_DICDI	Actin-10 OS=Dictyostelium discoideum GN=act10 PE=1 SV=1	DICDI	1
24	0	2,02	11,99999973	4,267000034	4,267000034	sp|Q4R561|ACTB_MACFA	Actin, cytoplasmic 1 OS=Macaca fascicularis GN=ACTB PE=2 S\	MACFA	1
24	0	2,02	11,99999973	4,267000034	4,267000034	sp|Q4L0Y2|ACTB_SPECI	Actin, cytoplasmic 1 OS=Spermophilus citellus GN=ACTB PE=2	SPECI	1
24	0	2,02	11,96999997	4,255000129	4,255000129	sp|Q26065|ACT_PLAMG	Actin, adductor muscle OS=Placopecten magellanicus PE=2 SV	PLAMG	1
24	0	2,02	11,90000027	4,233000055	4,233000055	sp|Q25472|ACT2_MOLOC	Actin, muscle-type OS=Molgula oculata PE=3 SV=1	MOLOC	1
24	0	2,02	11,96999997	4,255000129	4,255000129	sp|Q25010|ACT3A_HELAM	Actin, cytoplasmic A3a OS=Helicoverpa armigera GN=actA3a P	HELAM	1
24	0	2,02	11,96999997	4,255000129	4,255000129	sp|Q07903|ACTC_STRPU	Actin, cytoskeletal 2A OS=Strongylocentrotus purpuratus GN=	STRPU	1
24	0	2,02	11,99999973	4,267000034	4,267000034	sp|Q00215|ACTC_STYPL	Actin, cytoplasmic OS=Styela plicata PE=3 SV=1	STYPL	1
24	0	2,02	11,96999997	4,255000129	4,255000129	sp|P92182|ACT1_LUMTE	Actin-1 OS=Lumbricus terrestris GN=ACT1 PE=2 SV=1	LUMTE	1
24	0	2,02	11,96999997	4,255000129	4,255000129	sp|P92179|ACTC_BIOGL	Actin, cytoplasmic OS=Biomphalaria glabrata PE=2 SV=2	BIOGL	1
24	0	2,02	11,96999997	4,255000129	4,255000129	sp|P92176|ACT2_LUMTE	Actin-2 OS=Lumbricus terrestris GN=ACT2 PE=2 SV=1	LUMTE	1
24	0	2,02	12,09999993	4,301000014	4,301000014	sp|P91754|ACT_LUMRU	Actin (Fragment) OS=Lumbricus rubellus PE=2 SV=1	LUMRU	1
24	0	2,02	11,96999997	4,255000129	4,255000129	sp|P90689|ACT_BRUMA	Actin OS=Brugia malayi PE=1 SV=1	BRUMA	1
24	0	2,02	12,47000024	4,431999847	4,431999847	sp|P84856|ACTB_CHLPG	Actin, cytoplasmic 1 OS=Chlorocebus pygerythrus GN=ACTB P	CHLPG	1
24	0	2,02	11,99999973	4,267000034	4,267000034	sp|P84336|ACTB_CAMDR	Actin, cytoplasmic 1 OS=Camelus dromedarius GN=ACTB PE=1	CAMDR	1
24	0	2,02	11,96999997	4,255000129	4,255000129	sp|P84185|ACT5C_ANOGA	Actin-5C OS=Anopheles gambiae GN=Act5C PE=2 SV=1	ANOGA	1
24	0	2,02	11,96999997	4,255000129	4,255000129	sp|P84184|ACT3B_HELAM	Actin-A3b, cytoplasmic OS=Helicoverpa armigera GN=actA3b I	HELAM	1
24	0	2,02	11,96999997	4,255000129	4,255000129	sp|P84183|ACT4_BOMMO	Actin, cytoplasmic A4 OS=Bombyx mori GN=A4 PE=2 SV=1	BOMMO	1
24	0	2,02	11,96999997	4,255000129	4,255000129	sp|P83969|ACT1_BACDO	Actin, indirect flight muscle OS=Bactrocera dorsalis PE=3 SV=1	BACDO	1
24	0	2,02	11,96999997	4,255000129	4,255000129	sp|P83968|ACT6_DROSI	Actin, indirect flight muscle OS=Drosophila simulans GN=Act8	DROSI	1
24	0	2,02	11,96999997	4,255000129	4,255000129	sp|P83967|ACT6_DROME	Actin, indirect flight muscle OS=Drosophila melanogaster GN=	DROME	1
24	0	2,02	11,99999973	4,267000034	4,267000034	sp|P83751|ACTB_CTEID	Actin, cytoplasmic 1 OS=Ctenopharyngodon idella GN=actb PE	CTEID	1
24	0	2,02	11,99999973	4,267000034	4,267000034	sp|P83750|ACTB_CYPCA	Actin, cytoplasmic 1 OS=Cyprinus carpio GN=actb PE=3 SV=1	CYPCA	1
24	0	2,02	11,96999997	4,255000129	4,255000129	sp|P69005|ACTD_STRPU	Actin, cytoskeletal 2B OS=Strongylocentrotus purpuratus GN=	STRPU	1
24	0	2,02	11,96999997	4,255000129	4,255000129	sp|P69004|ACT2_STRFN	Actin-15B OS=Strongylocentrotus franciscanus PE=2 SV=1	STRFN	1
24	0	2,02	11,96999997	4,255000129	4,255000129	sp|P69003|ACT1_HELTB	Actin CyI, cytoplasmic OS=Heliocidaris tuberculata PE=3 SV=1	HELTB	1
24	0	2,02	11,96999997	4,255000129	4,255000129	sp|P69002|ACT1_HELER	Actin CyI, cytoplasmic OS=Heliocidaris erythrogramma PE=3 S\	HELER	1
24	0	2,02	11,96999997	4,255000129	4,255000129	sp|P68556|ACT1_DIPDE	Actin-1/4 OS=Diphyllobothrium dendriticum GN=ACT1 PE=2 S	DIPDE	1
24	0	2,02	11,96999997	4,255000129	4,255000129	sp|P68555|ACT_TAESO	Actin OS=Taenia solium GN=ACT1 PE=3 SV=1	TAESO	1
24	0	2,02	11,99999973	4,267000034	4,267000034	sp|P68143|ACTB_OREMO	Actin, cytoplasmic 1 OS=Oreochromis mossambicus GN=actb I	OREMO	1
24	0	2,02	11,99999973	4,267000034	4,267000034	sp|P68142|ACTB1_TAKRU	Actin, cytoplasmic 1 OS=Takifugu rubripes GN=actba PE=2 SV=	TAKRU	1
24	0	2,02	11,99999973	4,267000034	4,267000034	sp|P63261|ACTG_HUMAN	Actin, cytoplasmic 2 OS=Homo sapiens GN=ACTG1 PE=1 SV=1	HUMAN	1
24	0	2,02	11,99999973	4,267000034	4,267000034	sp|P63260|ACTG_MOUSE	Actin, cytoplasmic 2 OS=Mus musculus GN=Actg1 PE=1 SV=1	MOUSE	1
24	0	2,02	11,99999973	4,267000034	4,267000034	sp|P63259|ACTG_RAT	Actin, cytoplasmic 2 OS=Rattus norvegicus GN=Actg1 PE=1 SV	RAT	1
24	0	2,02	11,99999973	4,267000034	4,267000034	sp|P63258|ACTG_BOVIN	Actin, cytoplasmic 2 OS=Bos taurus GN=ACTG1 PE=1 SV=1	BOVIN	1
24	0	2,02	11,99999973	4,267000034	4,267000034	sp|P63257|ACTG_TRIVU	Actin, cytoplasmic 2 OS=Trichosurus vulpecula GN=ACTG1 PE=	TRIVU	1
24	0	2,02	11,99999973	4,267000034	4,267000034	sp|P63256|ACTG_ANSAN	Actin, cytoplasmic 2 OS=Anser anser anser GN=ACTG1 PE=2 S\	ANSAN	1
24	0	2,02	11,99999973	4,267000034	4,267000034	sp|P60713|ACTB_SHEEP	Actin, cytoplasmic 1 OS=Ovis aries GN=ACTB PE=2 SV=1	SHEEP	1
24	0	2,02	11,99999973	4,267000034	4,267000034	sp|P60712|ACTB_BOVIN	Actin, cytoplasmic 1 OS=Bos taurus GN=ACTB PE=1 SV=1	BOVIN	1
24	0	2,02	11,99999973	4,267000034	4,267000034	sp|P60711|ACTB_RAT	Actin, cytoplasmic 1 OS=Rattus norvegicus GN=Actb PE=1 SV=	RAT	1
24	0	2,02	11,99999973	4,267000034	4,267000034	sp|P60710|ACTB_MOUSE	Actin, cytoplasmic 1 OS=Mus musculus GN=Actb PE=1 SV=1	MOUSE	1
24	0	2,02	11,99999973	4,267000034	4,267000034	sp|P60709|ACTB_HUMAN	Actin, cytoplasmic 1 OS=Homo sapiens GN=ACTB PE=1 SV=1	HUMAN	1
24	0	2,02	11,99999973	4,267000034	4,267000034	sp|P60708|ACTB_HORSE	Actin, cytoplasmic 1 OS=Equus caballus GN=ACTB PE=2 SV=1	HORSE	1
24	0	2,02	11,99999973	4,267000034	4,267000034	sp|P60707|ACTB_TRIVU	Actin, cytoplasmic 1 OS=Trichosurus vulpecula GN=ACTB PE=2	TRIVU	1
24	0	2,02	11,99999973	4,267000034	4,267000034	sp|P60706|ACTB_CHICK	Actin, cytoplasmic 1 OS=Gallus gallus GN=ACTB PE=1 SV=1	CHICK	1
24	0	2,02	11,99999973	4,267000034	4,267000034	sp|P53689|ACT_PHARH	Actin OS=Phaffia rhodozyma PE=3 SV=1	PHARH	1
24	0	2,02	11,96999997	4,255000129	4,255000129	sp|P53506|ACT8_XENLA	Actin, cytoplasmic type 8 OS=Xenopus laevis PE=3 SV=1	XENLA	1
24	0	2,02	11,96999997	4,255000129	4,255000129	sp|P53505|ACT5_XENLA	Actin, cytoplasmic type 5 OS=Xenopus laevis PE=3 SV=1	XENLA	1
24	0	2,02	11,96999997	4,255000129	4,255000129	sp|P53501|ACT3_DROME	Actin-57B OS=Drosophila melanogaster GN=Act57B PE=1 SV=:	DROME	1
24	0	2,02	11,99999973	4,267000034	4,267000034	sp|P53486|ACTB3_TAKRU	Actin, cytoplasmic 3 OS=Takifugu rubripes GN=actbc PE=2 SV=	TAKRU	1
24	0	2,02	11,99999973	4,267000034	4,267000034	sp|P53485|ACTB2_TAKRU	Actin, cytoplasmic 2 OS=Takifugu rubripes GN=actbb PE=3 SV=	TAKRU	1
24	0	2,02	11,96999997	4,255000129	4,255000129	sp|P53478|ACT5_CHICK	Actin, cytoplasmic type 5 OS=Gallus gallus PE=3 SV=1	CHICK	1
24	0	2,02	11,96999997	4,255000129	4,255000129	sp|P53474|ACTE_STRPU	Actin, cytoskeletal 3A OS=Strongylocentrotus purpuratus GN=	STRPU	1
24	0	2,02	11,96999997	4,255000129	4,255000129	sp|P53473|ACTB_STRPU	Actin, cytoskeletal 1B OS=Strongylocentrotus purpuratus GN=	STRPU	1
24	0	2,02	11,96999997	4,255000129	4,255000129	sp|P53472|ACTA_STRPU	Actin, cytoskeletal 1A OS=Strongylocentrotus purpuratus GN=	STRPU	1
24	0	2,02	11,96999997	4,255000129	4,255000129	sp|P53471|ACT2_SCHMA	Actin-2 OS=Schistosoma mansoni PE=2 SV=1	SCHMA	1
24	0	2,02	11,96999997	4,255000129	4,255000129	sp|P53470|ACT1_SCHMA	Actin-1 OS=Schistosoma mansoni PE=2 SV=1	SCHMA	1
24	0	2,02	11,96999997	4,255000129	4,255000129	sp|P53466|ACT2_LYTPI	Actin, cytoskeletal 2 OS=Lytechinus pictus PE=2 SV=1	LYTPI	1
24	0	2,02	11,96999997	4,255000129	4,255000129	sp|P53465|ACT1_LYTPI	Actin, cytoskeletal 1 OS=Lytechinus pictus PE=2 SV=1	LYTPI	1
24	0	2,02	11,96999997	4,255000129	4,255000129	sp|P53464|ACTM_HELTB	Actin, cytoskeletal OS=Heliocidaris tuberculata PE=3 SV=1	HELTB	1
24	0	2,02	11,96999997	4,255000129	4,255000129	sp|P53463|ACTM_HELER	Actin, cytoskeletal OS=Heliocidaris erythrogramma PE=3 SV=1	HELER	1
24	0	2,02	12,1299997	4,312999919	4,312999919	sp|P53458|ACT5_DIPDE	Actin-5 (Fragment) OS=Diphyllobothrium dendriticum GN=ACT	DIPDE	1
24	0	2,02	11,96999997	4,255000129	4,255000129	sp|P53456|ACT2_DIPDE	Actin-2 OS=Diphyllobothrium dendriticum GN=ACT2 PE=2 SV=	DIPDE	1
24	0	2,02	11,96999997	4,255000129	4,255000129	sp|P49871|ACT_MANSE	Actin, muscle OS=Manduca sexta PE=2 SV=1	MANSE	1
24	0	2,02	11,96999997	4,255000129	4,255000129	sp|P49128|ACT1_AEDAE	Actin-1 OS=Aedes aegypti GN=ACT-1 PE=2 SV=2	AEDAE	1
24	0	2,02	11,99999973	4,267000034	4,267000034	sp|P48975|ACTB_CRIGR	Actin, cytoplasmic 1 OS=Cricetulus griseus GN=ACTB PE=3 SV=	CRIGR	1
24	0	2,02	11,99999973	4,267000034	4,267000034	sp|P48465|ACT_CRYNH	Actin OS=Cryptococcus neoformans var. grubii serotype A (str	CRYNH	1
24	0	2,02	11,96999997	4,255000129	4,255000129	sp|P45887|ACT5_BACDO	Actin-5, muscle-specific OS=Bactrocera dorsalis PE=2 SV=1	BACDO	1
24	0	2,02	11,96999997	4,255000129	4,255000129	sp|P45886|ACT3_BACDO	Actin-3, muscle-specific OS=Bactrocera dorsalis PE=2 SV=1	BACDO	1
24	0	2,02	11,96999997	4,255000129	4,255000129	sp|P45885|ACT2_BACDO	Actin-2, muscle-specific OS=Bactrocera dorsalis PE=2 SV=1	BACDO	1
24	0	2,02	11,96999997	4,255000129	4,255000129	sp|P41341|ACTY_LIMPO	Actin-11 OS=Limulus polyphemus PE=2 SV=1	LIMPO	1
24	0	2,02	11,96999997	4,255000129	4,255000129	sp|P41340|ACT3_LIMPO	Actin-3 OS=Limulus polyphemus PE=1 SV=1	LIMPO	1
24	0	2,02	11,96999997	4,255000129	4,255000129	sp|P41339|ACTA_LIMPO	Actin, acrosomal process isoform OS=Limulus polyphemus PE=	LIMPO	1
24	0	2,02	11,96999997	4,255000129	4,255000129	sp|P41113|ACT3_PODCA	Actin-3 OS=Podocoryna carnea GN=ACT3 PE=3 SV=1	PODCA	1
24	0	2,02	11,96999997	4,255000129	4,255000129	sp|P41112|ACT1_PODCA	Actin-1/2 OS=Podocoryna carnea GN=ACTIA PE=2 SV=1	PODCA	1
24	0	2,02	11,96999997	4,255000129	4,255000129	sp|P18603|ACT4_ARTSX	Actin, clone 403 OS=Artemia sp. PE=2 SV=1	ARTSX	1
24	0	2,02	11,96999997	4,255000129	4,255000129	sp|P18499|ACTF_STRPU	Actin, cytoskeletal 3B OS=Strongylocentrotus purpuratus GN=	STRPU	1
24	0	2,02	11,96999997	4,255000129	4,255000129	sp|P17126|ACT_HYDVU	Actin, non-muscle 6.2 OS=Hydra vulgaris PE=3 SV=1	HYDVU	1
24	0	2,02	11,96999997	4,255000129	4,255000129	sp|P15475|ACTB_XENBO	Actin, cytoplasmic 1 OS=Xenopus borealis GN=actb PE=3 SV=1	XENBO	1
24	0	2,02	11,96999997	4,255000129	4,255000129	sp|P12717|ACTM_PISOC	Actin, muscle OS=Pisaster ochraceus PE=3 SV=1	PISOC	1
24	0	2,02	11,96999997	4,255000129	4,255000129	sp|P12716|ACTC_PISOC	Actin, cytoplasmic OS=Pisaster ochraceus PE=3 SV=1	PISOC	1
24	0	2,02	12,03000024	4,278000072	4,278000072	sp|P12431|ACTM_STRPU	Actin, muscle OS=Strongylocentrotus purpuratus PE=3 SV=1	STRPU	1
24	0	2,02	11,96999997	4,255000129	4,255000129	sp|P10990|ACT1_STRFN	Actin-15A OS=Strongylocentrotus franciscanus PE=3 SV=1	STRFN	1
24	0	2,02	11,96999997	4,255000129	4,255000129	sp|P10987|ACT1_DROME	Actin-5C OS=Drosophila melanogaster GN=Act5C PE=1 SV=4	DROME	1
24	0	2,02	11,96999997	4,255000129	4,255000129	sp|P10981|ACT5_DROME	Actin-87E OS=Drosophila melanogaster GN=Act87E PE=1 SV=1	DROME	1
24	0	2,02	11,96999997	4,255000129	4,255000129	sp|P07836|ACT1_BOMMO	Actin, muscle-type A1 OS=Bombyx mori PE=3 SV=1	BOMMO	1
24	0	2,02	11,96999997	4,255000129	4,255000129	sp|P07830|ACT1_DICDI	Major actin OS=Dictyostelium discoideum GN=act1 PE=1 SV=2	DICDI	1
24	0	2,02	11,96999997	4,255000129	4,255000129	sp|P07829|ACT3_DICDI	Actin-3 OS=Dictyostelium discoideum GN=act3 PE=3 SV=3	DICDI	1
24	0	2,02	11,96999997	4,255000129	4,255000129	sp|P04829|ACT3_BOMMO	Actin, cytoplasmic A3 OS=Bombyx mori PE=3 SV=3	BOMMO	1

24	0	2,02	11,99999973	4,267000034	4,267000034	sp|P02578|ACT1_ACACA	Actin-1 OS=Acanthamoeba castellanii PE=1 SV=1	ACACA	1
24	0	2,02	11,96999997	4,255000129	4,255000129	sp|P02576|ACTA_PHYPO	Actin, plasmodial isoform OS=Physarum polycephalum GN=AR	PHYPO	1
24	0	2,02	11,96999997	4,255000129	4,255000129	sp|P02574|ACT4_DROME	Actin, larval muscle OS=Drosophila melanogaster GN=Act79B	DROME	1
24	0	2,02	11,96999997	4,255000129	4,255000129	sp|P02572|ACT2_DROME	Actin-42A OS=Drosophila melanogaster GN=Act42A PE=1 SV=:	DROME	1
24	0	2,02	11,99999973	4,267000034	4,267000034	sp|O93400|ACTB_XENLA	Actin, cytoplasmic 1 OS=Xenopus laevis GN=actb PE=2 SV=1	XENLA	1
24	0	2,02	11,94000021	4,244000092	4,244000092	sp|O65316|ACT_MESVI	Actin OS=Mesostigma viride PE=3 SV=1	MESVI	1
24	0	2,02	11,99999973	4,267000034	4,267000034	sp|O42161|ACTB_SALSA	Actin, cytoplasmic 1 OS=Salmo salar GN=actb PE=2 SV=1	SALSA	1
24	0	2,02	11,99999973	4,267000034	4,267000034	sp|O18840|ACTB_CANLF	Actin, cytoplasmic 1 OS=Canis lupus familiaris GN=ACTB PE=2	CANLF	1
24	0	2,02	11,96999997	4,255000129	4,255000129	sp|O18500|ACT2_SACKO	Actin-2 OS=Saccoglossus kowalevskii PE=2 SV=1	SACKO	1
24	0	2,02	11,96999997	4,255000129	4,255000129	sp|O17320|ACT_CRAGI	Actin OS=Crassostrea gigas PE=2 SV=1	CRAGI	1
24	0	2,02	11,96999997	4,255000129	4,255000129	sp|O16808|ACT_MAYDE	Actin OS=Mayetiola destructor PE=2 SV=1	MAYDE	1
24	0	2,02	11,99999973	4,267000034	4,267000034	sp|A2BDB0|ACTG_XENLA	Actin, cytoplasmic 2 OS=Xenopus laevis GN=actg1 PE=2 SV=1	XENLA	1
24	0	2,02	12,78000027	6,01500012	6,01500012	sp|Q92193|ACT_CRAVI	Actin (Fragment) OS=Crassostrea virginica PE=2 SV=1	CRAVI	1
24	0	2,02	9,497000277	4,469000176	4,469000176	sp|Q55EU6|ACT23_DICDI	Putative actin-23 OS=Dictyostelium discoideum GN=act23 PE=	DICDI	1
24	0	2,02	10,99999994	5,178000033	5,178000033	sp|Q03342|ACT3_ECHGR	Actin-3 (Fragment) OS=Echinococcus granulosus GN=ACTIII PE	ECHGR	1
24	0	2,02	8,970999718	4,222000018	4,222000018	sp|Q00214|ACTM_STYPL	Actin, muscle OS=Styela plicata PE=3 SV=1	STYPL	1
24	0	2,02	9,066999704	4,267000034	4,267000034	sp|P50138|ACT_PUCGR	Actin OS=Puccinia graminis PE=3 SV=1	PUCGR	1
24	0	2,02	9,042999893	4,255000129	4,255000129	sp|O18499|ACT1_SACKO	Actin-1 OS=Saccoglossus kowalevskii PE=2 SV=1	SACKO	1
24	0	2,01	11,99999973	4,267000034	4,267000034	sp|Q9UVW9|ACTG_ACRCH	Actin, gamma OS=Acremonium chrysogenum GN=ACT PE=3 S\	ACRCH	1
24	0	2,01	11,99999973	4,267000034	4,267000034	sp|Q8X119|ACT_EXODE	Actin OS=Exophiala dermatitidis PE=3 SV=1	EXODE	1
24	0	2,01	11,99999973	4,267000034	4,267000034	sp|Q6TCF2|ACT_GAEGA	Actin OS=Gaeumannomyces graminis var. avenae GN=ACT PE=	GAEGA	1
24	0	2,01	11,99999973	4,267000034	4,267000034	sp|P78711|ACT_NEUCR	Actin OS=Neurospora crassa (strain ATCC 24698 / 74-OR23-1A	NEUCR	1
24	0	2,01	11,99999973	4,267000034	4,267000034	sp|P53455|ACT_AJECG	Actin OS=Ajellomyces capsulatus (strain G186AR / H82 / ATCC	AJECG	1
24	0	2,01	11,99999973	4,267000034	4,267000034	sp|P20359|ACTG_EMENI	Actin, gamma OS=Emericella nidulans (strain FGSC A4 / ATCC :	EMENI	1
24	0	2,01	11,99999973	4,267000034	4,267000034	sp|P10365|ACT_THELA	Actin OS=Thermomyces lanuginosus PE=3 SV=1	THELA	1
24	0	2,01	11,99999973	4,267000034	4,267000034	sp|O13419|ACT_BOTFU	Actin OS=Botryotinia fuckeliana GN=actA PE=3 SV=1	BOTFU	1
24	0	2,01	9,042999893	4,255000129	4,255000129	sp|Q8BFZ3|ACTBL_MOUSE	Beta-actin-like protein 2 OS=Mus musculus GN=Actbl2 PE=1 S\	MOUSE	1
24	0	2,01	9,042999893	4,255000129	4,255000129	sp|Q562R1|ACTBL_HUMAN	Beta-actin-like protein 2 OS=Homo sapiens GN=ACTBL2 PE=1 S	HUMAN	1
24	0	2	7,180999964	4,255000129	4,255000129	sp|Q9UVZ8|ACT_CANDC	Actin OS=Candida dubliniensis (strain CD36 / ATCC MYA-646 /	CANDC	1
24	0	2	7,19999969	4,267000034	4,267000034	sp|Q9URS0|ACTG_PENCH	Actin, gamma OS=Penicillium chrysogenum GN=ACT PE=3 SV=	PENCH	1
24	0	2	7,19999969	4,267000034	4,267000034	sp|Q9BYX7|ACTBM_HUMAN	Putative beta-actin-like protein 3 OS=Homo sapiens GN=POTE	HUMAN	1
24	0	2	7,162000239	4,244000092	4,244000092	sp|Q98972|ACTS_ORYLA	Actin, alpha skeletal muscle OS=Oryzias latipes GN=acta1 PE=2	ORYLA	1
24	0	2	7,142999768	4,233000055	4,233000055	sp|Q93132|ACTM_BRAFL	Actin, muscle OS=Branchiostoma floridae PE=2 SV=1	BRAFL	1
24	0	2	7,124000043	4,222000018	4,222000018	sp|Q93130|ACTM_BRABE	Actin, muscle OS=Branchiostoma belcheri PE=2 SV=1	BRABE	1
24	0	2	7,162000239	4,244000092	4,244000092	sp|Q90X97|ACTS_ATRMM	Actin, alpha skeletal muscle OS=Atractaspis microlepidota mic	ATRMM	1
24	0	2	7,180999964	4,255000129	4,255000129	sp|Q75D00|ACT_ASHGO	Actin OS=Ashbya gossypii (strain ATCC 10895 / CBS 109.51 / F	ASHGO	1
24	0	2	2,511999942	1,487999968	1,487999968	sp|Q6S8J3|POTEE_HUMAN	POTE ankyrin domain family member E OS=Homo sapiens GN=	HUMAN	1
24	0	2	7,162000239	4,244000092	4,244000092	sp|Q6P8G3|ACT3_XENTR	Actin, alpha sarcomeric/skeletal OS=Xenopus tropicalis GN=ac	XENTR	1
24	0	2	7,162000239	4,244000092	4,244000092	sp|Q6P640|ACTC_XENTR	Actin, alpha cardiac muscle 1 OS=Xenopus tropicalis GN=actc1	XENTR	1
24	0	2	7,162000239	4,244000092	4,244000092	sp|Q5R9Q5|ACTS_PONAB	Actin, alpha skeletal muscle OS=Pongo abelii GN=ACTA1 PE=2	PONAB	1
24	0	2	7,180999964	4,255000129	4,255000129	sp|Q5E9B5|ACTH_BOVIN	Actin, gamma-enteric smooth muscle OS=Bos taurus GN=ACT(	BOVIN	1
24	0	2	7,162000239	4,244000092	4,244000092	sp|Q3ZC07|ACTC_BOVIN	Actin, alpha cardiac muscle 1 OS=Bos taurus GN=ACTC1 PE=2 :	BOVIN	1
24	0	2	7,19999969	4,267000034	4,267000034	sp|Q0PGG4|ACTB_BOSMU	Actin, cytoplasmic 1 OS=Bos mutus grunniens GN=ACTB PE=2	BOSMU	1
24	0	2	14,05999959	8,332999796	8,332999796	sp|P86700|ACT_CHIOP	Actin, muscle (Fragments) OS=Chionoecetes opilio PE=1 SV=1	CHIOP	1
24	0	2	7,19999969	4,267000034	4,267000034	sp|P79818|ACTB_ORYLA	Actin, cytoplasmic 1 OS=Oryzias latipes GN=actb PE=2 SV=2	ORYLA	1
24	0	2	7,162000239	4,244000092	4,244000092	sp|P68264|ACTS_OREMO	Actin, alpha skeletal muscle OS=Oreochromis mossambicus GI	OREMO	1
24	0	2	7,162000239	4,244000092	4,244000092	sp|P68140|ACTSA_TAKRU	Actin, alpha skeletal muscle A OS=Takifugu rubripes GN=acta1	TAKRU	1
24	0	2	7,162000239	4,244000092	4,244000092	sp|P68139|ACTS_CHICK	Actin, alpha skeletal muscle OS=Gallus gallus GN=ACTA1 PE=1	CHICK	1
24	0	2	7,162000239	4,244000092	4,244000092	sp|P68138|ACTS_BOVIN	Actin, alpha skeletal muscle OS=Bos taurus GN=ACTA1 PE=1 S\	BOVIN	1
24	0	2	7,162000239	4,244000092	4,244000092	sp|P68137|ACTS_PIG	Actin, alpha skeletal muscle OS=Sus scrofa GN=ACTA1 PE=3 SV	PIG	1
24	0	2	7,162000239	4,244000092	4,244000092	sp|P68136|ACTS_RAT	Actin, alpha skeletal muscle OS=Rattus norvegicus GN=Acta1 F	RAT	1
24	0	2	7,162000239	4,244000092	4,244000092	sp|P68135|ACTS_RABIT	Actin, alpha skeletal muscle OS=Oryctolagus cuniculus GN=ACT	RABIT	1
24	0	2	7,162000239	4,244000092	4,244000092	sp|P68134|ACTS_MOUSE	Actin, alpha skeletal muscle OS=Mus musculus GN=Acta1 PE=:	MOUSE	1
24	0	2	7,162000239	4,244000092	4,244000092	sp|P68133|ACTS_HUMAN	Actin, alpha skeletal muscle OS=Homo sapiens GN=ACTA1 PE=	HUMAN	1
24	0	2	7,162000239	4,244000092	4,244000092	sp|P68035|ACTC_RAT	Actin, alpha cardiac muscle 1 OS=Rattus norvegicus GN=Actc1	RAT	1
24	0	2	7,162000239	4,244000092	4,244000092	sp|P68034|ACTC_CHICK	Actin, alpha cardiac muscle 1 OS=Gallus gallus GN=ACTC1 PE=:	CHICK	1
24	0	2	7,162000239	4,244000092	4,244000092	sp|P68033|ACTC_MOUSE	Actin, alpha cardiac muscle 1 OS=Mus musculus GN=Actc1 PE=	MOUSE	1
24	0	2	7,162000239	4,244000092	4,244000092	sp|P68032|ACTC_HUMAN	Actin, alpha cardiac muscle 1 OS=Homo sapiens GN=ACTC1 PE	HUMAN	1
24	0	2	7,180999964	4,255000129	4,255000129	sp|P63270|ACTH_CHICK	Actin, gamma-enteric smooth muscle OS=Gallus gallus GN=AC	CHICK	1
24	0	2	7,180999964	4,255000129	4,255000129	sp|P63269|ACTH_RAT	Actin, gamma-enteric smooth muscle OS=Rattus norvegicus G	RAT	1
24	0	2	7,180999964	4,255000129	4,255000129	sp|P63268|ACTH_MOUSE	Actin, gamma-enteric smooth muscle OS=Mus musculus GN=/	MOUSE	1
24	0	2	7,180999964	4,255000129	4,255000129	sp|P63267|ACTH_HUMAN	Actin, gamma-enteric smooth muscle OS=Homo sapiens GN=/	HUMAN	1
24	0	2	7,162000239	4,244000092	4,244000092	sp|P62740|ACTA_RABIT	Actin, aortic smooth muscle OS=Oryctolagus cuniculus GN=AC	RABIT	1
24	0	2	7,162000239	4,244000092	4,244000092	sp|P62739|ACTA_BOVIN	Actin, aortic smooth muscle OS=Bos taurus GN=ACTA2 PE=1 S	BOVIN	1
24	0	2	7,162000239	4,244000092	4,244000092	sp|P62738|ACTA_RAT	Actin, aortic smooth muscle OS=Rattus norvegicus GN=Acta2 I	RAT	1
24	0	2	7,162000239	4,244000092	4,244000092	sp|P62737|ACTA_MOUSE	Actin, aortic smooth muscle OS=Mus musculus GN=Acta2 PE=	MOUSE	1
24	0	2	7,162000239	4,244000092	4,244000092	sp|P62736|ACTA_HUMAN	Actin, aortic smooth muscle OS=Homo sapiens GN=ACTA2 PE=	HUMAN	1
24	0	2	7,19999969	4,267000034	4,267000034	sp|P60011|ACT_SACBA	Actin OS=Saccharomyces bayanus GN=ACT1 PE=3 SV=1	SACBA	1
24	0	2	7,19999969	4,267000034	4,267000034	sp|P60010|ACT_YEAST	Actin OS=Saccharomyces cerevisiae (strain ATCC 204508 / S28	YEAST	1
24	0	2	7,19999969	4,267000034	4,267000034	sp|P60009|ACT_CANGA	Actin OS=Candida glabrata (strain ATCC 2001 / CBS 138 / JCM	CANGA	1
24	0	2	7,162000239	4,244000092	4,244000092	sp|P53482|ACTSB_TAKRU	Actin, alpha skeletal muscle B OS=Takifugu rubripes GN=acta1	TAKRU	1
24	0	2	7,162000239	4,244000092	4,244000092	sp|P53480|ACTC_TAKRU	Actin, alpha cardiac OS=Takifugu rubripes PE=2 SV=1	TAKRU	1
24	0	2	7,162000239	4,244000092	4,244000092	sp|P53479|ACTS_CYPCA	Actin, alpha skeletal muscle OS=Cyprinus carpio GN=acta1 PE=	CYPCA	1
24	0	2	7,142999768	4,233000055	4,233000055	sp|P53475|ACTN_STYCL	Actin, muscle OS=Styela clava GN=TB12 PE=2 SV=1	STYCL	1
24	0	2	7,142999768	4,233000055	4,233000055	sp|P53467|ACTM_MOLOC	Actin, larval muscle-type OS=Molgula oculata PE=3 SV=1	MOLOC	1
24	0	2	7,162000239	4,244000092	4,244000092	sp|P49055|ACTS_CARAU	Actin, alpha skeletal muscle OS=Carassius auratus GN=acta1 P	CARAU	1
24	0	2	7,19999969	4,267000034	4,267000034	sp|P43239|ACT1_PNECA	Actin-1 OS=Pneumocystis carinii PE=2 SV=1	PNECA	1
24	0	2	7,19999969	4,267000034	4,267000034	sp|P29751|ACTB_RABIT	Actin, cytoplasmic 1 OS=Oryctolagus cuniculus GN=ACTB PE=2	RABIT	1
24	0	2	7,142999768	4,233000055	4,233000055	sp|P27130|ACT2_HALRO	Actin, muscle 2/4/4A OS=Halocynthia roretzi GN=MA2 PE=2 S\	HALRO	1
24	0	2	7,142999768	4,233000055	4,233000055	sp|P26198|ACTM_STYCL	Actin, muscle OS=Styela clava PE=2 SV=1	STYCL	1
24	0	2	7,162000239	4,244000092	4,244000092	sp|P20399|ACT2_XENTR	Actin, alpha cardiac muscle 2 OS=Xenopus tropicalis PE=2 SV=	XENTR	1
24	0	2	7,180999964	4,255000129	4,255000129	sp|P18601|ACT2_ARTSX	Actin, clone 211 OS=Artemia sp. PE=2 SV=1	ARTSX	1
24	0	2	7,180999964	4,255000129	4,255000129	sp|P18600|ACT1_ARTSX	Actin, clone 205 OS=Artemia sp. PE=2 SV=1	ARTSX	1
24	0	2	7,180999964	4,255000129	4,255000129	sp|P17304|ACTM_APLCA	Actin, muscle OS=Aplysia californica PE=2 SV=1	APLCA	1
24	0	2	7,19999969	4,267000034	4,267000034	sp|P17128|ACT_KLULA	Actin OS=Kluyveromyces lactis (strain ATCC 8585 / CBS 2359 /	KLULA	1
24	0	2	7,180999964	4,255000129	4,255000129	sp|P14235|ACT_CANAX	Actin OS=Candida albicans GN=ACT1 PE=3 SV=1	CANAX	1
24	0	2	7,162000239	4,244000092	4,244000092	sp|P10995|ACT2_XENLA	Actin, alpha skeletal muscle 2 OS=Xenopus laevis GN=act2 PE=	XENLA	1
24	0	2	7,19999969	4,267000034	4,267000034	sp|P10989|ACT_SCHPO	Actin OS=Schizosaccharomyces pombe (strain 972 / ATCC 248	SCHPO	1
24	0	2	7,162000239	4,244000092	4,244000092	sp|P08023|ACTA_CHICK	Actin, aortic smooth muscle OS=Gallus gallus GN=ACTA2 PE=1	CHICK	1
24	0	2	7,180999964	4,255000129	4,255000129	sp|P07837|ACT2_BOMMO	Actin, muscle-type A2 OS=Bombyx mori PE=3 SV=1	BOMMO	1
24	0	2	7,162000239	4,244000092	4,244000092	sp|P04752|ACT3_XENLA	Actin, alpha skeletal muscle 3 OS=Xenopus laevis GN=act3 PE=	XENLA	1
24	0	2	7,162000239	4,244000092	4,244000092	sp|P04751|ACTC_XENLA	Actin, alpha cardiac muscle 1 OS=Xenopus laevis GN=actc1 PE=	XENLA	1
24	0	2	7,180999964	4,255000129	4,255000129	sp|O74258|ACT_OGAPD	Actin OS=Ogataea parapolymorpha (strain DL-1 / ATCC 26012	OGAPD	1
24	0	2	7,19999969	4,267000034	4,267000034	sp|O17503|ACTC_BRALA	Actin, cytoplasmic OS=Branchiostoma lanceolatum PE=2 SV=1	BRALA	1
24	0	2	7,105000317	4,210999981	4,210999981	sp|O17502|ACTM_BRALA	Actin, muscle OS=Branchiostoma lanceolatum PE=2 SV=1	BRALA	1
24	0	2	7,142999768	4,233000055	4,233000055	sp|O15998|ACTM_CIOSA	Actin, muscle OS=Ciona savignyi PE=2 SV=1	CIOSA	1
24	0	2	7,500000298	4,444000125	4,444000125	sp|A5DQP9|ACT_PICGU	Actin OS=Meyerozyma guilliermondii (strain ATCC 6260 / CBS	PICGU	1
24	0	2	2,511999942	1,487999968	1,487999968	sp|A5A3E0|POTEF_HUMAN	POTE ankyrin domain family member F OS=Homo sapiens GN=	HUMAN	1
24	0	2	4,255000129	4,255000129	4,255000129	sp|Q9P4D1|ACT_PICPG	Actin OS=Komagataella pastoris (strain GS115 / ATCC 20864) (	PICPG	1
24	0	2	5,754999816	5,754999816	5,754999816	sp|Q92192|ACT_CALFI	Actin (Fragment) OS=Calanus finmarchicus PE=2 SV=1	CALFI	1
24	0	2	4,156000167	4,156000167	4,156000167	sp|Q54HF0|ACT25_DICDI	Putative actin-25 OS=Dictyostelium discoideum GN=act25 PE=	DICDI	1

24	0	2	9,301999956	9,301999956	9,301999956	sp|Q25381|ACTM_LYTPI	Actin, muscle (Fragment) OS=Lytechinus pictus PE=3 SV=1	LYTPI	1
24	0	2	9,301999956	9,301999956	9,301999956	sp|Q25379|ACT3_LYTPI	Actin, cytoskeletal 3 (Fragment) OS=Lytechinus pictus PE=3 SV	LYTPI	1
24	0	2	4,233000055	4,233000055	4,233000055	sp|P53460|ACT1_HALRO	Actin, muscle 1A OS=Halocynthia roretzi GN=MA1A PE=3 SV=1	HALRO	1
24	0	2	4,969000071	4,969000071	4,969000071	sp|P45521|ACT_PROCL	Actin (Fragment) OS=Procambarus clarkii PE=1 SV=1	PROCL	1
26	2	2	3,370999917	1,765999943	1,765999943	sp|P21910|LAML2_XENLA	Lamin-L(II) OS=Xenopus laevis PE=2 SV=1	XENLA	1
27	2	2	8,392000198	8,392000198	8,392000198	sp|Q9PU28|LYSC_SCOMX	Lysozyme C OS=Scophthalmus maximus GN=lys PE=2 SV=1	SCOMX	1
27	0	2	8,392000198	8,392000198	8,392000198	sp|Q9DD65|LYSC_PAROL	Lysozyme C OS=Paralichthys olivaceus PE=2 SV=1	PAROL	1
27	0	2	8,107999712	8,107999712	8,107999712	sp|Q6B411|LYSM_BOVIN	Lysozyme C, milk isozyme OS=Bos taurus PE=2 SV=1	BOVIN	1
27	0	2	8,107999712	8,107999712	8,107999712	sp|Q659U5|LYSC_HALGR	Lysozyme C OS=Halichoerus grypus GN=LYZ PE=2 SV=1	HALGR	1
27	0	2	8,107999712	8,107999712	8,107999712	sp|Q659U1|LYSC_PHOVI	Lysozyme C OS=Phoca vitulina GN=LYZ PE=2 SV=1	PHOVI	1
27	0	2	8,107999712	8,107999712	8,107999712	sp|Q659U0|LYSC_LEPWE	Lysozyme C OS=Leptonychotes weddellii GN=LYZ PE=2 SV=1	LEPWE	1
27	0	2	8,107999712	8,107999712	8,107999712	sp|Q05820|LYSC2_RAT	Putative lysozyme C-2 OS=Rattus norvegicus GN=Lyz2 PE=5 SV	RAT	1
27	0	2	9,230999649	9,230999649	9,230999649	sp|P85345|LYSC_AMYCA	Lysozyme C OS=Amyda cartilaginea GN=LYZ PE=1 SV=1	AMYCA	1
27	0	2	8,218999952	8,218999952	8,218999952	sp|P85045|LYS_BUFGA	Lysozyme C (Fragment) OS=Bufo gargarizans andrewsi PE=1 S\	BUFGA	1
27	0	2	9,230999649	9,230999649	9,230999649	sp|P81709|LYSC2_CANLF	Lysozyme C, spleen isozyme OS=Canis lupus familiaris PE=1 SV	CANLF	1
27	0	2	8,107999712	8,107999712	8,107999712	sp|P79847|LYSC_PYGNE	Lysozyme C OS=Pygathrix nemaeus GN=LYZ PE=3 SV=1	PYGNE	1
27	0	2	8,107999712	8,107999712	8,107999712	sp|P79811|LYSC_NASLA	Lysozyme C OS=Nasalis larvatus GN=LYZ PE=3 SV=1	NASLA	1
27	0	2	8,107999712	8,107999712	8,107999712	sp|P79239|LYSC_PONPY	Lysozyme C OS=Pongo pygmaeus GN=LYZ PE=2 SV=1	PONPY	1
27	0	2	8,107999712	8,107999712	8,107999712	sp|P79180|LYSC_HYLLA	Lysozyme C OS=Hylobates lar GN=LYZ PE=2 SV=1	HYLLA	1
27	0	2	8,107999712	8,107999712	8,107999712	sp|P79179|LYSC_GORGO	Lysozyme C OS=Gorilla gorilla gorilla GN=LYZ PE=2 SV=1	GORGO	1
27	0	2	8,107999712	8,107999712	8,107999712	sp|P67980|LYSC_TRAFR	Lysozyme C OS=Trachypithecus francoisi GN=LYZ PE=2 SV=1	TRAFR	1
27	0	2	8,107999712	8,107999712	8,107999712	sp|P67979|LYSC_TRAOB	Lysozyme C OS=Trachypithecus obscurus GN=LYZ PE=2 SV=1	TRAOB	1
27	0	2	8,107999712	8,107999712	8,107999712	sp|P67978|LYSC_TRAVT	Lysozyme C OS=Trachypithecus vetulus GN=LYZ PE=3 SV=1	TRAVT	1
27	0	2	8,107999712	8,107999712	8,107999712	sp|P67977|LYSC_SEMEN	Lysozyme C OS=Semnopithecus entellus GN=LYZ PE=1 SV=1	SEMEN	1
27	0	2	8,392000198	8,392000198	8,392000198	sp|P61944|LYSC_TAKRU	Lysozyme C OS=Takifugu rubripes PE=2 SV=1	TAKRU	1
27	0	2	8,107999712	8,107999712	8,107999712	sp|P61632|LYSC_COLGU	Lysozyme C OS=Colobus guereza GN=LYZ PE=2 SV=1	COLGU	1
27	0	2	8,107999712	8,107999712	8,107999712	sp|P61631|LYSC_COLAN	Lysozyme C OS=Colobus angolensis GN=LYZ PE=3 SV=1	COLAN	1
27	0	2	8,107999712	8,107999712	8,107999712	sp|P61628|LYSC_PANTR	Lysozyme C OS=Pan troglodytes GN=LYZ PE=2 SV=1	PANTR	1
27	0	2	8,107999712	8,107999712	8,107999712	sp|P61627|LYSC_PANPA	Lysozyme C OS=Pan paniscus GN=LYZ PE=3 SV=1	PANPA	1
27	0	2	8,107999712	8,107999712	8,107999712	sp|P61626|LYSC_HUMAN	Lysozyme C OS=Homo sapiens GN=LYZ PE=1 SV=1	HUMAN	1
27	0	2	9,230999649	9,230999649	9,230999649	sp|P37712|LYSC_CAMDR	Lysozyme C OS=Camelus dromedarius GN=LYZ PE=1 SV=1	CAMDR	1
27	0	2	8,107999712	8,107999712	8,107999712	sp|P17897|LYZ1_MOUSE	Lysozyme C-1 OS=Mus musculus GN=Lyz1 PE=1 SV=1	MOUSE	1
27	0	2	9,230999649	9,230999649	9,230999649	sp|P16973|LYSC_RABIT	Lysozyme C OS=Oryctolagus cuniculus GN=LYZ PE=1 SV=1	RABIT	1
27	0	2	8,107999712	8,107999712	8,107999712	sp|P12069|LYSC3_PIG	Lysozyme C-3 OS=Sus scrofa PE=1 SV=2	PIG	1
27	0	2	8,218999952	8,218999952	8,218999952	sp|P12068|LYSC2_PIG	Lysozyme C-2 OS=Sus scrofa PE=1 SV=2	PIG	1
27	0	2	9,375	9,375	9,375	sp|P12067|LYSC1_PIG	Lysozyme C-1 OS=Sus scrofa PE=1 SV=1	PIG	1
27	0	2	8,332999796	8,332999796	8,332999796	sp|P11941|LYSC2_ONCMY	Lysozyme C II OS=Oncorhynchus mykiss PE=1 SV=2	ONCMY	1
27	0	2	8,107999712	8,107999712	8,107999712	sp|P08905|LYZ2_MOUSE	Lysozyme C-2 OS=Mus musculus GN=Lyz2 PE=1 SV=2	MOUSE	1
27	0	2	8,107999712	8,107999712	8,107999712	sp|P00697|LYSC1_RAT	Lysozyme C-1 OS=Rattus norvegicus GN=Lyz1 PE=1 SV=2	RAT	1
28	2	2	3,9760001	3,9760001	3,9760001	sp|P08453|GDB2_WHEAT	Gamma-gliadin OS=Triticum aestivum PE=3 SV=1	WHEAT	1
29	2	2	7,805000246	7,805000246	7,805000246	sp|P04792|HSPB1_HUMAN	Heat shock protein beta-1 OS=Homo sapiens GN=HSPB1 PE=1	HUMAN	1
30	1,86	1,86	19,85000074	19,85000074	11,76000014	sp|P47929|LEG7_HUMAN	Galectin-7 OS=Homo sapiens GN=LGALS7 PE=1 SV=2	HUMAN	1
32	1,49	1,59	4,887999967	4,887999967	3,259000182	sp|Q6IFX1|K1C24_RAT	Keratin, type I cytoskeletal 24 OS=Rattus norvegicus GN=Krt24	RAT	1
32	0	1,59	4,687999934	4,687999934	3,125	sp|A1L317|K1C24_MOUSE	Keratin, type I cytoskeletal 24 OS=Mus musculus GN=Krt24 PE	MOUSE	1
33	1,4	1,4	2,597000077	2,597000077	2,597000077	sp|Q08188|TGM3_HUMAN	Protein-glutamine gamma-glutamyltransferase E OS=Homo sa	HUMAN	1
35	0,67	0,67	0,460099988	0,460099988	0,460099988	sp|Q5D862|FILA2_HUMAN	Filaggrin-2 OS=Homo sapiens GN=FLG2 PE=1 SV=1	HUMAN	1
34	0,7	0,7	11,94999963	5,905999988	0	sp|Q8WNW3|PLAK_PIG	Junction plakoglobin OS=Sus scrofa GN=Jup PE=2 SV=1	PIG	0
34	0	0,7	11,94999963	5,905999988	0	sp|Q8SPJ1|PLAK_BOVIN	Junction plakoglobin OS=Bos taurus GN=JUP PE=2 SV=1	BOVIN	0
34	0	0,7	11,94999963	5,905999988	0	sp|Q6P0K8|PLAK_RAT	Junction plakoglobin OS=Rattus norvegicus GN=Jup PE=1 SV=1	RAT	0
34	0	0,7	11,94999963	5,905999988	0	sp|Q02257|PLAK_MOUSE	Junction plakoglobin OS=Mus musculus GN=Jup PE=1 SV=3	MOUSE	0
34	0	0,7	11,94999963	5,905999988	0	sp|P14923|PLAK_HUMAN	Junction plakoglobin OS=Homo sapiens GN=JUP PE=1 SV=3	HUMAN	0
36	0,4	0,4	5,189999938	1,080000028	0	sp|P15924|DESP_HUMAN	Desmoplakin OS=Homo sapiens GN=DSP PE=1 SV=3	HUMAN	0
36	0	0,37	4,856000096	0,381499995	0	sp|E9Q557|DESP_MOUSE	Desmoplakin OS=Mus musculus GN=Dsp PE=1 SV=1	MOUSE	0
37	0,37	0,37	2,563999966	2,563999966	0	RRRRRsp|C4Z5P8|UXAC_EUBE2	REVERSED Uronate isomerase OS=Eubacterium eligens (strain	EUBE2	0
38	0,18	0,18	6,263999641	1,118999999	0	sp|Q08554|DSC1_HUMAN	Desmocollin-1 OS=Homo sapiens GN=DSC1 PE=1 SV=2	HUMAN	0
39	0,18	0,18	3,759000078	3,759000078	0	sp|Q7VBI9|ISPT_PROMA	Isoprenyl transferase OS=Prochlorococcus marinus (strain SAR	PROMA	0
40	0,18	0,18	1,917999983	1,917999983	0	RRRRRsp|Q9SFC7|FB135_ARATH	REVERSED F-box protein At3g07870 OS=Arabidopsis thaliana (	ARATH	0
41	0,14	0,14	10,27999967	10,27999967	0	RRRRRsp|B9K6M6|Y433_THENN	REVERSED UPF0102 protein CTN_0433 OS=Thermotoga neapc	THENN	0
42	0,12	0,12	7,24600032	3,864999861	0	RRRRRsp|Q37626|NU6M_PROW	REVERSED NADH-ubiquinone oxidoreductase chain 6 OS=Prot	PROWI	0
42	0	0,12	1,013999991	1,013999991	0	RRRRRsp|Q0V147|AMPP2_PHAN	REVERSED Probable Xaa-Pro aminopeptidase SNOG_02267 OS	PHANO	0
42	0	0,12	0,863900036	0,863900036	0	RRRRRsp|E3RNJ5|AMPP2_PYRTT	REVERSED Probable Xaa-Pro aminopeptidase PTT_10145 OS=I	PYRTT	0
42	0	0,12	1,212000009	1,212000009	0	RRRRRsp|B2WKR4|AMPP2_PYRT	REVERSED Probable Xaa-Pro aminopeptidase PTRG_10574 OS	PYRTR	0
43	0,11	0,11	2,452000044	2,452000044	0	RRRRRsp|Q81M67|BUK_BACAN	REVERSED Probable butyrate kinase OS=Bacillus anthracis GN	BACAN	0
43	0	0,11	2,452000044	2,452000044	0	RRRRRsp|Q818T1|BUK_BACCR	REVERSED Probable butyrate kinase OS=Bacillus cereus (strain	BACCR	0
43	0	0,11	2,452000044	2,452000044	0	RRRRRsp|Q731D0|BUK_BACC1	REVERSED Probable butyrate kinase OS=Bacillus cereus (strain	BACC1	0
43	0	0,11	2,452000044	2,452000044	0	RRRRRsp|Q6HE00|BUK_BACHK	REVERSED Probable butyrate kinase OS=Bacillus thuringiensis	BACHK	0
43	0	0,11	2,452000044	2,452000044	0	RRRRRsp|Q635C1|BUK_BACCZ	REVERSED Probable butyrate kinase OS=Bacillus cereus (strain	BACCZ	0
43	0	0,11	2,452000044	2,452000044	0	RRRRRsp|C3P7U3|BUK_BACAA	REVERSED Probable butyrate kinase OS=Bacillus anthracis (str	BACAA	0
43	0	0,11	2,452000044	2,452000044	0	RRRRRsp|C3LJT8|BUK_BACAC	REVERSED Probable butyrate kinase OS=Bacillus anthracis (str	BACAC	0
43	0	0,11	2,452000044	2,452000044	0	RRRRRsp|C1ERN7|BUK_BACC3	REVERSED Probable butyrate kinase OS=Bacillus cereus (strain	BACC3	0
43	0	0,11	2,452000044	2,452000044	0	RRRRRsp|B9IXF8|BUK_BACCQ	REVERSED Probable butyrate kinase OS=Bacillus cereus (strain	BACCQ	0
43	0	0,11	2,452000044	2,452000044	0	RRRRRsp|B7JM15|BUK_BACC0	REVERSED Probable butyrate kinase OS=Bacillus cereus (strain	BACC0	0
43	0	0,11	2,452000044	2,452000044	0	RRRRRsp|B7IXF5|BUK_BACC2	REVERSED Probable butyrate kinase OS=Bacillus cereus (strain	BACC2	0
43	0	0,11	2,452000044	2,452000044	0	RRRRRsp|B7HNS7|BUK_BACC7	REVERSED Probable butyrate kinase OS=Bacillus cereus (strain	BACC7	0
43	0	0,11	2,452000044	2,452000044	0	RRRRRsp|B7HB35|BUK_BACC4	REVERSED Probable butyrate kinase OS=Bacillus cereus (strain	BACC4	0
43	0	0,11	2,452000044	2,452000044	0	RRRRRsp|A7GSI3|BUK_BACCN	REVERSED Probable butyrate kinase OS=Bacillus cytotoxicus (s	BACCN	0
43	0	0,01	2,452000044	2,452000044	0	RRRRRsp|A9VGB0|BUK_BACWK	REVERSED Probable butyrate kinase OS=Bacillus weihensteph	BACWK	0
44	0,08	0,08	0,4954	0,4954	0	sp|Q8TER0|SNED1_HUMAN	Sushi, nidogen and EGF-like domain-containing protein 1 OS=I	HUMAN	0
45	0,08	0,08	1,455000043	1,455000043	0	sp|Q7ZTM9|EIF3D_XENLA	Eukaryotic translation initiation factor 3 subunit D OS=Xenopu	XENLA	0
45	0	0,08	1,448999997	1,448999997	0	sp|Q6TH15|EIF3D_DANRE	Eukaryotic translation initiation factor 3 subunit D OS=Danio r	DANRE	0
45	0	0,08	1,448999997	1,448999997	0	sp|Q6P8G0|EIF3D_XENTR	Eukaryotic translation initiation factor 3 subunit D OS=Xenopu	XENTR	0
45	0	0,08	1,460000034	1,460000034	0	sp|Q6AYK8|EIF3D_RAT	Eukaryotic translation initiation factor 3 subunit D OS=Rattus	RAT	0
45	0	0,08	1,460000034	1,460000034	0	sp|Q5R925|EIF3D_PONAB	Eukaryotic translation initiation factor 3 subunit D OS=Pongo a	PONAB	0
45	0	0,08	1,460000034	1,460000034	0	sp|Q4R8R4|EIF3D_MACFA	Eukaryotic translation initiation factor 3 subunit D OS=Macaca	MACFA	0
45	0	0,08	1,460000034	1,460000034	0	sp|Q3T122|EIF3D_BOVIN	Eukaryotic translation initiation factor 3 subunit D OS=Bos tau	BOVIN	0
45	0	0,08	1,460000034	1,460000034	0	sp|O70194|EIF3D_MOUSE	Eukaryotic translation initiation factor 3 subunit D OS=Mus mu	MOUSE	0
45	0	0,08	1,460000034	1,460000034	0	sp|O15371|EIF3D_HUMAN	Eukaryotic translation initiation factor 3 subunit D OS=Homo s	HUMAN	0
46	0,08	0,08	4,061000049	4,061000049	0	sp|P12921|TMRB_BACSU	Tunicamycin resistance protein OS=Bacillus subtilis (strain 168	BACSU	0
47	0,08	0,08	3,508999944	3,508999944	0	sp|O13859|MU125_SCHPO	Meiotically up-regulated gene 125 protein OS=Schizosaccharo	SCHPO	0
48	0,08	0,08	1,267999969	1,267999969	0	RRRRRsp|Q9URZ3|PUT4_SCHPO	REVERSED Probable proline-specific permease put4 OS=Schizo	SCHPO	0
49	0,08	0,08	0,964200031	0,964200031	0	RRRRRsp|Q8Z303|KATG_SALTI	REVERSED Catalase-peroxidase OS=Salmonella typhi GN=katG	SALTI	0
49	0	0,08	0,964200031	0,964200031	0	RRRRRsp|Q5PK60|KATG_SALPA	REVERSED Catalase-peroxidase OS=Salmonella paratyphi A (st	SALPA	0
49	0	0,08	0,964200031	0,964200031	0	RRRRRsp|Q57HA8|KATG_SALCH	REVERSED Catalase-peroxidase OS=Salmonella choleraesuis (s	SALCH	0
49	0	0,08	1,971999928	1,971999928	0	RRRRRsp|P59487|AROB_BUCBP	REVERSED 3-dehydroquinate synthase OS=Buchnera aphidicol	BUCBP	0
49	0	0,08	0,964200031	0,964200031	0	RRRRRsp|P17750|KATG_SALTY	REVERSED Catalase-peroxidase OS=Salmonella typhimurium (s	SALTY	0
49	0	0,08	0,964200031	0,964200031	0	RRRRRsp|B5BJM5|KATG_SALPK	REVERSED Catalase-peroxidase OS=Salmonella paratyphi A (st	SALPK	0
49	0	0,08	0,964200031	0,964200031	0	RRRRRsp|B4TQG7|KATG_SALSV	REVERSED Catalase-peroxidase OS=Salmonella schwarzengrur	SALSV	0
49	0	0,08	0,964200031	0,964200031	0	RRRRRsp|B4TCP6|KATG_SALHS	REVERSED Catalase-peroxidase OS=Salmonella heidelberg (str	SALHS	0
49	0	0,08	0,964200031	0,964200031	0	RRRRRsp|B4T0V5|KATG_SALNS	REVERSED Catalase-peroxidase OS=Salmonella newport (strair	SALNS	0
49	0	0,08	0,964200031	0,964200031	0	RRRRRsp|A9N0F0|KATG_SALPB	REVERSED Catalase-peroxidase OS=Salmonella paratyphi B (st	SALPB	0
49	0	0,08	0,964200031	0,964200031	0	RRRRRsp|A9MI19|KATG_SALAR	REVERSED Catalase-peroxidase OS=Salmonella arizonae (strair	SALAR	0

49	0	0,08	0,964200031	0,964200031	0	RRRRRsp|A8AKX8|KATG_CITK8	REVERSED Catalase-peroxidase OS=Citrobacter koseri (strain A	CITK8	0
49	0	0,08	0,965499971	0,965499971	0	RRRRRsp|A6T9H9|KATG_KLEP7	REVERSED Catalase-peroxidase OS=Klebsiella pneumoniae sub	KLEP7	0
49	0	0,08	0,964200031	0,964200031	0	RRRRRsp|A4WG57|KATG_ENT38	REVERSED Catalase-peroxidase OS=Enterobacter sp. (strain 63	ENT38	0
50	0,07	0,08	1,855999976	1,855999976	0	RRRRRsp|A4SCK8|GCSPB_CHLPM	REVERSED Probable glycine dehydrogenase (decarboxylating)	CHLPM	0
51	0,07	0,07	3,726999834	3,726999834	0	sp|O35003|T1214_ARCFU	Probable transposase for insertion sequence element ISA1214	ARCFU	0
52	0,06	0,07	3,57099995	3,57099995	0	sp|A6WX58|Y840_OCHA4	UPF0314 protein Oant_0840 OS=Ochrobactrum anthropi (stra	OCHA4	0
52	0	0,07	1,243000012	1,243000012	0	RRRRRsp|Q5UPJ3|YL116_MIMIV	REVERSED Uncharacterized protein L116 OS=Acanthamoeba p	MIMIV	0
52	0	0,07	1,620000042	1,620000042	0	RRRRRsp|Q39K17|HEM1_BURL3	REVERSED Glutamyl-tRNA reductase OS=Burkholderia lata (str	BURL3	0
52	0	0,07	1,620000042	1,620000042	0	RRRRRsp|Q1BSB3|HEM1_BURCA	REVERSED Glutamyl-tRNA reductase OS=Burkholderia cenocep	BURCA	0
52	0	0,07	1,620000042	1,620000042	0	RRRRRsp|Q0BIP9|HEM1_BURCM	REVERSED Glutamyl-tRNA reductase OS=Burkholderia ambifar	BURCM	0
52	0	0,07	1,620000042	1,620000042	0	RRRRRsp|B4E6U7|HEM1_BURCJ	REVERSED Glutamyl-tRNA reductase OS=Burkholderia cenocep	BURCJ	0
52	0	0,07	1,620000042	1,620000042	0	RRRRRsp|B1YSD6|HEM1_BURA4	REVERSED Glutamyl-tRNA reductase OS=Burkholderia ambifar	BURA4	0
52	0	0,07	1,620000042	1,620000042	0	RRRRRsp|B1JUS5|HEM1_BURCC	REVERSED Glutamyl-tRNA reductase OS=Burkholderia cenocep	BURCC	0
52	0	0,07	1,620000042	1,620000042	0	RRRRRsp|A9AJ67|HEM1_BURM1	REVERSED Glutamyl-tRNA reductase OS=Burkholderia multivo	BURM1	0
52	0	0,07	1,620000042	1,620000042	0	RRRRRsp|A4JB51|HEM1_BURVG	REVERSED Glutamyl-tRNA reductase OS=Burkholderia vietnam	BURVG	0
52	0	0,07	1,620000042	1,620000042	0	RRRRRsp|A0K438|HEM1_BURCH	REVERSED Glutamyl-tRNA reductase OS=Burkholderia cenocep	BURCH	0
53	0,06	0,06	5,104000121	3,403000161	0	sp|Q7YR44|CDSN_PANTR	Corneodesmosin OS=Pan troglodytes GN=CDSN PE=2 SV=1	PANTR	0
53	0	0,06	5,056000128	3,370999917	0	sp|Q5TM45|CDSN_MACMU	Corneodesmosin OS=Macaca mulatta GN=CDSN PE=3 SV=1	MACMU	0
53	0	0,06	5,104000121	3,403000161	0	sp|Q15517|CDSN_HUMAN	Corneodesmosin OS=Homo sapiens GN=CDSN PE=1 SV=3	HUMAN	0
54	0,06	0,06	0,613899995	0,245500007	0	sp|Q756G2|TOM1_ASHGO	Probable E3 ubiquitin-protein ligase TOM1 OS=Ashbya gossyp	ASHGO	0
55	0,06	0,06	0,598799996	0,598799996	0	sp|Q9LZW9|RTOR2_ARATH	Regulatory-associated protein of TOR 2 OS=Arabidopsis thalia	ARATH	0
56	0,06	0,06	8,87100026	8,87100026	0	sp|P01085|IAA1_WHEAT	Alpha-amylase inhibitor 0.19 OS=Triticum aestivum PE=1 SV=1	WHEAT	0
56	0	0,06	8,87100026	8,87100026	0	sp|P01084|IAA5_WHEAT	Alpha-amylase inhibitor 0.53 OS=Triticum aestivum PE=1 SV=1	WHEAT	0
57	0,05	0,05	5,404999852	2,078999951	0	RRRRRsp|Q1IED4|GATB_PSEE4	REVERSED Aspartyl/glutamyl-tRNA(Asn/Gln) amidotransferase	PSEE4	0
58	0,05	0,05	2,219000086	0,986399967	0	RRRRRsp|C1CVF4|GYRA_DEIDV	REVERSED DNA gyrase subunit A OS=Deinococcus deserti (stra	DEIDV	0
59	0,05	0,05	3,430999815	3,430999815	0	sp|Q96MC5|CP045_HUMAN	Uncharacterized protein C16orf45 OS=Homo sapiens GN=C16	HUMAN	0
59	0	0,05	3,448000178	3,448000178	0	sp|Q8R1Y2|CP045_MOUSE	Uncharacterized protein C16orf45 homolog OS=Mus musculus	MOUSE	0
59	0	0,05	3,430999815	3,430999815	0	sp|Q5R562|CP045_PONAB	Uncharacterized protein C16orf45 homolog OS=Pongo abelii P	PONAB	0
59	0	0,05	3,448000178	3,448000178	0	sp|Q5FVJ5|CP045_RAT	Uncharacterized protein C16orf45 homolog OS=Rattus norveg	RAT	0
59	0	0,05	0,9296	0,9296	0	RRRRRsp|Q5XHX2|TPRN_RAT	REVERSED Taperin OS=Rattus norvegicus GN=Tprn PE=1 SV=2	RAT	0
59	0	0,05	0,934599992	0,934599992	0	RRRRRsp|A2AI08|TPRN_MOUSE	REVERSED Taperin OS=Mus musculus GN=Tprn PE=1 SV=1	MOUSE	0
60	0,05	0,05	1,453999989	1,453999989	0	sp|Q96K58|ZN668_HUMAN	Zinc finger protein 668 OS=Homo sapiens GN=ZNF668 PE=1 SV	HUMAN	0
60	0	0,05	1,453999989	1,453999989	0	sp|Q8K2R5|ZN668_MOUSE	Zinc finger protein 668 OS=Mus musculus GN=Znf668 PE=2 SV	MOUSE	0
60	0	0,05	1,453999989	1,453999989	0	sp|Q4R3I5|ZN668_MACFA	Zinc finger protein 668 OS=Macaca fascicularis GN=ZNF668 PE	MACFA	0
60	0	0,05	1,453999989	1,453999989	0	sp|Q2TA17|ZN668_BOVIN	Zinc finger protein 668 OS=Bos taurus GN=ZNF668 PE=2 SV=2	BOVIN	0
61	0,05	0,05	1,601999998	1,601999998	0	sp|Q92Y37|NHAA_RHIME	Na(+)/H(+) antiporter NhaA OS=Rhizobium meliloti (strain 102	RHIME	0
62	0,05	0,05	1,631999947	1,631999947	0	sp|Q42191|OXA1_ARATH	Mitochondrial inner membrane protein OXA1 OS=Arabidopsis	ARATH	0
63	0,05	0,05	1,999999955	1,999999955	0	RRRRRsp|Q7NN66|ARGD_GLOVI	REVERSED Acetylornithine aminotransferase OS=Gloeobacter	GLOVI	0
64	0,05	0,05	3,477999941	3,477999941	0	RRRRRsp|Q73GB0|PSD_WOLPM	REVERSED Phosphatidylserine decarboxylase proenzyme OS=V	WOLPM	0
64	0	0,05	3,477999941	3,477999941	0	RRRRRsp|C0R4E0|PSD_WOLWR	REVERSED Phosphatidylserine decarboxylase proenzyme OS=V	WOLWR	0

N	Ctrldudoso_CSI_1	Total	%Cov	%Cov(50)	%Cov(95)	Accession	Name	Species	Peptides(95%)
1	89,03	89,03	70,02999783	68,79000068	68,79000068	sp|P04264|K2C1_HUMAN	Keratin, type II cytoskeletal 1 OS=Homo sapiens GN=KRT	HUMAN	70
2	69,67	69,67	58,89999866	55,4799974	55,4799974	sp|P13645|K1C10_HUMAN	Keratin, type I cytoskeletal 10 OS=Homo sapiens GN=KR	HUMAN	48
3	67,55	74,99	77,14999914	70,88999748	60,72000265	sp|P35908|K22E_HUMAN	Keratin, type II cytoskeletal 2 epidermal OS=Homo sapie	HUMAN	46
4	57,29	57,4	78,64999771	75,59999824	69,98000145	sp|P35527|K1C9_HUMAN	Keratin, type I cytoskeletal 9 OS=Homo sapiens GN=KRT	HUMAN	42
6	20,26	20,26	64,49999809	64,49999809	64,49999809	sp|P00761|TRYP_PIG	Trypsin OS=Sus scrofa PE=1 SV=1	PIG	29
5	32,07	35,44	57,80000758	48,73000085	48,73000085	sp|P02533|K1C14_HUMAN	Keratin, type I cytoskeletal 14 OS=Homo sapiens GN=KR	HUMAN	21
7	17,59	30,68	38,80999982	32,37000108	27,45999992	sp|P13647|K2C5_HUMAN	Keratin, type II cytoskeletal 5 OS=Homo sapiens GN=KRT	HUMAN	16
7	0	28,83	36,1499995	29,73000109	24,8300001	sp|A5A6M8|K2C5_PANTR	Keratin, type II cytoskeletal 5 OS=Pan troglodytes GN=K	PANTR	15
9	13,2	29,24	34,92999971	33,16000104	27,84000039	sp|P48668|K2C6C_HUMAN	Keratin, type II cytoskeletal 6C OS=Homo sapiens GN=K	HUMAN	15
9	0	29,2	35,98999977	32,80000091	27,84000039	sp|P02538|K2C6A_HUMAN	Keratin, type II cytoskeletal 6A OS=Homo sapiens GN=K	HUMAN	15
24	5,47	9,78	12,98000067	11,42000034	11,42000034	sp|Q7Z794|K2C1B_HUMAN	Keratin, type II cytoskeletal 1b OS=Homo sapiens GN=K	HUMAN	12
28	4,02	22,86	34,25000012	24,95000064	24,95000064	sp|P08779|K1C16_HUMAN	Keratin, type I cytoskeletal 16 OS=Homo sapiens GN=KR	HUMAN	12
24	0	11,04	15,02999961	12,72000074	12,72000074	sp|Q6IG01|K2C1B_RAT	Keratin, type II cytoskeletal 1b OS=Rattus norvegicus GN	RAT	10
24	0	11,04	11,54000014	11,54000014	11,54000014	sp|Q6IFZ6|K2C1B_MOUSE	Keratin, type II cytoskeletal 1b OS=Mus musculus GN=K	MOUSE	10
29	4	18,72	29,39999998	23,14999998	23,14999998	sp|Q04695|K1C17_HUMAN	Keratin, type I cytoskeletal 17 OS=Homo sapiens GN=KR	HUMAN	10
10	12,07	12,07	14,86999989	11,2499997	9,437999874	sp|Q02413|DSG1_HUMAN	Desmoglein-1 OS=Homo sapiens GN=DSG1 PE=1 SV=2	HUMAN	9
29	0	16,67	26,39000118	20,13999969	20,13999969	sp|A5A6M0|K1C17_PANTR	Keratin, type I cytoskeletal 17 OS=Pan troglodytes GN=K	PANTR	9
8	14,1	14,1	40,50000012	33,46999884	33,46999884	sp|P31944|CASPE_HUMAN	Caspase-14 OS=Homo sapiens GN=CASP14 PE=1 SV=2	HUMAN	8
12	11,57	11,57	15,88000059	12,74999976	10,50999984	sp|Q08554|DSC1_HUMAN	Desmocollin-1 OS=Homo sapiens GN=DSC1 PE=1 SV=2	HUMAN	8
13	11,54	11,54	20,02999932	15,76000005	15,76000005	sp|P02768|ALBU_HUMAN	Serum albumin OS=Homo sapiens GN=ALB PE=1 SV=2	HUMAN	8
13	0	11,42	15,60000032	14,45000023	14,45000023	sp|Q5NVH5|ALBU_PONAB	Serum albumin OS=Pongo abelii GN=ALB PE=2 SV=2	PONAB	7
14	11,5	11,5	58,70000124	52,17000246	52,17000246	sp|P15252|REF_HEVBR	Rubber elongation factor protein OS=Hevea brasiliensis	HEVBR	7
15	11,29	11,29	31,63999915	25,67000091	25,67000091	sp|P04406|G3P_HUMAN	Glyceraldehyde-3-phosphate dehydrogenase OS=Homo	HUMAN	7
16	10,45	10,45	56,36000037	56,36000037	56,36000037	sp|P81605|DCD_HUMAN	Dermcidin OS=Homo sapiens GN=DCD PE=1 SV=2	HUMAN	7
11	11,65	11,65	25,90000033	22,82000035	18,71999949	sp|P29508|SPB3_HUMAN	Serpin B3 OS=Homo sapiens GN=SERPINB3 PE=1 SV=2	HUMAN	6
15	0	9,24	26,57000124	20,60000002	20,60000002	sp|Q5RAB4|G3P_PONAB	Glyceraldehyde-3-phosphate dehydrogenase OS=Pongo	PONAB	6
17	10,01	10,01	55,44999838	55,44999838	44,54999864	sp|P31151|S10A7_HUMAN	Protein S100-A7 OS=Homo sapiens GN=S100A7 PE=1 SV	HUMAN	5
18	8,55	8,55	20,46999931	20,46999931	17,11000055	sp|P25311|ZA2G_HUMAN	Zinc-alpha-2-glycoprotein OS=Homo sapiens GN=AZGP1	HUMAN	4
19	8	8	14,13999945	7,359000295	7,359000295	sp|Q08188|TGM3_HUMAN	Protein-glutamine gamma-glutamyltransferase E OS=Ho	HUMAN	4
20	7,17	7,17	30,91000021	17,88000017	17,88000017	sp|P01857|IGHG1_HUMAN	Ig gamma-1 chain C region OS=Homo sapiens GN=IGHG	HUMAN	4
37	2,01	8,24	10,96000001	7,69200027	7,69200027	sp|Q8N1N4|K2C78_HUMAN	Keratin, type II cytoskeletal 78 OS=Homo sapiens GN=K	HUMAN	4
21	6,27	6,27	4,492999986	1,881000027	0,940399989	sp|P15924|DESP_HUMAN	Desmoplakin OS=Homo sapiens GN=DSP PE=1 SV=3	HUMAN	3
22	6	6	37,72000074	37,72000074	37,72000074	sp|P06702|S10A9_HUMAN	Protein S100-A9 OS=Homo sapiens GN=S100A9 PE=1 SV	HUMAN	3
23	6	6	54,07999754	44,9000001	44,9000001	sp|P01040|CYTA_HUMAN	Cystatin-A OS=Homo sapiens GN=CSTA PE=1 SV=1	HUMAN	3
25	5,24	5,24	59,14000273	35,47999859	35,47999859	sp|P05109|S10A8_HUMAN	Protein S100-A8 OS=Homo sapiens GN=S100A8 PE=1 SV	HUMAN	3
27	4,21	4,21	39,03999925	28,76999974	23,28999937	sp|P12273|PIP_HUMAN	Prolactin-inducible protein OS=Homo sapiens GN=PIP P	HUMAN	3
30	4	6,02	10,86999997	8,071999997	8,071999997	sp|P02769|ALBU_BOVIN	Serum albumin OS=Bos taurus GN=ALB PE=1 SV=4	BOVIN	3
21	0	4,04	3,745999932	1,525999978	0,589699997	sp|E9Q557|DESP_MOUSE	Desmoplakin OS=Mus musculus GN=Dsp PE=1 SV=1	MOUSE	2
26	4,54	4,54	44,58999932	44,58999932	32,78999925	sp|Q8MKD1|UBB_HORSE	Polyubiquitin-B OS=Equus caballus GN=UBB PE=2 SV=3	HORSE	2
26	0	4,54	44,74000037	44,74000037	32,89000094	sp|Q865C5|UBIQ_CAMDR	Ubiquitin OS=Camelus dromedarius PE=3 SV=2	CAMDR	2
26	0	4,54	41,98000133	41,98000133	30,86000085	sp|Q63429|UBC_RAT	Polyubiquitin-C OS=Rattus norvegicus GN=Ubc PE=1 SV=	RAT	2
26	0	4,54	53,96999717	53,96999717	39,68000114	sp|P84589|UBIQ_LUMTE	Ubiquitin (Fragment) OS=Lumbricus terrestris PE=1 SV=	LUMTE	2
26	0	4,54	21,78999931	21,78999931	16,03000015	sp|P79781|RS27A_CHICK	Ubiquitin-40S ribosomal protein S27a OS=Gallus gallus	CHICK	2
26	0	4,54	26,55999959	26,55999959	19,52999979	sp|P68205|RL40_OPHHA	Ubiquitin-60S ribosomal protein L40 OS=Ophiophagus h	OPHHA	2
26	0	4,54	21,78999931	21,78999931	16,03000015	sp|P68203|RS27A_SPOFR	Ubiquitin-40S ribosomal protein S27a OS=Spodoptera fr	SPOFR	2
26	0	4,54	21,94000036	21,94000036	16,13000035	sp|P68202|RS27A_PLUXY	Ubiquitin-40S ribosomal protein S27a OS=Plutella xylost	PLUXY	2
26	0	4,54	21,78999931	21,78999931	16,03000015	sp|P68200|RS27A_ICTPU	Ubiquitin-40S ribosomal protein S27a OS=Ictalurus punc	ICTPU	2
26	0	4,54	44,74000037	44,74000037	32,89000094	sp|P68197|UBIQ_CERCA	Ubiquitin OS=Ceratitis capitata PE=1 SV=1	CERCA	2
26	0	4,54	26,55999959	26,55999959	19,52999979	sp|P63053|RL40_PIG	Ubiquitin-60S ribosomal protein L40 OS=Sus scrofa GN=	PIG	2
26	0	4,54	26,55999959	26,55999959	19,52999979	sp|P63052|RL40_FELCA	Ubiquitin-60S ribosomal protein L40 OS=Felis catus GN=	FELCA	2
26	0	4,54	26,55999959	26,55999959	19,52999979	sp|P63050|RL40_CANLF	Ubiquitin-60S ribosomal protein L40 OS=Canis lupus fam	CANLF	2
26	0	4,54	26,55999959	26,55999959	19,52999979	sp|P63048|RL40_BOVIN	Ubiquitin-60S ribosomal protein L40 OS=Bos taurus GN=	BOVIN	2
26	0	4,54	21,78999931	21,78999931	16,03000015	sp|P62992|RS27A_BOVIN	Ubiquitin-40S ribosomal protein S27a OS=Bos taurus GN	BOVIN	2
26	0	4,54	26,55999959	26,55999959	19,52999979	sp|P62987|RL40_HUMAN	Ubiquitin-60S ribosomal protein L40 OS=Homo sapiens	HUMAN	2
26	0	4,54	26,55999959	26,55999959	19,52999979	sp|P62986|RL40_RAT	Ubiquitin-60S ribosomal protein L40 OS=Rattus norvegic	RAT	2
26	0	4,54	26,55999959	26,55999959	19,52999979	sp|P62984|RL40_MOUSE	Ubiquitin-60S ribosomal protein L40 OS=Mus musculus	MOUSE	2
26	0	4,54	21,78999931	21,78999931	16,03000015	sp|P62983|RS27A_MOUSE	Ubiquitin-40S ribosomal protein S27a OS=Mus musculus	MOUSE	2
26	0	4,54	21,78999931	21,78999931	16,03000015	sp|P62982|RS27A_RAT	Ubiquitin-40S ribosomal protein S27a OS=Rattus norveg	RAT	2
26	0	4,54	21,78999931	21,78999931	16,03000015	sp|P62979|RS27A_HUMAN	Ubiquitin-40S ribosomal protein S27a OS=Homo sapiens	HUMAN	2
26	0	4,54	21,78999931	21,78999931	16,03000015	sp|P62978|RS27A_CAVPO	Ubiquitin-40S ribosomal protein S27a OS=Cavia porcellu	CAVPO	2
26	0	4,54	41,33999944	41,33999944	30,39999902	sp|P62976|UBIQP_CRIGR	Polyubiquitin OS=Cricetulus griseus PE=2 SV=2	CRIGR	2
26	0	4,54	44,74000037	44,74000037	32,89000094	sp|P62975|UBIQ_RABIT	Ubiquitin OS=Oryctolagus cuniculus PE=1 SV=1	RABIT	2
26	0	4,54	46,11000121	46,11000121	35,33000052	sp|P62972|UBIQP_XENLA	Polyubiquitin (Fragment) OS=Xenopus laevis PE=1 SV=2	XENLA	2
26	0	4,54	21,94000036	21,94000036	16,13000035	sp|P29504|RS27A_MANSE	Ubiquitin-40S ribosomal protein S27a OS=Manduca sext	MANSE	2
26	0	4,54	26,55999959	26,55999959	19,52999979	sp|P18101|RL40_DROME	Ubiquitin-60S ribosomal protein L40 OS=Drosophila mel	DROME	2
26	0	4,54	21,78999931	21,78999931	16,03000015	sp|P15357|RS27A_DROME	Ubiquitin-40S ribosomal protein S27a OS=Drosophila me	DROME	2
26	0	4,54	44,35000122	44,35000122	32,60999918	sp|P0CH28|UBC_BOVIN	Polyubiquitin-C OS=Bos taurus GN=UBC PE=1 SV=1	BOVIN	2
26	0	4,54	44,5600003	44,5600003	32,76999891	sp|P0CG69|UBIQP_DROME	Polyubiquitin OS=Drosophila melanogaster GN=Ubi-p63	DROME	2
26	0	4,54	44,65000033	44,65000033	32,82999992	sp|P0CG68|UBC_PIG	Polyubiquitin-C OS=Sus scrofa GN=UBC PE=2 SV=1	PIG	2
26	0	4,54	44,53999996	44,53999996	32,74999857	sp|P0CG67|UBB_GORGO	Polyubiquitin-B OS=Gorilla gorilla gorilla GN=UBB PE=3 S	GORGO	2
26	0	4,54	44,65999901	44,65999901	32,8399986	sp|P0CG66|UBC_GORGO	Polyubiquitin-C OS=Gorilla gorilla gorilla GN=UBC PE=3 S	GORGO	2
26	0	4,54	44,53999996	44,53999996	32,74999857	sp|P0CG65|UBB_PANTR	Polyubiquitin-B OS=Pan troglodytes GN=UBB PE=3 SV=1	PANTR	2
26	0	4,54	44,67999935	44,67999935	32,85000026	sp|P0CG64|UBC_PANTR	Polyubiquitin-C OS=Pan troglodytes GN=UBC PE=3 SV=1	PANTR	2
26	0	4,54	44,58999932	44,58999932	32,78999925	sp|P0CG62|UBB_CHICK	Polyubiquitin-B OS=Gallus gallus GN=UBB PE=2 SV=1	CHICK	2
26	0	4,54	44,67999935	44,67999935	32,85000026	sp|P0CG61|UBC_PONPY	Polyubiquitin-C OS=Pongo pygmaeus GN=UBC PE=3 SV=	PONPY	2
26	0	4,54	44,53999996	44,53999996	32,74999857	sp|P0CG60|UBB_PONPY	Polyubiquitin-B OS=Pongo pygmaeus GN=UBB PE=3 SV=	PONPY	2
26	0	4,54	44,58999932	44,58999932	32,78999925	sp|P0CG55|UBB_SHEEP	Polyubiquitin-B OS=Ovis aries GN=UBB PE=2 SV=1	SHEEP	2
26	0	4,54	43,72999966	43,72999966	32,15000033	sp|P0CG54|UBB_CAVPO	Polyubiquitin-B OS=Cavia porcellus GN=UBB PE=2 SV=1	CAVPO	2
26	0	4,54	44,58999932	44,58999932	32,78999925	sp|P0CG53|UBB_BOVIN	Polyubiquitin-B OS=Bos taurus GN=UBB PE=1 SV=1	BOVIN	2
26	0	4,54	44,58999932	44,58999932	32,78999925	sp|P0CG51|UBB_RAT	Polyubiquitin-B OS=Rattus norvegicus GN=Ubb PE=1 SV=	RAT	2
26	0	4,54	41,69000089	41,69000089	30,64999878	sp|P0CG50|UBC_MOUSE	Polyubiquitin-C OS=Mus musculus GN=Ubc PE=1 SV=2	MOUSE	2
26	0	4,54	44,58999932	44,58999932	32,78999925	sp|P0CG49|UBB_MOUSE	Polyubiquitin-B OS=Mus musculus GN=Ubb PE=2 SV=1	MOUSE	2
26	0	4,54	44,67000067	44,67000067	32,85000026	sp|P0CG48|UBC_HUMAN	Polyubiquitin-C OS=Homo sapiens GN=UBC PE=1 SV=3	HUMAN	2
26	0	4,54	44,53999996	44,53999996	32,74999857	sp|P0CG47|UBB_HUMAN	Polyubiquitin-B OS=Homo sapiens GN=UBB PE=1 SV=1	HUMAN	2
26	0	4,54	26,55999959	26,55999959	19,52999979	sp|P0C276|RL40_SHEEP	Ubiquitin-60S ribosomal protein L40 OS=Ovis aries GN=U	SHEEP	2
26	0	4,54	26,55999959	26,55999959	19,52999979	sp|P0C275|RL40_PONPY	Ubiquitin-60S ribosomal protein L40 OS=Pongo pygmae	PONPY	2
26	0	4,54	26,55999959	26,55999959	19,52999979	sp|P0C273|RL40_MACFA	Ubiquitin-60S ribosomal protein L40 OS=Macaca fascicu	MACFA	2
31	4	4	9,066999704	9,066999704	9,066999704	sp|Q9Y707|ACT2_SUIBO	Actin-2 OS=Suillus bovinus GN=ACT2 PE=2 SV=1	SUIBO	2
31	0	4	9,066999704	9,066999704	9,066999704	sp|Q9Y702|ACT1_SCHCO	Actin-1 OS=Schizophyllum commune GN=ACT1 PE=2 SV	SCHCO	2
31	0	4	9,066999704	9,066999704	9,066999704	sp|Q9UVX4|ACT_COPC7	Actin OS=Coprinopsis cinerea (strain Okayama-7 / 130 /	COPC7	2
31	0	4	9,066999704	9,066999704	9,066999704	sp|Q9UVW9|ACTG_ACRCH	Actin, gamma OS=Acremonium chrysogenum GN=ACT P	ACRCH	2
31	0	4	9,042999893	9,042999893	9,042999893	sp|Q964E3|ACTC_BIOAL	Actin, cytoplasmic OS=Biomphalaria alexandrina PE=3 S	BIOAL	2
31	0	4	9,042999893	9,042999893	9,042999893	sp|Q964E2|ACTC_BIOPF	Actin, cytoplasmic OS=Biomphalaria pfeifferi PE=3 SV=1	BIOPF	2
31	0	4	9,042999893	9,042999893	9,042999893	sp|Q964E1|ACTC_BIOOB	Actin, cytoplasmic OS=Biomphalaria obstructa PE=3 SV=	BIOOB	2
31	0	4	9,042999893	9,042999893	9,042999893	sp|Q964E0|ACTC_BIOTE	Actin, cytoplasmic OS=Biomphalaria tenagophila PE=3 S	BIOTE	2
31	0	4	9,042999893	9,042999893	9,042999893	sp|Q964D9|ACTC_PLATR	Actin, cytoplasmic OS=Planorbella trivolvis PE=3 SV=1	PLATR	2
31	0	4	9,066999704	9,066999704	9,066999704	sp|Q93131|ACTC_BRAFL	Actin, cytoplasmic OS=Branchiostoma floridae PE=2 SV=	BRAFL	2
31	0	4	9,066999704	9,066999704	9,066999704	sp|Q93129|ACTC_BRABE	Actin, cytoplasmic OS=Branchiostoma belcheri PE=2 SV=	BRABE	2
31	0	4	12,78000027	12,78000027	12,78000027	sp|Q92193|ACT_CRAVI	Actin (Fragment) OS=Crassostrea virginica PE=2 SV=1	CRAVI	2
31	0	4	9,066999704	9,066999704	9,066999704	sp|Q91ZK5|ACTB_SIGHI	Actin, cytoplasmic 1 OS=Sigmodon hispidus GN=ACTB P	SIGHI	2
31	0	4	9,066999704	9,066999704	9,066999704	sp|Q8X119|ACT_EXODE	Actin OS=Exophiala dermatitidis PE=3 SV=1	EXODE	2
31	0	4	9,066999704	9,066999704	9,066999704	sp|Q8JJB8|ACTG_TRISC	Actin, cytoplasmic 2 OS=Triakis scyllium GN=actg1 PE=2	TRISC	2

31	0	4	9,066999704	9,066999704	9,066999704	sp|Q7ZVI7|ACTB1_DANRE	Actin, cytoplasmic 1 OS=Danio rerio GN=actba PE=2 SV=	DANRE	2
31	0	4	9,066999704	9,066999704	9,066999704	sp|Q7ZVF9|ACTB2_DANRE	Actin, cytoplasmic 2 OS=Danio rerio GN=actbb PE=2 SV=	DANRE	2
31	0	4	9,066999704	9,066999704	9,066999704	sp|Q76N69|ACTB_CHLAE	Actin, cytoplasmic 1 OS=Chlorocebus aethiops GN=ACTE	CHLAE	2
31	0	4	9,066999704	9,066999704	9,066999704	sp|Q71FK5|ACTB_CAVPO	Actin, cytoplasmic 1 OS=Cavia porcellus GN=ACTB PE=2	CAVPO	2
31	0	4	9,066999704	9,066999704	9,066999704	sp|Q711N9|ACTB_MESAU	Actin, cytoplasmic 1 OS=Mesocricetus auratus GN=ACTE	MESAU	2
31	0	4	9,066999704	9,066999704	9,066999704	sp|Q6TCF2|ACT_GAEGA	Actin OS=Gaeumannomyces graminis var. avenae GN=A	GAEGA	2
31	0	4	9,066999704	9,066999704	9,066999704	sp|Q6QAQ1|ACTB_PIG	Actin, cytoplasmic 1 OS=Sus scrofa GN=ACTB PE=1 SV=2	PIG	2
31	0	4	9,066999704	9,066999704	9,066999704	sp|Q6P378|ACTG_XENTR	Actin, cytoplasmic 2 OS=Xenopus tropicalis GN=actg1 PE	XENTR	2
31	0	4	9,066999704	9,066999704	9,066999704	sp|Q6NVA9|ACTB_XENTR	Actin, cytoplasmic 1 OS=Xenopus tropicalis GN=actb PE=	XENTR	2
31	0	4	9,066999704	9,066999704	9,066999704	sp|Q5ZMQ2|ACTG_CHICK	Actin, cytoplasmic 2 OS=Gallus gallus GN=ACTG1 PE=1 S	CHICK	2
31	0	4	9,066999704	9,066999704	9,066999704	sp|Q5R6G0|ACTB_PONAB	Actin, cytoplasmic 1 OS=Pongo abelii GN=ACTB PE=2 SV	PONAB	2
31	0	4	9,066999704	9,066999704	9,066999704	sp|Q5R1X3|ACTB_PANTR	Actin, cytoplasmic 1 OS=Pan troglodytes GN=ACTB PE=2	PANTR	2
31	0	4	9,066999704	9,066999704	9,066999704	sp|Q5JAK2|ACTG_PELLE	Actin, cytoplasmic 2 OS=Pelophylax lessonae GN=actg1	PELLE	2
31	0	4	9,497000277	9,497000277	9,497000277	sp|Q55EU6|ACT23_DICDI	Putative actin-23 OS=Dictyostelium discoideum GN=act2	DICDI	2
31	0	4	9,042999893	9,042999893	9,042999893	sp|Q553U6|ACT22_DICDI	Putative actin-22 OS=Dictyostelium discoideum GN=act2	DICDI	2
31	0	4	9,042999893	9,042999893	9,042999893	sp|Q54GX7|ACT10_DICDI	Actin-10 OS=Dictyostelium discoideum GN=act10 PE=1 S	DICDI	2
31	0	4	9,066999704	9,066999704	9,066999704	sp|Q4R561|ACTB_MACFA	Actin, cytoplasmic 1 OS=Macaca fascicularis GN=ACTB P	MACFA	2
31	0	4	9,066999704	9,066999704	9,066999704	sp|Q4L0Y2|ACTB_SPECI	Actin, cytoplasmic 1 OS=Spermophilus citellus GN=ACTB	SPECI	2
31	0	4	9,042999893	9,042999893	9,042999893	sp|Q26065|ACT_PLAMG	Actin, adductor muscle OS=Placopecten magellanicus PE	PLAMG	2
31	0	4	8,995000273	8,995000273	8,995000273	sp|Q25472|ACT2_MOLOC	Actin, muscle-type OS=Molgula oculata PE=3 SV=1	MOLOC	2
31	0	4	9,042999893	9,042999893	9,042999893	sp|Q25010|ACT3A_HELAM	Actin, cytoplasmic A3a OS=Helicoverpa armigera GN=ac	HELAM	2
31	0	4	9,042999893	9,042999893	9,042999893	sp|Q07903|ACTC_STRPU	Actin, cytoskeletal 2A OS=Strongylocentrotus purpuratu	STRPU	2
31	0	4	10,99999994	10,99999994	10,99999994	sp|Q03342|ACT3_ECHGR	Actin-3 (Fragment) OS=Echinococcus granulosus GN=AC	ECHGR	2
31	0	4	9,066999704	9,066999704	9,066999704	sp|Q00215|ACTC_STYPL	Actin, cytoplasmic OS=Styela plicata PE=3 SV=1	STYPL	2
31	0	4	8,970999718	8,970999718	8,970999718	sp|Q00214|ACTM_STYPL	Actin, muscle OS=Styela plicata PE=3 SV=1	STYPL	2
31	0	4	9,042999893	9,042999893	9,042999893	sp|P92182|ACT1_LUMTE	Actin-1 OS=Lumbricus terrestris GN=ACT1 PE=2 SV=1	LUMTE	2
31	0	4	9,042999893	9,042999893	9,042999893	sp|P92179|ACTC_BIOGL	Actin, cytoplasmic OS=Biomphalaria glabrata PE=2 SV=2	BIOGL	2
31	0	4	9,042999893	9,042999893	9,042999893	sp|P92176|ACT2_LUMTE	Actin-2 OS=Lumbricus terrestris GN=ACT2 PE=2 SV=1	LUMTE	2
31	0	4	9,139999747	9,139999747	9,139999747	sp|P91754|ACT_LUMRU	Actin (Fragment) OS=Lumbricus rubellus PE=2 SV=1	LUMRU	2
31	0	4	9,042999893	9,042999893	9,042999893	sp|P90689|ACT_BRUMA	Actin OS=Brugia malayi PE=1 SV=1	BRUMA	2
31	0	4	9,418000281	9,418000281	9,418000281	sp|P84856|ACTB_CHLPG	Actin, cytoplasmic 1 OS=Chlorocebus pygerythrus GN=A	CHLPG	2
31	0	4	9,066999704	9,066999704	9,066999704	sp|P84336|ACTB_CAMDR	Actin, cytoplasmic 1 OS=Camelus dromedarius GN=ACTI	CAMDR	2
31	0	4	9,042999893	9,042999893	9,042999893	sp|P84185|ACT5C_ANOGA	Actin-5C OS=Anopheles gambiae GN=Act5C PE=2 SV=1	ANOGA	2
31	0	4	9,042999893	9,042999893	9,042999893	sp|P84184|ACT3B_HELAM	Actin-A3b, cytoplasmic OS=Helicoverpa armigera GN=ac	HELAM	2
31	0	4	9,042999893	9,042999893	9,042999893	sp|P84183|ACT4_BOMMO	Actin, cytoplasmic A4 OS=Bombyx mori GN=A4 PE=2 SV	BOMMO	2
31	0	4	9,042999893	9,042999893	9,042999893	sp|P83969|ACT1_BACDO	Actin, indirect flight muscle OS=Bactrocera dorsalis PE=3	BACDO	2
31	0	4	9,042999893	9,042999893	9,042999893	sp|P83968|ACT6_DROSI	Actin, indirect flight muscle OS=Drosophila simulans GN	DROSI	2
31	0	4	9,042999893	9,042999893	9,042999893	sp|P83967|ACT6_DROME	Actin, indirect flight muscle OS=Drosophila melanogaste	DROME	2
31	0	4	9,066999704	9,066999704	9,066999704	sp|P83751|ACTB_CTEID	Actin, cytoplasmic 1 OS=Ctenopharyngodon idella GN=a	CTEID	2
31	0	4	9,066999704	9,066999704	9,066999704	sp|P83750|ACTB_CYPCA	Actin, cytoplasmic 1 OS=Cyprinus carpio GN=actb PE=3 S	CYPCA	2
31	0	4	9,066999704	9,066999704	9,066999704	sp|P78711|ACT_NEUCR	Actin OS=Neurospora crassa (strain ATCC 24698 / 74-OR	NEUCR	2
31	0	4	9,042999893	9,042999893	9,042999893	sp|P69005|ACTD_STRPU	Actin, cytoskeletal 2B OS=Strongylocentrotus purpuratu	STRPU	2
31	0	4	9,042999893	9,042999893	9,042999893	sp|P69004|ACT2_STRFN	Actin-15B OS=Strongylocentrotus franciscanus PE=2 SV=	STRFN	2
31	0	4	9,042999893	9,042999893	9,042999893	sp|P69003|ACT1_HELTB	Actin CyI, cytoplasmic OS=Heliocidaris tuberculata PE=3	HELTB	2
31	0	4	9,042999893	9,042999893	9,042999893	sp|P69002|ACT1_HELER	Actin CyI, cytoplasmic OS=Heliocidaris erythrogramma F	HELER	2
31	0	4	9,042999893	9,042999893	9,042999893	sp|P68556|ACT1_DIPDE	Actin-1/4 OS=Diphyllobothrium dendriticum GN=ACT1 F	DIPDE	2
31	0	4	9,042999893	9,042999893	9,042999893	sp|P68555|ACT_TAESO	Actin OS=Taenia solium GN=ACT1 PE=3 SV=1	TAESO	2
31	0	4	9,066999704	9,066999704	9,066999704	sp|P68143|ACTB_OREMO	Actin, cytoplasmic 1 OS=Oreochromis mossambicus GN=	OREMO	2
31	0	4	9,066999704	9,066999704	9,066999704	sp|P68142|ACTB1_TAKRU	Actin, cytoplasmic 1 OS=Takifugu rubripes GN=actba PE	TAKRU	2
31	0	4	9,066999704	9,066999704	9,066999704	sp|P63261|ACTG_HUMAN	Actin, cytoplasmic 2 OS=Homo sapiens GN=ACTG1 PE=1	HUMAN	2
31	0	4	9,066999704	9,066999704	9,066999704	sp|P63260|ACTG_MOUSE	Actin, cytoplasmic 2 OS=Mus musculus GN=Actg1 PE=1 S	MOUSE	2
31	0	4	9,066999704	9,066999704	9,066999704	sp|P63259|ACTG_RAT	Actin, cytoplasmic 2 OS=Rattus norvegicus GN=Actg1 PE	RAT	2
31	0	4	9,066999704	9,066999704	9,066999704	sp|P63258|ACTG_BOVIN	Actin, cytoplasmic 2 OS=Bos taurus GN=ACTG1 PE=1 SV	BOVIN	2
31	0	4	9,066999704	9,066999704	9,066999704	sp|P63257|ACTG_TRIVU	Actin, cytoplasmic 2 OS=Trichosurus vulpecula GN=ACTC	TRIVU	2
31	0	4	9,066999704	9,066999704	9,066999704	sp|P63256|ACTG_ANSAN	Actin, cytoplasmic 2 OS=Anser anser anser GN=ACTG1 P	ANSAN	2
31	0	4	9,066999704	9,066999704	9,066999704	sp|P60713|ACTB_SHEEP	Actin, cytoplasmic 1 OS=Ovis aries GN=ACTB PE=2 SV=1	SHEEP	2
31	0	4	9,066999704	9,066999704	9,066999704	sp|P60712|ACTB_BOVIN	Actin, cytoplasmic 1 OS=Bos taurus GN=ACTB PE=1 SV=1	BOVIN	2
31	0	4	9,066999704	9,066999704	9,066999704	sp|P60711|ACTB_RAT	Actin, cytoplasmic 1 OS=Rattus norvegicus GN=Actb PE=	RAT	2
31	0	4	9,066999704	9,066999704	9,066999704	sp|P60710|ACTB_MOUSE	Actin, cytoplasmic 1 OS=Mus musculus GN=Actb PE=1 S	MOUSE	2
31	0	4	9,066999704	9,066999704	9,066999704	sp|P60709|ACTB_HUMAN	Actin, cytoplasmic 1 OS=Homo sapiens GN=ACTB PE=1 S	HUMAN	2
31	0	4	9,066999704	9,066999704	9,066999704	sp|P60708|ACTB_HORSE	Actin, cytoplasmic 1 OS=Equus caballus GN=ACTB PE=2 S	HORSE	2
31	0	4	9,066999704	9,066999704	9,066999704	sp|P60707|ACTB_TRIVU	Actin, cytoplasmic 1 OS=Trichosurus vulpecula GN=ACTE	TRIVU	2
31	0	4	9,066999704	9,066999704	9,066999704	sp|P60706|ACTB_CHICK	Actin, cytoplasmic 1 OS=Gallus gallus GN=ACTB PE=1 SV	CHICK	2
31	0	4	9,066999704	9,066999704	9,066999704	sp|P53689|ACT_PHARH	Actin OS=Phaffia rhodozyma PE=3 SV=1	PHARH	2
31	0	4	9,042999893	9,042999893	9,042999893	sp|P53506|ACT8_XENLA	Actin, cytoplasmic type 8 OS=Xenopus laevis PE=3 SV=1	XENLA	2
31	0	4	9,042999893	9,042999893	9,042999893	sp|P53505|ACT5_XENLA	Actin, cytoplasmic type 5 OS=Xenopus laevis PE=3 SV=1	XENLA	2
31	0	4	9,042999893	9,042999893	9,042999893	sp|P53501|ACT3_DROME	Actin-57B OS=Drosophila melanogaster GN=Act57B PE=	DROME	2
31	0	4	9,066999704	9,066999704	9,066999704	sp|P53486|ACTB3_TAKRU	Actin, cytoplasmic 3 OS=Takifugu rubripes GN=actbc PE	TAKRU	2
31	0	4	9,066999704	9,066999704	9,066999704	sp|P53485|ACTB2_TAKRU	Actin, cytoplasmic 2 OS=Takifugu rubripes GN=actbb PE	TAKRU	2
31	0	4	9,042999893	9,042999893	9,042999893	sp|P53478|ACT5_CHICK	Actin, cytoplasmic type 5 OS=Gallus gallus PE=3 SV=1	CHICK	2
31	0	4	9,042999893	9,042999893	9,042999893	sp|P53474|ACTE_STRPU	Actin, cytoskeletal 3A OS=Strongylocentrotus purpuratu	STRPU	2
31	0	4	9,042999893	9,042999893	9,042999893	sp|P53473|ACTB_STRPU	Actin, cytoskeletal 1B OS=Strongylocentrotus purpuratu	STRPU	2
31	0	4	9,042999893	9,042999893	9,042999893	sp|P53472|ACTA_STRPU	Actin, cytoskeletal 1A OS=Strongylocentrotus purpuratu	STRPU	2
31	0	4	9,042999893	9,042999893	9,042999893	sp|P53471|ACT2_SCHMA	Actin-2 OS=Schistosoma mansoni PE=2 SV=1	SCHMA	2
31	0	4	9,042999893	9,042999893	9,042999893	sp|P53470|ACT1_SCHMA	Actin-1 OS=Schistosoma mansoni PE=2 SV=1	SCHMA	2
31	0	4	9,042999893	9,042999893	9,042999893	sp|P53466|ACT2_LYTPI	Actin, cytoskeletal 2 OS=Lytechinus pictus PE=2 SV=1	LYTPI	2
31	0	4	9,042999893	9,042999893	9,042999893	sp|P53465|ACT1_LYTPI	Actin, cytoskeletal 1 OS=Lytechinus pictus PE=2 SV=1	LYTPI	2
31	0	4	9,042999893	9,042999893	9,042999893	sp|P53464|ACTM_HELTB	Actin, cytoskeletal OS=Heliocidaris tuberculata PE=3 SV	HELTB	2
31	0	4	9,042999893	9,042999893	9,042999893	sp|P53463|ACTM_HELER	Actin, cytoskeletal OS=Heliocidaris erythrogramma PE=3	HELER	2
31	0	4	9,164000303	9,164000303	9,164000303	sp|P53458|ACT5_DIPDE	Actin-5 (Fragment) OS=Diphyllobothrium dendriticum G	DIPDE	2
31	0	4	9,042999893	9,042999893	9,042999893	sp|P53456|ACT2_DIPDE	Actin-2 OS=Diphyllobothrium dendriticum GN=ACT2 PE	DIPDE	2
31	0	4	9,066999704	9,066999704	9,066999704	sp|P53455|ACT_AJECG	Actin OS=Ajellomyces capsulatus (strain G186AR / H82 /	AJECG	2
31	0	4	9,066999704	9,066999704	9,066999704	sp|P50138|ACT_PUCGR	Actin OS=Puccinia graminis PE=3 SV=1	PUCGR	2
31	0	4	9,042999893	9,042999893	9,042999893	sp|P49871|ACT_MANSE	Actin, muscle OS=Manduca sexta PE=2 SV=1	MANSE	2
31	0	4	9,042999893	9,042999893	9,042999893	sp|P49128|ACT1_AEDAE	Actin-1 OS=Aedes aegypti GN=ACT-1 PE=2 SV=2	AEDAE	2
31	0	4	9,066999704	9,066999704	9,066999704	sp|P48975|ACTB_CRIGR	Actin, cytoplasmic 1 OS=Cricetulus griseus GN=ACTB PE	CRIGR	2
31	0	4	9,066999704	9,066999704	9,066999704	sp|P48465|ACT_CRYNH	Actin OS=Cryptococcus neoformans var. grubii serotype	CRYNH	2
31	0	4	9,042999893	9,042999893	9,042999893	sp|P45887|ACT5_BACDO	Actin-5, muscle-specific OS=Bactrocera dorsalis PE=2 SV	BACDO	2
31	0	4	9,042999893	9,042999893	9,042999893	sp|P45886|ACT3_BACDO	Actin-3, muscle-specific OS=Bactrocera dorsalis PE=2 SV	BACDO	2
31	0	4	9,042999893	9,042999893	9,042999893	sp|P45885|ACT2_BACDO	Actin-2, muscle-specific OS=Bactrocera dorsalis PE=2 SV	BACDO	2
31	0	4	9,042999893	9,042999893	9,042999893	sp|P41341|ACTY_LIMPO	Actin-11 OS=Limulus polyphemus PE=2 SV=1	LIMPO	2
31	0	4	9,042999893	9,042999893	9,042999893	sp|P41340|ACT3_LIMPO	Actin-3 OS=Limulus polyphemus PE=1 SV=1	LIMPO	2
31	0	4	9,042999893	9,042999893	9,042999893	sp|P41339|ACTA_LIMPO	Actin, acrosomal process isoform OS=Limulus polyphem	LIMPO	2
31	0	4	9,042999893	9,042999893	9,042999893	sp|P41113|ACT3_PODCA	Actin-3 OS=Podocoryna carnea GN=ACT3 PE=3 SV=1	PODCA	2
31	0	4	9,042999893	9,042999893	9,042999893	sp|P41112|ACT1_PODCA	Actin-1/2 OS=Podocoryna carnea GN=ACTIA PE=2 SV=1	PODCA	2
31	0	4	9,066999704	9,066999704	9,066999704	sp|P20359|ACTG_EMENI	Actin, gamma OS=Emericella nidulans (strain FGSC A4 /	EMENI	2
31	0	4	9,042999893	9,042999893	9,042999893	sp|P18603|ACT4_ARTSX	Actin, clone 403 OS=Artemia sp. PE=2 SV=1	ARTSX	2
31	0	4	9,042999893	9,042999893	9,042999893	sp|P18499|ACTF_STRPU	Actin, cytoskeletal 3B OS=Strongylocentrotus purpuratu	STRPU	2
31	0	4	9,042999893	9,042999893	9,042999893	sp|P17126|ACT_HYDVU	Actin, non-muscle 6.2 OS=Hydra vulgaris PE=3 SV=1	HYDVU	2
31	0	4	9,042999893	9,042999893	9,042999893	sp|P15475|ACTB_XENBO	Actin, cytoplasmic 1 OS=Xenopus borealis GN=actb PE=3	XENBO	2
31	0	4	9,042999893	9,042999893	9,042999893	sp|P12717|ACTM_PISOC	Actin, muscle OS=Pisaster ochraceus PE=3 SV=1	PISOC	2
31	0	4	9,042999893	9,042999893	9,042999893	sp|P12716|ACTC_PISOC	Actin, cytoplasmic OS=Pisaster ochraceus PE=3 SV=1	PISOC	2
31	0	4	9,091000259	9,091000259	9,091000259	sp|P12431|ACTM_STRPU	Actin, muscle OS=Strongylocentrotus purpuratus PE=3 S	STRPU	2

31	0	4	9,042999893	9,042999893	9,042999893	sp|P10990|ACT1_STRFN	Actin-15A OS=Strongylocentrotus franciscanus PE=3 SV=	STRFN	2
31	0	4	9,042999893	9,042999893	9,042999893	sp|P10987|ACT1_DROME	Actin-5C OS=Drosophila melanogaster GN=Act5C PE=1 S	DROME	2
31	0	4	9,042999893	9,042999893	9,042999893	sp|P10981|ACT5_DROME	Actin-87E OS=Drosophila melanogaster GN=Act87E PE=:	DROME	2
31	0	4	9,066999704	9,066999704	9,066999704	sp|P10365|ACT_THELA	Actin OS=Thermomyces lanuginosus PE=3 SV=1	THELA	2
31	0	4	9,042999893	9,042999893	9,042999893	sp|P07836|ACT1_BOMMO	Actin, muscle-type A1 OS=Bombyx mori PE=3 SV=1	BOMMO	2
31	0	4	9,042999893	9,042999893	9,042999893	sp|P07830|ACT1_DICDI	Major actin OS=Dictyostelium discoideum GN=act1 PE=:	DICDI	2
31	0	4	9,042999893	9,042999893	9,042999893	sp|P07829|ACT3_DICDI	Actin-3 OS=Dictyostelium discoideum GN=act3 PE=3 SV:	DICDI	2
31	0	4	9,042999893	9,042999893	9,042999893	sp|P04829|ACT3_BOMMO	Actin, cytoplasmic A3 OS=Bombyx mori PE=3 SV=3	BOMMO	2
31	0	4	9,066999704	9,066999704	9,066999704	sp|P02578|ACT1_ACACA	Actin-1 OS=Acanthamoeba castellanii PE=1 SV=1	ACACA	2
31	0	4	9,042999893	9,042999893	9,042999893	sp|P02576|ACTA_PHYPO	Actin, plasmodial isoform OS=Physarum polycephalum (	PHYPO	2
31	0	4	9,042999893	9,042999893	9,042999893	sp|P02574|ACT4_DROME	Actin, larval muscle OS=Drosophila melanogaster GN=Ac	DROME	2
31	0	4	9,042999893	9,042999893	9,042999893	sp|P02572|ACT2_DROME	Actin-42A OS=Drosophila melanogaster GN=Act42A PE=	DROME	2
31	0	4	9,066999704	9,066999704	9,066999704	sp|O93400|ACTB_XENLA	Actin, cytoplasmic 1 OS=Xenopus laevis GN=actb PE=2 S	XENLA	2
31	0	4	9,019000083	9,019000083	9,019000083	sp|O65316|ACT_MESVI	Actin OS=Mesostigma viride PE=3 SV=1	MESVI	2
31	0	4	9,066999704	9,066999704	9,066999704	sp|O42161|ACTB_SALSA	Actin, cytoplasmic 1 OS=Salmo salar GN=actb PE=2 SV=:	SALSA	2
31	0	4	9,066999704	9,066999704	9,066999704	sp|O18840|ACTB_CANLF	Actin, cytoplasmic 1 OS=Canis lupus familiaris GN=ACTB	CANLF	2
31	0	4	9,042999893	9,042999893	9,042999893	sp|O18500|ACT2_SACKO	Actin-2 OS=Saccoglossus kowalevskii PE=2 SV=1	SACKO	2
31	0	4	9,042999893	9,042999893	9,042999893	sp|O18499|ACT1_SACKO	Actin-1 OS=Saccoglossus kowalevskii PE=2 SV=1	SACKO	2
31	0	4	9,042999893	9,042999893	9,042999893	sp|O17320|ACT_CRAGI	Actin OS=Crassostrea gigas PE=2 SV=1	CRAGI	2
31	0	4	9,042999893	9,042999893	9,042999893	sp|O16808|ACT_MAYDE	Actin OS=Mayetiola destructor PE=2 SV=1	MAYDE	2
31	0	4	9,066999704	9,066999704	9,066999704	sp|O13419|ACT_BOTFU	Actin OS=Botryotinia fuckeliana GN=actA PE=3 SV=1	BOTFU	2
31	0	4	9,066999704	9,066999704	9,066999704	sp|A2BDB0|ACTG_XENLA	Actin, cytoplasmic 2 OS=Xenopus laevis GN=actg1 PE=2	XENLA	2
32	3,49	3,49	25,49000084	21,0800007	16,17999971	sp|O82803|SRPP_HEVBR	Small rubber particle protein OS=Hevea brasiliensis GN=	HEVBR	2
33	3,19	3,19	35,85000038	26,4200002	26,4200002	sp|Q5R9M3|THIO_PONAB	Thioredoxin OS=Pongo abelii GN=TXN PE=3 SV=3	PONAB	2
33	0	3,19	36,19000018	26,66999996	26,66999996	sp|P10599|THIO_HUMAN	Thioredoxin OS=Homo sapiens GN=TXN PE=1 SV=3	HUMAN	2
34	3,08	3,08	9,933000058	2,952999994	2,952999994	sp|Q8SPJ1|PLAK_BOVIN	Junction plakoglobin OS=Bos taurus GN=JUP PE=2 SV=1	BOVIN	2
34	0	3,08	9,933000058	2,952999994	2,952999994	sp|Q6P0K8|PLAK_RAT	Junction plakoglobin OS=Rattus norvegicus GN=Jup PE=:	RAT	2
34	0	3,08	9,933000058	2,952999994	2,952999994	sp|Q02257|PLAK_MOUSE	Junction plakoglobin OS=Mus musculus GN=Jup PE=1 SV	MOUSE	2
34	0	3,08	9,933000058	2,952999994	2,952999994	sp|P14923|PLAK_HUMAN	Junction plakoglobin OS=Homo sapiens GN=JUP PE=1 SV	HUMAN	2
34	0	3,08	7,248000056	2,952999994	2,952999994	sp|Q8WNW3|PLAK_PIG	Junction plakoglobin OS=Sus scrofa GN=Jup PE=2 SV=1	PIG	2
39	2	4,03	15,89999944	7,951000333	7,951000333	sp|P01861|IGHG4_HUMAN	Ig gamma-4 chain C region OS=Homo sapiens GN=IGHG	HUMAN	2
39	0	4,03	13,78999949	6,897000223	6,897000223	sp|P01860|IGHG3_HUMAN	Ig gamma-3 chain C region OS=Homo sapiens GN=IGHG:	HUMAN	2
39	0	4,01	11,03999987	7,975000143	7,975000143	sp|P01859|IGHG2_HUMAN	Ig gamma-2 chain C region OS=Homo sapiens GN=IGHG:	HUMAN	2
40	2	2,78	4,68400009	4,68400009	4,68400009	sp|Q6IFX1|K1C24_RAT	Keratin, type I cytoskeletal 24 OS=Rattus norvegicus GN	RAT	2
40	0	2,78	4,492000118	4,492000118	4,492000118	sp|A1L317|K1C24_MOUSE	Keratin, type I cytoskeletal 24 OS=Mus musculus GN=Kr	MOUSE	2
56	1,44	1,44	16,11000001	16,11000001	16,11000001	sp|Q15828|CYTM_HUMAN	Cystatin-M OS=Homo sapiens GN=CST6 PE=1 SV=1	HUMAN	2
26	0	2,47	21,66000009	21,66000009	11,81999967	sp|P42740|UBIQP_AGLNE	Polyubiquitin OS=Aglaothamnion neglectum PE=2 SV=2	AGLNE	1
26	0	2,47	23,3799994	23,3799994	11,68999997	sp|Q8SWD4|UBIQ_ENCCU	Ubiquitin OS=Encephalitozoon cuniculi (strain GB-M1) G	ENCCU	1
26	0	2,47	14,05999959	14,05999959	7,03099966	sp|P69201|RL40_LEIMA	Ubiquitin-60S ribosomal protein L40 OS=Leishmania ma	LEIMA	1
26	0	2,47	14,05999959	14,05999959	7,03099966	sp|P69200|RL40_LEITA	Ubiquitin-60S ribosomal protein L40 OS=Leishmania tar	LEITA	1
26	0	2,47	11,99999973	11,99999973	5,999999866	sp|P69061|RS27A_KLULA	Ubiquitin-40S ribosomal protein S27a OS=Kluyveromyce	KLULA	1
26	0	2,47	21,66000009	21,66000009	9,847000241	sp|P59669|UBIQP_GEOCY	Polyubiquitin OS=Geodia cydonium PE=2 SV=2	GEOCY	1
26	0	2,47	14,05999959	14,05999959	7,03099966	sp|P49632|RL40_CAEEL	Ubiquitin-60S ribosomal protein L40 OS=Caenorhabditis	CAEEL	1
26	0	2,47	13,9500007	13,9500007	6,977000087	sp|P46575|RL40_EIMBO	Ubiquitin-60S ribosomal protein L40 OS=Eimeria bovis P	EIMBO	1
26	0	2,47	13,9500007	13,9500007	6,977000087	sp|P40909|RL40_CRYNJ	Ubiquitin-60S ribosomal protein L40 OS=Cryptococcus n	CRYNJ	1
26	0	2,47	13,9500007	13,9500007	6,977000087	sp|P33190|RL40_TETPY	Ubiquitin-60S ribosomal protein L40 OS=Tetrahymena p	TETPY	1
26	0	2,47	27,07000077	27,07000077	13,52999955	sp|P23398|UBIQP_STRPU	Polyubiquitin (Fragment) OS=Strongylocentrotus purpur	STRPU	1
26	0	2,47	23,5799998	23,5799998	11,7899999	sp|P22589|UBIQP_PHYIN	Polyubiquitin OS=Phytophthora infestans PE=1 SV=2	PHYIN	1
26	0	2,47	23,68000001	23,68000001	11,84	sp|P19848|UBIQ_COPCO	Ubiquitin OS=Coprinellus congregatus PE=1 SV=1	COPCO	1
26	0	2,47	11,6899997	11,6899997	5,843999982	sp|P14799|RS27A_NEUCR	Ubiquitin-40S ribosomal protein S27a OS=Neurospora cr	NEUCR	1
26	0	2,47	11,6899997	11,6899997	5,843999982	sp|P14797|RS27A_DICDI	Ubiquitin-40S ribosomal protein S27a OS=Dictyostelium	DICDI	1
26	0	2,47	14,05999959	14,05999959	7,03099966	sp|P14794|RL40_DICDI	Ubiquitin-60S ribosomal protein L40 OS=Dictyostelium c	DICDI	1
26	0	2,47	13,9500007	13,9500007	6,977000087	sp|P0DJ25|RL40_TETTS	Ubiquitin-60S ribosomal protein L40 OS=Tetrahymena tl	TETTS	1
26	0	2,47	14,05999959	14,05999959	7,03099966	sp|P0CH09|RL402_YEAST	Ubiquitin-60S ribosomal protein L40 OS=Saccharomyces	YEAST	1
26	0	2,47	14,05999959	14,05999959	7,03099966	sp|P0CH08|RL401_YEAST	Ubiquitin-60S ribosomal protein L40 OS=Saccharomyces	YEAST	1
26	0	2,47	14,05999959	14,05999959	7,03099966	sp|P0CH07|RL402_SCHPO	Ubiquitin-60S ribosomal protein L40 OS=Schizosaccharo	SCHPO	1
26	0	2,47	14,05999959	14,05999959	7,03099966	sp|P0CH06|RL401_SCHPO	Ubiquitin-60S ribosomal protein L40 OS=Schizosaccharo	SCHPO	1
26	0	2,47	23,61000031	23,61000031	11,80000007	sp|P0CG88|UBIQJ_DICDI	Polyubiquitin-J OS=Dictyostelium discoideum GN=ubqJ I	DICDI	1
26	0	2,47	23,62000048	23,62000048	11,81000024	sp|P0CG82|UBIQP_TETPY	Polyubiquitin OS=Tetrahymena pyriformis GN=TU20 PE=	TETPY	1
26	0	2,47	23,62000048	23,62000048	11,81000024	sp|P0CG81|UBIQH_DICDI	Polyubiquitin-H OS=Dictyostelium discoideum GN=ubqH	DICDI	1
26	0	2,47	23,61000031	23,61000031	11,80000007	sp|P0CG80|UBIQI_DICDI	Polyubiquitin-I OS=Dictyostelium discoideum GN=ubqI F	DICDI	1
26	0	2,47	23,62000048	23,62000048	11,81000024	sp|P0CG79|UBIQG_DICDI	Polyubiquitin-G OS=Dictyostelium discoideum GN=ubqG	DICDI	1
26	0	2,47	23,63999933	23,63999933	11,81999967	sp|P0CG78|UBIQF_DICDI	Polyubiquitin-F OS=Dictyostelium discoideum GN=ubqF	DICDI	1
26	0	2,47	23,5799998	23,5799998	11,7899999	sp|P0CG77|UBIQD_DICDI	Polyubiquitin-D OS=Dictyostelium discoideum GN=ubqD	DICDI	1
26	0	2,47	23,62000048	23,62000048	11,81000024	sp|P0CG76|UBIQA_DICDI	Polyubiquitin-A OS=Dictyostelium discoideum GN=ubqA	DICDI	1
26	0	2,47	23,62000048	23,62000048	11,81000024	sp|P0CG75|UBI4P_KLULA	Polyubiquitin OS=Kluyveromyces lactis (strain ATCC 858	KLULA	1
26	0	2,47	23,61000031	23,61000031	11,80000007	sp|P0CG74|UBI4P_CANAX	Polyubiquitin OS=Candida albicans GN=UBI4 PE=1 SV=1	CANAX	1
26	0	2,47	23,5799998	23,5799998	11,7899999	sp|P0CG73|UBI1P_CANAX	Polyubiquitin OS=Candida albicans GN=UBI1 PE=1 SV=1	CANAX	1
26	0	2,47	23,55999947	23,55999947	11,77999973	sp|P0CG72|UBI4P_SCHPO	Polyubiquitin OS=Schizosaccharomyces pombe (strain 9	SCHPO	1
26	0	2,47	23,63000065	23,63000065	11,81000024	sp|P0CG71|UBIQ1_CAEEL	Polyubiquitin-A OS=Caenorhabditis elegans GN=ubq-1 P	CAEEL	1
26	0	2,47	23,61000031	23,61000031	11,80000007	sp|P0CG70|UBI4P_NEUCR	Polyubiquitin OS=Neurospora crassa (strain ATCC 24698	NEUCR	1
26	0	2,47	23,62000048	23,62000048	11,81000024	sp|P0CG63|UBI4P_YEAST	Polyubiquitin OS=Saccharomyces cerevisiae (strain ATC(	YEAST	1
26	0	2,47	11,99999973	11,99999973	5,999999866	sp|P0C8R3|RS27B_SCHPO	Ubiquitin-40S ribosomal protein S27b OS=Schizosacchar	SCHPO	1
26	0	2,47	14,05999959	14,05999959	7,03099966	sp|P0C224|RL40_NEUCR	Ubiquitin-60S ribosomal protein L40 OS=Neurospora cra	NEUCR	1
26	0	2,47	11,99999973	11,99999973	5,999999866	sp|P0C016|RS27A_SCHPO	Ubiquitin-40S ribosomal protein S27a OS=Schizosacchar	SCHPO	1
26	0	2,47	11,84	11,84	5,92099987	sp|P05759|RS27A_YEAST	Ubiquitin-40S ribosomal protein S31 OS=Saccharomyces	YEAST	1
30	0	2	3,460000083	3,460000083	3,460000083	sp|P14639|ALBU_SHEEP	Serum albumin OS=Ovis aries GN=ALB PE=1 SV=1	SHEEP	1
31	0	2,01	9,042999893	4,255000129	4,255000129	sp|Q8BFZ3|ACTBL_MOUSE	Beta-actin-like protein 2 OS=Mus musculus GN=Actbl2 P	MOUSE	1
31	0	2,01	9,042999893	4,255000129	4,255000129	sp|Q562R1|ACTBL_HUMAN	Beta-actin-like protein 2 OS=Homo sapiens GN=ACTBL2	HUMAN	1
31	0	2	2,697999962	1,487999968	1,487999968	sp|Q6S8J3|POTEE_HUMAN	POTE ankyrin domain family member E OS=Homo sapier	HUMAN	1
31	0	2	2,697999962	1,487999968	1,487999968	sp|A5A3E0|POTEF_HUMAN	POTE ankyrin domain family member F OS=Homo sapier	HUMAN	1
31	0	2	4,800000042	4,800000042	4,800000042	sp|Q9Y896|ACT2_SCHCO	Actin-2 OS=Schizophyllum commune GN=ACT2 PE=3 SV:	SCHCO	1
31	0	2	4,800000042	4,800000042	4,800000042	sp|Q9Y701|ACT1_SUIBO	Actin-1 OS=Suillus bovinus GN=ACT1 PE=2 SV=1	SUIBO	1
31	0	2	4,255000129	4,255000129	4,255000129	sp|Q9UVZ8|ACT_CANDC	Actin OS=Candida dubliniensis (strain CD36 / ATCC MYA	CANDC	1
31	0	2	4,800000042	4,800000042	4,800000042	sp|Q9UVF3|ACT_YARLI	Actin OS=Yarrowia lipolytica (strain CLIB 122 / E 150) GN	YARLI	1
31	0	2	4,267000034	4,267000034	4,267000034	sp|Q9URS0|ACTG_PENCH	Actin, gamma OS=Penicillium chrysogenum GN=ACT PE:	PENCH	1
31	0	2	4,255000129	4,255000129	4,255000129	sp|Q9P4D1|ACT_PICPG	Actin OS=Komagataella pastoris (strain GS115 / ATCC 20	PICPG	1
31	0	2	4,267000034	4,267000034	4,267000034	sp|Q9BYX7|ACTBM_HUMAN	Putative beta-actin-like protein 3 OS=Homo sapiens GN=	HUMAN	1
31	0	2	4,786999896	4,786999896	4,786999896	sp|Q99023|ACT_HYPJE	Actin OS=Hypocrea jecorina GN=act PE=3 SV=1	HYPJE	1
31	0	2	4,244000092	4,244000092	4,244000092	sp|Q98972|ACTS_ORYLA	Actin, alpha skeletal muscle OS=Oryzias latipes GN=acta	ORYLA	1
31	0	2	5,356999859	5,356999859	5,356999859	sp|Q96483|ACT2_SOLLC	Actin-51 (Fragment) OS=Solanum lycopersicum PE=3 SV	SOLLC	1
31	0	2	5,356999859	5,356999859	5,356999859	sp|Q96482|ACT1_SOLLC	Actin-41 (Fragment) OS=Solanum lycopersicum PE=3 SV	SOLLC	1
31	0	2	4,233000055	4,233000055	4,233000055	sp|Q93132|ACTM_BRAFL	Actin, muscle OS=Branchiostoma floridae PE=2 SV=1	BRAFL	1
31	0	2	4,222000018	4,222000018	4,222000018	sp|Q93130|ACTM_BRABE	Actin, muscle OS=Branchiostoma belcheri PE=2 SV=1	BRABE	1
31	0	2	5,754999816	5,754999816	5,754999816	sp|Q92192|ACT_CALFI	Actin (Fragment) OS=Calanus finmarchicus PE=2 SV=1	CALFI	1
31	0	2	4,244000092	4,244000092	4,244000092	sp|Q90X97|ACTS_ATRMM	Actin, alpha skeletal muscle OS=Atractaspis microlepido	ATRMM	1
31	0	2	4,800000042	4,800000042	4,800000042	sp|Q8SWN8|ACT_ENCCU	Actin OS=Encephalitozoon cuniculi (strain GB-M1) GN=E	ENCCU	1
31	0	2	4,255000129	4,255000129	4,255000129	sp|Q75D00|ACT_ASHGO	Actin OS=Ashbya gossypii (strain ATCC 10895 / CBS 109.	ASHGO	1
31	0	2	4,244000092	4,244000092	4,244000092	sp|Q6P8G3|ACT3_XENTR	Actin, alpha sarcomeric/skeletal OS=Xenopus tropicalis (	XENTR	1
31	0	2	4,244000092	4,244000092	4,244000092	sp|Q6P640|ACTC_XENTR	Actin, alpha cardiac muscle 1 OS=Xenopus tropicalis GN	XENTR	1
31	0	2	4,244000092	4,244000092	4,244000092	sp|Q5R9Q5|ACTS_PONAB	Actin, alpha skeletal muscle OS=Pongo abelii GN=ACTA1	PONAB	1
31	0	2	4,255000129	4,255000129	4,255000129	sp|Q5E9B5|ACTH_BOVIN	Actin, gamma-enteric smooth muscle OS=Bos taurus GN	BOVIN	1
31	0	2	4,156000167	4,156000167	4,156000167	sp|Q54HF0|ACT25_DICDI	Putative actin-25 OS=Dictyostelium discoideum GN=act2	DICDI	1

31	0	2	4,244000092	4,244000092	4,244000092	sp|Q3ZC07|ACTC_BOVIN	Actin, alpha cardiac muscle 1 OS=Bos taurus GN=ACTC1	BOVIN	1
31	0	2	4,786999896	4,786999896	4,786999896	sp|Q39758|ACT_FUCVE	Actin OS=Fucus vesiculosus PE=2 SV=1	FUCVE	1
31	0	2	21,17999941	21,17999941	21,17999941	sp|Q39596|ACT_OXYRB	Actin (Fragment) OS=Oxybasis rubra PE=3 SV=1	OXYRB	1
31	0	2	9,301999956	9,301999956	9,301999956	sp|Q25381|ACTM_LYTPI	Actin, muscle (Fragment) OS=Lytechinus pictus PE=3 SV	LYTPI	1
31	0	2	9,301999956	9,301999956	9,301999956	sp|Q25379|ACT3_LYTPI	Actin, cytoskeletal 3 (Fragment) OS=Lytechinus pictus PI	LYTPI	1
31	0	2	10,98000035	10,98000035	10,98000035	sp|Q11212|ACT_SPOLI	Actin (Fragment) OS=Spodoptera littoralis PE=2 SV=1	SPOLI	1
31	0	2	4,774999991	4,774999991	4,774999991	sp|Q10DV7|ACT1_ORYSJ	Actin-1 OS=Oryza sativa subsp. japonica GN=ACT1 PE=2	ORYSJ	1
31	0	2	4,267000034	4,267000034	4,267000034	sp|Q0PGG4|ACTB_BOSMU	Actin, cytoplasmic 1 OS=Bos mutus grunniens GN=ACTB	BOSMU	1
31	0	2	4,774999991	4,774999991	4,774999991	sp|Q05214|ACT1_TOBAC	Actin OS=Nicotiana tabacum PE=3 SV=1	TOBAC	1
31	0	2	4,786999896	4,786999896	4,786999896	sp|Q03341|ACT2_ECHGR	Actin-2 OS=Echinococcus granulosus GN=ACTII PE=3 SV=	ECHGR	1
31	0	2	5,421999842	5,421999842	5,421999842	sp|P93587|ACT1_SOLTU	Actin-42 (Fragment) OS=Solanum tuberosum PE=3 SV=1	SOLTU	1
31	0	2	5,356999859	5,356999859	5,356999859	sp|P93584|ACT9_SOLTU	Actin-82 (Fragment) OS=Solanum tuberosum PE=3 SV=1	SOLTU	1
31	0	2	5,356999859	5,356999859	5,356999859	sp|P93375|ACT7_TOBAC	Actin-104 (Fragment) OS=Nicotiana tabacum PE=3 SV=1	TOBAC	1
31	0	2	5,310000107	5,310000107	5,310000107	sp|P93373|ACT3_TOBAC	Actin-54 (Fragment) OS=Nicotiana tabacum PE=3 SV=1	TOBAC	1
31	0	2	8,332999796	8,332999796	8,332999796	sp|P86700|ACT_CHIOP	Actin, muscle (Fragments) OS=Chionoecetes opilio PE=1	CHIOP	1
31	0	2	40,0000006	40,0000006	40,0000006	sp|P85911|ACT1_PSEMZ	Actin-1 (Fragment) OS=Pseudotsuga menziesii PE=1 SV=	PSEMZ	1
31	0	2	5,356999859	5,356999859	5,356999859	sp|P81229|ACT8_SOLTU	Actin-79 (Fragment) OS=Solanum tuberosum PE=3 SV=1	SOLTU	1
31	0	2	5,356999859	5,356999859	5,356999859	sp|P81228|ACT5_SOLTU	Actin-66 (Fragment) OS=Solanum tuberosum PE=3 SV=1	SOLTU	1
31	0	2	4,267000034	4,267000034	4,267000034	sp|P79818|ACTB_ORYLA	Actin, cytoplasmic 1 OS=Oryzias latipes GN=actb PE=2 S	ORYLA	1
31	0	2	4,244000092	4,244000092	4,244000092	sp|P68264|ACTS_OREMO	Actin, alpha skeletal muscle OS=Oreochromis mossambi	OREMO	1
31	0	2	4,244000092	4,244000092	4,244000092	sp|P68140|ACTSA_TAKRU	Actin, alpha skeletal muscle A OS=Takifugu rubripes GN	TAKRU	1
31	0	2	4,244000092	4,244000092	4,244000092	sp|P68139|ACTS_CHICK	Actin, alpha skeletal muscle OS=Gallus gallus GN=ACTA1	CHICK	1
31	0	2	4,244000092	4,244000092	4,244000092	sp|P68138|ACTS_BOVIN	Actin, alpha skeletal muscle OS=Bos taurus GN=ACTA1 F	BOVIN	1
31	0	2	4,244000092	4,244000092	4,244000092	sp|P68137|ACTS_PIG	Actin, alpha skeletal muscle OS=Sus scrofa GN=ACTA1 P	PIG	1
31	0	2	4,244000092	4,244000092	4,244000092	sp|P68136|ACTS_RAT	Actin, alpha skeletal muscle OS=Rattus norvegicus GN=A	RAT	1
31	0	2	4,244000092	4,244000092	4,244000092	sp|P68135|ACTS_RABIT	Actin, alpha skeletal muscle OS=Oryctolagus cuniculus G	RABIT	1
31	0	2	4,244000092	4,244000092	4,244000092	sp|P68134|ACTS_MOUSE	Actin, alpha skeletal muscle OS=Mus musculus GN=Acta	MOUSE	1
31	0	2	4,244000092	4,244000092	4,244000092	sp|P68133|ACTS_HUMAN	Actin, alpha skeletal muscle OS=Homo sapiens GN=ACTA	HUMAN	1
31	0	2	4,244000092	4,244000092	4,244000092	sp|P68035|ACTC_RAT	Actin, alpha cardiac muscle 1 OS=Rattus norvegicus GN=	RAT	1
31	0	2	4,244000092	4,244000092	4,244000092	sp|P68034|ACTC_CHICK	Actin, alpha cardiac muscle 1 OS=Gallus gallus GN=ACTC	CHICK	1
31	0	2	4,244000092	4,244000092	4,244000092	sp|P68033|ACTC_MOUSE	Actin, alpha cardiac muscle 1 OS=Mus musculus GN=Act	MOUSE	1
31	0	2	4,244000092	4,244000092	4,244000092	sp|P68032|ACTC_HUMAN	Actin, alpha cardiac muscle 1 OS=Homo sapiens GN=ACT	HUMAN	1
31	0	2	4,255000129	4,255000129	4,255000129	sp|P63270|ACTH_CHICK	Actin, gamma-enteric smooth muscle OS=Gallus gallus G	CHICK	1
31	0	2	4,255000129	4,255000129	4,255000129	sp|P63269|ACTH_RAT	Actin, gamma-enteric smooth muscle OS=Rattus norveg	RAT	1
31	0	2	4,255000129	4,255000129	4,255000129	sp|P63268|ACTH_MOUSE	Actin, gamma-enteric smooth muscle OS=Mus musculus	MOUSE	1
31	0	2	4,255000129	4,255000129	4,255000129	sp|P63267|ACTH_HUMAN	Actin, gamma-enteric smooth muscle OS=Homo sapiens	HUMAN	1
31	0	2	4,244000092	4,244000092	4,244000092	sp|P62740|ACTA_RABIT	Actin, aortic smooth muscle OS=Oryctolagus cuniculus G	RABIT	1
31	0	2	4,244000092	4,244000092	4,244000092	sp|P62739|ACTA_BOVIN	Actin, aortic smooth muscle OS=Bos taurus GN=ACTA2 F	BOVIN	1
31	0	2	4,244000092	4,244000092	4,244000092	sp|P62738|ACTA_RAT	Actin, aortic smooth muscle OS=Rattus norvegicus GN=A	RAT	1
31	0	2	4,244000092	4,244000092	4,244000092	sp|P62737|ACTA_MOUSE	Actin, aortic smooth muscle OS=Mus musculus GN=Acta	MOUSE	1
31	0	2	4,244000092	4,244000092	4,244000092	sp|P62736|ACTA_HUMAN	Actin, aortic smooth muscle OS=Homo sapiens GN=ACTA	HUMAN	1
31	0	2	4,267000034	4,267000034	4,267000034	sp|P60011|ACT_SACBA	Actin OS=Saccharomyces bayanus GN=ACT1 PE=3 SV=1	SACBA	1
31	0	2	4,267000034	4,267000034	4,267000034	sp|P60010|ACT_YEAST	Actin OS=Saccharomyces cerevisiae (strain ATCC 204508	YEAST	1
31	0	2	4,267000034	4,267000034	4,267000034	sp|P60009|ACT_CANGA	Actin OS=Candida glabrata (strain ATCC 2001 / CBS 138 /	CANGA	1
31	0	2	4,774999991	4,774999991	4,774999991	sp|P53504|ACT1_SORBI	Actin-1 OS=Sorghum bicolor GN=AC1 PE=2 SV=1	SORBI	1
31	0	2	4,774999991	4,774999991	4,774999991	sp|P53498|ACT_CHLRE	Actin OS=Chlamydomonas reinhardtii PE=2 SV=1	CHLRE	1
31	0	2	4,774999991	4,774999991	4,774999991	sp|P53497|ACT12_ARATH	Actin-12 OS=Arabidopsis thaliana GN=ACT12 PE=1 SV=1	ARATH	1
31	0	2	4,774999991	4,774999991	4,774999991	sp|P53496|ACT11_ARATH	Actin-11 OS=Arabidopsis thaliana GN=ACT11 PE=1 SV=1	ARATH	1
31	0	2	4,774999991	4,774999991	4,774999991	sp|P53494|ACT4_ARATH	Actin-4 OS=Arabidopsis thaliana GN=ACT4 PE=1 SV=1	ARATH	1
31	0	2	4,774999991	4,774999991	4,774999991	sp|P53492|ACT7_ARATH	Actin-7 OS=Arabidopsis thaliana GN=ACT7 PE=1 SV=1	ARATH	1
31	0	2	4,244000092	4,244000092	4,244000092	sp|P53482|ACTSB_TAKRU	Actin, alpha skeletal muscle B OS=Takifugu rubripes GN	TAKRU	1
31	0	2	4,244000092	4,244000092	4,244000092	sp|P53480|ACTC_TAKRU	Actin, alpha cardiac OS=Takifugu rubripes PE=2 SV=1	TAKRU	1
31	0	2	4,244000092	4,244000092	4,244000092	sp|P53479|ACTS_CYPCA	Actin, alpha skeletal muscle OS=Cyprinus carpio GN=act	CYPCA	1
31	0	2	4,786999896	4,786999896	4,786999896	sp|P53476|ACT_TOXGO	Actin OS=Toxoplasma gondii GN=ACT1 PE=3 SV=1	TOXGO	1
31	0	2	4,233000055	4,233000055	4,233000055	sp|P53475|ACTN_STYCL	Actin, muscle OS=Styela clava GN=TB12 PE=2 SV=1	STYCL	1
31	0	2	4,233000055	4,233000055	4,233000055	sp|P53467|ACTM_MOLOC	Actin, larval muscle-type OS=Molgula oculata PE=3 SV=1	MOLOC	1
31	0	2	4,786999896	4,786999896	4,786999896	sp|P53461|ACTC_HALRO	Actin, nonmuscle OS=Halocynthia roretzi GN=CA1 PE=3	HALRO	1
31	0	2	4,233000055	4,233000055	4,233000055	sp|P53460|ACT1_HALRO	Actin, muscle 1A OS=Halocynthia roretzi GN=MA1A PE=	HALRO	1
31	0	2	4,82599996	4,82599996	4,82599996	sp|P53459|ACT6_DIPDE	Actin-6 (Fragment) OS=Diphyllobothrium dendriticum G	DIPDE	1
31	0	2	4,774999991	4,774999991	4,774999991	sp|P53457|ACT3_DIPDE	Actin-3 OS=Diphyllobothrium dendriticum GN=ACT3 PE=	DIPDE	1
31	0	2	4,244000092	4,244000092	4,244000092	sp|P49055|ACTS_CARAU	Actin, alpha skeletal muscle OS=Carassius auratus GN=a	CARAU	1
31	0	2	4,774999991	4,774999991	4,774999991	sp|P46258|ACT3_PEA	Actin-3 OS=Pisum sativum PE=2 SV=1	PEA	1
31	0	2	4,969000071	4,969000071	4,969000071	sp|P45521|ACT_PROCL	Actin (Fragment) OS=Procambarus clarkii PE=1 SV=1	PROCL	1
31	0	2	4,267000034	4,267000034	4,267000034	sp|P43239|ACT1_PNECA	Actin-1 OS=Pneumocystis carinii PE=2 SV=1	PNECA	1
31	0	2	4,800000042	4,800000042	4,800000042	sp|P35432|ACT1_ECHGR	Actin-1 OS=Echinococcus granulosus GN=ACTI PE=3 SV=	ECHGR	1
31	0	2	4,774999991	4,774999991	4,774999991	sp|P30171|ACT11_SOLTU	Actin-97 OS=Solanum tuberosum GN=AC97 PE=3 SV=1	SOLTU	1
31	0	2	9,230999649	9,230999649	9,230999649	sp|P30170|ACT10_SOLTU	Actin-85C (Fragment) OS=Solanum tuberosum GN=AC85	SOLTU	1
31	0	2	4,774999991	4,774999991	4,774999991	sp|P30169|ACT7_SOLTU	Actin-75 OS=Solanum tuberosum GN=AC75 PE=3 SV=1	SOLTU	1
31	0	2	4,774999991	4,774999991	4,774999991	sp|P30168|ACT6_SOLTU	Actin-71 OS=Solanum tuberosum GN=AC71 PE=3 SV=2	SOLTU	1
31	0	2	4,774999991	4,774999991	4,774999991	sp|P30167|ACT3_SOLTU	Actin-58 OS=Solanum tuberosum GN=AC58 PE=3 SV=1	SOLTU	1
31	0	2	4,786999896	4,786999896	4,786999896	sp|P30165|ACT2_PEA	Actin-2 OS=Pisum sativum PE=2 SV=1	PEA	1
31	0	2	4,786999896	4,786999896	4,786999896	sp|P30164|ACT1_PEA	Actin-1 OS=Pisum sativum PE=2 SV=1	PEA	1
31	0	2	4,786999896	4,786999896	4,786999896	sp|P30163|ACT2_ONCVO	Actin-2 OS=Onchocerca volvulus GN=act-2b PE=3 SV=1	ONCVO	1
31	0	2	4,786999896	4,786999896	4,786999896	sp|P30162|ACT1_ONCVO	Actin-1 OS=Onchocerca volvulus GN=act-1a PE=3 SV=1	ONCVO	1
31	0	2	5,437999964	5,437999964	5,437999964	sp|P30161|ACT_COSCS	Actin (Fragment) OS=Costaria costata PE=3 SV=2	COSCS	1
31	0	2	4,267000034	4,267000034	4,267000034	sp|P29751|ACTB_RABIT	Actin, cytoplasmic 1 OS=Oryctolagus cuniculus GN=ACTE	RABIT	1
31	0	2	4,851999879	4,851999879	4,851999879	sp|P27132|ACT2_NAEFO	Actin-2 (Fragment) OS=Naegleria fowleri PE=2 SV=1	NAEFO	1
31	0	2	4,800000042	4,800000042	4,800000042	sp|P27131|ACT1_NAEFO	Actin-1 OS=Naegleria fowleri PE=2 SV=2	NAEFO	1
31	0	2	4,233000055	4,233000055	4,233000055	sp|P27130|ACT2_HALRO	Actin, muscle 2/4/4A OS=Halocynthia roretzi GN=MA2 F	HALRO	1
31	0	2	4,233000055	4,233000055	4,233000055	sp|P26198|ACTM_STYCL	Actin, muscle OS=Styela clava PE=2 SV=1	STYCL	1
31	0	2	4,774999991	4,774999991	4,774999991	sp|P26197|ACT2_ABSGL	Actin-2 OS=Absidia glauca GN=ACT2 PE=3 SV=1	ABSGL	1
31	0	2	4,786999896	4,786999896	4,786999896	sp|P26182|ACT_ACHBI	Actin OS=Achlya bisexualis PE=3 SV=1	ACHBI	1
31	0	2	4,904999956	4,904999956	4,904999956	sp|P24263|ACTD_PHYPO	Actin, spherule isoform OS=Physarum polycephalum GN	PHYPO	1
31	0	2	4,724000022	4,724000022	4,724000022	sp|P23344|ACT2_DAUCA	Actin-2 OS=Daucus carota PE=2 SV=1	DAUCA	1
31	0	2	4,800000042	4,800000042	4,800000042	sp|P22132|ACT2_PHYIN	Actin-2 OS=Phytophthora infestans GN=ACTB PE=2 SV=1	PHYIN	1
31	0	2	4,786999896	4,786999896	4,786999896	sp|P22131|ACT1_PHYIN	Actin-1 OS=Phytophthora infestans GN=ACTA PE=2 SV=1	PHYIN	1
31	0	2	4,774999991	4,774999991	4,774999991	sp|P20904|ACT_VOLCA	Actin OS=Volvox carteri PE=3 SV=1	VOLCA	1
31	0	2	4,244000092	4,244000092	4,244000092	sp|P20399|ACT2_XENTR	Actin, alpha cardiac muscle 2 OS=Xenopus tropicalis PE=	XENTR	1
31	0	2	5,505000055	5,505000055	5,505000055	sp|P18602|ACT3_ARTSX	Actin, clone 302 (Fragment) OS=Artemia sp. PE=2 SV=1	ARTSX	1
31	0	2	4,255000129	4,255000129	4,255000129	sp|P18601|ACT2_ARTSX	Actin, clone 211 OS=Artemia sp. PE=2 SV=1	ARTSX	1
31	0	2	4,255000129	4,255000129	4,255000129	sp|P18600|ACT1_ARTSX	Actin, clone 205 OS=Artemia sp. PE=2 SV=1	ARTSX	1
31	0	2	4,255000129	4,255000129	4,255000129	sp|P17304|ACTM_APLCA	Actin, muscle OS=Aplysia californica PE=2 SV=1	APLCA	1
31	0	2	4,267000034	4,267000034	4,267000034	sp|P17128|ACT_KLULA	Actin OS=Kluyveromyces lactis (strain ATCC 8585 / CBS 2	KLULA	1
31	0	2	4,255000129	4,255000129	4,255000129	sp|P14235|ACT_CANAX	Actin OS=Candida albicans GN=ACT1 PE=3 SV=1	CANAX	1
31	0	2	4,800000042	4,800000042	4,800000042	sp|P13363|ACT_PHYME	Actin OS=Phytophthora megasperma PE=3 SV=1	PHYME	1
31	0	2	4,244000092	4,244000092	4,244000092	sp|P10995|ACT2_XENLA	Actin, alpha skeletal muscle 2 OS=Xenopus laevis GN=ac	XENLA	1
31	0	2	4,267000034	4,267000034	4,267000034	sp|P10989|ACT_SCHPO	Actin OS=Schizosaccharomyces pombe (strain 972 / ATC	SCHPO	1
31	0	2	4,786999896	4,786999896	4,786999896	sp|P10986|ACT4_CAEEL	Actin-4 OS=Caenorhabditis elegans GN=act-4 PE=3 SV=2	CAEEL	1
31	0	2	4,786999896	4,786999896	4,786999896	sp|P10984|ACT2_CAEEL	Actin-2 OS=Caenorhabditis elegans GN=act-2 PE=3 SV=3	CAEEL	1
31	0	2	12,86000013	12,86000013	12,86000013	sp|P10982|ACT1_ABSGL	Actin-1 (Fragment) OS=Absidia glauca GN=ACT1 PE=3 S\	ABSGL	1
31	0	2	4,786999896	4,786999896	4,786999896	sp|P0DM42|ACT3_CAEEL	Actin-3 OS=Caenorhabditis elegans GN=act-3 PE=1 SV=1	CAEEL	1
31	0	2	4,786999896	4,786999896	4,786999896	sp|P0DM41|ACT1_CAEEL	Actin-1 OS=Caenorhabditis elegans GN=act-1 PE=1 SV=1	CAEEL	1
31	0	2	4,244000092	4,244000092	4,244000092	sp|P08023|ACTA_CHICK	Actin, aortic smooth muscle OS=Gallus gallus GN=ACTA2	CHICK	1

31	0	2	4,255000129	4,255000129	4,255000129	sp|P07837|ACT2_BOMMO	Actin, muscle-type A2 OS=Bombyx mori PE=3 SV=1	BOMMO	1
31	0	2	4,244000092	4,244000092	4,244000092	sp|P04752|ACT3_XENLA	Actin, alpha skeletal muscle 3 OS=Xenopus laevis GN=ac	XENLA	1
31	0	2	4,244000092	4,244000092	4,244000092	sp|P04751|ACTC_XENLA	Actin, alpha cardiac muscle 1 OS=Xenopus laevis GN=act	XENLA	1
31	0	2	4,774999991	4,774999991	4,774999991	sp|P02581|ACT1_SOYBN	Actin-1 OS=Glycine max GN=SAC1 PE=3 SV=2	SOYBN	1
31	0	2	3,302999958	3,302999958	3,302999958	sp|P00544|FGR_FSVGR	Tyrosine-protein kinase transforming protein Fgr OS=Fel	FSVGR	1
31	0	2	4,774999991	4,774999991	4,774999991	sp|O81221|ACT_GOSHI	Actin OS=Gossypium hirsutum PE=3 SV=1	GOSHI	1
31	0	2	4,255000129	4,255000129	4,255000129	sp|O74258|ACT_OGAPD	Actin OS=Ogataea parapolymorpha (strain DL-1 / ATCC 2	OGAPD	1
31	0	2	4,774999991	4,774999991	4,774999991	sp|O65315|ACT_COLSC	Actin OS=Coleochaete scutata PE=2 SV=1	COLSC	1
31	0	2	4,761999846	4,761999846	4,761999846	sp|O65314|ACT_SCHDU	Actin OS=Scherffelia dubia PE=2 SV=1	SCHDU	1
31	0	2	4,267000034	4,267000034	4,267000034	sp|O17503|ACTC_BRALA	Actin, cytoplasmic OS=Branchiostoma lanceolatum PE=2	BRALA	1
31	0	2	4,210999981	4,210999981	4,210999981	sp|O17502|ACTM_BRALA	Actin, muscle OS=Branchiostoma lanceolatum PE=2 SV=	BRALA	1
31	0	2	4,233000055	4,233000055	4,233000055	sp|O15998|ACTM_CIOSA	Actin, muscle OS=Ciona savignyi PE=2 SV=1	CIOSA	1
31	0	2	4,444000125	4,444000125	4,444000125	sp|A5DQP9|ACT_PICGU	Actin OS=Meyerozyma guilliermondii (strain ATCC 6260	PICGU	1
31	0	2	4,774999991	4,774999991	4,774999991	sp|A2XLF2|ACT1_ORYSI	Actin-1 OS=Oryza sativa subsp. indica GN=ACT1 PE=2 SV	ORYSI	1
35	2,02	2,02	7,767000049	3,155000135	3,155000135	sp|P07339|CATD_HUMAN	Cathepsin D OS=Homo sapiens GN=CTSD PE=1 SV=1	HUMAN	1
36	2,02	2,02	6,831999868	3,415999934	3,415999934	sp|P05089|ARGI1_HUMAN	Arginase-1 OS=Homo sapiens GN=ARG1 PE=1 SV=2	HUMAN	1
36	0	2	5,882000178	3,406000137	3,406000137	sp|Q61176|ARGI1_MOUSE	Arginase-1 OS=Mus musculus GN=Arg1 PE=1 SV=1	MOUSE	1
36	0	2	3,415999934	3,415999934	3,415999934	sp|Q95JC8|ARGI1_PIG	Arginase-1 OS=Sus scrofa GN=ARG1 PE=2 SV=1	PIG	1
36	0	2	3,415999934	3,415999934	3,415999934	sp|Q2KJ64|ARGI1_BOVIN	Arginase-1 OS=Bos taurus GN=ARG1 PE=2 SV=1	BOVIN	1
36	0	2	3,406000137	3,406000137	3,406000137	sp|P07824|ARGI1_RAT	Arginase-1 OS=Rattus norvegicus GN=Arg1 PE=1 SV=2	RAT	1
38	2,01	2,01	0,590999983	0,590999983	0,39400002	sp|P20930|FILA_HUMAN	Filaggrin OS=Homo sapiens GN=FLG PE=1 SV=3	HUMAN	1
41	2	2	8,41299966	4,086999968	4,086999968	sp|P34955|A1AT_BOVIN	Alpha-1-antiproteinase OS=Bos taurus GN=SERPINA1 PE	BOVIN	1
42	2	2	4,746000096	2,373000048	2,373000048	sp|Q6UWP8|SBSN_HUMAN	Suprabasin OS=Homo sapiens GN=SBSN PE=1 SV=2	HUMAN	1
43	2	2	13,51000071	8,107999712	8,107999712	sp|Q659U5|LYSC_HALGR	Lysozyme C OS=Halichoerus grypus GN=LYZ PE=2 SV=1	HALGR	1
43	0	2	13,51000071	8,107999712	8,107999712	sp|Q659U1|LYSC_PHOVI	Lysozyme C OS=Phoca vitulina GN=LYZ PE=2 SV=1	PHOVI	1
43	0	2	13,51000071	8,107999712	8,107999712	sp|Q659U0|LYSC_LEPWE	Lysozyme C OS=Leptonychotes weddellii GN=LYZ PE=2 S	LEPWE	1
43	0	2	13,51000071	8,107999712	8,107999712	sp|P79847|LYSC_PYGNE	Lysozyme C OS=Pygathrix nemaeus GN=LYZ PE=3 SV=1	PYGNE	1
43	0	2	13,51000071	8,107999712	8,107999712	sp|P79811|LYSC_NASLA	Lysozyme C OS=Nasalis larvatus GN=LYZ PE=3 SV=1	NASLA	1
43	0	2	13,51000071	8,107999712	8,107999712	sp|P67980|LYSC_TRAFR	Lysozyme C OS=Trachypithecus francoisi GN=LYZ PE=2 S	TRAFR	1
43	0	2	13,51000071	8,107999712	8,107999712	sp|P67979|LYSC_TRAOB	Lysozyme C OS=Trachypithecus obscurus GN=LYZ PE=2 S	TRAOB	1
43	0	2	13,51000071	8,107999712	8,107999712	sp|P67978|LYSC_TRAVT	Lysozyme C OS=Trachypithecus vetulus GN=LYZ PE=3 SV	TRAVT	1
43	0	2	13,51000071	8,107999712	8,107999712	sp|P67977|LYSC_SEMEN	Lysozyme C OS=Semnopithecus entellus GN=LYZ PE=1 S	SEMEN	1
43	0	2	13,51000071	8,107999712	8,107999712	sp|P61632|LYSC_COLGU	Lysozyme C OS=Colobus guereza GN=LYZ PE=2 SV=1	COLGU	1
43	0	2	13,51000071	8,107999712	8,107999712	sp|P61631|LYSC_COLAN	Lysozyme C OS=Colobus angolensis GN=LYZ PE=3 SV=1	COLAN	1
43	0	2	13,51000071	8,107999712	8,107999712	sp|P61628|LYSC_PANTR	Lysozyme C OS=Pan troglodytes GN=LYZ PE=2 SV=1	PANTR	1
43	0	2	13,51000071	8,107999712	8,107999712	sp|P61627|LYSC_PANPA	Lysozyme C OS=Pan paniscus GN=LYZ PE=3 SV=1	PANPA	1
43	0	2	13,51000071	8,107999712	8,107999712	sp|P61626|LYSC_HUMAN	Lysozyme C OS=Homo sapiens GN=LYZ PE=1 SV=1	HUMAN	1
43	0	2	8,392000198	8,392000198	8,392000198	sp|Q9PU28|LYSC_SCOMX	Lysozyme C OS=Scophthalmus maximus GN=lys PE=2 SV	SCOMX	1
43	0	2	8,392000198	8,392000198	8,392000198	sp|Q9DD65|LYSC_PAROL	Lysozyme C OS=Paralichthys olivaceus PE=2 SV=1	PAROL	1
43	0	2	8,107999712	8,107999712	8,107999712	sp|Q6B411|LYSM_BOVIN	Lysozyme C, milk isozyme OS=Bos taurus PE=2 SV=1	BOVIN	1
43	0	2	8,107999712	8,107999712	8,107999712	sp|Q05820|LYSC2_RAT	Putative lysozyme C-2 OS=Rattus norvegicus GN=Lyz2 PI	RAT	1
43	0	2	9,230999649	9,230999649	9,230999649	sp|P85345|LYSC_AMYCA	Lysozyme C OS=Amyda cartilaginea GN=LYZ PE=1 SV=1	AMYCA	1
43	0	2	8,218999952	8,218999952	8,218999952	sp|P85045|LYS_BUFGA	Lysozyme C (Fragment) OS=Bufo gargarizans andrewsi P	BUFGA	1
43	0	2	9,230999649	9,230999649	9,230999649	sp|P81709|LYSC2_CANLF	Lysozyme C, spleen isozyme OS=Canis lupus familiaris PI	CANLF	1
43	0	2	8,107999712	8,107999712	8,107999712	sp|P79239|LYSC_PONPY	Lysozyme C OS=Pongo pygmaeus GN=LYZ PE=2 SV=1	PONPY	1
43	0	2	8,107999712	8,107999712	8,107999712	sp|P79180|LYSC_HYLLA	Lysozyme C OS=Hylobates lar GN=LYZ PE=2 SV=1	HYLLA	1
43	0	2	8,107999712	8,107999712	8,107999712	sp|P79179|LYSC_GORGO	Lysozyme C OS=Gorilla gorilla gorilla GN=LYZ PE=2 SV=1	GORGO	1
43	0	2	8,392000198	8,392000198	8,392000198	sp|P61944|LYSC_TAKRU	Lysozyme C OS=Takifugu rubripes PE=2 SV=1	TAKRU	1
43	0	2	9,230999649	9,230999649	9,230999649	sp|P37712|LYSC_CAMDR	Lysozyme C OS=Camelus dromedarius GN=LYZ PE=1 SV=	CAMDR	1
43	0	2	8,107999712	8,107999712	8,107999712	sp|P17897|LYZ1_MOUSE	Lysozyme C-1 OS=Mus musculus GN=Lyz1 PE=1 SV=1	MOUSE	1
43	0	2	9,230999649	9,230999649	9,230999649	sp|P16973|LYSC_RABIT	Lysozyme C OS=Oryctolagus cuniculus GN=LYZ PE=1 SV=	RABIT	1
43	0	2	8,107999712	8,107999712	8,107999712	sp|P12069|LYSC3_PIG	Lysozyme C-3 OS=Sus scrofa PE=1 SV=2	PIG	1
43	0	2	8,218999952	8,218999952	8,218999952	sp|P12068|LYSC2_PIG	Lysozyme C-2 OS=Sus scrofa PE=1 SV=2	PIG	1
43	0	2	9,375	9,375	9,375	sp|P12067|LYSC1_PIG	Lysozyme C-1 OS=Sus scrofa PE=1 SV=1	PIG	1
43	0	2	8,332999796	8,332999796	8,332999796	sp|P11941|LYSC2_ONCMY	Lysozyme C II OS=Oncorhynchus mykiss PE=1 SV=2	ONCMY	1
43	0	2	8,107999712	8,107999712	8,107999712	sp|P08905|LYZ2_MOUSE	Lysozyme C-2 OS=Mus musculus GN=Lyz2 PE=1 SV=2	MOUSE	1
43	0	2	8,107999712	8,107999712	8,107999712	sp|P00697|LYSC1_RAT	Lysozyme C-1 OS=Rattus norvegicus GN=Lyz1 PE=1 SV=2	RAT	1
44	2	2	3,550000116	1,957999915	1,957999915	sp|P22735|TGM1_HUMAN	Protein-glutamine gamma-glutamyltransferase K OS=Ho	HUMAN	1
44	0	2	1,913999952	1,913999952	1,913999952	sp|P22758|TGM1_RABIT	Protein-glutamine gamma-glutamyltransferase K OS=Or	RABIT	1
45	2	2	3,325999901	3,325999901	3,325999901	sp|Q9ZRR5|TBA3_HORVU	Tubulin alpha-3 chain OS=Hordeum vulgare GN=TUBA3	HORVU	1
45	0	2	3,325999901	3,325999901	3,325999901	sp|Q9ZRB7|TBA_WHEAT	Tubulin alpha chain OS=Triticum aestivum GN=TUBA PE	WHEAT	1
45	0	2	3,341000155	3,341000155	3,341000155	sp|Q9BQE3|TBA1C_HUMAN	Tubulin alpha-1C chain OS=Homo sapiens GN=TUBA1C F	HUMAN	1
45	0	2	3,325999901	3,325999901	3,325999901	sp|Q96460|TBA2_HORVU	Tubulin alpha-2 chain OS=Hordeum vulgare GN=TUBA2	HORVU	1
45	0	2	3,325999901	3,325999901	3,325999901	sp|Q71U36|TBA1A_HUMAN	Tubulin alpha-1A chain OS=Homo sapiens GN=TUBA1A F	HUMAN	1
45	0	2	3,333000094	3,333000094	3,333000094	sp|Q6VAG0|TBA2_GOSHI	Tubulin alpha-2 chain OS=Gossypium hirsutum PE=2 SV=	GOSHI	1
45	0	2	3,333000094	3,333000094	3,333000094	sp|Q6VAF9|TBA4_GOSHI	Tubulin alpha-4 chain OS=Gossypium hirsutum PE=2 SV=	GOSHI	1
45	0	2	3,325999901	3,325999901	3,325999901	sp|Q6P9V9|TBA1B_RAT	Tubulin alpha-1B chain OS=Rattus norvegicus GN=Tuba1	RAT	1
45	0	2	3,341000155	3,341000155	3,341000155	sp|Q6AYZ1|TBA1C_RAT	Tubulin alpha-1C chain OS=Rattus norvegicus GN=Tuba1	RAT	1
45	0	2	3,325999901	3,325999901	3,325999901	sp|Q5R1W4|TBA1B_PANTR	Tubulin alpha-1B chain OS=Pan troglodytes GN=TUBA1B	PANTR	1
45	0	2	3,333000094	3,333000094	3,333000094	sp|Q56WH1|TBA3_ARATH	Tubulin alpha-3 chain OS=Arabidopsis thaliana GN=TUB/	ARATH	1
45	0	2	3,325999901	3,325999901	3,325999901	sp|Q53M52|TBA2_ORYSJ	Tubulin alpha-2 chain OS=Oryza sativa subsp. japonica G	ORYSJ	1
45	0	2	3,325999901	3,325999901	3,325999901	sp|Q4R538|TBA1B_MACFA	Tubulin alpha-1B chain OS=Macaca fascicularis GN=TUB	MACFA	1
45	0	2	3,341000155	3,341000155	3,341000155	sp|Q3ZCJ7|TBA1C_BOVIN	Tubulin alpha-1C chain OS=Bos taurus GN=TUBA1C PE=1	BOVIN	1
45	0	2	3,325999901	3,325999901	3,325999901	sp|Q2XVP4|TBA1B_PIG	Tubulin alpha-1B chain OS=Sus scrofa GN=TUBA1B PE=1	PIG	1
45	0	2	3,31900008	3,31900008	3,31900008	sp|Q2HJ86|TBA1D_BOVIN	Tubulin alpha-1D chain OS=Bos taurus GN=TUBA1D PE=1	BOVIN	1
45	0	2	3,341000155	3,341000155	3,341000155	sp|Q28IX8|TBA_XENTR	Tubulin alpha chain OS=Xenopus tropicalis GN=tuba PE=	XENTR	1
45	0	2	3,333000094	3,333000094	3,333000094	sp|Q0WV25|TBA4_ARATH	Tubulin alpha-4 chain OS=Arabidopsis thaliana GN=TUB/	ARATH	1
45	0	2	3,325999901	3,325999901	3,325999901	sp|P81947|TBA1B_BOVIN	Tubulin alpha-1B chain OS=Bos taurus PE=1 SV=2	BOVIN	1
45	0	2	3,341000155	3,341000155	3,341000155	sp|P68373|TBA1C_MOUSE	Tubulin alpha-1C chain OS=Mus musculus GN=Tuba1c P	MOUSE	1
45	0	2	3,325999901	3,325999901	3,325999901	sp|P68370|TBA1A_RAT	Tubulin alpha-1A chain OS=Rattus norvegicus GN=Tuba1	RAT	1
45	0	2	3,325999901	3,325999901	3,325999901	sp|P68369|TBA1A_MOUSE	Tubulin alpha-1A chain OS=Mus musculus GN=Tuba1a P	MOUSE	1
45	0	2	3,341000155	3,341000155	3,341000155	sp|P68365|TBA1C_CRIGR	Tubulin alpha-1C chain OS=Cricetulus griseus GN=TUBA1	CRIGR	1
45	0	2	3,325999901	3,325999901	3,325999901	sp|P68363|TBA1B_HUMAN	Tubulin alpha-1B chain OS=Homo sapiens GN=TUBA1B F	HUMAN	1
45	0	2	3,325999901	3,325999901	3,325999901	sp|P68362|TBA1A_CRIGR	Tubulin alpha-1A chain OS=Cricetulus griseus GN=TUBA1	CRIGR	1
45	0	2	3,325999901	3,325999901	3,325999901	sp|P68361|TBA1B_CRIGR	Tubulin alpha-1B chain OS=Cricetulus griseus GN=TUBA1	CRIGR	1
45	0	2	3,325999901	3,325999901	3,325999901	sp|P68360|TBA1B_MERUN	Tubulin alpha-1B chain OS=Meriones unguiculatus GN=T	MERUN	1
45	0	2	3,31900008	3,31900008	3,31900008	sp|P46259|TBA1_PEA	Tubulin alpha-1 chain OS=Pisum sativum GN=TUBA1 PE=	PEA	1
45	0	2	3,325999901	3,325999901	3,325999901	sp|P36220|TBA_TORMA	Tubulin alpha chain OS=Torpedo marmorata PE=2 SV=1	TORMA	1
45	0	2	3,333000094	3,333000094	3,333000094	sp|P33629|TBA_PRUDU	Tubulin alpha chain OS=Prunus dulcis GN=TUBA PE=2 SV	PRUDU	1
45	0	2	13,26999962	13,26999962	13,26999962	sp|P33628|TBA_PICAB	Tubulin alpha chain (Fragment) OS=Picea abies GN=TUB	PICAB	1
45	0	2	3,333000094	3,333000094	3,333000094	sp|P29511|TBA6_ARATH	Tubulin alpha-6 chain OS=Arabidopsis thaliana GN=TUB/	ARATH	1
45	0	2	3,325999901	3,325999901	3,325999901	sp|P14641|TBA2_MAIZE	Tubulin alpha-2 chain OS=Zea mays GN=TUBA2 PE=3 SV	MAIZE	1
45	0	2	3,325999901	3,325999901	3,325999901	sp|P14640|TBA1_MAIZE	Tubulin alpha-1 chain OS=Zea mays GN=TUBA1 PE=3 SV	MAIZE	1
45	0	2	3,341000155	3,341000155	3,341000155	sp|P08537|TBA_XENLA	Tubulin alpha chain OS=Xenopus laevis GN=tuba PE=2 S	XENLA	1
45	0	2	3,325999901	3,325999901	3,325999901	sp|P05213|TBA1B_MOUSE	Tubulin alpha-1B chain OS=Mus musculus GN=Tuba1b P	MOUSE	1
45	0	2	3,641000018	3,641000018	3,641000018	sp|P02552|TBA1_CHICK	Tubulin alpha-1 chain (Fragment) OS=Gallus gallus PE=1	CHICK	1
45	0	2	3,325999901	3,325999901	3,325999901	sp|P02550|TBA1A_PIG	Tubulin alpha-1A chain OS=Sus scrofa GN=TUBA1A PE=1	PIG	1
45	0	2	3,325999901	3,325999901	3,325999901	sp|O22347|TBA1_ELEIN	Tubulin alpha-1 chain OS=Eleusine indica GN=TUBA1 PE	ELEIN	1
45	0	2	3,333000094	3,333000094	3,333000094	sp|B9DHQ0|TBA5_ARATH	Tubulin alpha-5 chain OS=Arabidopsis thaliana GN=TUB/	ARATH	1
45	0	2	3,333000094	3,333000094	3,333000094	sp|B9DGT7|TBA2_ARATH	Tubulin alpha-2 chain OS=Arabidopsis thaliana GN=TUB/	ARATH	1
45	0	2	3,325999901	3,325999901	3,325999901	sp|A5A6J1|TBA1A_PANTR	Tubulin alpha-1A chain OS=Pan troglodytes GN=TUBA1A	PANTR	1
46	2	2	5,669999868	5,669999868	5,669999868	sp|Q9V3P0|PRDX1_DROME	Peroxiredoxin 1 OS=Drosophila melanogaster GN=Jafrac	DROME	1
46	0	2	5,640999973	5,640999973	5,640999973	sp|Q9NL98|PRDX_ASCSU	Peroxiredoxin OS=Ascaris suum PE=2 SV=1	ASCSU	1

46	0	2	5,556000024	5,556000024	5,556000024	sp|Q8K3U7|PRDX2_CRIGR	Peroxiredoxin-2 OS=Cricetulus griseus GN=PRDX2 PE=2	CRIGR	1
46	0	2	5,527999997	5,527999997	5,527999997	sp|Q6B4U9|PRDX1_MYOLU	Peroxiredoxin-1 OS=Myotis lucifugus GN=PRDX1 PE=1 S	MYOLU	1
46	0	2	5,556000024	5,556000024	5,556000024	sp|Q61171|PRDX2_MOUSE	Peroxiredoxin-2 OS=Mus musculus GN=Prdx2 PE=1 SV=	MOUSE	1
46	0	2	6,215000153	6,215000153	6,215000153	sp|Q5RC63|PRDX2_PONAB	Peroxiredoxin-2 OS=Pongo abelii GN=PRDX2 PE=2 SV=3	PONAB	1
46	0	2	5,556000024	5,556000024	5,556000024	sp|Q2PFZ3|PRDX2_MACFA	Peroxiredoxin-2 OS=Macaca fascicularis GN=PRDX2 PE=	MACFA	1
46	0	2	5,527999997	5,527999997	5,527999997	sp|Q06830|PRDX1_HUMAN	Peroxiredoxin-1 OS=Homo sapiens GN=PRDX1 PE=1 SV=	HUMAN	1
46	0	2	8,660999686	8,660999686	8,660999686	sp|P52552|PRDX2_PIG	Peroxiredoxin-2 (Fragment) OS=Sus scrofa GN=PRDX2 P	PIG	1
46	0	2	5,556000024	5,556000024	5,556000024	sp|P35704|PRDX2_RAT	Peroxiredoxin-2 OS=Rattus norvegicus GN=Prdx2 PE=1 S	RAT	1
46	0	2	5,556000024	5,556000024	5,556000024	sp|P32119|PRDX2_HUMAN	Peroxiredoxin-2 OS=Homo sapiens GN=PRDX2 PE=1 SV=	HUMAN	1
47	2	2	1,683999971	1,683999971	1,683999971	sp|Q86YZ3|HORN_HUMAN	Hornerin OS=Homo sapiens GN=HRNR PE=1 SV=2	HUMAN	1
48	2	2	3,403000161	3,403000161	3,403000161	sp|Q7YR44|CDSN_PANTR	Corneodesmosin OS=Pan troglodytes GN=CDSN PE=2 S\	PANTR	1
48	0	2	3,370999917	3,370999917	3,370999917	sp|Q5TM45|CDSN_MACMU	Corneodesmosin OS=Macaca mulatta GN=CDSN PE=3 S\	MACMU	1
48	0	2	3,403000161	3,403000161	3,403000161	sp|Q15517|CDSN_HUMAN	Corneodesmosin OS=Homo sapiens GN=CDSN PE=1 SV=	HUMAN	1
49	2	2	3,162999824	3,162999824	3,162999824	sp|Q5FMC0|GLYA_LACAC	Serine hydroxymethyltransferase OS=Lactobacillus acid	LACAC	1
50	2	2	7,805000246	7,805000246	7,805000246	sp|P04792|HSPB1_HUMAN	Heat shock protein beta-1 OS=Homo sapiens GN=HSPB1	HUMAN	1
51	2	2	9,544999897	9,544999897	9,544999897	sp|O75629|CREG1_HUMAN	Protein CREG1 OS=Homo sapiens GN=CREG1 PE=1 SV=1	HUMAN	1
52	1,77	1,77	6,7900002	6,7900002	6,7900002	sp|Q5VSP4|LC1L1_HUMAN	Putative lipocalin 1-like protein 1 OS=Homo sapiens GN=	HUMAN	1
52	0	1,77	6,25	6,25	6,25	sp|P31025|LCN1_HUMAN	Lipocalin-1 OS=Homo sapiens GN=LCN1 PE=1 SV=1	HUMAN	1
53	1,56	1,56	7,005000114	5,071999878	2,174000069	sp|Q8IW75|SPA12_HUMAN	Serpin A12 OS=Homo sapiens GN=SERPINA12 PE=1 SV=	HUMAN	1
54	1,54	1,54	4,191999882	4,191999882	4,191999882	sp|O60911|CATL2_HUMAN	Cathepsin L2 OS=Homo sapiens GN=CTSV PE=1 SV=2	HUMAN	1
55	1,49	1,49	24,26999956	24,26999956	24,26999956	sp|P55000|SLUR1_HUMAN	Secreted Ly-6/uPAR-related protein 1 OS=Homo sapiens	HUMAN	1
57	1,41	1,41	19,14999932	5,851000175	5,851000175	sp|O75223|GGCT_HUMAN	Gamma-glutamylcyclotransferase OS=Homo sapiens GN	HUMAN	1
58	1,38	1,38	10,75000018	7,009000331	7,009000331	sp|B9A064|IGLL5_HUMAN	Immunoglobulin lambda-like polypeptide 5 OS=Homo sa	HUMAN	1
58	0	1,38	14,14999962	14,14999962	14,14999962	sp|P0CG06|LAC3_HUMAN	Ig lambda-3 chain C regions OS=Homo sapiens GN=IGLC	HUMAN	1
58	0	1,38	14,14999962	14,14999962	14,14999962	sp|P0CG05|LAC2_HUMAN	Ig lambda-2 chain C regions OS=Homo sapiens GN=IGLC	HUMAN	1
58	0	1,38	14,14999962	14,14999962	14,14999962	sp|P0CG04|LAC1_HUMAN	Ig lambda-1 chain C regions OS=Homo sapiens GN=IGLC	HUMAN	1
58	0	1,38	14,14999962	14,14999962	14,14999962	sp|P0CF74|LAC6_HUMAN	Ig lambda-6 chain C region OS=Homo sapiens GN=IGLC6	HUMAN	1
58	0	1,38	14,14999962	14,14999962	14,14999962	sp|A0M8Q6|LAC7_HUMAN	Ig lambda-7 chain C region OS=Homo sapiens GN=IGLC7	HUMAN	1
59	1,23	1,23	3,282000124	3,282000124	1,554000005	sp|Q5T749|KPRP_HUMAN	Keratinocyte proline-rich protein OS=Homo sapiens GN=	HUMAN	1
60	1,08	1,08	0,598300016	0,598300016	0,239300006	RRRRRsp|Q19317|NBEA_CAEEL	REVERSED Putative neurobeachin homolog OS=Caenorh	CAEEL	1
61	0,85	0,85	1,917999983	1,917999983	1,917999983	RRRRRsp|Q9SFC7|FB135_ARATH	REVERSED F-box protein At3g07870 OS=Arabidopsis tha	ARATH	1
62	0,69	0,69	4,988000169	3,088000044	3,088000044	sp|Q9UI42|CBPA4_HUMAN	Carboxypeptidase A4 OS=Homo sapiens GN=CPA4 PE=1	HUMAN	1
62	0	0,69	5,000000075	3,095000051	3,095000051	sp|Q6P8K8|CBPA4_MOUSE	Carboxypeptidase A4 OS=Mus musculus GN=Cpa4 PE=2	MOUSE	1
63	0,64	0,64	13,33000064	4,69099991	1,975000091	sp|Q96P63|SPB12_HUMAN	Serpin B12 OS=Homo sapiens GN=SERPINB12 PE=1 SV=	HUMAN	1
64	0,63	0,63	0,870500039	0,870500039	0,870500039	sp|Q05884|AMY_STRLI	Alpha-amylase OS=Streptomyces lividans GN=amy PE=1	STRLI	1
65	0,55	0,55	100	100	100	sp|P84735|PS19_PINST	Putative LRR disease resistance protein/transmembrane	PINST	1
65	0	0,55	100	100	100	sp|P84732|PS16_PINST	Putative LRR disease resistance protein/transmembrane	PINST	1
65	0	0,55	100	100	100	sp|P84720|PS4_PINST	Putative LRR disease resistance protein/transmembrane	PINST	1
66	0,27	0,27	65,82000256	26,57999992	0	sp|P02814|SMR3B_HUMAN	Submaxillary gland androgen-regulated protein 3B OS=H	HUMAN	0
67	0,23	0,23	7,406999916	6,667000055	0	sp|Q05816|FABP5_MOUSE	Fatty acid-binding protein, epidermal OS=Mus musculus	MOUSE	0
67	0	0,23	7,406999916	6,667000055	0	sp|Q01469|FABP5_HUMAN	Fatty acid-binding protein, epidermal OS=Homo sapiens	HUMAN	0
67	0	0,23	7,406999916	6,667000055	0	sp|P55053|FABP5_RAT	Fatty acid-binding protein, epidermal OS=Rattus norvegi	RAT	0
68	0,21	0,26	0,836499967	0,836499967	0	sp|Q5D862|FILA2_HUMAN	Filaggrin-2 OS=Homo sapiens GN=FLG2 PE=1 SV=1	HUMAN	0
69	0,15	0,15	1,699000038	1,699000038	0	sp|Q9HH00|TRET_PYRFU	Trehalose synthase OS=Pyrococcus furiosus (strain ATCC	PYRFU	0
69	0	0,15	0,813999958	0,813999958	0	sp|Q7NAR7|SYV_MYCGA	Valine--tRNA ligase OS=Mycoplasma gallisepticum (strai	MYCGA	0
69	0	0,15	1,699000038	1,699000038	0	sp|Q7LYW5|TRET_THELN	Trehalose synthase OS=Thermococcus litoralis (strain AT	THELN	0
69	0	0,15	2,33299993	2,33299993	0	sp|Q5UNV7|YL698_MIMIV	Uncharacterized protein L698 OS=Acanthamoeba polypl	MIMIV	0
69	0	0,15	0,944699999	0,944699999	0	RRRRRsp|Q6KZR2|PURL_PICTO	REVERSED Phosphoribosylformylglycinamidine synthase	PICTO	0
69	0	0,15	2,692000009	2,692000009	0	RRRRRsp|Q6AX41|MC25A_XENLA	REVERSED MICOS complex subunit mic25-a OS=Xenopus	XENLA	0
69	0	0,15	2,766999975	2,766999975	0	RRRRRsp|Q5U509|MC25B_XENLA	REVERSED MICOS complex subunit mic25-b OS=Xenopus	XENLA	0
69	0	0,15	2,766999975	2,766999975	0	RRRRRsp|Q5FVV3|MIC25_XENTR	REVERSED MICOS complex subunit mic25 OS=Xenopus t	XENTR	0
69	0	0,15	0,773499999	0,773499999	0	RRRRRsp|O13955|VAM6_SCHPO	REVERSED Vacuolar morphogenesis protein 6 OS=Schizo	SCHPO	0
70	0,15	0,15	1,535	1,535	0	sp|O28606|SAT_ARCFU	Sulfate adenylyltransferase OS=Archaeoglobus fulgidus	ARCFU	0
71	0,1	0,1	45,28000057	13,2100001	0	sp|P01834|IGKC_HUMAN	Ig kappa chain C region OS=Homo sapiens GN=IGKC PE=	HUMAN	0
72	0,07	0,07	2,291999944	2,291999944	0	RRRRRsp|P31755|OCH1_YEAST	REVERSED Initiation-specific alpha-1,6-mannosyltransfe	YEAST	0
73	0,07	0,07	7,512000203	7,512000203	0	RRRRRsp|P13673|SKGR_XENLA	REVERSED Skin granule protein OS=Xenopus laevis GN=	XENLA	0
74	0,06	0,06	1,737000048	1,737000048	0	sp|A7M957|RPOC1_CUSRE	DNA-directed RNA polymerase subunit beta' OS=Cuscut	CUSRE	0
75	0,05	0,05	2,754000016	2,754000016	0	sp|Q67TB7|ATPB_SYMTH	ATP synthase subunit beta OS=Symbiobacterium therm	SYMTH	0
76	0,05	0,05	1,765999943	1,765999943	0	sp|P21910|LAML2_XENLA	Lamin-L(II) OS=Xenopus laevis PE=2 SV=1	XENLA	0
77	0,05	0,05	4,14500013	4,14500013	0	sp|P01012|OVAL_CHICK	Ovalbumin OS=Gallus gallus GN=SERPINB14 PE=1 SV=2	CHICK	0
78	0,05	0,05	2,524000034	2,524000034	0	RRRRRsp|Q10310|MUG68_SCHPO	REVERSED Meiotically up-regulated gene 68 protein OS=	SCHPO	0

Supplementary Data S2 | Peptide quantification according to a joint search performed as described in the Methods section

Row	Index	Peak Name	m/z	Ret. Time	Group	CSI_02	CSI_03	CSVII_01	CSI_01	CSI_04	CSVI_01	CSV_01	CSV_02	CSV_03
1	1	GPYESGSGHSSGLGHR	792,862	10,6	sp|Q86YZ3|HORN_HUMAN	1806,59897	2113,14565	2695,48013	2326,16195	1339,40981	1669,25312	2091,20831	553,932097	971,354705
2	2	KYEDEINKR	597,808	11,18	sp|P48668|K2C6C_HUMAN	15140,4977	13227,4831	1497,43565	280,103924	10380,5855	17767,6335	399,889136	1484,32559	4121,44175
3	3	KYEDEINKR	597,808	11,18	sp|Q7Z794|K2C1B_HUMAN	15140,4977	13227,4831	1497,43565	280,103924	10380,5855	17767,6335	399,889136	1484,32559	4121,44175
4	4	KYEDEINKR	597,808	11,18	sp|P02538|K2C6A_HUMAN	15140,4977	13227,4831	1497,43565	280,103924	10380,5855	17767,6335	399,889136	1484,32559	4121,44175
5	5	KYEDEINKR	597,808	11,18	sp|P13647|K2C5_HUMAN	15140,4977	13227,4831	1497,43565	280,103924	10380,5855	17767,6335	399,889136	1484,32559	4121,44175
6	6	LRSEIDNVKK	401,235	11,45	sp|P04264|K2C1_HUMAN	308,783749	81014,2591	65938,0397	34101,769	55263,4007	48764,3461	222,786472	129,062916	312,534794
7	7	FSSSSGYGGGSSR	618,268	12	sp|P35527|K1C9_HUMAN	9118,99572	19884,996	23478,3809	15894,3071	11076,238	16637,0865	194,98317	1984,03063	140,673244
8	8	VQDQDLPNTPHSK	739,866	12,61	sp|Q08554|DSC1_HUMAN	2396,59886	1394,32417	1686,20498	3838,7937	2480,90235	4290,29711	825,894615	383,923514	4254,07182
9	9	SKEEAEALYHSK	696,344	12,79	sp|P35908|K22E_HUMAN	36693,7011	25504,8327	2639,88923	931,369682	16689,0222	3265,43497	205,176338	161,37934	1324,35237
10	10	NKYEDEINKR	654,831	13,09	sp|P48668|K2C6C_HUMAN	50111,3703	82158,8256	6435,73931	579,647855	55130,5901	2795,17713	2185,22721	660,326581	1541,39492
11	11	NKYEDEINKR	654,831	13,09	sp|P02538|K2C6A_HUMAN	50111,3703	82158,8256	6435,73931	579,647855	55130,5901	2795,17713	2185,22721	660,326581	1541,39492
12	12	NKYEDEINKR	654,831	13,09	sp|P13647|K2C5_HUMAN	50111,3703	82158,8256	6435,73931	579,647855	55130,5901	2795,17713	2185,22721	660,326581	1541,39492
13	13	NKYEDEINKR	654,831	13,09	sp|P04264|K2C1_HUMAN	50111,3703	82158,8256	6435,73931	579,647855	55130,5901	2795,17713	2185,22721	660,326581	1541,39492
14	14	LQGEIAHVK	497,787	13,23	sp|P35908|K22E_HUMAN	36913,2955	27108,8673	1025,57406	262,555838	15615,4895	313,47833	284,563243	260,35855	522,840394
15	15	LLEGEEC[CAM]R	503,238	13,57	sp|P48668|K2C6C_HUMAN	10385,1853	12939,9576	11650,6205	7490,71475	5743,3509	8036,51915	216,048261	190,281571	263,469593
16	16	LLEGEEC[CAM]R	503,238	13,57	sp|Q8N1N4|K2C78_HUMAN	10385,1853	12939,9576	11650,6205	7490,71475	5743,3509	8036,51915	216,048261	190,281571	263,469593
17	17	LLEGEEC[CAM]R	503,238	13,57	sp|P02538|K2C6A_HUMAN	10385,1853	12939,9576	11650,6205	7490,71475	5743,3509	8036,51915	216,048261	190,281571	263,469593
18	18	LLEGEEC[CAM]R	503,238	13,57	sp|P13647|K2C5_HUMAN	10385,1853	12939,9576	11650,6205	7490,71475	5743,3509	8036,51915	216,048261	190,281571	263,469593
19	19	SKAEAESLYQSK	670,838	13,7	sp|P04264|K2C1_HUMAN	121144,581	155355,679	123377,669	67936,6146	112499,155	1030,12411	672,24795	1321,55357	1386,30099
20	20	YEELQQTAGR	597,791	14,17	sp|P13647|K2C5_HUMAN	10021,693	10312,2514	10099,0396	6827,02115	7024,38312	541,316427	222,766018	299,261711	854,270726
21	21	SQYEQLAEQNRK	747,371	14,21	sp|P13645|K1C10_HUMAN	205283,5	156023,225	138756,137	67818,4392	158300,007	224172,015	317,57083	250,740568	2620,21659
22	22	AQYEDIAQK	533,264	14,38	sp|P04264|K2C1_HUMAN	97758,6963	132847,255	95362,6969	51405,1248	81028,7436	81107,7708	153,428091	7703,8882	4764,38261
23	23	YDPEAASAPGSGNPC[CAM]HEASAAQK	772,339	14,51	sp|P81605|DCD_HUMAN	5219,77037	3869,09451	6635,00737	5664,79584	7790,44948	1093,34105	277,974153	622,314053	2511,81092
24	24	SEITELRR	502,279	14,54	sp|Q6IFW6|K1C10_RAT	228795,808	133698,814	154942,983	83566,3784	110065,08	190,973073	148,413202	281,146641	264,147465
25	25	SEITELRR	502,279	14,54	sp|P13645|K1C10_HUMAN	228795,808	133698,814	154942,983	83566,3784	110065,08	190,973073	148,413202	281,146641	264,147465
26	26	IKFEM[DTM]EQNLR	420,563	14,62	sp|P35527|K1C9_HUMAN	7065,84367	28132,7516	4208,7821	575,957996	15894,6421	219,183804	14741,1601	330,316805	156,586343
27	27	EVATNSELVQSGK	681,349	14,74	sp|Q04695|K1C17_HUMAN	11553,4852	20266,8657	14909,5548	8320,79639	9511,1323	1596,3177	1359,97552	287,458674	784,900933
28	28	EVATNSELVQSGK	681,349	14,74	sp|P02533|K1C14_HUMAN	11553,4852	20266,8657	14909,5548	8320,79639	9511,1323	1596,3177	1359,97552	287,458674	784,900933
29	29	GSC[CAM]GIGGGIGGGSSR	639,796	14,79	sp|P08779|K1C16_HUMAN	6578,25238	14453,7176	963,475909	144,704224	3436,95641	1731,88596	411,987913	131,037547	1123,24739
30	30	GSC[CAM]GIGGGIGGGSSR	639,796	14,79	sp|P02533|K1C14_HUMAN	6578,25238	14453,7176	963,475909	144,704224	3436,95641	1731,88596	411,987913	131,037547	1123,24739
31	31	GRLDSELR	473,259	14,82	sp|P48668|K2C6C_HUMAN	729,715624	731,279804	582,676947	146,984893	841,436001	1292,3036	13569,8405	138,407699	1128,74177
32	32	GRLDSELR	473,259	14,82	sp|P02538|K2C6A_HUMAN	729,715624	731,279804	582,676947	146,984893	841,436001	1292,3036	13569,8405	138,407699	1128,74177
33	33	GRLDSELR	473,259	14,82	sp|P13647|K2C5_HUMAN	729,715624	731,279804	582,676947	146,984893	841,436001	1292,3036	13569,8405	138,407699	1128,74177
34	34	AQYEEIANR	547,268	14,95	sp|P13647|K2C5_HUMAN	9819,15869	12643,0874	8433,09529	6971,16573	7165,96483	151,636962	240,777686	480,939772	1705,4547
35	35	VDNALQSGNSQESVTEQDSK	1068,49	15,06	sp|P01834|IGKC_HUMAN	131,685849	367,295024	507,401181	834,202448	2742,58914	256,943055	120,54331	123,721944	230,180499
36	36	NTKQEIAEINR	658,352	15,15	sp|P48668|K2C6C_HUMAN	1606,87748	5118,41389	3724,56962	1797,30537	2750,87653	676,302332	7995,9169	453,258227	611,770133
37	37	NTKQEIAEINR	658,352	15,15	sp|P02538|K2C6A_HUMAN	1606,87748	5118,41389	3724,56962	1797,30537	2750,87653	676,302332	7995,9169	453,258227	611,770133
38	38	SKDEAEALYQTK	691,844	15,57	sp|Q7Z794|K2C1B_HUMAN	5489,98512	1178,96942	958,697725	3319,09687	9561,59625	3126,54184	1842,40303	494,365365	1393,50994
39	39	GGSGGSHGGGSGFGGESGGSYGGGEEASGSGG	1075,1	15,59	sp|P35527|K1C9_HUMAN	3732,82425	9637,01111	348,258064	8235,03939	7416,611	8257,96084	151,859191	258,056763	513,500123
40	40	SSEDPNEDIVER	695,311	15,62	sp|P01591|IGJ_HUMAN	2011,45187	1582,11823	772,348342	498,995676	7107,76029	1628,6038	1029,1971	235,06152	907,176294
41	41	SLLEGEGSSGGGGR	631,801	15,62	sp|P13645|K1C10_HUMAN	59336,6569	97625,3743	59548,6525	41924,0706	48749,2982	2237,06106	902,96855	665,262665	5029,19297
42	42	AQYEEIAQR	554,276	15,62	sp|P48668|K2C6C_HUMAN	803,867173	507,604945	593,938004	492,050444	135,938031	36003,9714	24969,5514	140,496802	1567,57013
43	43	AQYEEIAQR	554,276	15,62	sp|P19013|K2C4_HUMAN	803,867173	507,604945	593,938004	492,050444	135,938031	36003,9714	24969,5514	140,496802	1567,57013
44	44	AQYEEIAQR	554,276	15,62	sp|P02538|K2C6A_HUMAN	803,867173	507,604945	593,938004	492,050444	135,938031	36003,9714	24969,5514	140,496802	1567,57013
45	45	AQYEEIAQR	554,276	15,62	sp|P35908|K22E_HUMAN	803,867173	507,604945	593,938004	492,050444	135,938031	36003,9714	24969,5514	140,496802	1567,57013
46	46	GFSSGSAVVSGGSR	627,806	15,7	sp|P35908|K22E_HUMAN	80519,3037	47411,5289	51826,3134	27340,041	43572,1117	281,64728	470,769312	179,917811	1516,67006
47	47	HGGGGGGFGGGGFGSR	660,795	15,71	sp|P35908|K22E_HUMAN	53595,2995	1225,59876	42170,4256	18804,2624	36656,9449	486,995252	3277,45583	532,774677	944,526448
48	48	AAR.ATMQNLNDR	545,768	15,72	sp|Q04695|K1C17_HUMAN	6817,13995	19750,6929	1323,23596	7041,9581	5466,02485	576,106987	170,864958	169,843104	1643,82261
49	49	VDPEIQNVK	521,282	15,88	sp|P35908|K22E_HUMAN	1442,83852	443,909882	145,922624	236,903695	227,153069	49298,3215	23919,4745	7793,64062	1365,54682
50	50	TKYEHELALR	630,339	15,97	sp|P08779|K1C16_HUMAN	1522,1957	17657,8792	2957,00872	196,708613	1392,48271	3262,06837	338,760171	164,723377	1546,62614
51	51	TLLEGEESR	517,262	15,97	sp|P04264|K2C1_HUMAN	193474,705	210613,915	217100,441	132047,865	161166,769	328,111534	267,786054	214,285175	13354,462
52	52	ISSVLAGGSC[CAM]R	553,784	16,2	sp|P08779|K1C16_HUMAN	12950,0866	27644,2531	12855,876	6946,16245	7235,85951	287,866428	924,370537	224,732424	766,012603
53	53	ISSVLAGGSC[CAM]R	553,784	16,2	sp|P02533|K1C14_HUMAN	12950,0866	27644,2531	12855,876	6946,16245	7235,85951	287,866428	924,370537	224,732424	766,012603
54	54	YENEVALR	497,254	16,44	sp|Q6IFW6|K1C10_RAT	36265,8466	24336,6114	24329,3583	12811,4495	19544,4335	19832,249	191,934235	150,121001	1714,84755
55	55	LASYLDK	405,223	16,47	sp|Q04695|K1C17_HUMAN	86327,8332	66896,226	68654,4532	36070,3236	51609,3425	36531,8035	827,363583	357,294425	1788,70821
56	56	LASYLDK	405,223	16,47	sp|P08779|K1C16_HUMAN	86327,8332	66896,226	68654,4532	36070,3236	51609,3425	36531,8035	827,363583	357,294425	1788,70821
57	57	LASYLDK	405,223	16,47	sp|P02533|K1C14_HUMAN	86327,8332	66896,226	68654,4532	36070,3236	51609,3425	36531,8035	827,363583	357,294425	1788,70821
58	58	AGEVQEPELR	564,29	16,51	sp|P25311|ZA2G_HUMAN	3953,5347	2187,86963	5912,70238	5350,53492	8907,98879	490,532903	168,280777	124,605224	2372,43972
59	59	NVKVDPEIQNVK	691,884	16,98	sp|P35908|K22E_HUMAN	10393,3617	20126,8822	18824,2962	9053,96308	12255,2854	1996,46701	2699,78399	480,177998	4650,77727
60	60	RQLDSIVGER	586,826	17,08	sp|P48668|K2C6C_HUMAN	8592,39771	8319,89751	15467,8419	7100,87372	7441,60357	2283,50064	2206,99461	1033,8711	4901,73557
61	61	RQLDSIVGER	586,826	17,08	sp|P02538|K2C6A_HUMAN	8592,39771	8319,89751	15467,8419	7100,87372	7441,60357	2283,50064	2206,99461	1033,8711	4901,73557
62	62	SRTEAESWYQTK	743,355	17,08	sp|P13647|K2C5_HUMAN	4243,93442	6953,34411	2430,66735	2271,90282	2333,54102	1589,97185	156,367013	340,428912	2359,20122
63	63	RQLDSIVGER	586,826	17,08	sp|P13647|K2C5_HUMAN	8592,39771	8319,89751	15467,8419	7100,87372	7441,60357	2283,50064	2206,99461	1033,8711	4901,73557
64	64	SGFSSVSVSR	506,757	17,1	sp|P02538|K2C6A_HUMAN	8394,32467	5479,1399	7311,57338	2356,94957	820,391868	326,292285	200,005196	177,207884	1959,90532
65	65	ASLENSLEETKGR	717,365	17,28	sp|P08779|K1C16_HUMAN	11750,1363	30534,3577	16732,1982	12337,7077	9768,55329	1379,81314	622,649453	175,056667	487,617525
66	66	ASLENSLEETKGR	717,365	17,28	sp|P02533|K1C14_HUMAN	11750,1363	30534,3577	16732,1982	12337,7077	9768,55329	1379,81314	622,649453	175,056667	487,617525
67	67	TFTC[CAM]TAAYPESK	688,313	17,33	sp|P01876|IGHA1_HUMAN	1257,88835	1819,34074	1785,96925	1399,65081	9100,95587	1653,43125	616,68474	351,140384	1451,76344
68	68	YDPEAASAPGSGNPC[CAM]HEASAAQKENAGI	856,886	17,38	sp|P81605|DCD_HUMAN	15441,2046	10154,7294	24927,1033	16911,2897	26423,7868	853,586083	476,372015	655,414096	1441,73907
69	69	LENEIQTYR	583,296	17,41	sp|Q6IFW6|K1C10_RAT	138173,646	112296,823	104727,405	56548,6127	86413,4466	171,626885	279,821142	293,682938	7395,0094
70	70	LENEIQTYR	583,296	17,41	sp|P13645|K1C10_HUMAN	138173,646	112296,823	104727,405	56548,6127	86413,4466	171,626885	279,821142	293,682938	7395,0094
71	71	GSGGLGGAC[CAM]GGAGFGSR	712,833	17,43	sp|P48668|K2C6C_HUMAN	12057,3812	16937,7535	9919,95018	9413,22764	5254,53153	3171,73813	748,019257	611,461936	2894,63551
72	72	GSGGLGGAC[CAM]GGAGFGSR	712,833	17,43	sp|P02538|K2C6A_HUMAN	12057,3812	16937,7535	9919,95018	9413,22764	5254,53153	3171,73813	748,019257	611,461936	2894,63551
73	73	IKFEM[Oxi]EQNLR	662,34	17,45	sp|P35527|K1C9_HUMAN	22732,0953	56263,9447	86353,6422	60193,3509	48856,4754	766,698267	511,242211	348,819289	5304,89854
74	74	IRDWYQR	518,769	17,48	sp|Q04695|K1C17_HUMAN	1338,98358	29724,4088	2161,57462	863,079417	518,446901	19396,6115	14345,6917	213,426537	261,914317
75	75	IRDWYQR	518,769	17,48	sp|P08779|K1C16_HUMAN	1338,98358	29724,4088	2161,57462	863,079417	518,446901	19396,6115	14345,6917	213,426537	261,914317
76	76	IRDWYQR	518,769	17,48	sp|P02533|K1C14_HUMAN	1338,98358	29724,4088	2161,57462	863,079417	518,446901	19396,6115	14345,6917	213,426537	261,914317
77	77	APSTYGGGLSVSSSR	713,35	17,54	sp|P02533|K1C14_HUMAN	22917,1283	40474,9929	32530,8572	18769,3036	18199,3665	1567,86445	121,421286	2736,65791	3559,58915
78	78	IKEWYEK	498,263	17,55	sp|Q6IFW6|K1C10_RAT	2441,23661	1383,59592	1709,82724	612,947195	1975,48153	100482,209	22460,2267	8247,85073	4447,10632
79	79	APSTYGGGLSVSSR	669,837	17,58	sp|P08779|K1C16_HUMAN	13600,4606	20770,1826	17427,8261	6788,76676	8687,38401	4613,08329	1033,06827	532,209289	1368,36051
80	80	LAADDFR	404,202	17,6	sp|Q6IFX1|K1C24_RAT	5330,01873	864,638344	1138,19704	1905,46544	136692,416	121149,829	58978,2194	14861,5603	3793,58599
81	81	LAADDFR	404,202	17,6	sp|P13646|K1C13_HUMAN	5330,01873	864,638344	1138,19704	1905,46544	136692,416	121149,829	58978,2194	14861,5603	3793,58599
82	82	LAADDFR	404,202	17,6	sp|Q6IFW6|K1C10_RAT	5330,01873	864,638344	1138,19704	1905,46544	136692,416	121149,829	58978,2194	14861,5603	3793,58599
83	83	LAADDFR	404,202	17,6	sp|Q15323|K1H1_HUMAN	5330,01873	864,638344	1138,19704	1905,46544	136692,416	121149,829	58978,2194	14861,5603	3793,58599
84	84	LAADDFR	404,202	17,6	sp|Q04695|K1C17_HUMAN	5330,01873	864,638344	1138,19704	1905,46544	136692,416	121149,829	58978,2194	14861,5603	3793,58599
85	85	LAADDFR	404,202	17,6	sp|P08779|K1C16_HUMAN	5330,01873	864,638344	1138,19704	1905,46544	136692,416	121149,829	58978,2194	14861,5603	3793,58599
86	86	LAADDFR	404,202	17,6	sp|P02533|K1C14_HUMAN	5330,01873	864,638344	1138,19704	1905,46544	136692,416	121149,829	58978,2194	14861,5603	3793,58599
87	87	IEISSPC[CAM]C[CAM]PR	609,783	17,63	sp|Q5T749|KPRP_HUMAN	4914,32818	17603,5926	12688,1929	8106,17256	28097,3194	1740,14172	899,794671	282,070405	2505,83546
88	88	LEQEIATYR	561,793	17,64	sp|P13646|K1C13_HUMAN	10266,9418	13973,2081	12991,6264	5319,32846	6481,21133	198,828229	621,71887	556,507257	3878,76533
89	89	LEQEIATYR	561,793	17,64	sp|Q04695|K1C17_HUMAN	10266,9418	13973,2081	12991,6264	5319,32846	6481,21133	198,828229	621,71887	556,507257	3878,76533
90	90	LEQEIATYR	561,793	17,64	sp|P08779|K1C16_HUMAN	10266,9418	13973,2081	12991,6264	5319,32846	6481,21133	198,828229	621,71887	556,507257	3878,76533
91	91	LEQEIATYR	561,793	17,64	sp|P02533|K1C14_HUMAN	10266,9418	13973,2081	12991,6264	5319,32846	6481,21133	198,828229	621,71887	556,507257	3878,76533
92	92	SLASSLPGQTK	544,801	17,77	sp|P15252|REF_HEVBR	17284,5623	12859,315	20142,1954	12684,651	27966,695	884,822722	1596,70748	156,353238	1718,13053
93	93	CAM.SGSGWSSSRGPY	642,789	17,79	sp|Q86YZ3|HORN_HUMAN	4129,80592	1506,88093	5204,04394	2001,34098	4972,73223	2817,89909	783,684328	563,001934	4146,98602
94	94	GSGSGWSSSRGPY	642,789	17,79	sp|Q86YZ3|HORN_HUMAN	4129,80592	1506,88093	5204,04394	2001,34098	4972,73223	2817,89909	783,684328	563,001934	4146,98602
95	95	VPDESEVVVER	629,32	17,8	sp|Q08188|TGM3_HUMAN	3247,20242	4752,53566	3628,61048	10685,743	6373,73772	921,467351	400,895309	263,836253	6949,89862
96	96	ALDGINSGITHAGR	691,362	17,9	sp|Q6UWP8|SBSN_HUMAN	4118,26041	785,547644	3246,67861	7180,99269	4438,26179	1757,68866	978,741189	415,094938	849,761745
97	97	TNAENEFVTIKK	697,369	18,04	sp|P04264|K2C1_HUMAN	484578,881	841379,315	767985,963	388961,216	679595,206	1680,56734	617,772279	690,64846	45094,8339
98	98	IEDDNDNAPYFEHR	578,918	18,1	sp|Q08554|DSC1_HUMAN	3319,95005	151,213952	4072,89629	6871,51605	684,277603	2984,17058	4694,91635	2046,0299	876,108618
99	99	VATVSLPR	421,755	18,11	sp|P00761|TRYP_PIG	8260638,54	7596313,06	10263701,2	139,818173	12770285,3	345,582977	237,916564	122,366329	536,016861
100	100	PGQ.QSESSHGWTGPSTGVR	828,372	18,14	sp|P20930|FILA_HUMAN	4933,50676	479,993699	3216,49991	35063,5705	5932,22312	1549,91968	626,4132	381,624428	2626,22823
101	101	TAAENDFVTLKK	668,859	18,23	sp|P35908|K22E_HUMAN	192004,673	168993,868	170925,828	90314,3102	96496,6594	1486,30801	3491,02981	247,778272	3054,50633
102	102	LKYENEVALR	617,843	18,27	sp|Q6IFW6|K1C10_RAT	638887,027	612182,703	682330,799	310595,881	476294,454	1600,20506	1783,49682	203,428714	27409,0715
103	103	LKYENEVALR	617,843	18,27	sp|P13645|K1C10_HUMAN	638887,027	612182,703	682330,799	310595,881	476294,454	1600,20506	1783,49682	203,428714	27409,0715
104	104	AYLEEEC[CAM]PATLRK	790,393	18,35	sp|P25311|ZA2G_HUMAN	945,490052	2617,98383	2791,70791	3790,7989	7184,51024	15152,5652	1052,9383	163,951259	3234,85711
105	105	LLEGEDAHLSSQQASGQSYSSR	1175,55	18,37	sp|P08779|K1C16_HUMAN	671,099442	565,26708	430,320628	803,405168	355,774284	408,056929	6546,9029	144,013413	264,708797
106	106	VTATDLDEPDTLHTR	842,418	18,4	sp|Q08554|DSC1_HUMAN	43076,6403	16344,2922	56542,0285	76050,5557	61127,4278	370,760414	1090,16011	439,76163	30398,1297
107	107	ALEESNYELEGK	691,327	18,43	sp|P13645|K1C10_HUMAN	493943,942	321579,382	340094,873	154759,293	390758,264	2270,5173	2480,46076	319,582937	674,215853
108	108	LASYLDKVR	532,808	18,47	sp|Q04695|K1C17_HUMAN	314464,315	380853,45	313837,639	150326,128	384256,528	322,897841	751,220916	240,401455	2535,49264
109	109	LASYLDKVR	532,808	18,47	sp|P08779|K1C16_HUMAN	314464,315	380853,45	313837,639	150326,128	384256,528	322,897841	751,220916	240,401455	2535,49264
110	110	LASYLDKVR	532,808	18,47	sp|P02533|K1C14_HUMAN	314464,315	380853,45	313837,639	150326,128	384256,528	322,897841	751,220916	240,401455	2535,49264

111	111	LASYLDKVR	532,808	18,47	sp|P13645|K1C10_HUMAN	314464,315	380853,45	313837,639	150326,128	384256,528	322,897841	751,220916	240,401455	2535,49264
112	112	TEAESWYQTK	621,787	18,5	sp|P13647|K2C5_HUMAN	3097,79212	11875,5073	4703,50259	3036,88754	4524,21441	898,582489	679,943935	287,384376	349,239188
113	113	ALEE[KXX]SNYELEGK	710,301	18,58	sp|P13645|K1C10_HUMAN	118648,22	62345,9247	60522,7517	8407,08203	80405,1722	971,564298	390,087003	623,215632	1616,94643
114	114	SISISVAR	416,748	18,61	sp|P04264|K2C1_HUMAN	307106,908	380255,589	344433,085	194676,764	340829,134	15412,6729	9600,82254	3290,01623	184,019461
115	115	SLVNLGGSK	437,753	18,66	sp|P04264|K2C1_HUMAN	270951,275	249110,24	256503,902	130188,526	217644,874	323,008677	315,414993	933,715494	12438,2412
116	116	YEELQVTAGR	583,297	18,74	sp|P48668|K2C6C_HUMAN	2745,5897	2573,85006	5763,02422	2800,07053	12858,4484	5926,85067	14205,6419	791,59507	6352,46255
117	117	YEELQVTAGR	583,297	18,74	sp|P02538|K2C6A_HUMAN	2745,5897	2573,85006	5763,02422	2800,07053	12858,4484	5926,85067	14205,6419	791,59507	6352,46255
118	118	TAAENEFVTLKK	675,868	18,82	sp|P48668|K2C6C_HUMAN	28691,1297	56102,0895	38535,9048	18467,7705	10095,555	1378,85049	2504,86529	2400,12216	1772,87922
119	119	TAAENEFVTLKK	675,868	18,82	sp|P02538|K2C6A_HUMAN	28691,1297	56102,0895	38535,9048	18467,7705	10095,555	1378,85049	2504,86529	2400,12216	1772,87922
120	120	CAM.VATVSLPR	450,268	18,88	sp|P00761|TRYP_PIG	120143,83	66833,7311	197927,476	290987,416	152737,138	110219,192	219,098997	199786,708	137340,223
121	121	M[Oxi]SGE[Dhy]C[CAM]APNVSVSVSTSHTTIS	855,057	18,91	sp|P04264|K2C1_HUMAN	6401,69805	83880,592	15112,2232	19066,221	23718,7112	952,600539	423,699096	2148,97618	1137,31409
122	122	M[Oxi]S[Dhy]GEC[CAM]APNVSVSVSTSHTTIS	855,057	18,91	sp|P04264|K2C1_HUMAN	6401,69805	83880,592	15112,2232	19066,221	23718,7112	952,600539	423,699096	2148,97618	1137,31409
123	123	VTVTPAR	421,758	18,92	sp|Q05884|AMY_STRLI	7522,88062	7596297,91	10263700,9	13152993,5	12769653,6	5179833,42	8713097,36	9024869,26	5826118,88
124	124	W[2Ox]TLLQEQGTK	618,326	18,95	sp|P48668|K2C6C_HUMAN	23083,8264	39106,2732	12657,1146	6700,74219	19549,9841	537519,25	240272,14	70069,5496	6199,17491
125	125	CRM.TAAENEFVTLKK	697,369	18,95	sp|P48668|K2C6C_HUMAN	21915,5103	39795,0045	27694,2272	2276,60273	15571,763	899113,623	489457,903	1088,16197	7303,09661
126	126	W[2Ox]TLLQEQGTK	618,326	18,95	sp|P02538|K2C6A_HUMAN	23083,8264	39106,2732	12657,1146	6700,74219	19549,9841	537519,25	240272,14	70069,5496	6199,17491
127	127	CRM.TAAENEFVTLKK	697,369	18,95	sp|P02538|K2C6A_HUMAN	21915,5103	39795,0045	27694,2272	2276,60273	15571,763	899113,623	489457,903	1088,16197	7303,09661
128	128	W[2Ox]TLLQEQGTK	618,326	18,95	sp|P13647|K2C5_HUMAN	23083,8264	39106,2732	12657,1146	6700,74219	19549,9841	537519,25	240272,14	70069,5496	6199,17491
129	129	TNETYGKLEAVQYK	822,421	18,98	sp|P01040|CYTA_HUMAN	6363,81641	5717,62547	7238,75393	17552,3523	9587,90972	1176,68074	1558,37419	755,223804	2869,8085
130	130	GQTGGDVNVE[CAM]MDAAPGVDLSR	1073	19,01	sp|P08779|K1C16_HUMAN	6316,51419	15658,7858	6990,51066	2207,4618	1468,19199	300,45538	2024,81868	852,056312	891,899138
131	131	CAM.GQTGGDVNVEMDAAPGVDLSR	715,669	19,01	sp|P08779|K1C16_HUMAN	10990,7414	22079,0866	7981,06758	5011,33887	3244,23279	9852,79178	2434,53994	2137,12749	1882,16787
132	132	W[2Ox]LQGSQELPR	623,315	19,04	sp|P01877|IGHA2_HUMAN	1603,76966	4976,55103	2646,53609	2275,7618	36238,943	2507,2199	875,339482	895,963228	3137,18021
133	133	W[2Ox]LQGSQELPR	623,315	19,04	sp|P01876|IGHA1_HUMAN	1603,76966	4976,55103	2646,53609	2275,7618	36238,943	2507,2199	875,339482	895,963228	3137,18021
134	134	QLDSIVGER	508,772	19,08	sp|P48668|K2C6C_HUMAN	1545,02698	911,188908	1871,40519	441,747587	1251,7653	33191,0774	45174,7417	9016,96341	3582,64785
135	135	QLDSIVGER	508,772	19,08	sp|P02538|K2C6A_HUMAN	1545,02698	911,188908	1871,40519	441,747587	1251,7653	33191,0774	45174,7417	9016,96341	3582,64785
136	136	QLDSIVGER	508,772	19,08	sp|P13647|K2C5_HUMAN	1545,02698	911,188908	1871,40519	441,747587	1251,7653	33191,0774	45174,7417	9016,96341	3582,64785
137	137	TGSENDFVVLKK	668,858	19,13	sp|Q7Z794|K2C1B_HUMAN	7150,57615	6488,74834	16229,4368	4082,64157	14251,8016	3344,90555	145659,103	1822,2648	7997,24168
138	138	RLLEGEDAHLSSSQFSSGSQSSR	822,394	19,14	sp|P02533|K1C14_HUMAN	5667,53526	6206,37209	1611,1186	293,067193	897,195511	1062,8887	982,345637	886,821056	2523,72721
139	139	TRLEQEIATYRR	512,614	19,17	sp|Q04695|K1C17_HUMAN	10869,8488	39138,0597	16260,5793	13698,1202	13338,0262	781,026486	573,375643	382,840602	848,407848
140	140	TRLEQEIATYRR	512,614	19,17	sp|P08779|K1C16_HUMAN	10869,8488	39138,0597	16260,5793	13698,1202	13338,0262	781,026486	573,375643	382,840602	848,407848
141	141	TRLEQEIATYRR	512,614	19,17	sp|P02533|K1C14_HUMAN	10869,8488	39138,0597	16260,5793	13698,1202	13338,0262	781,026486	573,375643	382,840602	848,407848
142	142	ALEEANAD[NaX]LEVK	662,323	19,26	sp|P13646|K1C13_HUMAN	11600,4505	18779,4649	6701,33701	1664,59678	4454,48726	697,559484	1012,97982	384,764085	6976,69475
143	143	ALEEANAD[NaX]LEVK	662,323	19,26	sp|P08779|K1C16_HUMAN	11600,4505	18779,4649	6701,33701	1664,59678	4454,48726	697,559484	1012,97982	384,764085	6976,69475
144	144	ALEEANAD[NaX]LEVK	662,323	19,26	sp|P02533|K1C14_HUMAN	11600,4505	18779,4649	6701,33701	1664,59678	4454,48726	697,559484	1012,97982	384,764085	6976,69475
145	145	ALEEANADLEVK	651,333	19,3	sp|P13646|K1C13_HUMAN	58963,8493	111236,489	63061,0871	42042,2036	33404,9851	2033,47423	3266,74599	874,487739	7424,63992
146	146	ALEEANADLEVK	651,333	19,3	sp|P08779|K1C16_HUMAN	58963,8493	111236,489	63061,0871	42042,2036	33404,9851	2033,47423	3266,74599	874,487739	7424,63992
147	147	ALEEANADLEVK	651,333	19,3	sp|P02533|K1C14_HUMAN	58963,8493	111236,489	63061,0871	42042,2036	33404,9851	2033,47423	3266,74599	874,487739	7424,63992
148	148	RQSVEADINGLR	679,362	19,33	sp|P13646|K1C13_HUMAN	32690,4784	40645,7976	33063,6762	2267,32739	1652,40927	467,128205	1570,5265	710,077596	955,634178
149	149	RQSVEADINGLR	679,362	19,33	sp|Q6IFW6|K1C10_RAT	32690,4784	40645,7976	33063,6762	2267,32739	1652,40927	467,128205	1570,5265	710,077596	955,634178
150	150	RQSVEADINGLR	679,362	19,33	sp|P13645|K1C10_HUMAN	32690,4784	40645,7976	33063,6762	2267,32739	1652,40927	467,128205	1570,5265	710,077596	955,634178
151	151	VQALEEANNDLENK	793,885	19,34	sp|P35527|K1C9_HUMAN	40327,3643	138268,836	143393,741	80369,0869	73810,8018	11254,229	3343,98106	3454,24334	16598,6923
152	152	TEELNKEVASNSELVQSSR	707,354	19,38	sp|P08779|K1C16_HUMAN	13962,0415	27747,0496	1490,36941	3982,41692	2516,70332	1696,06404	581,122083	1635,2864	7596,18663
153	153	AAR.VATVSLPR	435,774	19,4	sp|P00761|TRYP_PIG	5819194,61	5937851,97	7592202,94	3330928,33	9091064,01	4512444,39	14670,5702	515,531435	4360231,44
154	154	QVLDNLTM[Oxi]EK	603,805	19,4	sp|P35527|K1C9_HUMAN	29424,4629	59550,8976	77015,774	56245,1601	44310,2221	681,275945	3939,03843	4470,48987	13303,5279
155	155	TKYETELNLR	633,838	19,43	sp|P02533|K1C14_HUMAN	832,650657	2665,17166	759,264784	3463,60606	1260,70008	1125,82929	51841,3244	14361,2797	1923,35284
156	156	ASLENSLEETK	610,803	19,46	sp|P08779|K1C16_HUMAN	19638,8783	27761,1092	828,15346	11028,574	10484,0393	2460,83568	30105,048	10982,3922	5716,0875
157	157	ASLENSLEETK	610,803	19,46	sp|P02533|K1C14_HUMAN	19638,8783	27761,1092	828,15346	11028,574	10484,0393	2460,83568	30105,048	10982,3922	5716,0875
158	158	M[Oxi]T[Dhy]LDDFR	448,202	19,46	sp|P35527|K1C9_HUMAN	13973,4859	87125,3762	20614,6798	18434,2085	20827,5742	97731,6654	116808,753	1456,04452	4552,50904
159	159	ALEEANTELEVK	673,347	19,53	sp|Q04695|K1C17_HUMAN	6636,9002	15124,1744	11329,9213	3942,60023	7083,21376	1844,0764	2220,39904	2058,58586	5950,13834
160	160	W[Oxi]TLLQEQGTK	610,32	19,59	sp|P48668|K2C6C_HUMAN	16685,8878	36207,404	12189,4901	12222,7108	10748,4697	15678,4971	2378,8358	156,778183	3955,40653
161	161	W[Oxi]TLLQEQGTK	610,32	19,59	sp|P02538|K2C6A_HUMAN	16685,8878	36207,404	12189,4901	12222,7108	10748,4697	15678,4971	2378,8358	156,778183	3955,40653
162	162	W[Oxi]TLLQEQGTK	610,32	19,59	sp|P13647|K2C5_HUMAN	16685,8878	36207,404	12189,4901	12222,7108	10748,4697	15678,4971	2378,8358	156,778183	3955,40653
163	163	VDLHLPR	425,251	19,61	sp|P29508|SPB3_HUMAN	14117,1891	1163,94675	6755,70657	14249,7075	10327,9232	308,620564	1210,73124	311,386806	3316,31307
164	164	NFDIPK	733,387	19,65	sp|P12273|PIP_HUMAN	9590,69781	10368,7585	10626,891	9663,58916	19012,9664	4826,09132	2027,22612	424,613384	3644,58487
165	165	QEYEQLIAK	561,295	19,69	sp|P35527|K1C9_HUMAN	46523,0453	102582,7	78357,4599	44928,9583	55174,3074	68353,9503	956,346405	530,822991	10553,465
166	166	GAVHDVKDVLDS	627,82	19,72	sp|P81605|DCD_HUMAN	18024,8942	13433,818	26011,7116	1655,98705	36656,9798	4747,76215	9379,26927	841,075013	3974,77027
167	167	TEELNREVATNSELVQSGK	702,026	19,76	sp|Q04695|K1C17_HUMAN	805,983657	2172,82815	379,437423	1053,64084	552,832902	4612,4391	1993,59234	123,363332	2882,71631
168	168	TEELNREVATNSELVQSGK	702,026	19,76	sp|P02533|K1C14_HUMAN	805,983657	2172,82815	379,437423	1053,64084	552,832902	4612,4391	1993,59234	123,363332	2882,71631
169	169	FLEQQNQVLQTK	738,396	19,84	sp|Q7Z794|K2C1B_HUMAN	1099172,48	1578916,84	1335444,87	681892,482	1049596,59	2765,51668	1261,99897	320,333495	94976,5368
170	170	FLEQQNQVLQTK	738,396	19,84	sp|P35908|K22E_HUMAN	1099172,48	1578916,84	1335444,87	681892,482	1049596,59	2765,51668	1261,99897	320,333495	94976,5368
171	171	FLEQQNQVLQTK	738,396	19,84	sp|P04264|K2C1_HUMAN	1099172,48	1578916,84	1335444,87	681892,482	1049596,59	2765,51668	1261,99897	320,333495	94976,5368
172	172	LVSVSLPR	435,774	19,89	sp|C6E4P5|RL5_GEOSM	6894,08728	5938204	7120,94005	2573,34494	2889,60984	4513092,63	3595665,77	7429728,71	4359812,62
173	173	LLEGEDAHLSSSQFSSGSQSSR	770,361	19,92	sp|P02533|K1C14_HUMAN	16179,0238	37669,7915	29406,9791	21630,0329	17314,2357	2646,3662	3368,82336	3830,2794	9896,98489
174	174	NHKEEMSQLT[CAM]GQNSGDVNVEINVAPGK	853,173	19,92	sp|P35527|K1C9_HUMAN	778,993388	25291,9262	8102,67885	1636,53697	13852,8048	446,364858	808,330268	314,922136	806,987918
175	175	VYTVDLGR	461,753	19,93	sp|P01833|PIGR_HUMAN	184,823558	472,787935	166,432058	893,523775	9180,61801	585,254989	2230,62294	1322,94508	2166,93987
176	176	FLE[NaX]QQNQVLQTK	749,386	19,95	sp|Q7Z794|K2C1B_HUMAN	101341,684	105149,252	34150,1025	22631,648	26152,0412	2343,38812	629,525914	221,909397	15814,5248
177	177	VSLAGAC[CAM]GVGGYGSR	705,843	19,95	sp|P13647|K2C5_HUMAN	39854,8606	71631,3475	61642,0226	37494,5544	51835,0082	6170,73443	2133,79293	1473,51341	4032,93903
178	178	FLE[NaX]QQNQVLQTK	749,386	19,95	sp|P35908|K22E_HUMAN	101341,684	105149,252	34150,1025	22631,648	26152,0412	2343,38812	629,525914	221,909397	15814,5248
179	179	FLE[NaX]QQNQVLQTK	749,386	19,95	sp|P04264|K2C1_HUMAN	101341,684	105149,252	34150,1025	22631,648	26152,0412	2343,38812	629,525914	221,909397	15814,5248
180	180	FLEQQNKVLETK	738,394	19,96	sp|Q8N1N4|K2C78_HUMAN	1098820,05	1578254,43	1334146,34	681758,28	1049638,87	1251717,71	1367,55301	438,655971	95113,3613
181	181	FLEQQNKVLETK	738,394	19,96	sp|P19013|K2C4_HUMAN	1098820,05	1578254,43	1334146,34	681758,28	1049638,87	1251717,71	1367,55301	438,655971	95113,3613
182	182	FLEQQNKVLETK	738,394	19,96	sp|P02538|K2C6A_HUMAN	1098820,05	1578254,43	1334146,34	681758,28	1049638,87	1251717,71	1367,55301	438,655971	95113,3613
183	183	AIGGGLSSVGGGSSTIK	724,39	19,98	sp|P48668|K2C6C_HUMAN	22513,5196	26709,7567	24792,1877	11645,3774	7633,6726	542,00816	796,091774	691,024492	3149,1496
184	184	AIGGGLSSVGGGSSTIK	724,39	19,98	sp|P02538|K2C6A_HUMAN	22513,5196	26709,7567	24792,1877	11645,3774	7633,6726	542,00816	796,091774	691,024492	3149,1496
185	185	GQVGGDVNVE[CAM]MDAAPGVDLSR	715,008	20,06	sp|P02533|K1C14_HUMAN	17647,852	33103,564	27019,5624	19155,1748	13844,6015	3714,56703	342,891982	1324,36389	5762,26549
186	186	NVIEGPVFRPGSK	700,387	20,09	sp|Q02413|DSG1_HUMAN	2968,52339	3395,41998	3575,56285	17997,5396	890,332853	5903,05408	451,797998	168,944912	3545,84977
187	187	KDAEAWFNEK	619,296	20,11	sp|Q6IFW6|K1C10_RAT	30624,9546	25126,3825	24357,8664	1177,60788	24497,934	3659,27699	895,213711	1836,83985	2143,31523
188	188	SISGPGVDKEPFN	673,836	20,14	sp|Q08554|DSC1_HUMAN	2332,68518	3406,58422	6551,02235	4048,59207	5408,02344	4607,24753	8454,53867	1060,56923	4129,41096
189	189	TTAENEFVM[Oxi]LKK	713,866	20,19	sp|P13647|K2C5_HUMAN	5321,54379	500,766452	5288,93968	1013,21198	3855,59838	4128,27258	22675,8251	6185,7003	3782,35571
190	190	NILDRQDPPSVVVTSHQAPGEK	796,418	20,2	sp|P25311|ZA2G_HUMAN	7204,03019	4701,45628	3556,07495	8831,64426	9795,94457	2526,74156	1294,49208	481,981156	4654,11154
191	191	VQQLQISVDQHGDNLK	911,474	20,2	sp|P19013|K2C4_HUMAN	6279,73246	249,49476	515,068463	731,468756	435,497469	491,746669	189,645997	162,724175	820,511984
192	192	LQSSSASYGGGFGGGSC[CAM]QLGGGR	1073,98	20,27	sp|P13646|K1C13_HUMAN	4467,88314	3697,47131	472,862349	447,034683	686,238971	377,641145	1210,45901	226,831566	1733,85297
193	193	GGGLSVSSSRF	527,269	20,33	sp|P02533|K1C14_HUMAN	853,908496	1531,87102	2650,69901	8654,72765	822,861197	3542,80137	1167,34942	987,674211	2993,26014
194	194	NVQDAIADAEQR	665,324	20,37	sp|P35908|K22E_HUMAN	84370,351	108365,11	95767,4778	65311,7776	64513,8374	2272,86902	2043,68802	1112,3355	3000,5018
195	195	YEELQITAGR	590,304	20,37	sp|P04264|K2C1_HUMAN	690319,256	931014,728	742991,04	391233,45	787463,719	4360,10054	777,317056	1864,60448	385,654979
196	196	QGVDADINGLR	579,3	20,4	sp|P35527|K1C9_HUMAN	2613,19088	1815,64485	2346,38428	876,664076	1625,10592	171774,057	159152,493	20135,3088	2279,61741
197	197	YLDGLTAER	519,266	20,41	sp|P35908|K22E_HUMAN	206408,991	187356,294	166034,061	100058,652	131327,456	6248,79477	7116,57519	5233,72508	5659,30315
198	198	QFIENGSEFAQK	699,338	20,43	sp|Q9GZZ8|LACRT_HUMAN	2599,01689	6742,42339	25775,0798	2781,78199	18752,4319	34069,9005	953,870237	451,292799	2135,26625
199	199	NVQD[NaX]AIADAEQR	676,315	20,47	sp|P35908|K22E_HUMAN	16277,8725	14440,0601	10168,8831	4044,7739	6392,54899	4776,19893	1047,82965	941,32715	2266,34325
200	200	FLEQ[Dea]QNKVLETK	739,398	20,48	sp|Q8N1N4|K2C78_HUMAN	26989,6795	27423,8652	20349,8852	10972,5205	18529,4257	436347,709	229335,346	52453,1307	2665,62268
201	201	FLEQ[Dea]QNKVLETK	739,398	20,48	sp|P19013|K2C4_HUMAN	26989,6795	27423,8652	20349,8852	10972,5205	18529,4257	436347,709	229335,346	52453,1307	2665,62268
202	202	FLEQ[Dea]QNKVLETK	739,398	20,48	sp|P02538|K2C6A_HUMAN	26989,6795	27423,8652	20349,8852	10972,5205	18529,4257	436347,709	229335,346	52453,1307	2665,62268
203	203	IEISELNR	487,269	20,49	sp|P35908|K22E_HUMAN	3892,13616	6191,24066	2497,42922	1832,59001	3095,80544	436825,275	270743,696	74808,8553	3593,52889
204	204	IEISELNR	487,269	20,49	sp|P04264|K2C1_HUMAN	3892,13616	6191,24066	2497,42922	1832,59001	3095,80544	436825,275	270743,696	74808,8553	3593,52889
205	205	STSGFC[CAM]QGGGVGGFGGGR	822,861	20,53	sp|Q7Z794|K2C1B_HUMAN	1361,95763	2337,12244	6888,42221	2417,30118	10689,3706	3715,2965	1189,89647	3344,4961	2501,51039
206	206	ISISTSGGSFR	556,29	20,54	sp|P13647|K2C5_HUMAN	36252,9755	14852,1164	73348,5681	1197,83501	61911,9245	85885,2781	963,306105	33920,5645	2073,49154
207	207	QRPSEIKDYSPYFK	586,633	20,57	sp|P08779|K1C16_HUMAN	2660,913	17846,1947	6617,20489	794,938585	2290,10474	614,903685	612,127105	122,692225	2628,92391
208	208	QRP[Oxi]AEIKDYSPYFK	586,633	20,57	sp|P02533|K1C14_HUMAN	2660,913	17846,1947	6617,20489	794,938585	2290,10474	614,903685	612,127105	122,692225	2628,92391
209	209	FSSC[CAM]GGGGGSFGAGGGFGSR	883,371	20,6	sp|P04264|K2C1_HUMAN	104035,577	223025,753	202393,139	97913,3727	201836,046	2643,41623	4642,46328	891,113308	20605,2361
210	210	CAM.YQ[Dea]ELQITAGR	618,814	20,63	sp|Q7Z794|K2C1B_HUMAN	3498,02638	5605,67261	8358,89347	5789,49498	8867,90367	4732,82908	2672,14699	528,194646	2160,44765
211	211	FSTQC[CAM]QYQGSYSSC[CAM]GPQFQSR	835,351	20,64	sp|Q5T749|KPRP_HUMAN	3725,81472	5113,95322	8566,03304	3043,50017	13871,4337	5995,82322	2750,04075	200,590616	1894,23392
212	212	GLGVGFGSGGGSSSSVK	720,358	20,64	sp|P13647|K2C5_HUMAN	7580,05472	9034,63953	610,903794	1015,79043	7613,54645	6651,16223	990,256409	1680,96442	8531,61812
213	213	TQVVAGTNYYIK	678,861	20,66	sp|P01040|CYTA_HUMAN	14268,6096	6788,2595	20699,2421	29614,5631	25174,7761	3901,23629	860,810465	518,029165	5221,97836
214	214	TRLEQEIATYR	690,367	20,67	sp|P13646|K1C13_HUMAN	41012,8798	71348,8352	2515,4531	24802,0663	18346,7553	23465,5198	2008,59792	5305,06824	7479,48636
215	215	TRLEQEIATYR	690,367	20,67	sp|Q04695|K1C17_HUMAN	41012,8798	71348,8352	2515,4531	24802,0663	18346,7553	23465,5198	2008,59792	5305,06824	7479,48636
216	216	TRLEQEIATYR	690,367	20,67	sp|P08779|K1C16_HUMAN	41012,8798	71348,8352	2515,4531	24802,0663	18346,7553	23465,5198	2008,59792	5305,06824	7479,48636
217	217	TRLEQEIATYR	690,367	20,67	sp|P02533|K1C14_HUMAN	41012,8798	71348,8352	2515,4531	24802,0663	18346,7553	23465,5198	2008,59792	5305,06824	7479,48636
218	218	IRLENEIQTYR	717,889	20,67	sp|P13645|K1C10_HUMAN	564457,784	603834,05	731,102873	277230,968	377248,015	2543,05857	978,07557	498,486272	24714,3294
219	219	AGDKDDITEPAVC[CAM]ALR	865,921	20,67	sp|Q8WNW3|PLAK_PIG	5687,25119	8451,60507	9113,51553	2849,52976	3332,96005	8865,49904	6553,21298	3154,88054	7395,9307
220	220	SFSTASAITPSVSR	705,864	20,7	sp|P13647|K2C5_HUMAN	2200,60788	24179,5264	30977,0039	19186,2039	35161,3278	17703,6177	10214,079	4896,28108	8275,29231
221	221	FLEQQN[Dea]QVLQTK	738,887	20,7	sp|Q7Z794|K2C1B_HUMAN	43081,5398	70963,8626	54388,9197	25136,0634	40438,8527	24647,3144	511792,325	4029,9685	3684,8987

222	222	FLEQQN[Dea]QVLQTK	738,887	20,7	sp|P35908|K22E_HUMAN	43081,5398	70963,8626	54388,9197	25136,0634	40438,8527	24647,3144	511792,523	4029,9685	3684,8987
223	223	FLEQQN[Dea]QVLQTK	738,887	20,7	sp|P04264|K2C1_HUMAN	43081,5398	70963,8626	54388,9197	25136,0634	40438,8527	24647,3144	511792,523	4029,9685	3684,8987
224	224	QSVEADINGLRR	679,363	20,74	sp|P13646|K1C13_HUMAN	11252,1757	1923,08143	15606,0354	527,581883	18253,3889	795824,896	309287,118	94835,5731	7207,15499
225	225	QSVEADINGLRR	679,363	20,74	sp|Q6IFW6|K1C10_RAT	11252,1757	1923,08143	15606,0354	527,581883	18253,3889	795824,896	309287,118	94835,5731	7207,15499
226	226	QSVEADINGLRR	679,363	20,74	sp|P13645|K1C10_HUMAN	11252,1757	1923,08143	15606,0354	527,581883	18253,3889	795824,896	309287,118	94835,5731	7207,15499
227	227	YEELQVTVGR	597,311	20,74	sp|P35908|K22E_HUMAN	245161,167	202832,568	171538,034	106445,777	105320,259	810405,896	830537991	1172,66429	6587,85303
228	228	NLQIDPSIQR	592,326	20,76	sp|P13647|K2C5_HUMAN	2338,67783	4960,53034	4072,82033	1093,66525	3781,94873	16928,2699	813,308891	504,526039	1697,03251
229	229	ITAVC[CAM]KVPDESEVVVER	644,004	20,76	sp|Q08188|TGM3_HUMAN	5256,38948	5788,5594	1585,0397	2944,29253	7603,6887	2222,02763	1108,58838	377,731786	3755,96461
230	230	ILLDVK	350,735	20,77	sp|Q04695|K1C17_HUMAN	8732,37541	46546,5939	20475,8894	14809,8982	15081,9782	179,29423	10351,2719	629,63033	959,211934
231	231	ILLDVK	350,735	20,77	sp|P08779|K1C16_HUMAN	8732,37541	46546,5939	20475,8894	14809,8982	15081,9782	179,29423	10351,2719	629,63033	959,211934
232	232	ILLDVK	350,735	20,77	sp|P02533|K1C14_HUMAN	8732,37541	46546,5939	20475,8894	14809,8982	15081,9782	179,29423	10351,2719	629,63033	959,211934
233	233	ISIGGGSC[CAM]AISGGYGSR	799,882	20,81	sp|P48668|K2C6C_HUMAN	31632,585	65888,7324	1934,74733	448,539143	210,039312	5983,79409	609,89463	2236,22608	4560,61518
234	234	ISIGGGSC[CAM]AISGGYGSR	799,882	20,81	sp|P02538|K2C6A_HUMAN	31632,585	65888,7324	1934,74733	448,539143	210,039312	5983,79409	609,89463	2236,22608	4560,61518
235	235	QRPAEIKDYSPYFK	871,449	20,93	sp|P02533|K1C14_HUMAN	1179,543	13618,3101	14986,7759	1676,624	5742,19407	1717,21811	124,835292	581,141749	4436,68824
236	236	YQ[Dea]ELQITAGR	590,303	20,94	sp|Q7Z794|K2C1B_HUMAN	690267,392	931229,168	745514,738	5637,5556	787821,111	819523,321	368295,876	68914,4161	1580,3027
237	237	NVAVPLYNR	523,294	20,94	sp|P15252|REF_HEVBR	10470,7431	1981,68188	1326,72882	2972,69664	558,58589	29158,6312	49541,8108	18785,0792	5995,43778
238	238	FLEQQ[Dea]NQVLQTK	738,887	21,04	sp|Q7Z794|K2C1B_HUMAN	16134,9811	35909,0735	23560,6714	670,860653	16089,1798	46308,2311	14757,4048	2981,7518	2301,5109
239	239	FLEQQ[Dea]NQVLQTK	738,887	21,04	sp|P35908|K22E_HUMAN	16134,9811	35909,0735	23560,6714	670,860653	16089,1798	46308,2311	14757,4048	2981,7518	2301,5109
240	240	ALDPEISSGEGLR	672,344	21,08	sp|Q08554|DSC1_HUMAN	12060,4325	8035,42592	9903,51255	17323,2427	11047,5545	7592,87461	1474,45118	546,720912	3219,26908
241	241	FLEQQNQ[Dea]VLQTK	738,889	21,1	sp|Q7Z794|K2C1B_HUMAN	16152,1064	35669,8776	23678,2602	13276,2098	16082,4613	46324,5387	14761,5475	2980,86293	6344,57641
242	242	DTGDIFC[CAM]TR	542,741	21,11	sp|Q08554|DSC1_HUMAN	356,138921	1327,10138	905,090674	5047,54098	2425,95202	507,3493	4456,02428	200,016421	9254,6831
243	243	LLNDEDPVVVTK	671,362	21,11	sp|Q8WNW3|PLAK_PIG	17931,3956	35417,0008	20573,6863	17531,9962	14607,8346	2780,26067	5248,32803	460,186336	8678,71836
244	244	QVDLLSAEQM[Oxi]R	653,328	21,11	sp|Q7Z794|K2C1B_HUMAN	11949,7136	2579,15087	8242,28675	1050,44973	10713,636	995,300347	1665,58517	402,036011	8333,6102
245	245	AYLEEEC[CAM]PATLR	726,346	21,11	sp|P25311|ZA2G_HUMAN	400,304295	2556,60302	3884,88274	309,695466	11066,6112	3084,75075	3188,82129	196,64516	1894,88622
246	246	YIEKDTGDIFC[CAM]TR	539,925	21,17	sp|Q08554|DSC1_HUMAN	1893,09368	4968,0856	1565,39781	10188,9549	3668,06168	3160,39959	4861,12791	330,288617	9653,11941
247	247	GQTGGDVNVEM[Oxi]DAAPGVDLSR	1052,48	21,21	sp|P08779|K1C16_HUMAN	3921,37974	2114,30638	7386,84704	4230,47268	1914,95531	10052,6856	978,821382	242,210666	3182,2077
248	248	FLEQ[Dea]QNQVLQTK	738,887	21,27	sp|Q7Z794|K2C1B_HUMAN	822,816387	36081,8264	23500,9086	13296,1806	16107,1808	21778,3708	25803,0795	5349,14474	3964,86032
249	249	FLEQ[Dea]QNQVLQTK	738,887	21,27	sp|P35908|K22E_HUMAN	822,816387	36081,8264	23500,9086	13296,1806	16107,1808	21778,3708	25803,0795	5349,14474	3964,86032
250	250	CRM.TAAENEFVTLK	633,322	21,27	sp|P48668|K2C6C_HUMAN	162282,804	214588,755	423,080776	148912,359	193069,607	1146,98718	1310,43706	683,624009	13419,8811
251	251	CRM.TAAENEFVTLK	633,322	21,27	sp|P02538|K2C6A_HUMAN	162282,804	214588,755	423,080776	148912,359	193069,607	1146,98718	1310,43706	683,624009	13419,8811
252	252	TNAENEFVTIK	633,322	21,27	sp|P04264|K2C1_HUMAN	162282,804	214588,755	423,080776	148912,359	193069,607	1146,98718	1310,43706	683,624009	13419,8811
253	253	NLQIDPAIQR	584,329	21,3	sp|P48668|K2C6C_HUMAN	882,469191	2363,05247	1448,38191	10592,5069	5181,39046	905,189555	892,150848	175,456602	1602,02625
254	254	CRM.TAAENE[NaX]FVTLK	644,314	21,31	sp|P48668|K2C6C_HUMAN	18607,2875	397,088496	13843,0022	6636,52446	1601,28714	1639,28648	1446,81126	897,285675	3338,44295
255	255	CRM.TAAENE[NaX]FVTLK	644,314	21,31	sp|P02538|K2C6A_HUMAN	18607,2875	397,088496	13843,0022	6636,52446	1601,28714	1639,28648	1446,81126	897,285675	3338,44295
256	256	SC[CAM]AAAGTEC[CAM]LISGW[2Ox]GNTK[A	971,933	21,33	sp|P00761|TRYP_PIG	3775,58349	5295,04748	886,229941	725,74897	9129,98563	1828,05371	740,596719	172,750487	1416,87765
257	257	YLTWASR	448,735	21,41	sp|P01877|IGHA2_HUMAN	291,931775	2108,63971	683,394329	416,084117	23006,0936	339,205713	506,97554	178,962516	1051,08508
258	258	YLTWASR	448,735	21,41	sp|P01876|IGHA1_HUMAN	291,931775	2108,63971	683,394329	416,084117	23006,0936	339,205713	506,97554	178,962516	1051,08508
259	259	CAM.FLEQQNQVLQTK	766,906	21,43	sp|Q7Z794|K2C1B_HUMAN	1035,95151	3431,92014	1657,8603	909,415218	3389,11299	2954,7693	14201,2567	2619,69301	2087,1081
260	260	CAM.FLEQQNQVLQTK	766,906	21,43	sp|P35908|K22E_HUMAN	1035,95151	3431,92014	1657,8603	909,415218	3389,11299	2954,7693	14201,2567	2619,69301	2087,1081
261	261	LLETEC[CAM]PQYIR	711,359	21,48	sp|P05109|S10A8_HUMAN	417,733821	3391,06974	8615,07276	6688,50211	9467,54925	1237,21341	1409,21688	531,854539	4292,39829
262	262	RDPTAEQFQEELEK	573,944	21,54	sp|P31944|CASPE_HUMAN	2468,29086	6219,19074	5845,99874	18469,3739	10278,4509	4856,1648	4174,92399	673,81816	1492,53877
263	263	QLQNIIQATSR	636,357	21,56	sp|P15924|DESP_HUMAN	2073,1871	9517,04281	7349,21523	1670,61564	6853,64675	1075,91281	1440,65802	269,784124	1811,29771
264	264	ALEEQLQQIR	614,337	21,61	sp|Q6IFW6|K1C10_RAT	1131,55825	778,592569	26044,0739	5141,39365	9440,79029	1987,59868	930,436247	3089,89095	1744,80654
265	265	CAM.GQVGGEINVEMDAAPGVDLSR	1086,03	21,63	sp|Q04695|K1C17_HUMAN	159,19244	3650,2427	1904,89381	343,16161	155,831984	292,940805	389,01376	238,805066	388,584568
266	266	ESTLHLVLR	534,314	21,64	sp|P68202|RS27A_PLUXY	14539,4812	6027,76232	10729,95	24503,5818	20070,4087	3525,05256	5466,53026	6406,2009	5951,59415
267	267	SISGPGVDKEPF	616,812	21,82	sp|Q08554|DSC1_HUMAN	4290,27796	11177,9973	8814,18576	30666,0069	7977,49804	6360,26587	12011,7169	1157,30673	7517,08177
268	268	DAEAW[2Ox]FNEK	571,243	21,87	sp|Q6IFW6|K1C10_RAT	3683,95706	13914,6069	4471,72781	5484,46976	14747,231	484,496815	1817,03467	1212,37599	9982,24914
269	269	STSGGTAALGC[CAM]LVK	661,342	21,88	sp|P01857|IGHG1_HUMAN	1126,05096	3183,17967	5114,73796	9576,76562	4223,72718	3653,05891	612,973506	1260,47452	5464,50435
270	270	VIAPSSSLPTSLTIHHPR	638,359	21,89	sp|Q02413|DSG1_HUMAN	1037,67258	7312,18723	6984,49365	1221,75693	3561,68605	1012,1367	302,029764	2075,06349	6800,30225
271	271	KVPQVSTPTLVEVSR	547,318	21,92	sp|P02769|ALBU_BOVIN	15368,2302	151,939711	17767,5708	15334,0628	19776,3841	612,352719	754,64729	283,833109	11989,2889
272	272	KVPQVSTPTLVEVSR	547,318	21,92	sp|P02768|ALBU_HUMAN	15368,2302	151,939711	17767,5708	15334,0628	19776,3841	612,352719	754,64729	283,833109	11989,2889
273	273	IISNASC[CAM]TTNC[CAM]LAPLAK	917,463	22,02	sp|P04406|G3P_HUMAN	2304,25849	4075,90972	5430,29649	4748,78013	5835,22064	950,092795	1473,47614	181,875373	875,195625
274	274	GLTGGFGSHSVC[CAM]GGFR	798,371	22,05	sp|O43790|KRT86_HUMAN	1085,51529	951,959431	2813,39136	959,618317	363,134742	3723,20758	3533,19212	486,545808	6044,11439
275	275	LVNEVTEFAK	575,313	22,05	sp|P02768|ALBU_HUMAN	8957,63573	11363,643	13280,8389	12160,9761	18190,5488	1050,19796	1115,06034	551,297408	7827,16109
276	276	QSLEASLAETEGR	695,845	22,07	sp|Q6IFW6|K1C10_RAT	255305,064	329759,065	274765,004	160053,327	279686,821	2500,42627	5197,01221	2338,96627	27626,361
277	277	QSLEASLAETEGR	695,845	22,07	sp|P13645|K1C10_HUMAN	255305,064	329759,065	274765,004	160053,327	279686,821	2500,42627	5197,01221	2338,96627	27626,361
278	278	FQNALLVR	480,786	22,12	sp|P49065|ALBU_RABIT	3835,41107	1842,47262	1810,6606	7801,66178	9490,17055	364,883361	471,380667	198,118692	24594,4626
279	279	FQNALLVR	480,786	22,12	sp|P02768|ALBU_HUMAN	3835,41107	1842,47262	1810,6606	7801,66178	9490,17055	364,883361	471,380667	198,118692	24594,4626
280	280	SNLC[CAM]ALC[CAM]IGDEQGENK	904,402	22,14	sp|P02788|TRFL_HUMAN	808,918775	1564,51642	751,494176	630,839025	8144,80117	1002,33908	3189,74805	274,337183	7488,04661
281	281	DGAGDVAFIR	510,76	22,14	sp|P02788|TRFL_HUMAN	1230,86212	700,851463	1167,06089	304,374455	12277,4286	206,033249	189,071303	141,669425	3543,132
282	282	SGGGFSSGSAGIINYQR	829,399	22,15	sp|P04264|K2C1_HUMAN	84476,0866	74265,8086	113076,707	33074,7823	158238,827	24484,5114	1798,21005	526,118133	12513,2755
283	283	FASFIDKVR	541,803	22,19	sp|P48668|K2C6C_HUMAN	274746,54	375547,286	206616,56	12122,657	161858,048	249,653809	1501,21009	575,205588	40178,7661
284	284	FASFIDKVR	541,803	22,19	sp|Q8N1N4|K2C78_HUMAN	274746,54	375547,286	206616,56	12122,657	161858,048	249,653809	1501,21009	575,205588	40178,7661
285	285	FASFIDKVR	541,803	22,19	sp|Q7Z794|K2C1B_HUMAN	274746,54	375547,286	206616,56	12122,657	161858,048	249,653809	1501,21009	575,205588	40178,7661
286	286	FASFIDKVR	541,803	22,19	sp|Q9NSB2|KRT84_HUMAN	274746,54	375547,286	206616,56	12122,657	161858,048	249,653809	1501,21009	575,205588	40178,7661
287	287	FASFIDKVR	541,803	22,19	sp|P02538|K2C6A_HUMAN	274746,54	375547,286	206616,56	12122,657	161858,048	249,653809	1501,21009	575,205588	40178,7661
288	288	FASFIDKVR	541,803	22,19	sp|P13647|K2C5_HUMAN	274746,54	375547,286	206616,56	12122,657	161858,048	249,653809	1501,21009	575,205588	40178,7661
289	289	LGEHNIDVLEGNEQ	783,873	22,19	sp|P00761|TRYP_PIG	9823,02127	34965,0173	49233,6332	15555,5802	34487,6633	10060,6881	6670,45271	505,260052	32663,4434
290	290	FASFIDKVR	541,803	22,19	sp|P35908|K22E_HUMAN	274746,54	375547,286	206616,56	12122,657	161858,048	249,653809	1501,21009	575,205588	40178,7661
291	291	FASFIDKVR	541,803	22,19	sp|P04264|K2C1_HUMAN	274746,54	375547,286	206616,56	12122,657	161858,048	249,653809	1501,21009	575,205588	40178,7661
292	292	FASFIDK	414,219	22,21	sp|P48668|K2C6C_HUMAN	2820,3202	3068,17648	1812,31227	818,708785	2287,66182	107101,908	50221,4355	12872,3448	8313,11357
293	293	FASFIDK	414,219	22,21	sp|Q8N1N4|K2C78_HUMAN	2820,3202	3068,17648	1812,31227	818,708785	2287,66182	107101,908	50221,4355	12872,3448	8313,11357
294	294	FASFIDK	414,219	22,21	sp|P19013|K2C4_HUMAN	2820,3202	3068,17648	1812,31227	818,708785	2287,66182	107101,908	50221,4355	12872,3448	8313,11357
295	295	FASFIDK	414,219	22,21	sp|Q7Z794|K2C1B_HUMAN	2820,3202	3068,17648	1812,31227	818,708785	2287,66182	107101,908	50221,4355	12872,3448	8313,11357
296	296	FASFIDK	414,219	22,21	sp|Q9NSB2|KRT84_HUMAN	2820,3202	3068,17648	1812,31227	818,708785	2287,66182	107101,908	50221,4355	12872,3448	8313,11357
297	297	FASFIDK	414,219	22,21	sp|P02538|K2C6A_HUMAN	2820,3202	3068,17648	1812,31227	818,708785	2287,66182	107101,908	50221,4355	12872,3448	8313,11357
298	298	FASFIDK	414,219	22,21	sp|P13647|K2C5_HUMAN	2820,3202	3068,17648	1812,31227	818,708785	2287,66182	107101,908	50221,4355	12872,3448	8313,11357
299	299	FASFIDK	414,219	22,21	sp|P35908|K22E_HUMAN	2820,3202	3068,17648	1812,31227	818,708785	2287,66182	107101,908	50221,4355	12872,3448	8313,11357
300	300	TLLDIDNTR	530,785	22,22	sp|P35527|K1C9_HUMAN	228246,411	785105,454	637169,125	450239,045	391854,802	1825,81734	2949,91229	462,02307	67608,355
301	301	GGGFSSGGFSGGSFSR	725,82	22,23	sp|P13645|K1C10_HUMAN	31859,5262	64473,1988	52234,9468	5095,31441	75499,8427	2555,34829	2511,65257	899,601544	7641,85095
302	302	TSFSPC[CAM]VPQC[CAM]QTQGSYGSFTEQHI	930,405	22,25	sp|Q5T749|KPRP_HUMAN	2325,26237	9927,75611	9053,95404	2571,29085	23760,3794	1534,74991	2910,6749	395,198093	2023,99706
303	303	GQVGGDVNVEM[Oxi]DAAPGVDLSR	1051,49	22,28	sp|P02533|K1C14_HUMAN	2644,14451	7858,16865	17176,1085	8938,21925	7840,65327	267,994141	3460,82209	648,725477	4078,32502
304	304	YQ[Dea]ELQVSAQL	590,303	22,31	sp|Q8N1N4|K2C78_HUMAN	4334,10099	1858,23088	2469,03782	411,044245	3009,27925	6043,4594	638,849762	746,883823	22905,7745
305	305	TAAENDFVTLK	604,812	22,36	sp|P35908|K22E_HUMAN	2608,06051	841,592872	2977,63475	601,815386	2652,31854	75445,1199	38886,5694	16526,4174	16202,2232
306	306	CAM.MSGDLSSNVTVSVTSSTISSNVASK	839,078	22,38	sp|P35908|K22E_HUMAN	76048,3849	74990,6704	65304,9495	49660,1675	49241,8019	583,493357	512,379079	210,441297	2133,62143
307	307	QSVEADINGLR	601,313	22,39	sp|P13646|K1C13_HUMAN	5801,77046	8424,49943	12433,6899	838,311383	4690,43155	96117,2431	42768,7977	25134,5952	6836,19568
308	308	QSVEADINGLR	601,313	22,39	sp|Q6IFW6|K1C10_RAT	5801,77046	8424,49943	12433,6899	838,311383	4690,43155	96117,2431	42768,7977	25134,5952	6836,19568
309	309	LGEHNIDVLEGN	655,324	22,39	sp|P00761|TRYP_PIG	3045,95551	8843,91288	14139,0492	15971,7162	16697,2269	6737,22228	996,21177	2169,98206	13723,3248
310	310	ALHFAISEYNK	646,835	22,41	sp|P01037|CYTN_HUMAN	1566,79605	31573,6908	3559,7134	1288,69572	11780,9591	2920,1124	1493,86142	1049,61226	3184,01131
311	311	TRLEQEIATY	612,317	22,44	sp|P13646|K1C13_HUMAN	7980,5593	16445,8475	7891,05057	6766,85323	4556,38157	6324,33829	2489,3668	169,375175	2876,09568
312	312	TRLEQEIATY	612,317	22,44	sp|Q04695|K1C17_HUMAN	7980,5593	16445,8475	7891,05057	6766,85323	4556,38157	6324,33829	2489,3668	169,375175	2876,09568
313	313	TRLEQEIATY	612,317	22,44	sp|P08779|K1C16_HUMAN	7980,5593	16445,8475	7891,05057	6766,85323	4556,38157	6324,33829	2489,3668	169,375175	2876,09568
314	314	TRLEQEIATY	612,317	22,44	sp|P02533|K1C14_HUMAN	7980,5593	16445,8475	7891,05057	6766,85323	4556,38157	6324,33829	2489,3668	169,375175	2876,09568
315	315	RVLDELTLAR	593,352	22,46	sp|Q04695|K1C17_HUMAN	10007,3854	19288,8994	11732,5601	5834,87123	6253,549	990,871429	1331,22499	425,937181	10216,6266
316	316	RVLDELTLAR	593,352	22,46	sp|P08779|K1C16_HUMAN	10007,3854	19288,8994	11732,5601	5834,87123	6253,549	990,871429	1331,22499	425,937181	10216,6266
317	317	RVLDELTLAR	593,352	22,46	sp|P02533|K1C14_HUMAN	10007,3854	19288,8994	11732,5601	5834,87123	6253,549	990,871429	1331,22499	425,937181	10216,6266
318	318	VGDFVATDLDTGRPSTTVR	669,678	22,52	sp|Q02413|DSG1_HUMAN	3492,21288	5414,36152	6958,99188	10393,372	6697,29415	1035,63176	383,439382	124,002226	2258,20658
319	319	NKLEGLEDALQK	679,369	22,54	sp|P48668|K2C6C_HUMAN	23504,7279	75463,7258	31998,4241	22791,1914	9835,34036	1761,54608	1100,90885	673,733999	13751,5364
320	320	NKLEGLEDALQK	679,369	22,54	sp|P02538|K2C6A_HUMAN	23504,7279	75463,7258	31998,4241	22791,1914	9835,34036	1761,54608	1100,90885	673,733999	13751,5364
321	321	GGVVC[CAM]GDLC[CAM]ASTTAPVVSTR	1003,98	22,55	sp|O43790|KRT86_HUMAN	1056,81622	5481,56215	2946,01305	4225,02432	582,469407	3915,66061	483,120637	329,793017	1376,26738
322	322	FKVEESYDLKDTLR	581,638	22,56	sp|P29508|SPB3_HUMAN	7621,09827	2992,35278	121,727269	6758,60213	267,464409	793,115297	465,753494	222,033257	5473,74815
323	323	C[CAM]PVEIPPIR	540,796	22,64	sp|Q5T749|KPRP_HUMAN	7635,75909	23080,3455	17129,5069	7665,95255	43840,7745	1202,20671	2247,91815	1502,4275	2919,37497
324	324	QISNLQQSISDAEQR	858,929	22,64	sp|P04264|K2C1_HUMAN	149490,004	323188,407	297697,181	217159,599	181123,421	4951,82832	2102,30935	1072,76357	21308,0722
325	325	C[CAM]LAENAGDVAFVK	697,343	22,65	sp|P02788|TRFL_HUMAN	2341,63464	1991,02109	2837,49767	1492,19149	12527,9854	41354,8183	12196,3012	7701,42164	13674,7847
326	326	GLVLSGVLHK	511,822	22,66	sp|P29508|SPB3_HUMAN	16823,7796	1524,57699	2525,58211	14383,0089	7946,84032	337,748124	529,653429	425,557533	3682,92897
327	327	LGEHNIDVLE	569,79	22,67	sp|P00761|TRYP_PIG	6319,22887	9141,13431	9694,52736	10787,4609	10192,5935	4990,3994	296,571591	1348,30859	15450,1293
328	328	ALNSM[Oxi]GQDLERPLELR	619,988	22,68	sp|Q02413|DSG1_HUMAN	15549,6444	16872,5762	22357,8416	22321,6761	17779,0619	1471,4541	2589,75068	1501,78859	2742,39623
329	329	GVAGGSVAVLC[CAM]PYNR	760,387	22,69	sp|P01833|PIGR_HUMAN	1245,84647	1436,1033	1393,58482	3046,71731	10267,48	5160,41873	924,477905	197,645177	6149,75195
330	330	PGQ.QKWEAPVYVQR	758,384	22,69	sp|P25311|ZA2G_HUMAN	3965,80433	1475,5309	1279,97612	8054,5341	888,894179	3751,26227	2919,6288	1391,7969	4676,23748
331	331	SKEEAEALYHSKYEELQVTVGR	856,099	22,71	sp|P35908|K22E_HUMAN	14952,6505	11335,6218	10137,8606	6509,5757	5163,49826	668,416829	613,942995	239,285486	3617,0277
332	332	LAELEEALQKAKQDM	572,956	22,74	sp|P13647|K2C5_HUMAN	67803,0964	127241,243	90142,1633	103049,368	93290,0704	75643,0043	1600,04057	609,859334	1361,97178

333	333	LAELEEALQK	572,318	22,74	sp|P13647|K2C5_HUMAN	16293,9461	26358,7407	33280,5281	33510,6522	17254,8655	4212,35986	1307,44402	606,171146	19712,2915
334	334	LGEHNIDVLEGNE	719,844	22,75	sp|P00761|TRYP_PIG	4313,76412	6014,26911	7616,46292	5446,03181	4285,60752	3701,85488	4388,10393	1086,96484	9931,5561
335	335	QITVNDLPVGR	606,342	22,76	sp|Q8K3U7|PRDX2_CRIGR	6442,25225	10871,4974	14936,3931	7709,38905	6772,31912	896,001586	1801,37654	724,81518	4465,32438
336	336	LGKDAVEDLESVGK	730,386	22,81	sp|P81605|DCD_HUMAN	51445,3002	64003,1911	81682,091	89000,5568	98955,3643	2816,27563	4451,47735	1137,97538	16886,3054
337	337	LGEHNIDVLEG	598,301	22,84	sp|P00761|TRYP_PIG	22844,477	30348,6775	33572,3272	28320,7033	33562,4963	1092,98519	1064,14933	1317,08291	23321,4722
338	338	DASIQVVSAIR	579,828	22,94	sp|P15252|REF_HEVBR	54099,7497	54106,32	61441,6053	60705,048	118456,308	2323,87098	3794,23596	748,042432	6798,262
339	339	CRM.LKYENEVALR	639,347	22,95	sp|Q6IFW6|K1C10_RAT	25511,1361	25086,5363	23818,5466	9356,68179	18748,0358	7219,86512	9406,72529	9237,62776	2410,57811
340	340	YYYDGKDYIEFNK	573,265	22,99	sp|P25311|ZA2G_HUMAN	9525,03233	7147,36095	6963,35475	7128,32224	16125,9952	67930,9956	1015,48367	7560,15654	10673,5137
341	341	ALEEQLQQIRAE	714,38	23,02	sp|Q6IFW6|K1C10_RAT	1356,21232	1374,84427	6551,19694	12312,8781	4382,00335	4868,98171	2095,52624	1054,67006	6301,10415
342	342	DAVEDLESVGK	581,285	23,05	sp|P81605|DCD_HUMAN	37728,1008	45014,2303	44106,8118	59228,257	63335,663	2922,83276	712,40085	526,876006	12249,7048
343	343	VLDELTLTK	516,303	23,07	sp|P13645|K1C10_HUMAN	828037,74	822115,195	612958,062	402639,586	652046,112	268,543933	993,432369	1867,72398	83283,5305
344	344	LLRDYQELMNTK	508,602	23,1	sp|P04264|K2C1_HUMAN	657,768631	51723,6539	7167,32359	9008,0526	15932,1085	198,876311	142,112943	124,764784	1013,21043
345	345	SKAEAESLYQSKYEELQITAGR	834,422	23,11	sp|P04264|K2C1_HUMAN	28716,8482	31757,6002	34793,1424	19657,6547	36679,934	2350,48923	1793,68472	177,646947	2724,98139
346	346	AELIVQPELK	570,336	23,18	sp|P15924|DESP_HUMAN	626329,925	314331,981	110986,131	356542,601	123408,298	92060,502	1173,80463	85014,3511	108795,869
347	347	DASGATFTWTPSSGK	756,853	23,25	sp|P01877|IGHA2_HUMAN	704,131986	4311,67315	1207,40514	2732,70974	28392,9118	3819,43347	2375,27706	2104,38946	7453,63469
348	348	AELIVQ[Dea]PELK	570,838	23,3	sp|P15924|DESP_HUMAN	1262,46713	10405,626	8198,86853	2171,26206	8903,1065	1331,65803	7276,85868	3393,4396	3416,52558
349	349	ANSVSC[CAM]WSGPGFR	712,823	23,31	sp|Q9NSB2|KRT84_HUMAN	981,509239	1748,76071	2508,15656	302,842152	1239,3054	15917,8697	4226,33536	846,973161	2668,47309
350	350	ENPVKGSPGVPSFAAGPPISEGK	741,39	23,38	sp|Q15517|CDSN_HUMAN	1201,8412	3160,04447	4886,21083	1048,24583	1509,33307	1647,38246	1852,78828	4078,89876	15631,8806
351	351	PGQ.QS[CAM]VEADINGLRR	699,361	23,38	sp|P13646|K1C13_HUMAN	6824,84322	718,87339	19593,001	4641,49751	3326,93672	206,8235	996,384671	1948,02797	14993,7989
352	352	PGQ.QRPAEIKDYSPYFK	862,936	23,43	sp|P02533|K1C14_HUMAN	7105,55394	14849,1989	10278,1911	567,999896	5418,65852	2985,9487	4208,84596	972,546476	1592,70152
353	353	ALNSIIDVYHK	636,849	23,47	sp|P05109|S10A8_HUMAN	10468,7705	9695,2672	18211,9391	7015,2914	16176,717	3459,21307	5556,33784	1902,79827	7038,21036
354	354	NNFLDREQYGQY	773,85	23,48	sp|Q02413|DSG1_HUMAN	2577,35623	1715,67471	2799,91165	11016,149	1216,18553	7285,91348	6080,90017	1883,59356	5183,68873
355	355	PGQ.QGVDADINGLR	570,787	23,51	sp|P35527|K1C9_HUMAN	53377,4149	214383,673	194020,286	147338,319	115683,2	620,674088	851,474074	2037,15451	20467,5403
356	356	PGQ.QGVDADIN[Dea]GLR	571,287	23,55	sp|P35527|K1C9_HUMAN	104698,009	138718,999	123361,067	159923,082	85548,1103	44297,9408	25518,9446	19323,7728	45762,8547
357	357	ALYAN[Dea]LEPKAEQY	755,877	23,56	sp|O82803|SRPP_HEVBR	2403,64981	3045,94671	10327,2147	3263,61294	6879,22559	2149,91404	454,60967	1004,73236	13142,2944
358	358	ALDEVLALTK	536,775	23,58	sp|Q72KU3|PYRD_THET2	1008,70453	2415,8925	255,125754	911,120514	1231,67579	2036,43521	438,929975	665,526023	779715,748
359	359	IIEGGIYDADLNDER	846,908	23,58	sp|P09228|CYTT_HUMAN	1365,0584	9053,23401	2669,93868	492,362318	2070,09326	4493,65127	1714,34382	1483,24925	2941,20334
360	360	AIGGGLSSVGGGSSTIKY	805,923	23,62	sp|P48668|K2C6C_HUMAN	8771,26832	14064,0803	11735,384	2952,68219	9430,4608	3821,81685	2240,43506	1677,54757	1853,72326
361	361	AIGGGLSSVGGGSSTIKY	805,923	23,62	sp|P02538|K2C6A_HUMAN	8771,26832	14064,0803	11735,384	2952,68219	9430,4608	3821,81685	2240,43506	1677,54757	1853,72326
362	362	PGQ.QSVEADINGLRR	670,85	23,63	sp|P13646|K1C13_HUMAN	694878,079	826580,895	649004,163	382776,361	553478,392	2819,97669	2503,49037	3570,01674	58041,4088
363	363	PGQ.QSVEADINGLRR	670,85	23,63	sp|Q6IFW6|K1C10_RAT	694878,079	826580,895	649004,163	382776,361	553478,392	2819,97669	2503,49037	3570,01674	58041,4088
364	364	PGQ.QSVEADINGLRR	670,85	23,63	sp|P13645|K1C10_HUMAN	694878,079	826580,895	649004,163	382776,361	553478,392	2819,97669	2503,49037	3570,01674	58041,4088
365	365	IGFIEEVK	467,766	23,67	sp|Q96P63|SPB12_HUMAN	3748,22632	5824,44666	5610,19458	10566,9334	8615,45808	424,330239	408,633926	140,688089	2488,34121
366	366	LC[CAM]EGVGPVNISVSSSR	830,92	23,68	sp|Q9NSB2|KRT84_HUMAN	1302,25002	2245,16619	2172,05356	1042,86059	2323,55099	42769,7029	189,807718	632,696603	4546,85127
367	367	NGSDC[CAM]PDKFC[CAM]LFQSETK	678,298	23,68	sp|P02788|TRFL_HUMAN	1776,60173	3646,90003	2999,0735	1883,6483	9195,08145	881,665109	3324,64505	471,696517	11580,2814
368	368	VLDELTLAR	515,301	23,76	sp|Q04695|K1C17_HUMAN	88885,7477	217667,114	120544,949	96153,1877	75346,6483	815,685766	2721,13933	1652,30939	10538,0605
369	369	GALQNIIPASTGAAK	706,399	23,76	sp|P04406|G3P_HUMAN	21019,4513	18557,0924	27456,0136	30394,5562	34253,2345	3009,07928	234,053852	1454,47732	19972,0867
370	370	VLDELTLAR	515,301	23,76	sp|P08779|K1C16_HUMAN	88885,7477	217667,114	120544,949	96153,1877	75346,6483	815,685766	2721,13933	1652,30939	10538,0605
371	371	VLDELTLAR	515,301	23,76	sp|P02533|K1C14_HUMAN	88885,7477	217667,114	120544,949	96153,1877	75346,6483	815,685766	2721,13933	1652,30939	10538,0605
372	372	DAEAWFNEK	555,249	23,78	sp|Q6IFW6|K1C10_RAT	526157,124	407601,877	315352,68	179635,7	457128,332	687,074831	4008,03622	1928,93203	27914,6067
373	373	Y[Iod]Q[Dea]ELQITAGR	653,252	23,78	sp|Q7Z794|K2C1B_HUMAN	6920,52385	5534,33791	5079,64448	2255,72137	3860,92132	2119,63782	2295,34661	372,597843	3502,33484
374	374	DAEAWFNEK	555,249	23,78	sp|P13645|K1C10_HUMAN	526157,124	407601,877	315352,68	179635,7	457128,332	687,074831	4008,03622	1928,93203	27914,6067
375	375	VAPEEHPVLLTEAPLNPK	652,028	23,86	sp|Q5ZMQ2|ACTG_CHICK	13517,2125	6708,25088	3094,32357	8193,94492	14212,0359	2804,99891	3594,82176	1474,0222	1541,3985
376	376	VLDELTLT[-2H]K	515,3	23,88	sp|P13645|K1C10_HUMAN	88924,2771	217663,429	120571,607	96319,2629	75386,2425	1186,76351	2757,45965	1851,54394	7188,93916
377	377	QC[CAM]ANLQNAIADAEQR	851,4	23,91	sp|P13647|K2C5_HUMAN	2170,77926	7375,75393	7012,88262	1213,87111	3128,91262	461,354709	5877,06682	1136,35743	3376,04495
378	378	PGQ.QRPSEIKDYSPYFK	870,935	23,97	sp|P08779|K1C16_HUMAN	2724,96633	3144,82323	1703,18315	979,824016	1261,98335	2148,51781	10400,4345	927,595707	7360,58293
379	379	PGQ.QRP[Oxi]AEIKDYSPYFK	870,935	23,97	sp|P02533|K1C14_HUMAN	2724,96633	3144,82323	1703,18315	979,824016	1261,98335	2148,51781	10400,4345	927,595707	7360,58293
380	380	GMQD[2CM]LVEDFKNKYEDEINKR	647,065	24,01	sp|P02538|K2C6A_HUMAN	6600,40565	7264,92599	9740,92874	1692,55962	9888,30386	1359,64262	724,3056	456,345201	1736,23503
381	381	CAM.NMQDLVEDFKNKYEDEINKR	647,065	24,01	sp|P13647|K2C5_HUMAN	6600,40565	7264,92599	9740,92874	1692,55962	9888,30386	1359,64262	724,3056	456,345201	1736,23503
382	382	GPSVFPLAPSSK	593,828	24,03	sp|P01857|IGHG1_HUMAN	6473,39605	4649,80912	5849,2279	21152,1122	7718,68147	3047,10102	1884,96164	3524,8553	9077,93621
383	383	FSYIPNGALK	555,303	24,03	sp|P15252|REF_HEVBR	270969,976	58851,3502	62068,1509	48974,6666	113294,133	141003,734	3224,63131	2382,76123	45739,041
384	384	GALQ[Dea]NIIPASTGAAK	706,891	24,16	sp|P04406|G3P_HUMAN	5115,52182	2276,08658	7290,18979	18085,1071	3602,40193	26268,0883	2527,04962	3302,45494	8296,32023
385	385	SC[CAM]AAAGTEC[CAM]LISGW[Oxi]GNTK[A	963,936	24,18	sp|P00761|TRYP_PIG	5350,9378	10192,204	1678,787	513,155447	13815,2383	1222,04978	1068,56084	2009,78635	981,939882
386	386	M[Oxi]S[Dhy]GDLSSNVTVSVTSSTISSNVASK	819,398	24,18	sp|P35908|K22E_HUMAN	4077,00542	12100,7707	7246,60314	4931,67011	6161,60823	2380,38991	196,778769	1729,20522	5604,17284
387	387	DALNIETAIK	544,304	24,18	sp|Q6TEQ7|ANXA2_CANLF	411,162017	4961,29962	6515,65934	9224,3553	6154,7318	1545,34939	2355,88231	513,976447	10136,5248
388	388	GSPGVPSFAAGPPISEGK	827,926	24,19	sp|Q15517|CDSN_HUMAN	10400,2304	13981,1858	8619,18443	17914,3243	10790,0532	3187,18145	1963,06575	416,374599	5595,96937
389	389	TAFQEALDAAGDK	668,825	24,19	sp|Q5R9M3|THIO_PONAB	14752,9488	22748,8312	14575,778	18944,594	24273,7673	7641,46557	3614,68505	642,455894	7745,97827
390	390	TLNNKFASFIDK	466,585	24,21	sp|P48668|K2C6C_HUMAN	2502,89753	5009,80475	929,094459	534,230589	2624,62158	521,186337	725,561407	211,749074	1737,90103
391	391	TLNNKFASFIDK	466,585	24,21	sp|Q9NSB2|KRT84_HUMAN	2502,89753	5009,80475	929,094459	534,230589	2624,62158	521,186337	725,561407	211,749074	1737,90103
392	392	TLNNKFASFIDK	466,585	24,21	sp|P02538|K2C6A_HUMAN	2502,89753	5009,80475	929,094459	534,230589	2624,62158	521,186337	725,561407	211,749074	1737,90103
393	393	TLNNKFASFIDK	466,585	24,21	sp|P13647|K2C5_HUMAN	2502,89753	5009,80475	929,094459	534,230589	2624,62158	521,186337	725,561407	211,749074	1737,90103
394	394	DISGPLKPGVDTIE	720,879	24,21	sp|O82803|SRPP_HEVBR	1252,94068	5053,88066	13589,7024	8712,43952	8852,22318	2536,2542	7634,11693	2649,29354	5474,91804
395	395	DVDAAYLNKVELEAK	839,438	24,26	sp|P19013|K2C4_HUMAN	9494,32272	838,753827	2188,13687	1321,91228	962,691223	6172,24042	1399,23325	405,462862	5443,49664
396	396	GGC[CAM]ITLISSEGYVSSK	829,408	24,31	sp|P01833|PIGR_HUMAN	2811,35618	2347,57317	4587,39423	1022,07871	18627,5253	2631,57675	1530,03569	1654,50921	8250,35333
397	397	TNSILFYGR	535,786	24,32	sp|P29508|SPB3_HUMAN	16326,2531	1232,93365	4101,52408	11939,7761	5162,99205	497,612418	604,708434	470,074447	2751,2228
398	398	DPTAEQFQEELEK	782,362	24,32	sp|P31944|CASPE_HUMAN	8303,89588	6830,75908	1048,62622	18400,8934	14646,4321	1988,10537	3295,21353	1869,87677	4758,84949
399	399	TYVVTGNM[Oxi]GSNDKVGDFVATDLDTGRPST	848,166	24,34	sp|Q02413|DSG1_HUMAN	4468,90731	3646,54998	4143,23508	1912,89849	4194,83765	823,302226	514,296324	530,531088	1525,94628
400	400	VPTANVSVVDLTC[CAM]R	765,901	24,36	sp|P04406|G3P_HUMAN	23602,2078	33681,4129	25893,0967	12182,2527	28410,9605	1778,30114	1710,60401	2594,21873	4878,57235
401	401	GQVGGEINVEM[Oxi]DAAPGVDLSR	1065,51	24,4	sp|Q04695|K1C17_HUMAN	193,467323	314,033447	311,351588	485,665558	179,947365	1410,60076	2614,91793	162,872399	3132,93802
402	402	VM[Oxi]GNNPADLLAVDSR	794,392	24,43	sp|Q02413|DSG1_HUMAN	2249,47873	7430,983	1795,83782	6732,07199	1557,29929	1943,23843	1414,32947	641,83912	8267,27633
403	403	CAM.TMQNLEIELQSQLSM[Oxi]K	655,992	24,45	sp|P02533|K1C14_HUMAN	7182,16095	1204,27956	10678,3345	7016,48001	4108,63434	2883,75696	3198,54295	483,574797	1675,19531
404	404	EIKIEISELNR	672,381	24,49	sp|P35908|K22E_HUMAN	1682,82823	6965,88368	541,403524	1206,07115	1424,53211	1634,0629	24936,8189	7238,5538	2239,44999
405	405	VDALM[Oxi]DEINFM[Oxi]K	729,337	24,54	sp|P13647|K2C5_HUMAN	13938,9682	5959,42749	16659,871	773,040474	14216,0759	1959,42417	4978,6921	507,357607	8645,39136
406	406	IEVLEEELR	565,31	24,54	sp|P15924|DESP_HUMAN	9362,65796	27943,6324	18433,8473	1200,31832	10001,2535	6670,23755	6795,60841	1120,11029	5162,59802
407	407	KGGSFQLNELQGLK	759,916	24,54	sp|P02788|TRFL_HUMAN	5382,6422	1360,88441	1948,81726	714,981227	14691,41	1286,60212	565,490383	653,926763	3725,87189
408	408	SVITFGSYSPR	607,315	24,61	sp|Q9NSB2|KRT84_HUMAN	7272,98278	5399,94262	12817,0991	8987,11193	6595,91237	62186,7074	4596,1042	85248,7248	20169,8276
409	409	NNQFASFIDK	592,293	24,63	sp|Q8N1N4|K2C78_HUMAN	31256,5649	26892,0017	15455,8088	77865,5128	35997,9655	37526,6001	13885,5658	14323,982	28056,1674
410	410	LALDVEIATYRK	696,398	24,65	sp|P48668|K2C6C_HUMAN	54529,2797	66993,7032	54156,4357	29677,6793	35565,3671	3599,2623	9054,69304	7003,36336	11008,9545
411	411	LALDVEIATYRK	696,398	24,65	sp|P02538|K2C6A_HUMAN	54529,2797	66993,7032	54156,4357	29677,6793	35565,3671	3599,2623	9054,69304	7003,36336	11008,9545
412	412	LALDVEIATYRK	696,398	24,65	sp|P13647|K2C5_HUMAN	54529,2797	66993,7032	54156,4357	29677,6793	35565,3671	3599,2623	9054,69304	7003,36336	11008,9545
413	413	LALDVEIATYRK	696,398	24,65	sp|P35908|K22E_HUMAN	54529,2797	66993,7032	54156,4357	29677,6793	35565,3671	3599,2623	9054,69304	7003,36336	11008,9545
414	414	SSSSGGVGLGGGLSVGGSGFSASSGR	1086,02	24,67	sp|P13647|K2C5_HUMAN	785,403951	3886,8757	2181,54514	2295,7073	1369,96671	1981,43324	1322,03926	587,822421	5388,22047
415	415	CRM.VATVSLPR	443,261	24,7	sp|P00761|TRYP_PIG	211262,918	226750,992	267214,595	233235,562	332835,044	10786,5527	11860,6613	3505,66845	139911,05
416	416	SC[CAM]AAAGTEC[CAM]LISGW[Kyn]GNTK[A	957,937	24,7	sp|P00761|TRYP_PIG	36804,529	106825,375	39691,7289	32842,5251	105395,941	4241,43994	6777,14771	2946,03514	6828,88089
417	417	ENFPNFLSAC[CAM]DKK	785,372	24,82	sp|P31151|S10A7_HUMAN	1461,75754	2067,02565	18519,4905	17093,4661	8402,01964	10674,0039	1084,13138	1425,49828	4025,99088
418	418	LNVEVDAAPTVDLNR	813,426	24,84	sp|Q15323|K1H1_HUMAN	637,40225	667,389948	842,445003	1401,21006	1384,67365	6445,1548	2293,46863	213,286418	124748,216
419	419	KDSPQTIPTYTDALHV	595,973	24,86	sp|P31944|CASPE_HUMAN	3142,11261	4658,86416	4006,94647	13110,6631	4882,7699	5061,96775	3408,44083	4580,86529	1573,15624
420	420	FSYIPN[Dea]GALK	555,796	24,92	sp|P15252|REF_HEVBR	7099,12014	4560,28588	6904,15963	7481,61648	13232,1196	1763,93747	68433,6705	426,983769	6869,79158
421	421	YLGPQYVAGITNLKK	555,651	24,93	sp|P02788|TRFL_HUMAN	174,030185	1338,64711	788,940302	1462,94416	25574,7993	390,83593	1147,45559	155,285244	4338,12432
422	422	GSLGGGFSSGGFSGGSFSR	854,39	24,98	sp|P13645|K1C10_HUMAN	792734,812	1040349,11	576105,442	366730,237	870967,651	787,55858	1427,50431	947,951167	61443,7619
423	423	ELGIC[CAM]PDDAAVIPIKNNR	998,017	25	sp|P12273|PIP_HUMAN	351,630521	3356,96436	2978,68939	3871,86226	7536,47836	248,439074	754,842456	455,244309	2144,16881
424	424	VVWC[CAM]AVGEQELR	723,364	25	sp|P02788|TRFL_HUMAN	3733,8073	3812,74893	2989,18178	3940,45264	15764,9347	1381,62843	1068,97646	2079,25057	4745,46192
425	425	DAGAGIALNDHF	600,789	25,02	sp|P04406|G3P_HUMAN	2499,37759	4028,43886	3787,16065	9480,51178	2917,32434	3062,43259	3732,01981	2399,96144	6460,75635
426	426	VTEVEENRIDVEI	772,894	25,02	sp|Q08554|DSC1_HUMAN	3572,27163	1961,35297	1959,50825	19140,1671	3560,57803	3013,51043	1711,60035	674,116181	8941,58867
427	427	FYQTSVESVDFANAPEESR	726	25,05	sp|P29508|SPB3_HUMAN	7278,50833	2279,84081	1901,54546	425,562842	1753,92893	2152,61738	2268,8864	585,388085	6583,15943
428	428	GPSVFPLAPC[CAM]SR	644,331	25,06	sp|P01861|IGHG4_HUMAN	8008,14044	8088,1261	6224,3246	5845,60647	2321,87725	4692,20133	12069,3913	773,994853	16544,6813
429	429	DISGPLKPGVDTIEN[Dea]VVK	628,011	25,06	sp|O82803|SRPP_HEVBR	2263,35286	3834,73773	4982,30341	2798,80341	9787,75509	2711,27271	1409,74406	2695,47644	3651,14224
430	430	FNWYVDGVEVHNAK	839,404	25,09	sp|P01861|IGHG4_HUMAN	6050,48125	1112,00509	3965,71992	9327,04041	800,533753	3956,12602	3575,42328	495,245399	6126,68692
431	431	FNWYVDGVEVHNAK	839,404	25,09	sp|P01857|IGHG1_HUMAN	6050,48125	1112,00509	3965,71992	9327,04041	800,533753	3956,12602	3575,42328	495,245399	6126,68692
432	432	EVQLVESGGGLVQPGGSLR	941,504	25,13	sp|P01777|HV316_HUMAN	989,927875	1622,08075	7472,90596	3664,38786	9866,92363	1142,84436	1113,26868	370,035734	3432,83239
433	433	C[CAM]LAYDFYPGK	617,286	25,15	sp|P25311|ZA2G_HUMAN	6808,3512	6330,8026	5095,8032	10620,762	19002,0123	5665,51851	3302,8926	2439,22424	14186,6875
434	434	LADLEC[CAM]ALQQAK	680,35	25,17	sp|Q9NSB2|KRT84_HUMAN	10702,0958	145,269314	756,293277	7022,12662	2585,94726	46374,4832	15466,4284	4168,31476	9622,239
435	435	ALYDAELSQM[Oxi]QTHISDTSVVLSM[Oxi]DNI	1024,14	25,19	sp|P48668|K2C6C_HUMAN	1022,96641	1387,43017	6831,53256	1593,50711	1353,01183	1074,19109	1185,85895	402,834592	725,847849
436	436	ALYDAELSQM[Oxi]QTHISDTSVVLSM[Oxi]DNI	1024,14	25,19	sp|P02538|K2C6A_HUMAN	1022,96641	1387,43017	6831,53256	1593,50711	1353,01183	1074,19109	1185,85895	402,834592	725,847849
437	437	W[2Ox]ELLQ[Dea]QVDTSTR	754,875	25,19	sp|P04264|K2C1_HUMAN	216225,86	495190,769	340259,857	179572,316	250978,899	4082,27807	3070,01515	1888,39364	13485,3385
438	438	GLVGIEFK	431,755	25,22	sp|P15924|DESP_HUMAN	3394,61601	8994,10009	5372,33222	5730,50204	8722,18496	663,866496	124,723313	397,751043	2572,64619
439	439	TPEVTC[CAM]VVVDVSHEDPEVK	1070,02	25,32	sp|P01857|IGHG1_HUMAN	1976,39027	844,569477	2035,32535	5898,35484	1984,8752	992,998907	358,699218	162,491878	2084,17529
440	440	SGNVGESGLIDLPGC[CAM]PR	864,421	25,35	sp|P17314|IAAC3_WHEAT	2419,49515	2950,92821	12601,9245	2284,18317	3820,08428	223,933855	2898,94969	923,144421	8190,48989
441	441	PGQ.QLDSIVGER	500,257	25,4	sp|P48668|K2C6C_HUMAN	21917,1946	28954,6007	29541,5432	15381,0205	18639,9139	3083,26176	3165,99142	3367,28675	10310,574
442	442	PGQ.QLDSIVGER	500,257	25,4	sp|P02538|K2C6A_HUMAN	21917,1946	28954,6007	29541,5432	15381,0205	18639,9139	3083,26176	3165,99142	3367,28675	10310,574
443	443	PGQ.QLDSIVGER	500,257	25,4	sp|P13647|K2C5_HUMAN	21917,1946	28954,6007	29541,5432	15381,0205	18639,9139	3083,26176	3165,99142	3367,28675	10310,574

444	444	VDNALQSGNSQESVTEQDSKDSTYSLSSTLTLSK	1207,24	25,42	sp|P01834|IGKC_HUMAN	518,54571	2064,54702	1266,58667	1784,89642	12088,4595	1394,52166	240,605315	217,363282	1513,59486
445	445	DASGVTFTWTPSSGK	770,869	25,42	sp|P01876|IGHA1_HUMAN	2059,32625	13839,6016	7958,72964	4639,83937	37036,8755	4309,11419	2258,18991	621,824027	11127,241
446	446	NVQDAIADAEQRGEHALK	655,665	25,43	sp|P35908|K22E_HUMAN	56888,1216	56289,8339	43365,2662	29976,0611	29422,2756	2246,43998	1138,60236	1289,11039	4931,29665
447	447	VGGVGVPAAPSITAVTVNK	869	25,44	sp|Q9NSB2|KRT84_HUMAN	1532,70064	573,11048	1562,55768	1716,44915	2541,12469	72419,3294	120,175135	170,738039	5273,63953
448	448	AEQYAVITWR	618,825	25,52	sp|O82803|SRPP_HEVBR	10720,776	1637,08359	8527,00276	2236,0828	9871,2511	10792,4624	4671,38925	3600,57377	10402,7849
449	449	CAM.HGTTHGQTGDTT	635,312	25,54	sp|Q5D862|FILA2_HUMAN	36111,2709	36393,8633	39452,4285	81120,8197	36737,7473	39405,6058	22958,167	47284,1228	33707,7827
450	450	EIETYHNLLEGGQE[KXX]DFESSGAGK	850,033	25,61	sp|P35527|K1C9_HUMAN	16382,6081	47799,1245	39965,6089	14541,4444	21366,1443	972,37618	712,561785	613,630089	673,227936
451	451	EIETYHNLLEGGQE[NaX]DFESSGAGK	844,71	25,62	sp|P35527|K1C9_HUMAN	10821,4862	23905,3818	8675,65706	9707,07258	8344,87024	4672,41871	2352,55797	482,441589	3643,96474
452	452	EIETYHNLLE[KXX]GGQEDFESSGAGK	850,032	25,62	sp|P35527|K1C9_HUMAN	16362,4302	47435,0774	39933,9333	14541,8427	21382,2135	982,81153	705,141093	614,579289	652,289461
453	453	EIETYHN[Dea]LLEGGQ[Dea]EDFESSGAGK	838,051	25,62	sp|P35527|K1C9_HUMAN	79357,2691	303843,585	251238,817	228521,22	150972,007	968,910872	690,506733	745,953458	2961,32674
454	454	YVM[Oxi]GNNPADLLAVDSR	875,929	25,67	sp|Q02413|DSG1_HUMAN	10910,1064	15789,6649	19921,382	12713,0472	13920,3516	6099,69606	606,951558	2138,57736	9926,08357
455	455	SLDGVVPPVIK	562,342	25,68	sp|O82803|SRPP_HEVBR	24273,682	29584,9208	73678,7033	17918,9188	47237,4204	21069,282	15911,8506	29150,7746	6730,13702
456	456	W[2Ox]ELLQQVDTSTR	754,372	25,69	sp|P04264|K2C1_HUMAN	34414,4315	58083,5724	409181,238	13939,542	40598,0713	529078,728	235554,437	48542,7451	8925,56189
457	457	NKLNDLED[KXX]ALQQAK	819,395	25,69	sp|P04264|K2C1_HUMAN	20219,0469	46012,7368	40880,2243	2790,72296	22552,4044	5758,97537	6606,24113	4089,32343	13481,1701
458	458	EIETYHNLLEGGQEDFESSGAGK	837,383	25,73	sp|P35527|K1C9_HUMAN	99183,5426	368309,271	296922,559	734,846047	183244,569	2357,38816	1474,6446	525,729253	41878,726
459	459	NKLNDLEDALQQAK	800,419	25,76	sp|P04264|K2C1_HUMAN	252446,033	642572,787	427694,14	261521,586	286325,72	595,732459	1043,39371	1505,36538	19558,6911
460	460	LNDLEEALQQAK	686,359	25,82	sp|P35908|K22E_HUMAN	21778,5786	21698,4229	31174,9935	21543,7509	16011,1783	3088,80562	2623,15196	2044,09392	9704,48797
461	461	NKLAELEEALQK	693,385	25,85	sp|P13647|K2C5_HUMAN	27644,6914	87352,0212	36101,728	41169,197	35197,9073	2357,18758	4598,93212	1618,49363	7945,70257
462	462	TLNNKFASFIDKVR	551,642	25,89	sp|P48668|K2C6C_HUMAN	20680,8608	21229,4506	8720,13903	886,201873	1471,49632	759,909929	274,466443	382,42534	2551,24743
463	463	TLNNKFASFIDKVR	551,642	25,89	sp|Q9NSB2|KRT84_HUMAN	20680,8608	21229,4506	8720,13903	886,201873	1471,49632	759,909929	274,466443	382,42534	2551,24743
464	464	TLNNKFASFIDKVR	551,642	25,89	sp|P02538|K2C6A_HUMAN	20680,8608	21229,4506	8720,13903	886,201873	1471,49632	759,909929	274,466443	382,42534	2551,24743
465	465	TLNNKFASFIDKVR	551,642	25,89	sp|P13647|K2C5_HUMAN	20680,8608	21229,4506	8720,13903	886,201873	1471,49632	759,909929	274,466443	382,42534	2551,24743
466	466	TLNNKFASFIDKVR	551,642	25,89	sp|P35908|K22E_HUMAN	20680,8608	21229,4506	8720,13903	886,201873	1471,49632	759,909929	274,466443	382,42534	2551,24743
467	467	PGQ.QSLE[NaX]ASLAETEGR	698,322	25,92	sp|Q6IFW6|K1C10_RAT	32148,4872	33625,3418	13572,37	7684,15599	15018,298	7583,98613	1909,24229	1177,65337	10367,7985
468	468	PGQ.QSVEADINGLR	592,799	25,93	sp|P13646|K1C13_HUMAN	46174,2129	48814,3467	45859,0904	29366,8315	35551,0673	1222,73452	793,822954	881,152543	18425,5066
469	469	PGQ.QSVEADINGLR	592,799	25,93	sp|Q6IFW6|K1C10_RAT	46174,2129	48814,3467	45859,0904	29366,8315	35551,0673	1222,73452	793,822954	881,152543	18425,5066
470	470	GAVHDVKDVLDSV	677,353	25,93	sp|P81605|DCD_HUMAN	44790,3362	80745,1462	70052,637	125459,911	74584,4854	3428,18694	3903,37701	710,387365	7788,81272
471	471	PGQ.QSVEADINGLR	592,799	25,93	sp|P13645|K1C10_HUMAN	46174,2129	48814,3467	45859,0904	29366,8315	35551,0673	1222,73452	793,822954	881,152543	18425,5066
472	472	PGQ.QSLEASLAETEGR	687,331	25,96	sp|Q6IFW6|K1C10_RAT	144668,253	189493,26	131308,634	97380,0308	169742,488	4303,1787	2143,82057	2349,9473	207220,594
473	473	TM[Oxi]NNFLDREQYGQYALAVR	769,039	25,96	sp|Q02413|DSG1_HUMAN	9603,70553	5299,18601	7932,92988	7933,42974	10771,1371	6433,99693	1434,77892	475,62787	6221,64511
474	474	PGQ.QSLEASLAETEGR	687,331	25,96	sp|P13645|K1C10_HUMAN	144668,253	189493,26	131308,634	97380,0308	169742,488	4303,1787	2143,82057	2349,9473	207220,594
475	475	TITLEVEPSDTIENVK	894,468	25,97	sp|P68202|RS27A_PLUXY	17489,6583	10030,6245	16404,8011	41325,1971	21926,9215	6780,13735	1149,07498	1560,31228	6423,38096
476	476	SAIVHLINYQDDAELATR	677,354	25,98	sp|Q8WNW3|PLAK_PIG	44672,5366	80719,4314	69904,6049	125345,997	74552,7713	3438,46002	1186,59696	713,109551	8117,5483
477	477	TM[Oxi]NN[Dea]FLDREQYGQYALAVR	769,375	26	sp|Q02413|DSG1_HUMAN	24537,2115	28440,0477	4638,91497	18373,0015	23083,8695	4968,11882	3136,27365	1667,12464	6734,01522
478	478	GLSTESILIPR	395,899	26,01	sp|Q5VSP4|LC1L1_HUMAN	191,090492	2653,64127	1931,9448	388,960653	8705,52704	2446,40472	592,468199	1661,58739	954,983381
479	479	DKSGPLQPGVDIIEGPVK	617,007	26,04	sp|P15252|REF_HEVBR	993,294744	1571,81104	2738,16257	1452,74423	985,743483	16256,9444	20764,1925	9281,73938	1282,80492
480	480	NNLEALEDFEK	661,32	26,06	sp|Q5VSP4|LC1L1_HUMAN	5830,28594	8678,44262	8454,30071	10630,1099	88541,5217	1673,00587	3538,66372	1484,45084	5904,34052
481	481	SC[CAM]AAAGTE[KXX]C[CAM]LISGWGNTK[A	650,279	26,08	sp|P00761|TRYP_PIG	45366,6697	72181,7007	23035,0353	15785,0353	101483,269	4701,2167	3181,24708	3014,23119	8779,82954
482	482	SC[CAM]AAAGTEC[CAM]LISGWGNTK[AAR]	955,939	26,08	sp|P00761|TRYP_PIG	231714,037	659748,156	221634,889	184833,948	776113,765	3719,06798	942,182005	1982,99309	47187,7586
483	483	CAM.VC[CAM]GR[AGA]GGGGSFGYSYGGGSG	1064,47	26,08	sp|P35527|K1C9_HUMAN	3545,39126	14090,3829	11739,3221	8788,91163	8787,53473	2692,60594	1163,99065	780,744106	5012,84543
484	484	Frm.ELLQQVDTSTR	659,335	26,11	sp|P04264|K2C1_HUMAN	47978,9973	142364,943	76530,1362	63145,018	85639,6037	96355,4285	6063,66971	3731,71646	10157,5882
485	485	GTNYLADVFEKK	692,861	26,13	sp|P31151|S10A7_HUMAN	2209,46052	5308,45508	4201,98619	14071,3232	16727,4131	1078,39291	4852,52162	1332,45286	733,145118
486	486	RHPDYSVVLLLR	489,953	26,14	sp|P49065|ALBU_RABIT	21022,2053	2492,03241	3149,16982	17815,3725	17784,3454	2405,39125	715,838522	2389,73171	1134,69281
487	487	RHPDYSVVLLLR	489,953	26,14	sp|P02768|ALBU_HUMAN	21022,2053	2492,03241	3149,16982	17815,3725	17784,3454	2405,39125	715,838522	2389,73171	1134,69281
488	488	CRM.YQ[Dea]ELQITAGR	611,807	26,18	sp|Q7Z794|K2C1B_HUMAN	2841,64848	22013,7696	4971,25186	8264,92251	12550,3619	22157,3871	3017,91554	2751,87929	9826,43278
489	489	NLLFNDNTEC[CAM]LAR	790,38	26,23	sp|P02788|TRFL_HUMAN	3314,85903	733,141027	5219,6589	1484,69924	26483,8227	3321,53946	2879,71514	974,383481	16113,5387
490	490	CAM.SC[CAM]AAAGTEC[CAM]LISGWGNTK[A	984,451	26,24	sp|P00761|TRYP_PIG	5484,7633	14955,2065	4522,48464	5442,00019	19016,1724	2079,62899	6044,62476	2748,59061	14011,4038
491	491	LGEHNIDVLEGNEQ[Dea]FINAAK	738,043	26,29	sp|P00761|TRYP_PIG	75050,3902	109490,455	115323,935	147925,046	134367,166	2302,73655	1926,24682	1136,35534	60805,3799
492	492	GGLVC[CAM]GPEPLVAGSTLSR	885,464	26,32	sp|Q9NSB2|KRT84_HUMAN	813,845067	3377,70798	1326,0161	3098,58479	275,644382	58997,7441	566,853307	2297,64379	3562,85331
493	493	YQGTILSIDDNLQR	818,42	26,34	sp|Q02413|DSG1_HUMAN	13272,4483	18400,8489	14480,909	28051,2053	22857,2162	2668,1309	5146,83194	992,221339	961,151788
494	494	SLPGQNEDLVLTGYQVDK	988,505	26,35	sp|P01040|CYTA_HUMAN	9751,5171	9900,226	6758,65445	19097,0666	18466,4195	1290,35884	2127,81421	1843,25638	17826,5911
495	495	LGEHNIDVLEGNEQFINAAK	737,706	26,41	sp|P00761|TRYP_PIG	66077,7318	91748,41	90475,7151	134191,305	113301,688	2091,17059	1945,64083	769,550819	6083,17811
496	496	STDYGIFQINSR	700,843	26,43	sp|P61628|LYSC_PANTR	21755,3795	15641,1792	32554,9736	17828,4847	134345,523	4294,82385	8577,30929	5904,28223	9388,7473
497	497	W[Oxi]ELLQQMN[Dea]VGTR	746,375	26,44	sp|P35908|K22E_HUMAN	16123,324	19800,5444	28496,8087	11423,0289	15702,5624	1868,51472	2980,60364	3576,08875	6438,21642
498	498	TM[Oxi]IQSPSGVILQEAADVHAR	713,701	26,5	sp|P15924|DESP_HUMAN	3499,19996	15423,937	12482,8798	4960,66091	7429,50079	1377,48321	471,431521	1386,51348	7170,24384
499	499	SC[CAM]AAAGTEC[CAM]LISGWGN[Dea]TK[A	956,432	26,5	sp|P00761|TRYP_PIG	6174,04356	20891,9474	17950,3354	5763,56906	16980,4353	119456,673	3478,24832	175074,04	11696,2002
500	500	NKLNDLEEALQQAK	807,429	26,55	sp|P35908|K22E_HUMAN	133405,693	171757,595	164371,462	96072,142	85430,3686	40330,9603	20651,9475	35228,4445	3823,78173
501	501	RFEPC[CAM]SSSYLPLRPSEGFPNYC[CAM]TPP	779,369	26,6	sp|Q5T749|KPRP_HUMAN	3698,61316	12690,9804	2384,16785	10685,1449	22969,0772	9886,16801	3027,8458	1789,6522	1634,96017
502	502	C[CAM]YTAVVPLVYGGETK	828,917	26,62	sp|P01591|IGJ_HUMAN	3291,43638	2895,21461	2664,32165	380,866047	8050,24321	2530,28051	2789,9219	2254,1834	29560,3327
503	503	ATC[CAM]NNYTPQFQLRPSYSSC[CAM]FPQYR	995,787	26,66	sp|Q5T749|KPRP_HUMAN	3822,82789	14736,3013	3179,80495	7894,29673	27960,8224	916,880267	1982,74654	739,300945	4609,12094
504	504	LGEHNIDVLEGNEQFINAAK[AAR]	1120,07	26,67	sp|P00761|TRYP_PIG	3965,34638	4656,93002	13721,658	12231,6152	6004,28074	1238,60125	429,404108	530,466244	5718,5308
505	505	SQGVFQC[CAM]GPASVIGVR	831,423	26,67	sp|Q08188|TGM3_HUMAN	4647,36674	12016,2764	10970,0803	13883,9001	14304,12	3662,04314	1387,4014	1002,566	7403,16265
506	506	ASKKTEAAAEAAN	631,33	26,76	sp|Q92LB8|RL21_RHIME	1065,77515	2771,95925	709,702791	3251,57453	4103,60133	30362,1489	2380,05544	32608,0729	3104,80972
507	507	VSLDVNHFAPDELTVK	892,463	26,78	sp|P04792|HSPB1_HUMAN	753,186689	3735,5182	6258,59727	5186,66823	315,410822	3200,77184	1792,73574	2722,5154	11130,4244
508	508	TIGIGAPFSK	552,327	26,81	sp|P05089|ARGI1_HUMAN	11763,6031	12401,7	11666,5162	18776,8462	23171,157	1552,76458	2682,28137	301,263038	17885,2118
509	509	LLEAQIATGGIIDPK	769,943	26,83	sp|P15924|DESP_HUMAN	5409,26706	23263,0366	18239,7398	13970,6622	19324,1944	2321,98959	2121,94631	1310,50025	6872,77151
510	510	NVQDAIADAEQRGEHALKDAR	577,542	26,84	sp|P35908|K22E_HUMAN	68010,7935	72013,1787	45378,5548	27450,2081	50002,591	231,603426	781,061779	131,238901	5225,81693
511	511	SGPLQPGVDIIEGPVK	803,446	26,9	sp|P15252|REF_HEVBR	43223,5775	39428,0964	50315,1735	47515,8675	79855,4691	4687,84935	739,577694	1294,16179	15186,8736
512	512	ADEGWYWC[CAM]GVK	685,795	26,95	sp|P01833|PIGR_HUMAN	2360,39707	2022,65078	1014,49713	2006,04981	12092,9291	2263,553	3626,02341	1154,93481	7199,12999
513	513	ADDSLYPILK	567,778	27	RRRRRsp|Q7VN62|UBIB_HAEDU	1847,72978	3970,05706	282,210582	368,072862	1629,23023	4444,76873	820,367734	493,665299	227198,614
514	514	ILNVGDIGGNETVTLR	835,957	27	sp|P22758|TGM1_RABIT	6471,64314	16053,3789	3791,38336	11153,3372	10273,3156	2143,38466	1438,39211	260,683314	3487,39616
515	515	LGEHNIDVLEGNEQF	857,407	27,05	sp|P00761|TRYP_PIG	20307,5884	37977,965	46778,8278	40407,4233	34494,3691	3528,57202	3899,21257	2382,37038	65583,204
516	516	AETEC[CAM]QNTEYQQLLDIK	1041,99	27,07	sp|P13645|K1C10_HUMAN	137788,902	403995,719	267153,082	141410,996	258778,375	3539,78979	1060,98958	762,504341	7607,82318
517	517	EGLYITEEIYK	679,347	27,09	sp|P05089|ARGI1_HUMAN	5813,42523	12048,1375	1675,13179	11670,5665	16190,9287	3316,01238	3555,29367	974,876316	5565,98531
518	518	GSSGGGC[CAM]FGGSSGGYGGLGGFGGGSFR	1171,99	27,13	sp|P13645|K1C10_HUMAN	58408,7433	176677,324	124679,753	71842,9037	118065,751	729,420151	1603,88222	416,821889	8306,17034
519	519	AETEC[CAM]QN[Dea]TEYQQLLDIK	695,328	27,24	sp|P13645|K1C10_HUMAN	87689,8701	192494,37	104563,506	92540,8728	189169,212	3944,33819	513,123818	1689,99446	8789,68416
520	520	GSSGGYGGLGGFGGGSFR	787,343	27,25	sp|P13645|K1C10_HUMAN	11366,6859	25748,5662	22674,7179	16470,8492	20513,2272	13909,9636	1053,63796	3302,69328	3862,30209
521	521	HGVQELEIELQSQLSKK	656,026	27,32	sp|P35527|K1C9_HUMAN	134051,056	530890,117	333924,016	227495,747	258775,218	1494,25058	909,044766	927,37221	5252,35733
522	522	QNQEYQVLLDVR	752,892	27,34	sp|Q15323|K1H1_HUMAN	1370,3255	4373,41157	680,306794	2210,02064	3801,01168	33276,1285	4660,35404	870,710861	5550,79811
523	523	HHLLASDEEIQDVSGTWYLK	586,045	27,4	sp|Q5VSP4|LC1L1_HUMAN	1251,14122	3840,1285	4029,78032	7867,90705	15847,2434	44628,0994	811,886031	909,470541	1722,95933
524	524	CRM.TM[Oxi]QALEIELQSQLSM[Oxi]K	642,319	27,4	sp|Q04695|K1C17_HUMAN	2243,6458	4392,5863	2182,7342	12034,7006	10463,2278	4856,82588	9118,43809	4491,187	6452,82653
525	525	TM[Oxi]QNLEIELQSQLSM[Oxi]K	642,319	27,4	sp|P02533|K1C14_HUMAN	2243,6458	4392,5863	2182,7342	12034,7006	10463,2278	4856,82588	9118,43809	4491,187	6452,82653
526	526	YDENGNPWSAYGGDFGDTPNDR	816,331	27,46	sp|A7ZWZ1|BGAL_ECOHS	9657,16687	6256,23293	2580,39795	1396,51263	4388,16922	3681,74683	2964,14355	2407,33559	14949,1733
527	527	LGEH[AAR]NIDVLEGNEQF	871,421	27,51	sp|P00761|TRYP_PIG	7154,23237	9953,30304	11686,0766	11778,8688	8978,93804	2746,15536	4290,19229	2486,63639	7300,2785
528	528	ENFPNFLSAC[CAM]DK	721,325	27,51	sp|P31151|S10A7_HUMAN	3927,81271	2822,77483	8925,36291	14170,975	10386,0464	21250,488	1832,17558	2873,61559	19246,9181
529	529	LVQNC[CAM]LWTLR	651,853	27,54	sp|Q8WNW3|PLAK_PIG	4433,23205	16981,5941	10206,1174	7886,86169	9502,85059	6213,58432	773,846878	751,777998	3674,89778
530	530	YDENGN[Dea]PWSAYGGDFGDTPNDR	1224,49	27,62	sp|A7ZWZ1|BGAL_ECOHS	2403,74463	3468,03164	2203,33233	406,512244	787,198985	10020,2835	879,06918	1865,62251	3614,59395
531	531	SYLPLLPTEK	580,829	27,63	sp|O82803|SRPP_HEVBR	3958,57613	7689,84279	26515,6912	12110,5671	16576,4161	1170,99702	1253,3245	5157,38229	3525,00254
532	532	LGEHNIDVLEGNEQFIN	970,97	27,67	sp|P00761|TRYP_PIG	38821,6042	69324,0304	81269,4706	75808,6501	61074,9958	799,96434	1430,32466	680,423503	714991,472
533	533	VSVELTNSLFK	618,844	27,74	sp|Q8WNW3|PLAK_PIG	15775,7724	28621,7434	14328,6552	15188,0748	17596,402	3065,75303	5048,60509	3893,38158	9824,50282
534	534	LASYLDKVQALE[NaX]EANNDLENK	800,394	27,81	sp|P35527|K1C9_HUMAN	77674,6663	70438,622	68291,56	55491,1725	65569,7323	4331,60818	5128,20848	2150,20602	16559,736
535	535	IKDFLQGSSC[CAM]IAGIYNETTK	749,045	27,83	sp|P15924|DESP_HUMAN	3470,95215	630,28939	1300,5767	6010,88979	6499,33419	2895,11731	861,264786	1100,58907	9421,15111
536	536	C[CAM]PLLVDSEGWVK	701,855	27,83	sp|P01833|PIGR_HUMAN	3991,5047	3686,32696	3964,39482	2388,92985	8717,89934	2354,0135	1959,98351	2019,70054	8771,31762
537	537	VDTLTGEVNFLK	668,361	27,85	sp|Q7Z794|K2C1B_HUMAN	35492,4681	13404,5178	14976,0792	15068,8704	20823,3942	2006,73375	2303,32128	699,075044	10663,9949
538	538	GGGGSFGYSYGGGSGGGFSASSLGGGFGGGSR	902,391	27,85	sp|P35527|K1C9_HUMAN	84202,6256	216037,672	172249,882	161197,054	157425,587	206582,875	2359,0539	3334,55234	38836,6698
539	539	ILLNPQDKDGSFSVVITGLRK	767,438	27,86	sp|P01833|PIGR_HUMAN	161277,129	190857,185	163055,574	299221,874	183386,906	184330,488	114594,915	184661,555	162508,173
540	540	LALDVEIATYR	632,35	27,89	sp|P48668|K2C6C_HUMAN	179710,079	284720,542	198227,533	212178,435	133290,091	1060,67523	2590,64642	1502,7522	27560,4869
541	541	LALDVEIATYR	632,35	27,89	sp|P02538|K2C6A_HUMAN	179710,079	284720,542	198227,533	212178,435	133290,091	1060,67523	2590,64642	1502,7522	27560,4869
542	542	LALDVEIATYR	632,35	27,89	sp|P13647|K2C5_HUMAN	179710,079	284720,542	198227,533	212178,435	133290,091	1060,67523	2590,64642	1502,7522	27560,4869
543	543	LASYLDKVQALEEANNDLENK	793,067	27,89	sp|P35527|K1C9_HUMAN	85043,4942	358432,276	199791,622	141349,683	174862,473	2778,70115	901,19485	1426,22696	1697,99183
544	544	LALDVEIATYR	632,35	27,89	sp|P35908|K22E_HUMAN	179710,079	284720,542	198227,533	212178,435	133290,091	1060,67523	2590,64642	1502,7522	27560,4869
545	545	SKELTTEIDNNIEQISSYK	738,037	27,89	sp|P13645|K1C10_HUMAN	293077,443	258123,834	182064,683	151270,836	203654,834	1009,555	6154,46164	1314,86493	933,934852
546	546	YLGPQYVAGITNLK	768,927	27,89	sp|P02788|TRFL_HUMAN	1004,71505	2680,10911	1955,63518	677,119602	8972,10505	7031,53181	1195,66312	747,286489	8267,89678
547	547	LALILC[CAM]VTK	515,819	27,97	sp|P31944|CASPE_HUMAN	24143,2882	24720,1868	23995,8243	79093,2594	42494,6968	3902,11935	2100,30696	2381,68111	787,254479
548	548	GGGFGGGSSFGGGSGFSGGGFGGGFGGGR	1200,01	27,97	sp|P35908|K22E_HUMAN	66027,7318	65179,6653	91000,0941	47545,823	53984,3361	1389,19742	2092,8161	271,583718	7949,17387
549	549	LSLDVEIATYR	640,348	27,97	sp|Q8N1N4|K2C78_HUMAN	2982,22001	8030,89532	4477,39322	10960,8802	5089,86975	5801,61084	2455,09588	893,562566	5153,18658
550	550	LSLDVEIATYR	640,348	27,97	sp|Q7Z794|K2C1B_HUMAN	2982,22001	8030,89532	4477,39322	10960,8802	5089,86975	5801,61084	2455,09588	893,562566	5153,18658
551	551	SLNNQFASFIDK	692,348	28,05	sp|P04264|K2C1_HUMAN	1072532,27	1822419,22	1261772,4	723925,859	1120397,07	3613,36239	1956,20398	2810,04802	122460,159
552	552	DAETWFLSK	548,768	28,17	sp|P08779|K1C16_HUMAN	28597,8993	54992,1069	16546,496	11629,0014	6743,62729	2830,52672	2318,00955	304,1265	21994,4933
553	553	SVRPNDEVTAVLAVQTELK	690,381	28,22	sp|P12273|PIP_HUMAN	1923,38454	7181,65892	8709,61731	6848,07727	11196,1499	8486,64722	1643,43112	4114,36809	27289,8911
554	554	SYELPDGQVITIGNER	895,945	28,35	sp|Q5ZMQ2|ACTG_CHICK	7386,65174	7496,96811	4846,14595	8145,90035	13513,0568	3920,70229	2179,67755	487,577521	13401,4491

555	555	CRM.TLNNQFASFIDK	720,859	28,38	sp|Q8N1N4|K2C78_HUMAN	18367,707	18446,3304	24038,8791	13965,4208	15014,028	7940,9459	3100,66289	3973,7492	11111,2672
556	556	LGEHNIDVLEGNEQFINA	1006,48	28,39	sp|P00761|TRYP_PIG	727,009511	9501,21874	2607,94745	10875,5746	8298,98905	1013,36816	7775,51085	3661,45705	96029,7752
557	557	ELGIC[CAM]PDDAAVIPIK	805,924	28,42	sp|P12273|PIP_HUMAN	2908,51947	10024,7617	2892,9781	12941,4612	17914,482	2544,04988	151,05684	2196,67785	9656,50415
558	558	KSPIFGPEEVNSVEGNSVSITC[CAM]YYPPTSVN	1143,22	28,45	sp|P01833|PIGR_HUMAN	244,450723	354,506425	1390,20147	351,084377	5855,3028	1694,08635	457,488235	444,965772	617,662845
559	559	NVSTGDVNVE[CAM]MNAAPGVDLTQLLNNM	982,474	28,46	sp|Q6IFW6|K1C10_RAT	57169,8546	34149,8275	31825,8079	14681,5433	54303,8977	1113,00439	4160,17478	1585,49692	20263,7695
560	560	NVSTGDVNVE[CAM]MNAAPGVDLTQLLNNM	982,474	28,46	sp|P13645|K1C10_HUMAN	57169,8546	34149,8275	31825,8079	14681,5433	54303,8977	1113,00439	4160,17478	1585,49692	20263,7695
561	561	+1R.TVAAPSVFIFPPSDEQLK	701,38	28,55	sp|P01834|IGKC_HUMAN	1976,84212	10325,8084	8393,78512	2482,03595	17719,8959	7333,91202	143,984866	8129,41355	17344,9096
562	562	CRM.FLEQQNQVLQTK	759,899	28,59	sp|Q7Z794|K2C1B_HUMAN	22505,4975	45272,1193	29136,7593	14981,775	30983,1044	4483,44145	302,244278	1651,63335	8717,72386
563	563	CRM.FLEQQNQVLQTK	759,899	28,59	sp|P35908|K22E_HUMAN	22505,4975	45272,1193	29136,7593	14981,775	30983,1044	4483,44145	302,244278	1651,63335	8717,72386
564	564	IITHPNFNGNTLDNDIM[Oxi]LIK	767,063	28,61	sp|P00761|TRYP_PIG	13057,5716	19940,761	18696,3029	1836,05065	1179,54815	1019,88553	901,740606	484,253777	5840,30311
565	565	LGEHNIDVLEGNEQFINAA	1042,01	28,66	sp|P00761|TRYP_PIG	5334,93856	23288,6031	58321,5558	15996,0028	26325,8105	2502,90782	1665,8717	854,862781	205323,297
566	566	SKELTTEIDN[Dea]NIEQISSYK	738,366	28,68	sp|P13645|K1C10_HUMAN	50710,1108	36587,6169	28127,9645	17640,4044	27283,0158	6253,15267	97927,1461	429,639077	33283,1065
567	567	FYTIEILK	513,797	28,71	sp|P12273|PIP_HUMAN	339,063783	29441,2753	625,394463	16486,6417	41525,9984	5527,61368	480,514775	1406,80829	4102,65329
568	568	GTNYLADVFEK	628,81	28,78	sp|P31151|S10A7_HUMAN	6565,03498	5097,77125	7899,87055	21017,6019	13533,389	4975,2775	1432,73591	1428,83942	3801,87305
569	569	NKIIAATIENAQPILQIDNAR	769,434	28,78	sp|P08779|K1C16_HUMAN	10031,3217	48532,0258	17501,477	14883,7581	18272,2958	17681,6788	5657,29896	17712,4517	12249,9666
570	570	YWC[CAM]NDGKTPGAVNAC[CAM]HLSC[CAM	963,442	28,78	sp|P61628|LYSC_PANTR	2586,92653	1238,512	6693,16398	2805,2242	15836,9696	11651,9564	2233,65935	2756,59684	17029,0238
571	571	NKIIAATIEN[Dea]AQPILQIDNAR	769,765	28,81	sp|P08779|K1C16_HUMAN	4485,50531	20426,9691	4270,24291	896,071181	4446,09964	459,746351	2873,86303	150,001058	10029,0006
572	572	QVILQVGVINEAQFSK	887	28,82	sp|Q08554|DSC1_HUMAN	3078,11278	3818,08059	652,134566	5400,63316	4590,04894	531,309851	1603,25519	416,811297	2427,61569
573	573	EEDRIIPGGIYNADLNDEWVQR	868,091	28,85	sp|P01037|CYTN_HUMAN	329,204988	4389,19625	3248,24858	351,180785	2016,30017	3608,30751	1600,36573	508,296802	2594,05769
574	574	NEGTATYAAAVLFR	742,38	28,85	sp|Q8WNW3|PLAK_PIG	5915,31703	16059,4435	9217,71615	3057,73315	11476,3966	5494,84106	1397,37431	3204,38498	2856,33382
575	575	TPGAVNAC[CAM]HLSC[CAM]SALLQDNIADA\	976,465	28,88	sp|P61628|LYSC_PANTR	1209,24411	6733,68586	3118,2142	715,037005	31274,359	2599,60378	1496,86428	1526,68381	6380,97133
576	576	QEPSQGTTTFAVTSILR	918,479	28,94	sp|P01877|IGHA2_HUMAN	3236,28522	11942,7041	3691,56033	1591,18294	100541,569	1285,98032	1099,55276	972,921742	1203,27054
577	577	QEPSQGTTTFAVTSILR	918,479	28,94	sp|P01876|IGHA1_HUMAN	3236,28522	11942,7041	3691,56033	1591,18294	100541,569	1285,98032	1099,55276	972,921742	1203,27054
578	578	HGVQELEIE[NaX]LQSQLSK	620,654	28,95	sp|P35527|K1C9_HUMAN	13155,1775	37153,1073	13998,1734	12868,915	12698,8751	774,937184	589,663197	548,4807	7636,03239
579	579	YSLTYIYTGLSK	704,871	28,97	sp|P25311|ZA2G_HUMAN	24253,4169	12951,7912	14730,7717	21120,6921	37032,7662	2277,80004	3328,32171	1429,63129	9128,63703
580	580	HGVQELEIELQSQLSK	613,327	29,01	sp|P35527|K1C9_HUMAN	205477,181	608576,477	466663,06	386059,352	368460,431	551,231713	5132,59294	971,921176	18754,8673
581	581	VDSLNDEINFLK	703,86	29,04	sp|P19013|K2C4_HUMAN	16867,2627	6189,15941	4329,42388	1742,54689	3081,99234	2511,56339	20154,4191	296,086313	24031,7777
582	582	NRKDIENQYETQITQIEHEVSSSGQEVQSSAK	916,444	29,05	sp|P35527|K1C9_HUMAN	10609,7744	16546,6528	12497,8922	3472,99834	5185,15376	2932,70823	3889,28426	1572,32997	2413,24189
583	583	HGVQELEIELQSQ[Dea]LSK	613,662	29,05	sp|P35527|K1C9_HUMAN	181822,117	557893,357	408310,566	348569,65	316074,506	1303,94306	2789,96767	418,657378	12856,7688
584	584	IIAATIENAQPILQIDNAR	688,719	29,11	sp|P08779|K1C16_HUMAN	40624,6282	80530,2002	24718,4336	17963,8129	10549,3604	2323,79025	1273,07007	654,633786	15225,1817
585	585	LTEEETVC[CAM]LDLDKVEAYR	1092,03	29,15	sp|P15924|DESP_HUMAN	687,01301	6701,31925	2226,45777	410,361675	292,339062	660,863704	2237,83731	202,305912	4943,27823
586	586	YAASSYLSLTPEQWK	872,433	29,3	sp|B9A064|IGLL5_HUMAN	4142,0684	11850,7766	10991,0752	9359,72979	39618,2953	6798,51766	2220,70611	548,588162	6315,60638
587	587	LISWYDNEFGYSNR	882,405	29,31	sp|P04406|G3P_HUMAN	57288,0408	58626,8785	26190,5152	13024,056	20646,89	5494,58095	4942,08914	2153,1414	9022,02214
588	588	ELTTEIDNNIEQISSYK	998,986	29,34	sp|P13645|K1C10_HUMAN	176577,689	170026,509	161997,284	89782,866	107317,259	2761,50995	654,438915	764,290802	17263,6926
589	589	QISNLQQSISDAEQRGENALKDAK	661,589	29,34	sp|P04264|K2C1_HUMAN	153141,104	306700,464	124239,2	2677,95451	2990,80782	511,639734	191,722104	196,436399	1465,80962
590	590	FVDSTVVASVTIIDR	811,441	29,37	sp|P15252|REF_HEVBR	21293,4148	30728,9221	41388,5837	32835,989	59768,5055	1855,68603	346,870193	1140,62007	13003,255
591	591	FASFIDKVQFLEQQNK	648,008	29,41	sp|P19013|K2C4_HUMAN	11717,4732	22723,8057	2012,82589	5460,13739	1121,60534	2660,85438	2610,02822	1431,71018	5992,6399
592	592	RTVQ[Dea]T[Dhy]LEIELQSQLALK	977,06	29,48	sp|Q6IFX1|K1C24_RAT	5430,4561	8524,17587	2897,41938	1308,67348	945,786143	1879,46511	1000,3582	291,051851	1976,55094
593	593	AAS.RTVQ[Dea]GLEIELQSQLALK	977,06	29,48	sp|Q6IFW6|K1C10_RAT	5430,4561	8524,17587	2897,41938	1308,67348	945,786143	1879,46511	1000,3582	291,051851	1976,55094
594	594	IVLDVASSVFN[Dea]TGVQEGAK	968,006	29,53	sp|O82803|SRPP_HEVBR	1306,39028	2394,05788	1771,30157	3517,91649	6639,26711	446,178465	3542,93384	3559,23936	7535,35656
595	595	NADLQVLKPEPELVYEDLR	747,731	29,53	sp|P01833|PIGR_HUMAN	1319,77056	7190,44028	992,385019	2508,42111	26704,4521	1008,75489	204,511812	802,118538	5049,55901
596	596	RISGVGIDQPPYGIFVINQK	734,409	29,57	sp|Q02413|DSG1_HUMAN	1540,89966	1598,20702	2143,46828	17025,2261	2687,89505	1012,31406	3038,05487	257,629679	5190,39448
597	597	NNQFASFIDKVR	719,875	29,63	sp|Q8N1N4|K2C78_HUMAN	925,685683	680,879854	1405,7736	3102,74661	7699,01879	4119,6533	5477,74096	859,984385	7094,56896
598	598	CRM.SC[CAM]AAAGTEC[CAM]LISGWGNTK[A	977,444	29,63	sp|P00761|TRYP_PIG	4664,13916	11708,4678	4663,30067	5074,89859	16559,8862	3148,74818	3703,46663	3210,03642	8033,03962
599	599	LALDIEIATY[CAM]R[AGA]	646,348	29,66	sp|P19013|K2C4_HUMAN	27087,8319	53943,3154	29880,7028	35297,7869	35711,5703	1284,5719	1272,0202	1064,21147	8735,46312
600	600	LALDLEIN[Dea]AYR	646,35	29,71	sp|P21910|LAML2_XENLA	26970,1469	53807,7435	29699,2732	35268,6272	35696,0219	1243,28656	1276,70186	1291,59984	11099,9356
601	601	PGQ.QYTSFHFASLEDVQAK	927,439	29,76	sp|P29508|SPB3_HUMAN	11825,5004	4902,18153	6773,76729	9287,41847	5960,77055	6044,44567	1771,31511	4607,87762	7799,41175
602	602	LVSLTLNLVTR	614,886	29,77	sp|P01833|PIGR_HUMAN	2991,51498	14334,6223	1815,99082	3645,67419	53783,6915	6142,94729	5821,28499	1403,3965	3497,45705
603	603	VNWIQQTIAAN	629,333	29,77	sp|P00761|TRYP_PIG	164610,039	203149,909	296404,999	167447,727	327809,61	293,209375	2874,61989	1175,07552	86956,997
604	604	VLYDAEISQIHQSVTDTNVILSM[Oxi]DNSR	1022,17	29,8	sp|P35908|K22E_HUMAN	37524,35	1273,07857	1078,97724	22823,525	14301,8923	2470,30177	1583,18522	431,934506	2501,42099
605	605	TVQIAAVVDVIR	642,386	29,84	sp|P12273|PIP_HUMAN	31012,081	57894,3701	81824,6125	59408,0341	81319,0199	2247,83259	2653,35057	4706,38162	8607,33989
606	606	KDIENQYETQITQIEHEVSSSGQEVQSSAK	848,909	29,87	sp|P35527|K1C9_HUMAN	7622,25805	27728,8803	22916,5453	4012,0043	17845,5547	6529,81827	4838,41921	456,950682	10463,9719
607	607	SLNNQ[Dea]FASFIDKVR	547,292	29,87	sp|P04264|K2C1_HUMAN	794008,557	1418709,83	589899,283	526048,953	1036923,73	12073,6556	7467,09503	10176,9788	10529,0168
608	608	LGLDIEIATYR	632,351	29,91	sp|O43790|KRT86_HUMAN	20954,9326	32410,3663	39214,5311	29633,0405	17646,0808	36881,2778	26755,5194	13573,6466	39185,9245
609	609	LGLDIEIATYR	632,351	29,91	sp|Q9NSB2|KRT84_HUMAN	20954,9326	32410,3663	39214,5311	29633,0405	17646,0808	36881,2778	26755,5194	13573,6466	39185,9245
610	610	SLNNQFASFIDKVR	546,958	29,96	sp|P04264|K2C1_HUMAN	917037,7	1645562,05	672921,285	2501,41384	1210358,76	3490,83457	1902,53981	3734,46084	14259,4441
611	611	EFNAETFTFHADIC[CAM]	851,369	30,04	sp|P49065|ALBU_RABIT	12719,8376	11125,9473	8625,78726	1193,67236	4541,04698	5738,15973	859,33064	2436,62528	11660,7777
612	612	EFNAETFTFHADIC[CAM]	851,369	30,04	sp|P02768|ALBU_HUMAN	12719,8376	11125,9473	8625,78726	1193,67236	4541,04698	5738,15973	859,33064	2436,62528	11660,7777
613	613	VQALEEANNDLENKIQDWYDK	845,739	30,04	sp|P35527|K1C9_HUMAN	14124,8255	49834,7009	29007,1417	5513,64783	21655,2314	1138,56212	620,438542	715,085215	4875,28177
614	614	DAEDWFFSK	572,751	30,07	sp|Q04695|K1C17_HUMAN	3167,53463	11119,1929	5598,71597	8286,11833	1241,98754	1665,00574	946,611242	273,873448	11998,4382
615	615	DAEEWFFTKTEELNR	957,947	30,1	sp|P02533|K1C14_HUMAN	7508,74791	25841,0724	17330,7142	1154,94589	6391,578	5275,54547	2176,65259	897,269076	7444,55059
616	616	TIEIQENTLNSNLLEIR	1000,54	30,12	sp|Q02413|DSG1_HUMAN	7299,98624	2975,60364	3330,20253	9245,12503	1899,07681	9529,30465	1006,79995	405,847476	3091,6402
617	617	SVNGKEDAIWNLLR	807,934	30,12	sp|P02788|TRFL_HUMAN	3035,49212	6443,78264	2439,98994	811,99633	10051,0197	2649,6866	952,479368	951,58381	7302,38521
618	618	SHFC[CAM]GGSLINSQWVVSAAHC[CAM]Y	794,028	30,12	sp|P00761|TRYP_PIG	1556,4128	2409,21471	1366,54904	476,351007	893,281803	4872,17885	2277,44709	6836,42851	3387,86913
619	619	NKILTATVDNANVLLQIDNAR	766,092	30,13	sp|P02533|K1C14_HUMAN	1638,18509	10774,799	5361,34907	956,238389	1235,68317	239,736274	863,743732	2871,34596	3117,74409
620	620	CRM.TLNNKFASFIDKVR	565,964	30,22	sp|P48668|K2C6C_HUMAN	10893,3751	13813,3771	4136,98814	4231,46325	13642,7156	6302,85054	3668,59414	3975,55158	940,553189
621	621	CRM.TLNNKFASFIDKVR	565,964	30,22	sp|Q9NSB2|KRT84_HUMAN	10893,3751	13813,3771	4136,98814	4231,46325	13642,7156	6302,85054	3668,59414	3975,55158	940,553189
622	622	CRM.TLNNKFASFIDKVR	565,964	30,22	sp|P02538|K2C6A_HUMAN	10893,3751	13813,3771	4136,98814	4231,46325	13642,7156	6302,85054	3668,59414	3975,55158	940,553189
623	623	CRM.TLNNKFASFIDKVR	565,964	30,22	sp|P13647|K2C5_HUMAN	10893,3751	13813,3771	4136,98814	4231,46325	13642,7156	6302,85054	3668,59414	3975,55158	940,553189
624	624	CRM.TLNNKFASFIDKVR	565,964	30,22	sp|P35908|K22E_HUMAN	10893,3751	13813,3771	4136,98814	4231,46325	13642,7156	6302,85054	3668,59414	3975,55158	940,553189
625	625	CRM.TLNNQFASFIDKVR	848,44	30,25	sp|Q8N1N4|K2C78_HUMAN	3154,70502	13731,4785	3163,21625	6356,43039	8663,28131	5053,89816	2433,3951	4790,14147	9941,02505
626	626	FQLFGSPSGQ[Dea]KDLLFK	604,998	30,25	sp|P02788|TRFL_HUMAN	1277,35841	2370,05894	3318,09989	784,082948	8122,31785	9332,03562	4292,78389	1568,71578	4189,90095
627	627	LENLFEALNNK	652,847	30,31	sp|P31944|CASPE_HUMAN	22096,4108	21229,5449	10505,5043	13903,2936	6912,36079	3002,58315	13114,4573	20746,4482	8127,58407
628	628	GHILELLTEVTR	690,895	30,31	sp|P31944|CASPE_HUMAN	2891,10725	2266,36719	5861,90226	17291,846	6844,22585	3042,47437	1850,46488	3642,77868	10583,8801
629	629	PGQ.QLQNIIQATSR	627,869	30,32	sp|P15924|DESP_HUMAN	17364,3566	20934,0722	62176,8699	18388,0894	9910,66346	8999,25011	3726,74747	29172,8159	4125,65674
630	630	DAEEWFFTK	586,767	30,39	sp|P02533|K1C14_HUMAN	461,08678	1404,95023	1208,20132	717,321616	1033,88792	1459,50151	19994,6548	891,344882	8578,67045
631	631	ILLNPQDKDGSFSVVITGLR	724,739	30,42	sp|P01833|PIGR_HUMAN	894,648096	6825,95637	906,041653	2006,38547	29079,4624	809,71879	1644,15487	1206,51058	930,918084
632	632	DLDSIIAEVK	551,804	30,43	sp|P48668|K2C6C_HUMAN	26751,0484	14507,4446	2944,32141	46353,7056	5366,68315	686,103309	6131,75802	5414,20795	7600,52197
633	633	DLDSIIAEVK	551,804	30,43	sp|P02538|K2C6A_HUMAN	26751,0484	14507,4446	2944,32141	46353,7056	5366,68315	686,103309	6131,75802	5414,20795	7600,52197
634	634	DLDSIIAEVK	551,804	30,43	sp|P13647|K2C5_HUMAN	26751,0484	14507,4446	2944,32141	46353,7056	5366,68315	686,103309	6131,75802	5414,20795	7600,52197
635	635	ILTATVD[NaX]NANVLLQIDNAR	692,708	30,46	sp|P02533|K1C14_HUMAN	3945,15615	7012,80131	1082,91379	5461,28477	3242,44698	1739,83705	526,292486	169,139475	9545,80599
636	636	ILTATVDNANVLLQIDNAR	685,379	30,52	sp|P02533|K1C14_HUMAN	55276,67	115836,909	73731,4713	59786,0514	51701,8017	2632,31015	122,317611	40187,6295	6930,76653
637	637	SKELTTEIDNNIEQ[Dea]ISSYKSEITELRR	800,165	30,54	sp|P13645|K1C10_HUMAN	7965,675	24392,1309	95559,5593	6252,76445	31196,6366	158739,277	336,028725	10679,7612	2940,23261
638	638	KSVFEISDEINGLATKAR	660,024	30,6	sp|P21883|ODP2_BACSU	475,097177	1978,1059	2053,8261	1041,99949	232,680257	14107,4194	3367,8352	1655,8613	1767,6992
639	639	ILRGASTMLELSSIETTR	660,024	30,6	sp|O64793|Y1675_ARATH	475,097177	1978,1059	2053,8261	1041,99949	232,680257	14107,4194	3367,8352	1655,8613	1767,6992
640	640	CAM.LALDIEIATYR	667,868	30,6	sp|P19013|K2C4_HUMAN	5257,42483	410,43159	16879,0653	4920,37406	2640,08316	22057,6308	3109,37479	3324,42645	6908,9501
641	641	FLEQQNQVLQTKWELLQQMN[Dea]VGTR	978,177	30,61	sp|P35908|K22E_HUMAN	76077,324	68377,0326	481,544451	25978,4769	63017,9477	4045,40628	235,721814	1669,27091	2914,46636
642	642	TT[Dhy]GIVMDSGDGVTHTVPIYEGYALPHAILR	792,172	30,65	sp|Q5ZMQ2|ACTG_CHICK	425,593398	1562,17156	2712,70306	511,215522	3906,56526	929,197525	1530,1377	537,293376	4725,29854
643	643	QVSLNSGSHFC[CAM]GGSLINSQWVVSAAHC[	1022,47	30,65	sp|P00761|TRYP_PIG	4773,92113	12248,9903	11936,7174	4304,04624	13000,9369	56008,1414	21745,1539	3636,3446	7659,38324
644	644	VFDEFKPLVEEPQNLIK	682,371	30,67	sp|P02768|ALBU_HUMAN	917,257366	5864,06317	3430,93967	12887,6676	12939,1121	16868,8326	4360,33329	7410,08278	7926,03653
645	645	ADTLTDEINFLR	704,358	30,72	sp|P48668|K2C6C_HUMAN	85354,1986	194882,926	96422,0305	51221,4856	37135,8781	4334,47293	4392,60705	2966,65786	23845,4634
646	646	ADTLTDEINFLR	704,358	30,72	sp|P02538|K2C6A_HUMAN	85354,1986	194882,926	96422,0305	51221,4856	37135,8781	4334,47293	4392,60705	2966,65786	23845,4634
647	647	QEIEC[CAM]QNQEYSLLLSIK	1048,02	30,74	sp|P35527|K1C9_HUMAN	16902,2639	101604,754	58093,7672	44727,9885	32447,9454	3419,11728	2581,00191	405,356818	3229,94691
648	648	FLEQQNQVLQTKWELLQQVDTSTR	978,176	30,75	sp|P04264|K2C1_HUMAN	76479,2523	68414,8423	49937,9027	10506,1611	36465,8223	1517,1545	236,50645	382,516298	3742,9091
649	649	NVSTGDVNVEM[2Ox]N[Dea]AAPGVDLTQLLN	974,475	30,76	sp|Q6IFW6|K1C10_RAT	7733,20942	5509,94892	4097,40436	4694,22547	6645,19479	2834,44117	2144,66882	1693,30155	8156,11602
650	650	NVSTGDVNVEM[Oxi]NAAPGVDLTQLLNNM[C	968,799	30,79	sp|Q6IFW6|K1C10_RAT	79164,188	44082,3526	100303,656	49428,0227	84933,9967	3279,38299	1009,21397	284,493604	8970,79415
651	651	NVSTGDVNVEM[Oxi]NAAPGVDLTQLLNNM[C	968,799	30,79	sp|P13645|K1C10_HUMAN	79164,188	44082,3526	100303,656	49428,0227	84933,9967	3279,38299	1009,21397	284,493604	8970,79415
652	652	IVGGYTC[CAM]AANSIPYQVSLNSGSHFC[CAM	987,281	30,81	sp|P00761|TRYP_PIG	624,139817	40164,4704	885,139311	1431,91329	41065,8088	2702,27905	3329,32332	734,448804	2410,3736
653	653	NVSTGDVNVEMNAAPGVD[CAM]LTQLLNNM	977,145	30,88	sp|Q6IFW6|K1C10_RAT	53385,194	112598,665	5863,55644	14139,7773	47145,1656	1457,6469	263,959974	744,190329	6576,10758
654	654	NFPPSQDASGDLYTTSSQLTLPATQC[CAM]LAG	1056,84	30,88	sp|P01876|IGHA1_HUMAN	821,37186	2480,82542	662,147776	362,02594	8773,08121	1404,37333	1478,76461	924,190917	5183,25857
655	655	NVSTGDVNVEMNAAPGVD[CAM]LTQLLNNM	977,145	30,88	sp|P13645|K1C10_HUMAN	53385,194	112598,665	5863,55644	14139,7773	47145,1656	1457,6469	263,959974	744,190329	6576,10758
656	656	IPPPPPAPYGPGIFPPPPPQP	710,72	30,89	sp|P02814|SMR3B_HUMAN	8993,26186	34488,4514	3818,84538	5886,8586	10138,9749	8028,12244	485,678373	602,751672	6625,00217
657	657	YFIALPVPSQPVDPR	849,962	30,93	sp|P17314|IAAC3_WHEAT	1113,982	2402,30958	22340,4683	1662,767	2237,74077	5691,93755	38264,0531	4036,01205	18963,2971
658	658	IVGGYTC[CAM]AANSIPYQVSLNSGSHFC[CAM	1233,59	30,99	sp|P00761|TRYP_PIG	219,130856	5942,48675	967,935812	670,19471	7816,33258	3387,94695	573,938598	1267,77328	12113,0057
659	659	ILTATVD[KXX]NANVLLQIDNAR	698,029	31,03	sp|P02533|K1C14_HUMAN	1229,91141	1828,56839	2932,87259	360,831943	671,11935	11820,8174	1227,35869	577,17716	2238,58972
660	660	VNWIQQ[Dea]TIAAN	629,825	31,04	sp|P00761|TRYP_PIG	25034,8542	36828,663	41312,3875	26410,5979	47030,7304	6564,44357	1043,89729	6186,33263	7492,23374
661	661	NLNDRLASYLDKVRAL	621,015	31,17	sp|Q04695|K1C17_HUMAN	1334,85148	5094,52557	1327,9824	32328,1164	32791,8181	1016,24243	425,850285	485,246537	6206,13374
662	662	NLNDRLASYLDKVRAL	621,015	31,17	sp|P08779|K1C16_HUMAN	1334,85148	5094,52557	1327,9824	32328,1164	32791,8181	1016,24243	425,850285	485,246537	6206,13374
663	663	NLNDRLASYLDKVRAL	621,015	31,17	sp|P02533|K1C14_HUMAN	1334,85148	5094,52557	1327,9824	32328,1164	32791,8181	1016,24243	425,850285	485,246537	6206,13374
664	664	CAM.SDLEMQYETLQEELMALKK	589,791	31,22	sp|P35527|K1C9_HUMAN	4604,55325	18725,7259	834,661936	1153,69244	5258,92988	552,874874	2909,4976	678,451593	13859,4373
665	665	IITHPNFNGNTLDNDIM[Oxi]LIK[AAR]LSSPATL	839,196	31,24	sp|P00761|TRYP_PIG	953,673537	3611,72343	1234,37696	15767,2019	9603,14521	2054,82369	1398,30787	2111,83308	4713,22289

666	666	ELTTEIDNNIEQISSYKSEITELRR	746,132	31,27	sp|P13645|K1C10_HUMAN	3021,17283	2068,08067	3345,59443	734,592811	883,857415	85442,4626	1927,98717	3022,1236	2418,79807
667	667	TVAAPSVFIFPPSDEQLK	973,515	31,37	sp|P01834|IGKC_HUMAN	4383,999	9164,58282	7130,88541	11031,4922	65235,4992	3545,58555	478,974729	1215,95637	10318,574
668	668	APFASIVAGIGGQ	594,324	31,53	sp|P08453|GDB2_WHEAT	65330,5196	20744,0356	62078,8937	69104,0353	17392,4846	41114,6575	12007,3225	11991,9742	6564,92384
669	669	LSGQTIEVTSEYLFR	871,95	31,53	sp|A7ZWZ1|BGAL_ECOHS	1322,84847	2343,31978	1289,80551	443,294302	532,787896	17662,1625	805,823495	1122,05646	11501,0777
670	670	LVINGNPITIFQER	807,452	31,58	sp|P04406|G3P_HUMAN	2209,03447	2691,11829	3189,63152	14464,108	1451,02464	8628,77404	765,374341	1641,49485	10285,2438
671	671	VLQGLEIELQSQLSM[Oxi]K	916,495	31,6	sp|P08779|K1C16_HUMAN	1297,55556	3133,09629	2047,81274	5949,7261	1257,86411	1522,75085	22141,978	1010,71841	4689,30068
672	672	FSLLKPWA	481,278	31,6	sp|Q9GZZ8|LACRT_HUMAN	1543,12648	6654,61722	280,414435	2147,72972	36654,4623	8230,59952	7447,85882	10016,0814	12045,5068
673	673	LEEHLEGIVNIFHQYSVR	728,38	31,7	sp|P80511|S10AC_HUMAN	16957,5866	3061,19123	4765,69629	5718,18203	49819,6988	995,229395	3373,10878	2490,5801	12211,3866
674	674	VLYDAEISQIHQSVTDTNVILSMDNSR	1016,84	31,71	sp|P35908|K22E_HUMAN	793,845669	22497,4692	2192,05056	4951,35929	4493,13199	2589,56098	1716,76335	702,80465	2662,66604
675	675	AVFVDLEPTVIDEVR	851,457	31,74	sp|Q9ZRB7|TBA_WHEAT	2611,86924	1111,19691	5337,77064	11440,0851	7449,99845	6748,1807	2107,73012	3091,95439	10278,6626
676	676	FYTIEILKVE	627,852	31,76	sp|P12273|PIP_HUMAN	33921,3071	35328,9399	28519,6947	310684,386	52960,3717	3418,98423	1774,06937	1517,83616	2447,34721
677	677	ISGVGIDQPPYGIFVINQK	682,371	31,77	sp|Q02413|DSG1_HUMAN	2307,87117	20360,6616	5668,42351	16476,5832	28009,7536	6424,35112	2637,52562	6331,14945	32412,8046
678	678	QQTVGGVNYFFDVEVGR	957,97	31,81	sp|P01037|CYTN_HUMAN	947,481576	8043,77647	3585,18921	1479,36722	2719,87287	804,073683	3714,43249	569,742888	4308,87322
679	679	ILTATVDNANILLQIDNAR	1034,57	31,84	sp|Q04695|K1C17_HUMAN	8089,33081	27784,3223	738,154271	7348,39751	8754,36672	1623,79165	334,476098	1015,07764	1175,12043
680	680	NKLN[Dea]DLEDALQQ[Dea]AKEDLAR	729,383	31,84	sp|P04264|K2C1_HUMAN	1869,79645	571261,07	5017,3988	259674,55	350679,724	21398,5349	3711,88772	29614,8493	9614,12571
681	681	NKLNDLEDALQQ[Dea]AKEDLAR	729,048	31,84	sp|P04264|K2C1_HUMAN	6374,6136	876196,448	2747,80483	395324,643	556713,915	422,853876	1154,14557	239,447703	6002,0037
682	682	NIETIINTFHQYSVK	903,971	32,03	sp|P06702|S10A9_HUMAN	50692,3565	685,901741	30520,7638	1779,39593	1134,41346	3728,90182	1909,76521	428,889516	8252,68003
683	683	TINQSLLQPLNVEIDPEIQK	764,753	32,04	sp|P04264|K2C1_HUMAN	19339,1158	35207,8092	1652,24889	2483,40821	82273,556	1760,53691	988,293213	595,025099	6300,83723
684	684	YVNWIQQTIAAN	710,863	32,2	sp|P00761|TRYP_PIG	27037,5357	25682,2892	49928,0581	68342,154	31787,8989	3381,58305	1436,67816	1834,62461	34998,5476
685	685	PGQ.QISNLQQSISDAEQRGENALK	771,388	32,2	sp|P04264|K2C1_HUMAN	25858,0962	40246,0101	3117,77755	24240,0351	30479,1112	7925,41746	2327,98179	1857,74895	22043,5976
686	686	LNDLEDALQQAKEDLAR	648,001	32,22	sp|P04264|K2C1_HUMAN	53799,2771	97046,5454	70338,5578	1298,53745	1573,55299	56769,4229	607,128385	455,162798	2714,73318
687	687	IDSGLYLGSGYFTAIQNLRK	739,395	32,25	sp|P02788|TRFL_HUMAN	1731,1655	1459,89828	14476,9604	3356,76773	12677,2211	7282,99987	4399,6441	2229,38508	27197,2427
688	688	SLLTPLNLEIDPNAQR	897,489	32,26	sp|Q9NSB2|KRT84_HUMAN	465,86338	5826,93416	1492,86767	3121,61869	8253,58633	68528,6624	2028,71165	1235,39728	3948,74315
689	689	Frm.TLNN[Dea]QFASFIDK	713,85	32,3	sp|Q8N1N4|K2C78_HUMAN	17326,3374	30562,4453	20385,7047	7386,32797	18334,8272	4173,43354	6714,13234	1388,46956	10473,4182
690	690	GAVHDVKDVLDSVL	733,894	32,33	sp|P81605|DCD_HUMAN	5697,23032	4543,05516	6428,82812	4449,72765	3532,18325	49605,6748	1469,31599	38360,1473	10710,1816
691	691	Frm.TLN[Dea]NQFASFIDK	713,85	32,39	sp|Q8N1N4|K2C78_HUMAN	17550,683	30586,6162	20380,09	7327,25934	18403,2584	4175,32038	1856,76037	1396,51745	10401,4425
692	692	FGGFGGPGGVGGLGGPGGFGPGGYPGGIHEV	891,095	32,41	sp|P35908|K22E_HUMAN	1181,25287	5625,93292	1196,95126	11014,7763	13429,3057	2409,15722	1034,55153	543,954078	941,024094
693	693	QLC[CAM]DAELKAK	588,316	32,46	sp|A6ZP10|SEY1_YEAS7	3677,02318	3069,78126	4783,49259	3094,08913	526,756	3733,22958	12180,6283	5083,55353	16219,1698
694	694	NKLN[Dea]DLEDALQQAKEDLAR	729,046	32,46	sp|P04264|K2C1_HUMAN	447004,759	53154,2928	513276,101	9330,35272	27554,1773	530571,131	489,442226	19463,8713	1019,15491
695	695	PGQ.QEPSQGTTTFAVTSILR	909,963	32,47	sp|P01877|IGHA2_HUMAN	1521,92989	9566,08563	2238,36606	2481,12119	59533,3412	6253,6042	2823,49412	738,966678	4493,35752
696	696	PGQ.QEPSQGTTTFAVTSILR	909,963	32,47	sp|P01876|IGHA1_HUMAN	1521,92989	9566,08563	2238,36606	2481,12119	59533,3412	6253,6042	2823,49412	738,966678	4493,35752
697	697	DIENQYETQITQIEHE[KXX]VSSSGQEVQSSAK	661,3	32,5	sp|P35527|K1C9_HUMAN	5567,27106	14839,4083	11735,0485	5279,13097	9106,828	4105,23306	3449,72268	4079,89944	12424,9987
698	698	DIENQYETQITQIEHE[NaX]VSSSGQEVQSSAK	822,38	32,5	sp|P35527|K1C9_HUMAN	6166,2698	11276,492	4238,89564	1721,3789	2589,36638	2242,47809	3321,2949	1389,58557	17160,3891
699	699	DIENQYETQITQIE[NaX]HEVSSSGQEVQSSAK	1096,17	32,5	sp|P35527|K1C9_HUMAN	748,641742	24444,2254	10426,0702	5819,40951	4387,71867	1422,16017	1464,16662	395,971091	605,317198
700	700	DIENQYETQITQIE[KXX]HEVSSSGQEVQSSAK	826,372	32,5	sp|P35527|K1C9_HUMAN	14656,7446	41884,1782	29395,9199	11728,4661	19243,5173	11667,374	2209,65328	2173,74047	11518,8442
701	701	QLC[CAM]SFEIYEVPWEN[Dea]R	985,949	32,63	sp|P01037|CYTN_HUMAN	922,97275	12751,2034	667,387492	714,703855	597,966151	3274,04339	2911,24127	127,392484	6769,80978
702	702	SDLEM[Oxi]QYETLQEELM[Oxi]ALKK	777,709	32,63	sp|P35527|K1C9_HUMAN	27179,7093	56515,5834	66820,3566	50756,0619	46071,2157	13701,1842	652,642632	308,332576	11546,4191
703	703	TKLEEHLEGIVNIFHQYSVR	603,822	32,68	sp|P80511|S10AC_HUMAN	39518,6372	3238,41304	5584,45331	4412,55531	3878,97984	2472,93962	2097,0972	389,436411	3365,76428
704	704	DIENQYETQITQIEHEVSSSGQEVQSSAK	1088,84	32,68	sp|P35527|K1C9_HUMAN	125598,521	480667,326	320201,416	1687,88701	196557,935	397325,753	535,552331	338,96899	5516,73289
705	705	NKLNDLEEALQQAKEDLAR	733,386	32,72	sp|P35908|K22E_HUMAN	177947,894	195746,911	140900,55	22061,7823	8218,7199	8226,95903	4678,99328	5869,89177	8084,53102
706	706	NVSTGDVNVE[CAM]MNAAPGVDLTQLLNNM	977,144	32,73	sp|Q6IFW6|K1C10_RAT	42671,8927	108238,793	553,398544	14428,2235	40840,646	710,672178	1109,71329	411,739156	1925,17246
707	707	NVSTGDVNVE[CAM]MNAAPGVDLTQLLNNM	977,144	32,73	sp|P13645|K1C10_HUMAN	42671,8927	108238,793	553,398544	14428,2235	40840,646	710,672178	1109,71329	411,739156	1925,17246
708	708	PGQ.QISNLQQSISDAEQRGENALKDAK	876,107	32,75	sp|P04264|K2C1_HUMAN	107133,269	290744,439	113440,072	1655,33681	161910,329	152690,561	1606,02166	797,181272	1263,5945
709	709	CRM.FASFIDKVR	563,307	32,77	sp|P48668|K2C6C_HUMAN	3227,38994	2659,11272	5038,13038	8968,35577	8241,65112	4515,29836	11007,8083	16912,3313	8362,70685
710	710	SVVTVIDVFYK	635,358	32,77	sp|Q5D862|FILA2_HUMAN	37520,1688	68255,2812	2009,29765	131547,841	106524,95	6307,83893	2062,40205	6364,12284	7356,1358
711	711	CRM.FASFIDKVR	563,307	32,77	sp|Q8N1N4|K2C78_HUMAN	3227,38994	2659,11272	5038,13038	8968,35577	8241,65112	4515,29836	11007,8083	16912,3313	8362,70685
712	712	CRM.FASFIDKVR	563,307	32,77	sp|Q7Z794|K2C1B_HUMAN	3227,38994	2659,11272	5038,13038	8968,35577	8241,65112	4515,29836	11007,8083	16912,3313	8362,70685
713	713	CRM.FASFIDKVR	563,307	32,77	sp|Q9NSB2|KRT84_HUMAN	3227,38994	2659,11272	5038,13038	8968,35577	8241,65112	4515,29836	11007,8083	16912,3313	8362,70685
714	714	CRM.FASFIDKVR	563,307	32,77	sp|P02538|K2C6A_HUMAN	3227,38994	2659,11272	5038,13038	8968,35577	8241,65112	4515,29836	11007,8083	16912,3313	8362,70685
715	715	CRM.FASFIDKVR	563,307	32,77	sp|P13647|K2C5_HUMAN	3227,38994	2659,11272	5038,13038	8968,35577	8241,65112	4515,29836	11007,8083	16912,3313	8362,70685
716	716	LGEHNIDVLEGNEQFINAAK[Frm]IITHPNFNGN	837,427	32,8	sp|P00761|TRYP_PIG	19186,1349	22151,1141	2723,95242	34805,2152	14131,2865	11224,6205	3059,60413	1917,34795	33272,6119
717	717	FC[CAM]FDLFQEIGKDDR	597,28	32,81	sp|Q96P63|SPB12_HUMAN	4476,53679	5424,92291	3141,27196	12748,0728	15421,152	11364,8255	9018,62023	4274,63605	15051,6776
718	718	YPPGPLAPPQ[Dea]PFGPGFVPPPPPPPYGPGR	983,518	32,83	sp|P02814|SMR3B_HUMAN	1092,58776	8352,36214	1153,9634	1114,90669	810,928472	3221,71782	2791,30584	1047,45807	7171,23087
719	719	LALDIEIATY[Iod]R	702,305	32,86	sp|P19013|K2C4_HUMAN	9739,76569	13636,117	14359,8887	11166,2077	7105,955	3738,38413	3483,53904	6691,82394	21901,9825
720	720	TYLISSIPLQGAFNYK	907,987	32,89	sp|P12273|PIP_HUMAN	8497,52436	33086,8297	17365,8002	18678,702	30016,5103	4776,21484	1641,37714	737,088458	2723,65908
721	721	C[CAM]IESLIAVFQK	654,36	32,91	sp|Q6B345|S10AB_RAT	11399,1632	13773,5948	2041,053	4597,81836	1485,5618	648,862769	2733,16466	2037,39906	21122,7967
722	722	YNTIDDLKDQIVDLTVGNNK	760,058	32,96	sp|P35527|K1C9_HUMAN	4763,87623	15440,7733	12002,4185	8047,27806	8273,8046	1068,92722	4443,12042	1963,07958	498,103259
723	723	QEFLILVIK	551,846	32,98	sp|P05109|S10A8_HUMAN	15197,5774	9472,96075	3484,19618	3786,68883	4706,60237	1068,64835	1334,92103	1868,46369	2074,97562
724	724	GPYPPGPLAPPQPFGPGFVPPPPPPPYGPGR	1034,54	33,01	sp|P02814|SMR3B_HUMAN	10959,0433	2935,49563	7028,5122	1751,60313	6309,47291	7491,71946	1626,8894	434,076399	7362,70792
725	725	GYATTADGYAPEYPLPLIIK	1077,06	33,04	sp|Q08554|DSC1_HUMAN	732,18549	30135,0841	759,12777	25461,013	12724,3913	1481,22904	1600,75402	154,566282	4655,36304
726	726	LALDIEIATYR	639,357	33,04	sp|P19013|K2C4_HUMAN	1289,21728	10550,661	4225,03157	12505,1938	7413,35954	4792,10271	2382,1672	130,039788	17218,2242
727	727	TVVTPVYYIPLEAVK	846,484	33,1	sp|O82803|SRPP_HEVBR	797,8746	2483,47954	2581,54214	918,379763	536,000452	12307,6959	3117,81195	5450,8592	12765,7287
728	728	EVVDPEVFFNATGC[CAM]LR	926,948	33,19	sp|Q13835|PKP1_HUMAN	1996,93729	10639,0586	3645,86121	3347,40511	3802,45416	4158,0706	2087,74992	1091,03275	7633,13877
729	729	LASYLDKVQALEEANNDLENKIQDWYDKKGPA/	824,627	33,19	sp|P35527|K1C9_HUMAN	4511,71241	10877,4251	1315,79527	206,085669	4431,58139	992,153339	1925,42318	170,589579	1154,04374
730	730	VDLLNQEIEFLK	730,903	33,21	sp|P35908|K22E_HUMAN	832189,35	908194,915	574341,993	413787,629	409709,971	7005,10893	8611,85562	137262,106	3775,10862
731	731	LGEHNIDVLEGNEQFINAAK[Frm]IITHPNFNGN	976,982	33,23	sp|P00761|TRYP_PIG	337,760389	5050,65376	3786,84815	6897,05662	12108,9372	7889,11856	1944,0565	437,683244	18561,3949
732	732	EHIMEDLDTNADKQLSFEEFI	842,055	33,32	sp|P06702|S10A9_HUMAN	12742,1735	8230,28248	2330,51026	3318,41904	854,624331	2781,44103	2828,73221	1787,16443	2704,21339
733	733	DIENQYETQITQ[Dea]IEHEVSSSGQEVQSSAK	1089,17	33,32	sp|P35527|K1C9_HUMAN	3036,17041	23500,0738	8284,49333	4816,33884	10361,9466	11897,5919	382144,618	1807,83085	832,713939
734	734	THNLEPYFESFINNLRR	717,364	33,32	sp|P04264|K2C1_HUMAN	424692,647	767587,489	426972,465	15561,2549	375953,836	2540,22419	3191,44612	2288,34834	37446,5976
735	735	TVQTLEIELQSQLALK	907,521	33,49	sp|Q6IFX1|K1C24_RAT	15303,3063	24855,2364	23356,1907	8471,99017	10800,8746	824,757373	1284,808	1385,62295	7523,95296
736	736	TLELQGLINDLQR	756,92	33,49	sp|P15924|DESP_HUMAN	5372,80072	18857,2139	1408,63675	10208,2184	8815,39423	3829,99901	1694,6089	1937,22456	11757,6045
737	737	NVQALEIE[NaX]LQSQLALK	909,997	33,49	sp|P13645|K1C10_HUMAN	65083,2623	79185,41	33270,8714	20443,9725	27460,9288	658,13048	599,354628	1590,89346	5570,67312
738	738	NVQALEIE[KXX]LQSQLALK	917,981	33,49	sp|P13645|K1C10_HUMAN	54779,9196	80242,3891	62141,2441	14269,3402	32752,9193	3815,992	1604,32284	628,444809	4060,51503
739	739	LASYLDKVQALEEANNDLENKIQDWYDKK	864,186	33,53	sp|P35527|K1C9_HUMAN	342,120437	35682,1105	24677,0381	14860,6692	8365,63455	1616,86313	1658,11566	1190,47792	2716,64912
740	740	EIPAWVPFDPAAQITK	891,973	33,56	sp|P25311|ZA2G_HUMAN	20471,8632	14557,016	1489,79868	20829,771	34023,5593	1648,48022	1635,76257	645,293208	4739,05775
741	741	RHPYFYAPELLFFAK	633,669	33,57	sp|P02768|ALBU_HUMAN	13723,6037	624,918459	408,352037	341,507566	1712,78983	596,302539	977,613843	136,308925	9178,01433
742	742	NVQALEIELQSQLALK	899,008	33,6	sp|P13645|K1C10_HUMAN	548102,393	708865,127	499630,918	317677,821	400501,85	1004,53473	1164,26674	708,766158	12927,4019
743	743	CRM.TVQ[Dea]GLEIELQSQLALK	907,519	33,62	sp|Q6IFW6|K1C10_RAT	15314,1599	24887,3296	23353,8472	980,104872	10880,6727	1458,91372	1323,43673	1525,82657	6099,03336
744	744	RDPTAEQFQEELEKFQQAIDSR	667,079	33,62	sp|P31944|CASPE_HUMAN	1512,83614	2325,59313	1294,3249	5543,53183	3315,1157	764,563813	2640,42128	1546,89003	1104,43172
745	745	DIENQ[Dea]YETQITQIEHEVSSSGQEVQSSAK	1089,17	33,63	sp|P35527|K1C9_HUMAN	1074,67228	23461,0661	574,58599	559,309511	955,095118	20490,1919	6078,9058	1774,04222	811,223447
746	746	IVGGYTC[CAM]AANSIPYQVSLNSGSHFC[CAM	1133,78	33,68	sp|P00761|TRYP_PIG	367,533052	1842,29485	1015,16137	709,916993	4040,3298	1980,08741	1491,68567	518,09345	950,906199
747	747	Frm.TLN[Dea]NQFASFIDKVR	841,434	33,72	sp|Q8N1N4|K2C78_HUMAN	20068,8191	37186,3088	1964,56876	11294,8837	25154,0043	5276,66572	4168,14555	1362,4004	8537,33128
748	748	GVDEVTIVNILTNR	771,927	33,73	sp|Q6TEQ7|ANXA2_CANLF	10286,8151	1277,60798	7835,01537	1498,44784	12919,8136	4908,58281	1342,01184	719,006431	10132,1624
749	749	NLDLD[NaX]SIIAEVK	676,356	33,83	sp|P48668|K2C6C_HUMAN	64351,3978	82476,4033	5042,99401	15526,7575	23754,1425	2826,9013	6510,00535	1386,2793	45214,5056
750	750	NLDLD[NaX]SIIAEVK	676,356	33,83	sp|P02538|K2C6A_HUMAN	64351,3978	82476,4033	5042,99401	15526,7575	23754,1425	2826,9013	6510,00535	1386,2793	45214,5056
751	751	NLDLD[NaX]SIIAEVK	676,356	33,83	sp|P13647|K2C5_HUMAN	64351,3978	82476,4033	5042,99401	15526,7575	23754,1425	2826,9013	6510,00535	1386,2793	45214,5056
752	752	NLDLD[NaX]SIIAEVK	676,356	33,83	sp|P35908|K22E_HUMAN	64351,3978	82476,4033	5042,99401	15526,7575	23754,1425	2826,9013	6510,00535	1386,2793	45214,5056
753	753	TK[AAR]VC[CAM]NYVNWIQQTIAAN	1026,02	33,85	sp|P00761|TRYP_PIG	92425,9604	180027,059	150063,305	107544,799	209256,047	3677,45717	2378,95392	455,510083	38516,7444
754	754	SLDLD[NaX]SIIAEVK	662,852	33,88	sp|P04264|K2C1_HUMAN	103269,717	197110,338	58968,9724	34294,2493	62834,3513	2743,57446	5781,45329	2126,04641	13569,9351
755	755	YLGYLEQLLR	634,383	33,89	sp|P02662|CASA1_BOVIN	185695,024	230523,193	160536,973	189157,962	148187,854	168863,31	74579,6579	132883,62	72669,6472
756	756	DLNLDGIIAEVK	650,36	33,9	sp|Q9NSB2|KRT84_HUMAN	5338,96828	2912,21241	4600,09157	7067,43196	2672,94626	65894,5985	4403,03204	2924,39477	14590,9351
757	757	LDLDSIIAEVK	608,343	33,9	sp|P48668|K2C6C_HUMAN	13212,9204	5970,26727	15390,9278	5536,12282	11783,2048	5078,96987	5759,75691	3409,25102	35409,4979
758	758	LDLDSIIAEVK	608,343	33,9	sp|P02538|K2C6A_HUMAN	13212,9204	5970,26727	15390,9278	5536,12282	11783,2048	5078,96987	5759,75691	3409,25102	35409,4979
759	759	LDLDSIIAEVK	608,343	33,9	sp|P13647|K2C5_HUMAN	13212,9204	5970,26727	15390,9278	5536,12282	11783,2048	5078,96987	5759,75691	3409,25102	35409,4979
760	760	NLDLDSIIAEVK	665,367	33,93	sp|P48668|K2C6C_HUMAN	1502427,48	1747765,81	904292,575	595676,532	702726,148	12151,4207	6624,90325	19553,1395	53810,6516
761	761	NLDLDSIIAEVK	665,367	33,93	sp|P02538|K2C6A_HUMAN	1502427,48	1747765,81	904292,575	595676,532	702726,148	12151,4207	6624,90325	19553,1395	53810,6516
762	762	NLDLDSIIAEVK	665,367	33,93	sp|P13647|K2C5_HUMAN	1502427,48	1747765,81	904292,575	595676,532	702726,148	12151,4207	6624,90325	19553,1395	53810,6516
763	763	NLDLDSIIAEVK	665,367	33,93	sp|P35908|K22E_HUMAN	1502427,48	1747765,81	904292,575	595676,532	702726,148	12151,4207	6624,90325	19553,1395	53810,6516
764	764	TLQ[Dea]GLQFLHVVTR	756,924	34	sp|Q2KZ15|DXS_BORA1	1675,56687	2885,32954	2677,68701	2297,37617	389,03739	11429,6344	1727,00399	1528,3896	4210,96589
765	765	EVTPFFIIYC[CAM]R	722,868	34,02	sp|Q02413|DSG1_HUMAN	1369,51535	18664,4246	7012,8392	23131,2494	26961,8587	4119,15955	1153,03635	1829,4403	6977,58936
766	766	EVQGFESATFLGYFK	861,925	34,09	sp|P20305|GELS_PIG	6190,99415	1195,76706	5176,32658	1939,24869	461,33717	5424,58396	490,660486	775,785641	6476,44522
767	767	Frm.TLNN[Dea]QFASFIDKVR	841,435	34,2	sp|Q8N1N4|K2C78_HUMAN	1327,82476	3041,32602	17725,2974	2044,51047	2642,28728	28154,5904	1244,42397	2809,9468	23612,1113
768	768	VC[CAM]NYVNW[Kyn]IQQTIAAN	899,43	34,2	sp|P00761|TRYP_PIG	10536,0924	25817,895	11292,3079	14441,4113	16026,1796	4721,28313	13470,8291	7617,5157	32111,8196
769	769	PGQ.QEIEC[CAM]QNQEYSLLLSIK	1039,51	34,2	sp|P35527|K1C9_HUMAN	8039,04741	60692,8966	35423,0607	27681,7034	20216,1923	8522,15436	1425,38568	1280,94835	7542,85937
770	770	LALDIEIATY	561,308	34,24	sp|P19013|K2C4_HUMAN	19349,0136	36163,2446	10931,1151	11797,3431	23778,9789	25947,7427	5485,38614	1088,47052	71130,6068
771	771	LDN[Dea]LTTSPILSR	665,868	34,26	sp|F1MDL2|INTU_BOVIN	35515,9371	51627,6801	45930,3905	46582,6894	27474,426	1046832,22	448949,111	149979,036	8903,72582
772	772	NLDLLQ[Dea]KEFPL	665,868	34,26	sp|Q9FMA3|PEX5_ARATH	35515,9371	51627,6801	45930,3905	46582,6894	27474,426	1046832,22	448949,111	149979,036	8903,72582
773	773	ARFKAANM[Oxi]EAVK	676,357	34,26	sp|A1A316|RPOC_BIFAA	4910,19731	2988,62827	33649,2717	1639,34916	5406,07742	55407,8791	32722,3353	9279,70596	13212,481
774	774	NLDLD[KXX]SIIAEVK	456,563	34,26	sp|P48668|K2C6C_HUMAN	1201,13531	1379,17168	2316,02327	3365,08721	1017,45801	44085,4129	794,861259	2888,84179	1190,2013
775	775	N[Dea]LDLDSIIAEVK	665,868	34,26	sp|P48668|K2C6C_HUMAN	35515,9371	51627,6801	45930,3905	46582,6894	27474,426	1046832,22	448949,111	149979,036	8903,72582
776	776	NLDLD[KXX]SIIAEVK	456,563	34,26	sp|P02538|K2C6A_HUMAN	1201,13531	1379,17168	2316,02327	3365,08721	1017,45801	44085,4129	794,861259	2888,84179	1190,2013

777	777	N[Dea]LDLDSIIAEVK	665,868	34,26	sp|P02538|K2C6A_HUMAN	35515,9371	51627,6801	45930,3905	46582,6894	27474,426	1046832,22	448949,111	149979,036	8903,72582
778	778	NLDLD[KXX]SIIAEVK	456,563	34,26	sp|P13647|K2C5_HUMAN	1201,13531	1379,17168	2316,02327	3365,08721	1017,45801	44085,4129	794,861259	2888,84179	1190,2013
779	779	N[Dea]LDLDSIIAEVK	665,868	34,26	sp|P13647|K2C5_HUMAN	35515,9371	51627,6801	45930,3905	46582,6894	27474,426	1046832,22	448949,111	149979,036	8903,72582
780	780	N[Dea]LDLDSIIAEVK	665,868	34,26	sp|P35908|K22E_HUMAN	35515,9371	51627,6801	45930,3905	46582,6894	27474,426	1046832,22	448949,111	149979,036	8903,72582
781	781	FGGFGGPGGVGGLGGPGGFGPGGYPGGIHEVS	1024,03	34,32	sp|P35908|K22E_HUMAN	172739,946	119943,579	98328,4834	67456,8462	96709,7111	31761,8833	1540,9076	1489,16282	22117,5205
782	782	FGGFGGPGGVGGLGGPGGFGPGGYPGGIHEVS	1023,78	34,32	sp|P35908|K22E_HUMAN	68714,7814	54960,741	42188,2017	29188,855	43737,8547	2419,30501	1149,12276	968,865371	2462,46957
783	783	NQILNLTTD[NaX]NANILLQIDNAR	797,087	34,37	sp|P13645|K1C10_HUMAN	25914,0604	26940,1124	8316,78412	6986,00727	11555,0233	902,797746	1121,90094	678,010179	1943,50623
784	784	Frm.TVQGLEIELQSQLALK	600,011	34,37	sp|Q6IFW6|K1C10_RAT	14027,658	6648,09473	23299,8715	8562,97038	6219,89813	12722,9493	161864,284	5333,76233	1736,86862
785	785	TYLFLQEYLDAIKK	872,982	34,37	sp|P29508|SPB3_HUMAN	514,316633	1292,61622	534,013718	7803,80003	2161,4605	3184,43252	2712,55533	1081,56367	3402,00005
786	786	CAM.NLDLDSIIAEVK	693,874	34,38	sp|P48668|K2C6C_HUMAN	17142,991	15264,4314	14914,5368	137,925059	5800,75883	9344,24666	1434,29502	2745,33476	10384,7592
787	787	CAM.NLDLDSIIAEVK	693,874	34,38	sp|P02538|K2C6A_HUMAN	17142,991	15264,4314	14914,5368	137,925059	5800,75883	9344,24666	1434,29502	2745,33476	10384,7592
788	788	CAM.NLDLDSIIAEVK	693,874	34,38	sp|P13647|K2C5_HUMAN	17142,991	15264,4314	14914,5368	137,925059	5800,75883	9344,24666	1434,29502	2745,33476	10384,7592
789	789	YLDFSSIITEVR	721,882	34,38	sp|Q8N1N4|K2C78_HUMAN	16464,5879	17646,7609	1559,30609	10390,9361	11894,619	12135,054	846,618679	1192,18967	13543,0631
790	790	NQILNLTTDNANILLQIDNAR	789,76	34,45	sp|P13645|K1C10_HUMAN	309726,881	306641,527	216914,637	150579,211	230716,292	1353,3849	1314,06151	892,88934	17921,3242
791	791	LAGGEKATMQNLNDRLASY	1026,52	34,46	sp|Q04695|K1C17_HUMAN	26588,0145	42134,6534	4997,24556	38240,7631	55771,4233	96400,4856	1509,15099	130278,104	7444,91635
792	792	FVTHVSDWGALATISTLEAVR	758,402	34,5	sp|O75629|CREG1_HUMAN	3048,96218	3539,95907	6756,48996	9862,18197	16798,2019	6760,80483	7637,09362	9664,50032	7952,3978
793	793	AAS.TVQ[Dea]GLEIELQSQLALK	899,007	34,63	sp|Q6IFW6|K1C10_RAT	7131,10897	13898,6737	3418,83279	1203,00132	3179,0402	11965,8295	338920,539	1172,61354	5214,31316
794	794	KDSPQTIPTYTDALHVYSTVEGYIAYR	772,888	34,65	sp|P31944|CASPE_HUMAN	1624,07568	1153,84165	727,134839	8190,65733	5381,65991	2814,69596	1592,24286	1622,70554	5190,087
795	795	GFGGASGGGYSSSGGFGGGFGGGSGGGFGGG	1225,77	34,66	sp|P35527|K1C9_HUMAN	1035,96546	16615,1008	12393,8412	2482,53827	2045,67076	1293,02262	397,029477	189,730615	1175,83431
796	796	GFGGASGGGYSSSGGFGGGFGGGSGGGFGGG	1216,28	34,68	sp|P35527|K1C9_HUMAN	4503,28905	17727,2217	26693,7407	22357,7799	25950,4155	873,606031	1385,10191	198,500342	5855,01798
797	797	LVDQNIFSFYLSR	801,419	34,8	sp|P07339|CATD_HUMAN	2470,56374	1259,99182	3126,89543	21757,8814	15774,2158	9331,57964	3457,34259	1885,68743	23652,2362
798	798	THN[Dea]LEPYFESFINN[Dea]LR	998,493	34,86	sp|P04264|K2C1_HUMAN	118309,888	322270,319	2195,6344	94584,1515	130625,178	3781,41188	2907,23157	1435,47206	13548,6058
799	799	THNLE[KXX]PYFESFINNLR	677,979	34,86	sp|P04264|K2C1_HUMAN	56523,9019	90531,6038	2337,85007	1664,50113	44418,7597	921,002336	1297,3968	1238,81257	2905,55619
800	800	THNLEPYFESFIN[Dea]NLR	665,665	34,86	sp|P04264|K2C1_HUMAN	677988,942	1312911,78	1074,05777	568186,3	832301,992	494,168529	2019,45832	250,075939	11042,0403
801	801	ADLEMQIENLKEELAYLKK	761,06	34,93	sp|Q04695|K1C17_HUMAN	1267,20688	187,83152	2012,76022	140639,862	272714,229	3196,37964	2793,81418	899,603337	10525,259
802	802	NIETIINTFHQYSV	839,92	34,96	sp|P06702|S10A9_HUMAN	19549,9389	9851,9455	1555,91465	455,923104	7549,29242	6377,54339	376,194519	815,849835	2101,1205
803	803	SLDLDSIIDAVR	658,856	35,01	sp|Q7Z794|K2C1B_HUMAN	49017,4262	8990,52324	17791,4334	13052,096	596,769934	13721,1565	3307,60268	1762,58927	1380,88179
804	804	SFGGYLYYPGSYPSSLVYSTALC[CAM]SPSTC[CA	1161,21	35,02	sp|Q3LI77|KR134_HUMAN	505,333022	1496,56667	868,592906	1256,70732	451,36686	3910,26726	294,782748	257,172196	387,915894
805	805	ASAISVTVLNVIEGPVFRPGSK	747,758	35,02	sp|Q02413|DSG1_HUMAN	11345,0554	14471,4444	1357,4768	2797,06844	11349,4542	1564,61157	1847,49103	2586,27239	1036,9646
806	806	SGTASVVC[CAM]LLNNFYPR	899,451	35,04	sp|P01834|IGKC_HUMAN	6567,45398	24099,792	10215,9224	20581,2932	86653,8608	10203,8716	7352,90032	6371,93735	7677,75014
807	807	IDSGLYLGSGYFTAIQNLR	1044,54	35,04	sp|P02788|TRFL_HUMAN	879,264031	2689,68692	1662,53201	2959,34041	18443,5824	1276,0035	1229,14239	528,694945	1148,15882
808	808	NNKFASFIDKVRFL	566,972	35,07	sp|P48668|K2C6C_HUMAN	3566,04972	1983,59509	504,002003	10409,4848	15402,5399	368,070068	353,797737	556,589115	1142,08777
809	809	NNQFASFIDKVRFL	566,972	35,07	sp|Q8N1N4|K2C78_HUMAN	3566,04972	1983,59509	504,002003	10409,4848	15402,5399	368,070068	353,797737	556,589115	1142,08777
810	810	NNKFASFIDKVRFL	566,972	35,07	sp|Q7Z794|K2C1B_HUMAN	3566,04972	1983,59509	504,002003	10409,4848	15402,5399	368,070068	353,797737	556,589115	1142,08777
811	811	NNKFASFIDKVRFL	566,972	35,07	sp|Q9NSB2|KRT84_HUMAN	3566,04972	1983,59509	504,002003	10409,4848	15402,5399	368,070068	353,797737	556,589115	1142,08777
812	812	NNKFASFIDKVRFL	566,972	35,07	sp|P02538|K2C6A_HUMAN	3566,04972	1983,59509	504,002003	10409,4848	15402,5399	368,070068	353,797737	556,589115	1142,08777
813	813	NNKFASFIDKVRFL	566,972	35,07	sp|P13647|K2C5_HUMAN	3566,04972	1983,59509	504,002003	10409,4848	15402,5399	368,070068	353,797737	556,589115	1142,08777
814	814	ADLEM[DTM]QIESLTEELAYLKK	726,055	35,12	sp|Q6IFW6|K1C10_RAT	2829,5201	2762,39963	1052,55222	1672,16284	1644,33027	1433,25547	907,808189	1345,12686	1543,52697
815	815	CAM.ADLEMQIESLTEELAYLKK	761,059	35,15	sp|Q6IFW6|K1C10_RAT	362701,067	505260,353	1098,62658	140553,438	272696,768	558,080713	2750,21165	890,809885	9437,95651
816	816	CAM.ADLEMQIESLTEELAYLKK	761,059	35,15	sp|P13645|K1C10_HUMAN	362701,067	505260,353	1098,62658	140553,438	272696,768	558,080713	2750,21165	890,809885	9437,95651
817	817	NVSTGDVNVEM[Oxi]NAAPGVDLTQLLNNMR	963,467	35,21	sp|Q6IFW6|K1C10_RAT	2126,69123	11644,8351	3358,90291	3630,50871	7259,95675	1249,01367	1585,87714	2949,68028	5820,33541
818	818	DAFLGSFLYEYSR	784,373	35,22	sp|P02769|ALBU_BOVIN	28058,8519	28872,7818	3098,26584	23585,7863	20604,3433	3515,25627	1562,74522	1301,79361	10858,554
819	819	VIEHIMEDLDTNADKQLSFEEFI	912,774	35,29	sp|P06702|S10A9_HUMAN	53303,6096	23845,5351	861,374394	18448,9707	476,167966	2820,10896	927,131223	849,397446	2627,95318
820	820	LC[CAM]YVALDFEQEMATAASSSSLEK	850,733	35,47	sp|Q5ZMQ2|ACTG_CHICK	10933,9692	14385,9573	9841,61341	679,383883	814,915426	4045,7323	1249,97829	226,416612	6792,03583
821	821	LGEHNIDVLEGNEQFINAAKIITHPNFNGNTLDN	803,136	35,48	sp|P00761|TRYP_PIG	320235,732	13699,2919	256100,535	11132,6258	11506,0379	7864,90756	2625,84394	11304,0278	47556,7077
822	822	THN[Dea]LEPYFESFINNLR	665,664	35,49	sp|P04264|K2C1_HUMAN	3275,53047	1312865,12	744642,691	3623,92754	3567,66951	1044506,75	900,342001	83211,205	3190,5677
823	823	ADLEMQIE[CAM]SLTEELAYLKK	761,059	35,51	sp|Q6IFW6|K1C10_RAT	362736,351	505265,129	272080,249	7247,1999	10806,8167	369987,304	3082,90016	3416,9707	4722,61554
824	824	SLFTDVVAEKDLFHC[CAM]VSFTLPR	861,11	35,56	sp|Q7SIH1|A2MG_BOVIN	15706,1249	10524,8156	6557,32022	520,664241	1074,6858	1885,30962	2562,88217	3236,76329	3821,71512
825	825	ADLE[CAM]MQIESLTEELAYLKK	761,06	35,75	sp|Q6IFW6|K1C10_RAT	361012,091	36946,4599	268042,519	6913,6522	10806,8345	369964,341	2543,0304	50232,4122	1116,34866
826	826	DAGFYWC[CAM]LTNGDTLWR	987,942	35,9	sp|P01833|PIGR_HUMAN	184,923096	721,630646	1219,40815	593,739117	9078,42886	1270,51909	873,456072	234,867625	490,903161
827	827	FFVAPFPEVFGK	692,867	35,92	sp|P02662|CASA1_BOVIN	683,877938	1686,96207	26890,881	180,604896	15422,3535	9943,60702	369,096117	493,569597	704,43939
828	828	SNLEPLFESYITNLR	898,458	36,25	sp|Q9NSB2|KRT84_HUMAN	14347,0372	29288,0367	4340,89787	21548,9296	24834,6792	14950,2277	491,628618	967,900591	8980,6741
829	829	YC[CAM]GQLQMIQE[CAM]Q[Dea]ISNLEAQIT	932,454	36,3	sp|P35527|K1C9_HUMAN	23114,1312	72979,374	1846,92569	19122,3688	19279,4962	2336,22486	1157,37011	451,753669	8530,17849
830	830	YC[CAM]GQLQMIQE[CAM]QISNLEAQITDVR	932,122	36,3	sp|P35527|K1C9_HUMAN	13686,011	46323,7145	2473,92775	7333,88208	11923,4386	793,814905	747,661511	478,96314	3355,95491
831	831	QNLEPLFEQYINNLR	630,996	36,36	sp|P48668|K2C6C_HUMAN	13549,4577	16961,2613	3020,57594	2223,03215	237,131712	680,711228	212,471904	4967,22255	702,037811
832	832	QNLEPLFEQYINNLR	630,996	36,36	sp|P02538|K2C6A_HUMAN	13549,4577	16961,2613	3020,57594	2223,03215	237,131712	680,711228	212,471904	4967,22255	702,037811
833	833	QNLEPLFEQYINNLR	630,996	36,36	sp|P13647|K2C5_HUMAN	13549,4577	16961,2613	3020,57594	2223,03215	237,131712	680,711228	212,471904	4967,22255	702,037811
834	834	VC[CAM]NYVNWIQQTIAAN	897,435	36,36	sp|P00761|TRYP_PIG	21810,3236	42074,7557	6358,46557	31530,9134	38545,5547	5360,88431	4442,58337	21625,8429	11710,8375
835	835	TAFQEALDAAGDKLVVVDFSATWC[CAM]GPC[	1019,49	36,39	sp|Q5R9M3|THIO_PONAB	8250,36343	5170,09459	337,034751	5001,54926	2398,65828	4702,44761	591,768051	2636,69481	8048,93681
836	836	TVNTAVAITLAC[CAM]FGLAR	593,339	36,59	sp|P05089|ARGI1_HUMAN	14140,591	19694,861	8502,75174	26483,3364	13956,1312	9098,65749	3705,04846	13166,0299	8654,39787
837	837	DIILDNPTLTLEVLNEAR	1020,05	36,7	sp|Q08188|TGM3_HUMAN	10180,8314	7472,95641	1583,33173	9933,66031	10171,3965	1445,40097	138,862313	4032,24346	195,637763
838	838	GLVLIAFSQYLQQC[CAM]PFDEHVK	831,426	36,73	sp|P02769|ALBU_BOVIN	34865,8806	138,709345	1958,25565	26501,4223	15561,1073	1820,92466	3231,40585	2069,83718	10593,4847
839	839	ILVNQLSVDDVNVLTC[CAM]ATGTLSNLTC[CAI	1088,55	36,82	sp|Q8WNW3|PLAK_PIG	1933,56054	5238,12132	3735,18683	4354,73122	2102,46929	3884,53997	1255,23618	140,406013	1214,1059
840	840	NLEPLFETYLSVLRK	608,017	36,84	sp|P19013|K2C4_HUMAN	17738,8514	12357,9802	5185,66053	17612,5575	1314,16702	967,775928	798,838361	574,847325	300,82338
841	841	NYSPYYNTIDDLKDQIVDLTVGNNK	726,358	36,85	sp|P35527|K1C9_HUMAN	33544,2346	120298,79	2155,46327	62815,0666	60343,5891	104835,437	789,887719	4661,41924	30311,6379
842	842	NYSPYYNTIDD[KXX]LKDQIVDLTVGNNK	735,845	36,88	sp|P35527|K1C9_HUMAN	8808,01594	34295,2263	13673,6346	2479,62105	12619,4225	21817,1666	499,423895	448,865552	4225,43248
843	843	LGEHNIDVLEGNEQFINAAKIITHPNFNGNTLDN	797,275	36,92	sp|P00761|TRYP_PIG	9565,94164	3599,58748	12642,4906	5004,41638	14642,5099	5527,09059	2622,97931	3093,49174	1401,55143
844	844	DVFLGM[Oxi]FLYEYAR	820,397	37,01	sp|P02768|ALBU_HUMAN	20071,3389	17428,5157	1701,56792	22345,2593	12420,8832	1096,31373	876,437249	5107,71717	3549,36108
845	845	CRM.NLDLDSIIAEVK	686,868	37,08	sp|P48668|K2C6C_HUMAN	9799,75667	12628,1475	4650,44529	2901,89796	6508,93987	7846,58802	2044,22461	203,224785	5013,44339
846	846	CRM.NLDLDSIIAEVK	686,868	37,08	sp|P02538|K2C6A_HUMAN	9799,75667	12628,1475	4650,44529	2901,89796	6508,93987	7846,58802	2044,22461	203,224785	5013,44339
847	847	CRM.NLDLDSIIAEVK	686,868	37,08	sp|P13647|K2C5_HUMAN	9799,75667	12628,1475	4650,44529	2901,89796	6508,93987	7846,58802	2044,22461	203,224785	5013,44339
848	848	CRM.NLDLDSIIAEVK	686,868	37,08	sp|P35908|K22E_HUMAN	9799,75667	12628,1475	4650,44529	2901,89796	6508,93987	7846,58802	2044,22461	203,224785	5013,44339
849	849	DSPQTIPTYTDALHVYSTVEGYIAYR	987,484	37,09	sp|P31944|CASPE_HUMAN	634,387614	2438,40033	14489,4702	5541,30853	2448,70545	10887,2219	732,457579	1736,86613	6627,18984
850	850	NVSTGDVNVEMNAAPGVDLTQLLNNMR	958,136	37,11	sp|Q6IFW6|K1C10_RAT	3250,89217	15935,4213	1831,04896	4538,34149	4749,536	1056,66644	359,023463	2776,0763	6422,98633
851	851	NYSPYYNTIDDLKDQ[Dea]IVDLTVGNNK	726,608	37,12	sp|P35527|K1C9_HUMAN	357,706968	193313,505	89022,821	2068,77085	1681,74008	124064,448	694,872717	7665,39547	2004,03292
852	852	HVEDVPAFQALGSLNDLQFFR	801,743	37,17	sp|P25311|ZA2G_HUMAN	16717,5601	2042,62192	5881,04646	34178,2262	39054,3327	2889,36526	3549,92247	1010,41111	2552,37987
853	853	CRM.LALDIEIATYR	660,86	37,31	sp|P19013|K2C4_HUMAN	3295,56723	9497,75238	7600,77016	7814,80528	7404,49562	1512,18132	406,864414	912,654331	2257,43545
854	854	CAM.LGLDIEIATYR	660,863	37,45	sp|O43790|KRT86_HUMAN	3336,68007	2049,1815	7647,83521	1326,98451	7785,00844	7071,40749	396,89127	770,551251	3582,40708
855	855	CAM.LGLDIEIATYR	660,863	37,45	sp|Q9NSB2|KRT84_HUMAN	3336,68007	2049,1815	7647,83521	1326,98451	7785,00844	7071,40749	396,89127	770,551251	3582,40708
856	856	NYAEAKDVFLGMFLYEYAR	767,379	37,46	sp|P02768|ALBU_HUMAN	11786,0675	7003,88355	9342,34989	3551,38911	1986,72942	6084,54146	683,298721	2570,05327	9487,03546
857	857	TDLEM[Oxi]QIEGLKEELAYLR	723,04	37,47	sp|P08779|K1C16_HUMAN	564,518212	1481,55684	1075,60916	169,44918	604,639152	1910,46081	8051,80639	499,898307	665,795587
858	858	LGEHNIDVLEGNE[KXX]QFINAAKIITHPNFNGN	1120,18	37,49	sp|P00761|TRYP_PIG	1432,71725	6354,30908	4478,28907	3901,70832	1897,14603	2344,10967	918,374836	1283,16183	3860,54839
859	859	GGMILSNNLEIGK	673,364	37,54	sp|Q5FMC0|GLYA_LACAC	23534,182	54508,8851	35014,1285	33349,623	41292,8646	580,378713	296,446815	2367,1881	9746,52101
860	860	LGEHNIDVLEGNEQFINAAKIITHPNFNGNTLDN	1112,58	37,62	sp|P00761|TRYP_PIG	35612,5351	39351,5689	13846,7113	4615,26656	8329,09615	1102,62688	759,617734	1052,68625	2735,9357
861	861	CRM.SLDLDSIIAEVK	673,365	37,62	sp|P04264|K2C1_HUMAN	23530,1117	54566,9761	34950,0284	33345,032	41317,6513	573,859473	1450,83893	1569,3482	9852,33615
862	862	PGQ.QLC[CAM]SFQ[Dea]IYEVPWEDR	977,436	37,69	sp|P09228|CYTT_HUMAN	1043,69594	6326,36296	4407,675	4276,08212	2272,77652	3601,86439	4111,5955	2539,39331	4922,04577
863	863	PGQ.QLC[CAM]SFEIYEVPWEN[Dea]R	977,436	37,69	sp|P01037|CYTN_HUMAN	1043,69594	6326,36296	4407,675	4276,08212	2272,77652	3601,86439	4111,5955	2539,39331	4922,04577
864	864	ADLEMQIESLTEELAYLKK	742,053	37,88	sp|Q6IFW6|K1C10_RAT	28851,7833	88165,8926	10110,3706	13193,0459	16005,7847	1885,93315	687,507144	293,281575	1119,30334
865	865	ADLEMQIESLTEELAYLKK	742,053	37,88	sp|P13645|K1C10_HUMAN	28851,7833	88165,8926	10110,3706	13193,0459	16005,7847	1885,93315	687,507144	293,281575	1119,30334
866	866	LGEHNIDVLEGNEQFINAAKIITHPNFNGNT[Dh	933,819	37,98	sp|P00761|TRYP_PIG	3046,01052	2998,27052	5882,36983	3501,05348	7812,73724	3184,84224	2832,05102	3742,39824	2599,38064
867	867	ADLEM[Oxi]QIESLTEELAYLKK	560,791	38,09	sp|Q6IFW6|K1C10_RAT	39729,9545	15511,3828	638,834905	8910,44873	31308,4219	1852,57107	152,985297	152,831266	2388,62958
868	868	ADLEM[Oxi]QIESLTEELAYLKK	560,791	38,09	sp|P13645|K1C10_HUMAN	39729,9545	15511,3828	638,834905	8910,44873	31308,4219	1852,57107	152,985297	152,831266	2388,62958
869	869	LGEHNIDVLEGNEQFINAAKIITHPNFNGNTLDN	803,28	38,18	sp|P00761|TRYP_PIG	25312,6312	91263,9307	137092,538	129429,027	165595,622	415403,54	84591,2687	79756,2964	20786,9153
870	870	LGEHNIDVLEGNEQFINAAKIITHPNFNGNTLDN	795,137	38,41	sp|P00761|TRYP_PIG	8232,39411	9397,79977	5932,01289	25981,2465	6023,44402	1686,21416	576,926146	3900,20974	1744,115
871	871	KKGADVWFKELDINTDGAVNFQEFLILVIKMGV.	873,63	38,42	sp|P05109|S10A8_HUMAN	1277,36676	205,279499	300,415599	841,019385	1025,19787	962,880782	347,423622	1138,94735	1846,69921
872	872	TLVTQNSGVEALIHAILR	645,706	38,42	sp|Q8WNW3|PLAK_PIG	4484,43476	18239,2115	7504,59008	3220,36992	1830,37785	1125,18493	1391,10003	123,671651	1596,17379
873	873	QNLEPLFEQYINN[dAm]LRR	1015,52	38,52	sp|P48668|K2C6C_HUMAN	562,1385	102971,424	47363,6782	347,477791	3516,69103	61810,3504	828,969739	8968,81985	11031,7913
874	874	QNLEPLFEQYINN[dAm]LRR	1015,52	38,52	sp|P02538|K2C6A_HUMAN	562,1385	102971,424	47363,6782	347,477791	3516,69103	61810,3504	828,969739	8968,81985	11031,7913
875	875	QNLEPLFEQYINN[dAm]LRR	1015,52	38,52	sp|P13647|K2C5_HUMAN	562,1385	102971,424	47363,6782	347,477791	3516,69103	61810,3504	828,969739	8968,81985	11031,7913
876	876	VIEHIMEDLDTNADKQLSFEEFIML	994,151	38,55	sp|P06702|S10A9_HUMAN	13700,9931	5753,98506	1283,19047	3914,24473	1065,58122	1180,79644	1071,98164	376,411934	1728,58033
877	877	ADLE[CAM]MQIESLTEELAYLK	718,361	38,57	sp|Q6IFW6|K1C10_RAT	84458,9356	89961,6346	53978,4876	25964,0371	49574,9906	3060,94805	720,430841	4354,94838	34505,9706
878	878	ADLE[CAM]MQIESLTEELAYLK	718,361	38,57	sp|P13645|K1C10_HUMAN	84458,9356	89961,6346	53978,4876	25964,0371	49574,9906	3060,94805	720,430841	4354,94838	34505,9706
879	879	NLDLDSIIAEV	601,319	38,6	sp|P48668|K2C6C_HUMAN	34729,9579	56652,159	44166,9743	54758,5855	44521,0893	39606,905	32486,9321	40489,9606	5851,31252
880	880	NLDLDSIIAEV	601,319	38,6	sp|P19013|K2C4_HUMAN	34729,9579	56652,159	44166,9743	54758,5855	44521,0893	39606,905	32486,9321	40489,9606	5851,31252
881	881	NLDLDSIIAEV	601,319	38,6	sp|P02538|K2C6A_HUMAN	34729,9579	56652,159	44166,9743	54758,5855	44521,0893	39606,905	32486,9321	40489,9606	5851,31252
882	882	NLDLDSIIAEV	601,319	38,6	sp|P13647|K2C5_HUMAN	34729,9579	56652,159	44166,9743	54758,5855	44521,0893	39606,905	32486,9321	40489,9606	5851,31252
883	883	CAM.ADLEMQIESLTEELAYLK	718,361	38,67	sp|Q6IFW6|K1C10_RAT	84467,3353	89987,48	53961,498	25965,4832	49540,125	3049,9332	711,345064	4594,05253	34437,0358
884	884	CAM.ADLEMQIESLTEELAYLK	718,361	38,67	sp|P13645|K1C10_HUMAN	84467,3353	89987,48	53961,498	25965,4832	49540,125	3049,9332	711,345064	4594,05253	34437,0358
885	885	ADLEM[Oxi]Q[Dea]IESLTEELAYLKK	747,718	38,72	sp|Q6IFW6|K1C10_RAT	3440,30877	9914,51267	180389,392	14154,9934	12879,8975	261026,426	78692,585	16683,6611	9928,2756
886	886	LMLLEVISGER	686,898	38,91	sp|Q08043|ACTN3_HUMAN	11079,333	7022,54	1341,2885	1934,71355	1466,08036	547,315178	815,153732	756,226566	1987,46394
887	887	VIEHIMEDLDTNADKQLSFEEFIMLMAR	1113,54	38,95	sp|P06702|S10A9_HUMAN	128385,603	8576,76135	3575,16019	8991,14274	10855,7588	4604,04183	2733,67682	3873,30486	4027,09511

888	888	LDNLQQEIDFLTALYQAELSQMQTQISETNVILS	1161,07	39,19	sp|P04264|K2C1_HUMAN	62109,818	66610,4346	39184,4378	41586,3315	72054,9836	3526,29633	263,784377	485,397737	3815,30317
889	889	ALVLIAFAQYLQQC[CAM]PFEDHVK	830,767	39,22	sp|P02768|ALBU_HUMAN	28913,113	13822,2623	362,283311	13882,5502	4355,78012	1820,23121	1535,10502	440,355305	6572,0107
890	890	FKTIEDLRNKIIAATIENAQPILQIDNAR	1103,61	39,28	sp|P08779|K1C16_HUMAN	8048,87398	8136,15389	580,908417	705,64792	5281,25267	1778,9273	171,343473	226,02379	4546,07888
891	891	TIDD[KXX]LKNQILNLTTDNANILLQIDNAR	1030,86	39,28	sp|P13645|K1C10_HUMAN	16617,7039	26363,4962	737,629924	2412,60474	4006,83573	1173,83422	393,816696	755,519752	2095,81851
892	892	TIDDLKNQILNLTTD[KXX]NANILLQIDNAR	1030,86	39,28	sp|P13645|K1C10_HUMAN	16617,7039	26363,4962	737,629924	2412,60474	4006,83573	1173,83422	393,816696	755,519752	2095,81851
893	893	TIDDLKNQILNLTTD[NaX]NANILLQIDNAR	1025,54	39,28	sp|P13645|K1C10_HUMAN	28150,0849	33858,6135	1263,73168	10240,3323	16206,3758	3823,86344	445,757277	459,089091	296,893101
894	894	NFQEFLILVIK	682,405	39,31	sp|P05109|S10A8_HUMAN	11155,1597	645,135444	1953,58038	3234,58761	3904,5472	14120,5489	1077,13292	1380,04813	8370,78622
895	895	Frm.TIEELQNKILTATVDN[Dea]ANILLQIDNAR	1018,21	39,38	sp|Q04695|K1C17_HUMAN	609951,251	724513,646	387925,407	249089,294	504764,767	2815,42291	1373,72535	619,450712	27315,0915
896	896	SGNTFRPEVHLLPPPSEELALNELVTLTC[CAM]L	894,224	39,52	sp|P01877|IGHA2_HUMAN	1576,16252	2679,83227	1756,21741	2552,03155	21242,5775	3528,84132	1440,958	1386,67799	1846,97347
897	897	SGNTFRPEVHLLPPPSEELALNELVTLTC[CAM]L	894,224	39,52	sp|P01876|IGHA1_HUMAN	1576,16252	2679,83227	1756,21741	2552,03155	21242,5775	3528,84132	1440,958	1386,67799	1846,97347
898	898	TIDDLKNQILNLTTDNANILLQIDNAR	1018,22	39,63	sp|P13645|K1C10_HUMAN	610479,551	724945,749	388092,702	249199,942	505123,611	886793,471	1367,95744	67907,0705	27544,2657
899	899	LGEHNIDVLEGNEQFINAAKIITHPNFNGNTLDN	1124,19	39,66	sp|P00761|TRYP_PIG	146,560751	9027,10186	2970,31818	26720,3894	6301,17317	17370,7915	6461,33417	12131,2238	1895,53144
900	900	NLEPLFETYLSVLR	847,461	39,77	sp|P19013|K2C4_HUMAN	12775,6271	2791,21467	1784,61324	5150,5027	791,323842	508,945826	241,21837	1079,81095	3662,4217
901	901	TIDDLKNQILN[Dea]LTTDNANILLQIDNAR	1018,55	39,82	sp|P13645|K1C10_HUMAN	946430,288	46463,4932	634976,121	390353,687	5816,58036	1355075,29	327818,753	104728,309	8454,15155
902	902	DVFLGMFLYEYAR	812,399	39,9	sp|P02768|ALBU_HUMAN	61745,6417	42969,2862	3049,98104	29838,9862	37549,7253	4615,50218	774,366152	2793,02664	13860,5695
903	903	VLDELTLARADLEM[2Ox]QIESLKEELAYLKK	824,191	40,04	sp|P02533|K1C14_HUMAN	2568,07248	12638,5992	2685,13013	1336,24118	1153,31148	5303,70581	531,38669	2316,45373	3613,21726
904	904	GADVWFKELDINTDGAVNFQEFLILVIKMGVAA	996,917	40,82	sp|P05109|S10A8_HUMAN	12218,9876	477,934734	968,555964	914,699844	1679,08205	1928,17453	319,534018	2014,87584	9856,64432
905	905	ATLVC[CAM]LISDFYPGAVTVAWK	1106,08	41,3	sp|B9A064|IGLL5_HUMAN	817,050658	1139,90975	721,251775	392,440244	10971,3267	1331,54989	530,392899	812,850831	1915,50841
906	906	PGQ.QNLEPLFEQYINN[Dea]LR	937,976	41,34	sp|P48668|K2C6C_HUMAN	13456,9879	45388,8949	27796,8161	24441,6277	6484,50012	28906,9552	1512,51435	5616,41731	4297,72651
907	907	PGQ.QNLEPLFEQYINN[Dea]LR	937,976	41,34	sp|P02538|K2C6A_HUMAN	13456,9879	45388,8949	27796,8161	24441,6277	6484,50012	28906,9552	1512,51435	5616,41731	4297,72651
908	908	PGQ.QNLEPLFEQYINN[Dea]LR	937,976	41,34	sp|P13647|K2C5_HUMAN	13456,9879	45388,8949	27796,8161	24441,6277	6484,50012	28906,9552	1512,51435	5616,41731	4297,72651
909	909	LGEHNIDVLEGNEQFINAAK[Frm]IITHPNFNG	927,151	41,41	sp|P00761|TRYP_PIG	1344,69874	1670,15013	1377,26524	1305,01213	1405,69653	2624,24998	1392,52838	2664,88724	4431,53499
910	910	LDNLQQEIDFLTALYQAE[CAM]LSQMQTQISET	1146,81	41,81	sp|P04264|K2C1_HUMAN	182,395231	21156,4398	1577,13073	2181,83276	3484,57958	1157,63924	625,053996	1032,34329	2821,92101
911	911	QLSFEEFIMLMAR	807,905	41,84	sp|P06702|S10A9_HUMAN	231197,76	126176,278	60260,3055	90483,5595	53860,6488	3008,60385	989,845537	2814,25561	6270,8897
912	912	YC[CAM]VQLSQIQAQISALEEQLQQIR	916,146	41,85	sp|P13645|K1C10_HUMAN	142908,207	391847,412	2377,17003	112360,005	342527,864	2812,79552	312,759825	789,735934	3592,62346
913	913	YC[CAM]MQLAQIQ[Dea]E[Dhy]MIGSVEEQL	907,796	41,94	sp|P02533|K1C14_HUMAN	1147,09867	25650,4641	2174,0591	429,15064	729,524067	1179,93843	579,901932	953,19621	583,230623
914	914	YC[CAM]VQLSQIQSQISALE[NaX]EQLQQIR	696,847	41,97	sp|Q6IFW6|K1C10_RAT	9689,39711	24466,1056	1866,83794	2909,38332	17394,7348	1235,29225	430,125604	615,176919	1848,81275
915	915	YC[CAM]VQLSQIQAQISALE[KXX]EQLQQIR	696,847	41,97	sp|P13645|K1C10_HUMAN	9689,39711	24466,1056	1866,83794	2909,38332	17394,7348	1235,29225	430,125604	615,176919	1848,81275
916	916	YC[CAM]VQLSQIQSQ[Dea]ISALEEQLQQIR	921,818	42,01	sp|Q6IFW6|K1C10_RAT	149,339605	9677,24001	1493,45444	1474,80139	3566,23673	11083,753	581,139399	1000,23216	731,109504
917	917	KELDINTDGAVNFQEFLILVIK	840,46	42,07	sp|P05109|S10A8_HUMAN	605,692767	1989,95982	5363,55562	2444,66596	3736,00694	13838,5603	2765,15182	5233,64246	8186,03444
918	918	VIEHIMEDLDTNADKQLSFEEFIMLMA	1061,51	42,07	sp|P06702|S10A9_HUMAN	876,830114	15717,6639	2466,02486	1290,64492	1823,42449	4340,5306	1706,91634	785,442589	4328,0069
919	919	CAM.YC[CAM]MQLSQIQGLIGSVEEQLAQLR	907,794	42,07	sp|P08779|K1C16_HUMAN	1153,06142	25525,4109	2199,19022	456,64069	739,036943	1091,97842	393,757282	939,634615	1058,4471
920	920	YC[CAM]VQLSQIQ[Dea]S[Dhy]Q[Dea]ISALEE	687,36	42,09	sp|Q6IFW6|K1C10_RAT	3814,30327	31336,493	17233,4839	3607,76245	34202,3423	47758,3026	1135,95275	3057,9587	18208,8218
921	921	NLDLDSIIAEVKAQYEEIAQR	806,758	42,22	sp|P48668|K2C6C_HUMAN	679,45713	154,321908	1729,36964	396,754317	1110,18045	16031,5608	737,193271	685,401909	666,527935
922	922	NLDLDSIIAEVKAQYEEIAQR	806,758	42,22	sp|P02538|K2C6A_HUMAN	679,45713	154,321908	1729,36964	396,754317	1110,18045	16031,5608	737,193271	685,401909	666,527935
923	923	NLDLDSIIAEVKAQYEEIAQR	806,758	42,22	sp|P35908|K22E_HUMAN	679,45713	154,321908	1729,36964	396,754317	1110,18045	16031,5608	737,193271	685,401909	666,527935
924	924	YC[CAM]VQLSQIQSQISALE[KXX]EQLQQIR	700,841	42,23	sp|Q6IFW6|K1C10_RAT	21574,1727	43703,441	12424,6948	1121,99929	48631,0908	30636,5682	319,886801	1784,92287	9343,07262
925	925	YC[CAM]VQLSQIQSQISALEEQLQQIR	921,482	42,25	sp|Q6IFW6|K1C10_RAT	2991,04355	4402,61607	2049,48027	642,354677	1598,80411	3249,23602	1603,98539	1280,88105	5854,13037
926	926	LGEHNIDVLEGNEQFINAAKIITH[AAR]PNFNG	927,153	42,27	sp|P00761|TRYP_PIG	748,826482	393,548061	2103,98912	1561,42516	510,390253	5317,6595	464,170217	215,07529	3257,18323
927	927	KQSLEASLAETEGR[AGA]YC[CAM]VQLSQIQSC	1055,54	42,28	sp|Q6IFW6|K1C10_RAT	6168,58007	1309,36402	4088,51125	3286,51651	5324,14024	9327,18602	1897,23607	594,067908	6023,1982
928	928	CAM.YC[CAM]VQLSQIQ[Dea]S[Dhy]Q[Dea]IS	935,152	42,34	sp|Q6IFW6|K1C10_RAT	734,312983	5424,64349	2476,98518	3885,20487	3965,88625	20419,3604	1019,51827	1646,42058	1993,36529
929	929	ADLEM[Oxi]QIESLTEELAYLK	704,686	42,7	sp|Q6IFW6|K1C10_RAT	18980,4262	28388,7707	18965,8894	10742,9153	11195,7797	2110,42018	274,819778	2335,01956	2128,08908
930	930	VLDPNTVFALVNYISFK	647,355	42,81	sp|P34955|A1AT_BOVIN	4364,26097	8108,41095	8260,57856	3920,83497	6343,97098	5521,8781	1593,1393	3576,04561	8655,22119
931	931	YC[CAM]VQLSQIQ[Dea]S[Dhy]Q[Dea]ISALEE	916,477	42,9	sp|Q6IFW6|K1C10_RAT	4373,57893	27153,5409	12772,9797	7245,28892	14347,8377	5719,05901	2492,13816	1336,78629	994,185645
932	932	YC[CAM]VQLSQ[Dea]IQ[Dea]S[Dhy]Q[Dea]IS	916,474	42,99	sp|Q6IFW6|K1C10_RAT	4363,8934	27085,6718	4603,07698	7295,76926	14343,0443	8121,49563	2587,85258	1387,01761	1024,31687
933	933	LDNLQQEIDFLTALYQAELSQMQTQISE[CAM]T	1150,81	43	sp|P04264|K2C1_HUMAN	14985,2793	19141,9024	5453,19487	7582,89453	16439,87	1496,02119	946,805357	308,763426	153,230522
934	934	GADVWFKELDINT[Dhy]DGAVN[Dea]FQEFLIL	712,227	43,03	sp|P05109|S10A8_HUMAN	1139,17253	2545,44331	3787,7275	3497,21902	3462,6373	1230,98607	3579,28885	4126,6895	6287,83331
935	935	CAM.RKKGADVWFKELDINTDGAVN[Dea]FQ[	779,702	43,04	sp|P05109|S10A8_HUMAN	3608,47174	990,94337	402,176242	527,553616	272,520745	522,3537	1010,64745	411,115873	1128,75532
936	936	GADVWFKELDINTDGAVN[Dea]FQEFLILVIKM	831,099	43,13	sp|P05109|S10A8_HUMAN	4834,85732	15783,2132	392,148758	5433,66689	3777,29617	355,759775	1295,11895	1384,40998	1830,12733
937	937	KIDFSEFLSLLGDIATDYHK	771,401	43,49	sp|P31151|S10A7_HUMAN	2617,09369	1474,67132	3447,68394	13780,7295	13970,4314	6873,35984	2498,78319	2781,07353	2477,91005
938	938	AQFVPLPVSVSVEFAVAATDC[CAM]IAK	840,446	43,74	sp|P12763|FETUA_BOVIN	6770,63399	7807,75301	8002,57684	7059,12988	2280,77217	2136,96088	342,697626	1356,46106	9227,73591
939	939	LDIQGTGQLLFSVVINQLR	705,407	44,46	sp|P01833|PIGR_HUMAN	3840,48247	5487,70356	9856,4104	17673,4198	6943,33438	3699,56143	11471,6742	3272,04225	19417,7112
940	940	ELDINTDGAVNFQEFLILVIK	797,765	44,69	sp|P05109|S10A8_HUMAN	77772,2475	50399,8748	2603,20589	12153,6613	39245,7304	463,903895	1468,43954	12468,4322	6850,8213
941	941	CRM.GADVWFKELDIN[Dea]TDGAVNFQEFLIL	838,256	46,17	sp|P05109|S10A8_HUMAN	2216,74023	3955,0395	3724,94518	6370,12425	2266,31242	3532,30482	1118,4445	1932,95559	733,875286
942	942	GADVWFKELDINTDGAVNFQEFLILVIK	1065,56	46,31	sp|P05109|S10A8_HUMAN	2263,49127	2958,97162	2453,12248	769,846901	1108,73529	4538,18506	1052,96769	1001,13027	6619,03209
943	943	YLGFVQDAATYAVTTFSNVYLFAK	897,125	46,57	sp|P15252|REF_HEVBR	19831,9286	1223,07149	453,099071	394,267495	1234,7266	920,93145	856,105728	375,376807	1842,50528
944	944	AQFVPLPVSVSVEFAVAATDC[CAM]IAKEVVDP	1029,79	47,13	sp|P12763|FETUA_BOVIN	4749,73182	1125,59712	2476,44654	1117,27465	927,937327	661,834809	265,992466	1401,92821	2432,23985
945	945	FNDVGEEHFIGLVLITFSQYLQK	899,805	47,35	sp|P49065|ALBU_RABIT	344,647471	1195,76521	707,934662	1362,55616	674,813041	918,04372	501,14444	7371,16301	245,268544
946	946	LDNLQQEIDFLTALYQAELSQMQTQISE[CAM]T	1146,81	47,89	sp|P04264|K2C1_HUMAN	549,040335	11849,4199	586,096029	648,151907	856,44362	2330,92769	730,311519	807,236875	751,930559
947	947	PGQ.QLSFEEFIMLMAR	799,392	48,01	sp|P06702|S10A9_HUMAN	94148,8067	47932,5819	377,42442	82911,0035	32151,7442	6129,27596	58247,5224	18204,8972	25551,1202
